# Construction of C2-indolyl-quaternary centers by branch-selective allylation: enabling concise total synthesis of the (±)-mersicarpine alkaloid†

**DOI:** 10.1039/d3sc04732f

**Published:** 2023-12-18

**Authors:** Minakshi Ghosh, Samrat Sahu, Shuvendu Saha, Modhu Sudan Maji

**Affiliations:** a Department of Chemistry, Indian Institute of Technology Kharagpur Kharagpur 721302 WB India msm@chem.iitkgp.ac.in

## Abstract

Herein we report a branch-selective allylation strategy for accessing C2-indolyl-all-carbon quaternary centers using allylboronic acids. This approach boasts broad functional group tolerance, scalability, and relies on easily accessible allyl alcohol precursors. Importantly, the C3-position of the indole remains free, offering a handle for further synthetic refinement. Mechanistic pathways, corroborated by density functional theory (DFT), suggest the involvement of an indolenine intermediate and a Zimmerman–Traxler-like transition state during allylboration. Demonstrating its efficacy, the method was applied to the total synthesis of the (±)-mersicarpine alkaloid and enabled formal synthesis of additional alkaloids, such as (±)-scholarisine G, (±)-melodinine E, and (±)-leuconoxine.

## Abstract 2

Herein we report a branch-selective allylation strategy for accessing C2-indolyl-all-carbon quaternary centers using allylboronic acids and implemented this method for the total synthesis of the mersicarpine alkaloid.

## Graphical abstract

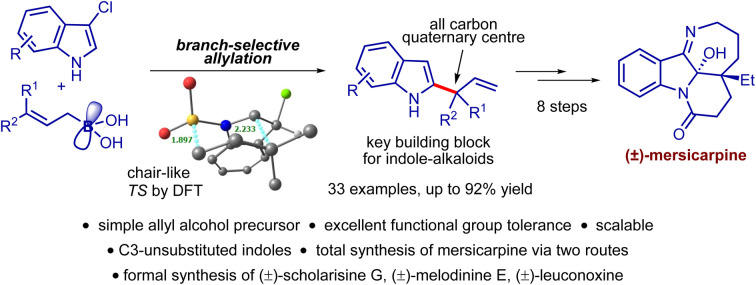

## Introduction

**Scheme 1:**
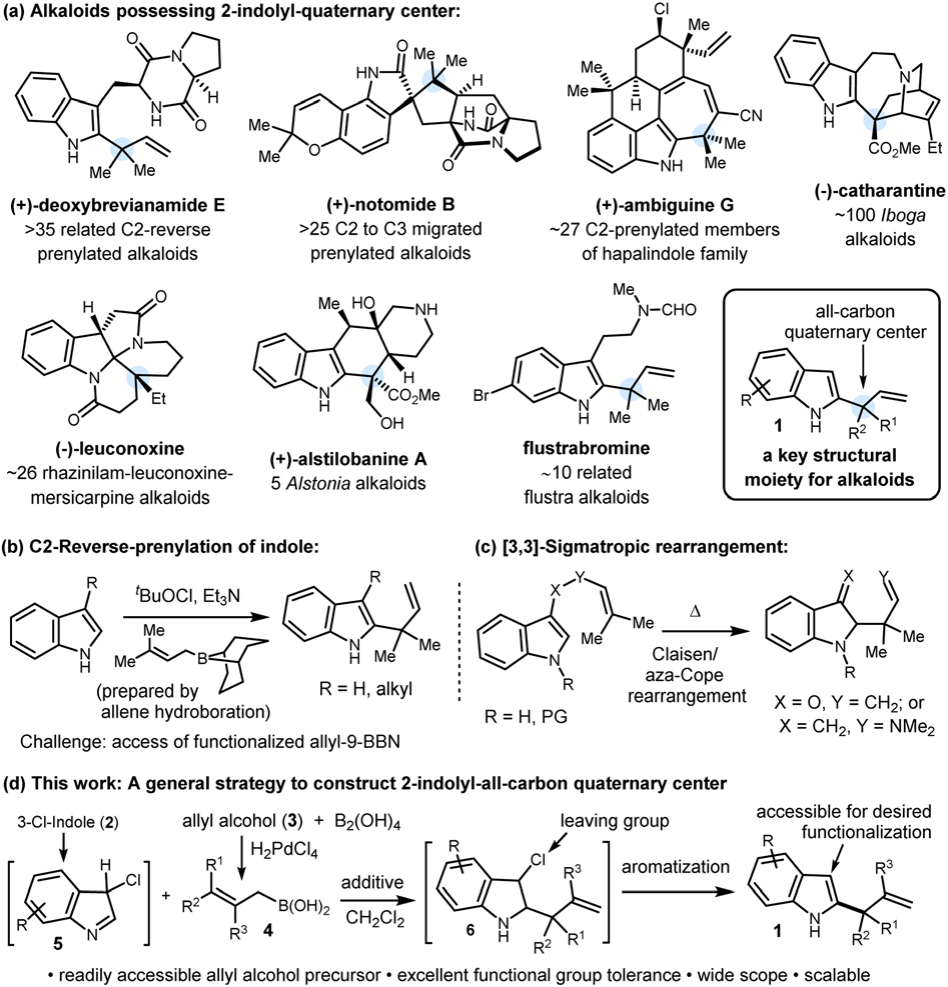
(a) Representative alkaloids possessing structural moiety 1. (b and c) Literature reports for the construction of a 2-indolyl quaternary center. (d) Reverse allylation using allylboronic acids.

The construction of all-carbon quaternary stereocenters remains a formidable challenge in organic synthesis due to issues of steric hindrance, regioselectivity, and the frequent incompatibility with existing functional groups. Quaternary centers possessing one 2-indolyl group and a two-carbon moiety serve as a linchpin for several alkaloids, particularly indole terpenoids (Scheme 1a).^1^ This key structural moiety (1) can ideally be accessed by the branch-selective indole C2-allylation. Despite numerous applications and future prospects in total synthesis, this reaction is still underdeveloped, and to our knowledge, Danishefsky's reverse prenylation is the only method known to date (Scheme 1b).^2*a*^ This method has played a key role in the late-stage reverse prenylation to access several C2-reverse-prenylated indole alkaloids of diverse chemical structures displaying a broad spectrum of biological activities.^3^ Extension of this method to other functionalized γ,γ-disubstituted-allyl-9-BBN reagents to access such all-carbon quaternary centers did not progress further which might stem from the fact that these are prepared *via* hydroboration of allenes or borylation of allyl-lithium reagents.^4^ These precursors are either difficult to access or key allylic C–B bond formation reactions are incompatible with common functional groups. This long-standing problem can be addressed by developing a unique and general reverse-allylation strategy by employing readily accessible allylating agents.

Although indole allylation chemistry has drawn considerable attention over the past few decades, the studies mostly focused on linear allylation. Transition-metal-catalyzed C–H allylation,^5*a*–*c*^ acid catalyzed or mediated allylation,^5*d*,*e*^ and lithiation of indole followed by allylation^5*f*^ are some representative methods of linear allylation where indole plays the role of a nucleophile. In sharp contrast, branch-selective allylation using an Umpolung strategy where indole plays an electrophilic part is highly challenging and elusive in the literature. Besides Danishefsky's reverse prenylation reaction,^2*a*^ [3,3]-sigmatropic rearrangement has been utilized to access reverse allylated products, and as a part of the method, the products contain specific substitution at the C3-position of indole (Scheme 1c).^2*b*,*c*^ Thus a strategy which can provide direct access to several functionalized C2-indolyl-all-carbon quaternary centers 1 while the C3-position is free for further synthetic manipulation is challenging and highly desirable, particularly considering the total synthesis of indole alkaloids.

In recent years, allylboronic acid has emerged as a potent allylating agent of carbonyls and imines to generate adjacent quaternary stereocenters.^6*a*,*b*^ Easy accessibility, geometrical stability, high reactivity, and functional group tolerance are the key aspects of allylboronic acids.^6*c*^ Recently, indole C2-reverse allylation with allyl-trifluoroborate salts and allylboronic acids has been accomplished; with their own merits, these protocols led to indoline formation.^7^ In continuation of our interest in the synthesis of alkaloids and developing new reactions using allylboronic acids,^8^ herein, we report a mild, catalyst-free indole-C2-reverse-allylation to construct 1 employing easily accessible 3-chloroindoles as electrophiles and a wide range of functionalized γ,γ-disubstituted allylboronic acids as nucleophiles (Scheme 1d). The application of this newly developed method is also demonstrated in the total synthesis of (±)-mersicarpine and formal synthesis of (±)-scholarisine G, (±)-melodinine E, and (±)-leuconoxine indole alkaloids.

## Results and discussion

We hypothesize that, like indole, 3-chloroindole 2 can undergo protonation at the C3-position to eventually produce an imine intermediate 5 which upon *in situ* reaction with an allylboronic acid should furnish allylated indoline 6. Finally, aromatization should occur under redox-neutral conditions through the elimination of HCl leading to the formation of the desired C2-reverse allylated indoles 1 (Scheme 1d). To achieve our goal, we started investigating several reaction parameters by employing 3-chloroindole 2a as an electrophile and prenylboronic acid 4a as a nucleophilic allylating agent. As allylboronic acids are known to generate an indolenine intermediate from indoles,^6*b*^ we initiated our study without employing any Brønsted acid additive. Among different screened solvents such as toluene, ethyl acetate, ether, and dichloromethane, the last one was found to be the most suitable providing a 1 : 1 mixture of 1aa and 7a in 67% yield (Table 1, entry 1). The formation of 7a can be explained by two mechanisms, firstly, 6 may be aromatized in the presence of molecular oxygen as an oxidant. In a second route, the product 1aa may undergo successive chlorination by the *in situ* generated 3-Cl-indolenine intermediate 5 which can potentially act as a chlorinating agent.^9*a*^

**Table 1:** Optimization of reaction conditions^a^

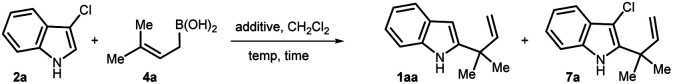				
Entry	Additive/equiv of additive	Time (h)	Yield^b^ (%)	Ratio [1aa : 7a]^c^
1	No additive/–	10	67	1 : 1
2	2,6-Di-^*t*^BuC_6_H_3_OH/3.0	12	64	4 : 1
3	3-Methoxyphenol/3.0	12	62	1.7 : 1
4	Resorcinol/3.0	12	57	1.66 : 1
5	1,3,5-(MeO)_3_C_6_H_3_/3.0	12	42	6 : 1
6	*N*,*N*-dimethylaniline/3.0	12	78	>20 : 1
7	*N*,*N*-dimethylaniline/2.0	24	65	>20 : 1
**8**	***N*,*N*-dimethylaniline/1.0**	**24**	**83**	**>20 : 1**
9	*N*,*N*-dimethylaniline/0.5	48	53	3 : 1
10^d^	*N*,*N*-dimethylaniline/1.0	36	51	>20 : 1
11^e^	*N*,*N*-dimethylaniline/1.0	24	79	>20 : 1
12^f^	*N*,*N*-dimethylaniline/1.0	24	61	>20 : 1
13^g^	*N*,*N*-dimethylaniline/1.0	24	0	—
14^h^	*N*,*N*-dimethylaniline/1.0	24	81	>20 : 1
15^i^	*N*,*N*-dimethylaniline/1.0	24	—	—

a Reaction conditions: 2a (0.2 mmol, 1.0 equiv), 4a (0.4 mmol, 2.0 equiv), additive, CH_2_Cl_2_ (2 mL), 80 °C, Ar.

b Isolated yield.

c Determined from the crude ^1^H NMR analysis.

d At 60 °C.

e At 100 °C.

f 1.0 equiv of allylboronic acid was used.

g Prenyl-BPin (0.4 mmol, 2.0 equiv) was used as an allylating agent.

h 3-Bromoindole (0.2 mmol, 1.0 equiv) was used instead of 2a.

i 3-Iodoindole (0.2 mmol, 1.0 equiv) was used instead of 2a.

We postulated that, if the second route is operative, then formation of 7a can be suppressed by introducing an external electron-rich aromatic as a Cl^+^ scavenger^9*b*^ or a base which can control the generation of 5 by neutralizing *in situ* generated HCl. Pleasingly, 2,6-di-^*t*^BuPhOH additive improved the product ratio to 4 : 1 (entry 2). Use of other additives, such as 3-methoxyphenol, resorcinol, and 1,3,5-trimethoxybenzene, was not very effective (entries 3–5). Interestingly, *N*,*N*-dimethylaniline was found to be a superior additive almost suppressing the formation of 7a and providing 1aa in 78% yield (entry 6). 1.0 equiv of this additive gave the best results (entry 8) as reducing the amount of additive further led to a decrease in yield and product selectivity (entries 7 to 9). A decrease, as well as an increase in the reaction temperature, diminished the yield of the reaction, although the product ratio remained unaltered (entries 10–11). Lowering the equivalency of allylboronic acid was not effective as yield decreased (entry 12). When the nucleophile was changed to prenyl-pinacol boronate ester, no allylated product was detected, highlighting the superior reactivity profile of allylboronic acid (entry 13). Next, the suitability of 3-bromo- and 3-iodoindole as electrophiles was tested in place of 2a. Pleasingly, 3-bromoindole was found to be highly effective for this reverse-prenylation reaction providing 1aa in 81% yield (entry 14). However, 3-iodoindole failed to provide 1aa (entry 15).

**Scheme 2:**
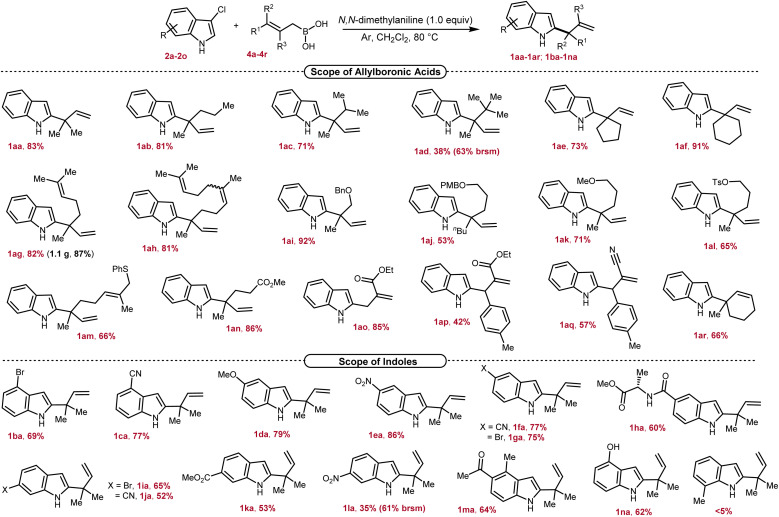
Scope of allylboronic acids and indoles^*a*^. ^*a*^Reaction conditions: 3-Cl-indole 2 (0.2 mmol, 1.0 equiv), allylboronic acid 4 (0.4 mmol, 2.0 equiv), *N*,*N*-dimethylaniline (0.2 mmol, 1.0 equiv), CH_2_Cl_2_ (2 mL), 80 °C, under Ar; isolated yield.

To probe the generality of our method, we first explored the scope of allylboronic acids of diverse structures and bearing different functional groups under the optimized conditions (Scheme 2). Allyl boronic acids having linear and branched alkyl substitutions at the γ,γ-position efficiently reacted to provide 1aa–1ad possessing the desired 2-indolyl-quaternary-center in good to excellent yields (38–83%). The decrease in yield with increased branching at the α-position of the newly generated quaternary center could be explained by the increased steric congestion. Nonetheless, even the *tert*-butyl group in 1ad can also be introduced as a substitution at the quaternary center albeit in low isolated yield. The rigid γ,γ-cyclic boronic acids of varied chain lengths smoothly reacted to furnish 1ae–1af in 73–91% yields. Both geranyl and farnesyl boronic acids underwent smooth conversion and gave the corresponding allylated products in excellent yields (1ag–1ah, 81–82%). Pleasingly, γ,γ-disubstituted allylboronic acids bearing ether (–OBn, –OPMB, –OMe), sulfonate (–OTs), thioether (–SPh) and ester (–CO_2_Me) functional groups were successfully tolerated under the reaction conditions to provide the desired products in good to excellent yields (1ai–1an, 53–92%). Allylboronic acids with substitutions at the β- and γ-positions were next tested, and to our delight, allylated products 1ao–1aq containing ester and cyanide functional groups were isolated in 42–85% yields. This proves that our developed method can also be applicable for accessing secondary as well as tertiary centers at the C2-position. To our delight, the secondary boronic acid also underwent smooth conversion and furnished the corresponding allylated product 1ar in 66% yield. The compatibility of key functional groups is another strength of this protocol as they could be utilized for further downstream modifications in organic synthesis.

We further investigated the scope of electrophiles by incorporating different substitutions on the benzene ring of indole, revealing pronounced trends in reactivity and selectivity. 4-Bromo and -cyano containing 3-chloroindoles underwent smooth conversion to furnish the desired allylated products 1ba–1ca in 69–77% yields (Scheme 2). Remarkably, the reaction appears largely insensitive to the electronic nature of the substituents at the C5-position, as both electron-donating and withdrawing groups such as methoxy, nitro, cyanide, and bromide furnished desired products 1da–1ga in comparable yields (75–86%). Notably, alanine coupled indole-5-carboxylic acid was also well tolerated, providing 1ha in 60% yield. Further probing revealed that 3-chloroindoles with bromo, cyano, ester, and nitro groups at the C6-position were amenable substrates, albeit with slightly diminished yields (1ia–1la, 35–65%). Being a reactive functional group toward allyl-boronic acid,^6*a*^ to our joy, a ketone also survived under the reaction conditions to furnish 1ma in 64% yield. Indole containing a free-hydroxyl group also participated in this reaction providing 1na in 62% yield. Next, the effect of substitution closer to the coordinating imine nitrogen was tested which revealed that, due to sensitivity to spatial constraints, 3-chloro-7-methylindole failed to react under our optimized conditions. Next, the scalability of the developed protocol is demonstrated by carrying out the reaction on a 5.0 mmol scale by employing geranyl boronic acid 4g, and pleasingly 1.1 g of 1ag was isolated, recording a yield of 87%. In sum, our method offers a nuanced landscape of both opportunities and limitations for targeted molecule synthesis.

**Scheme 3:**
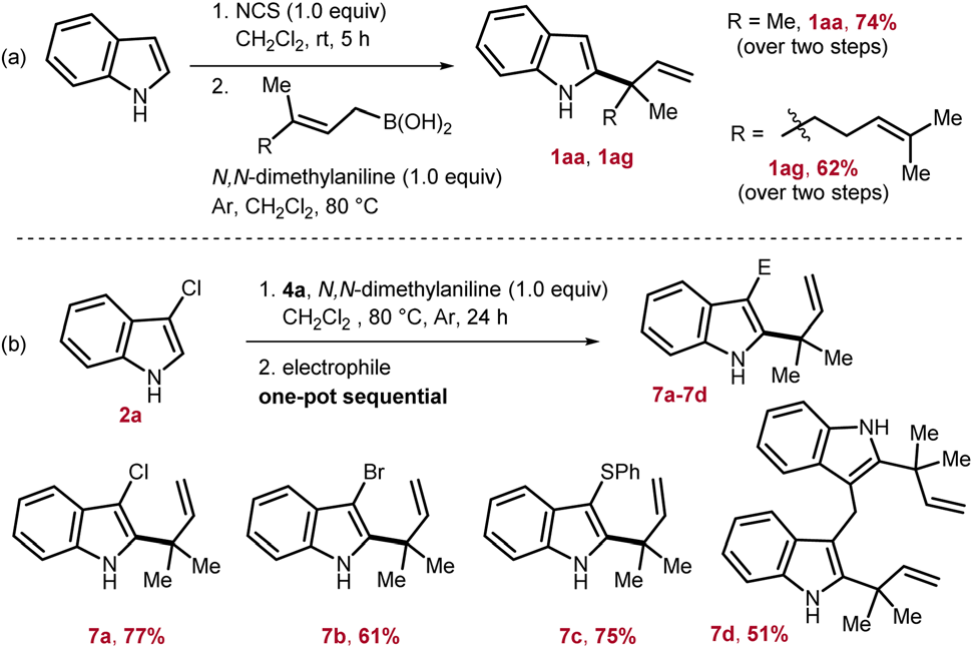
(a) One-pot allylation from indole. (b) One-pot sequential indole 2,3-difunctionalization.

To access reverse-allylated products directly from indole, a one-pot two step strategy is adopted. Here, first, indole was subjected to chlorination by employing *N*-chlorosuccinimide, and then, subsequently *in situ* generated 2a was reacted with the allyl-boronic acids. Through this one-pot strategy, 1aa and 1ag were isolated in 62–74% yields from indole (Scheme 3a). Next, considering the applicability of the allylated products 1, the nucleophilicity of the indole nucleus was further exploited by *in situ* reacting with various electrophiles. This would lead to a formal one-pot indole 2,3-difunctionalization in an elegant way and can potentially furnish valuable intermediates for total synthesis. To this end, first 1aa was generated by reacting 2a with 4a, and then, in one pot sequentially treated with an appropriate electrophile to access 7. Through this strategy, chlorination, bromination, sulfenylation, and also the formation of bis(indolyl)methane were successfully achieved to furnish 7a–7d in 51–77% yields (Scheme 3b).

**Scheme 4:**
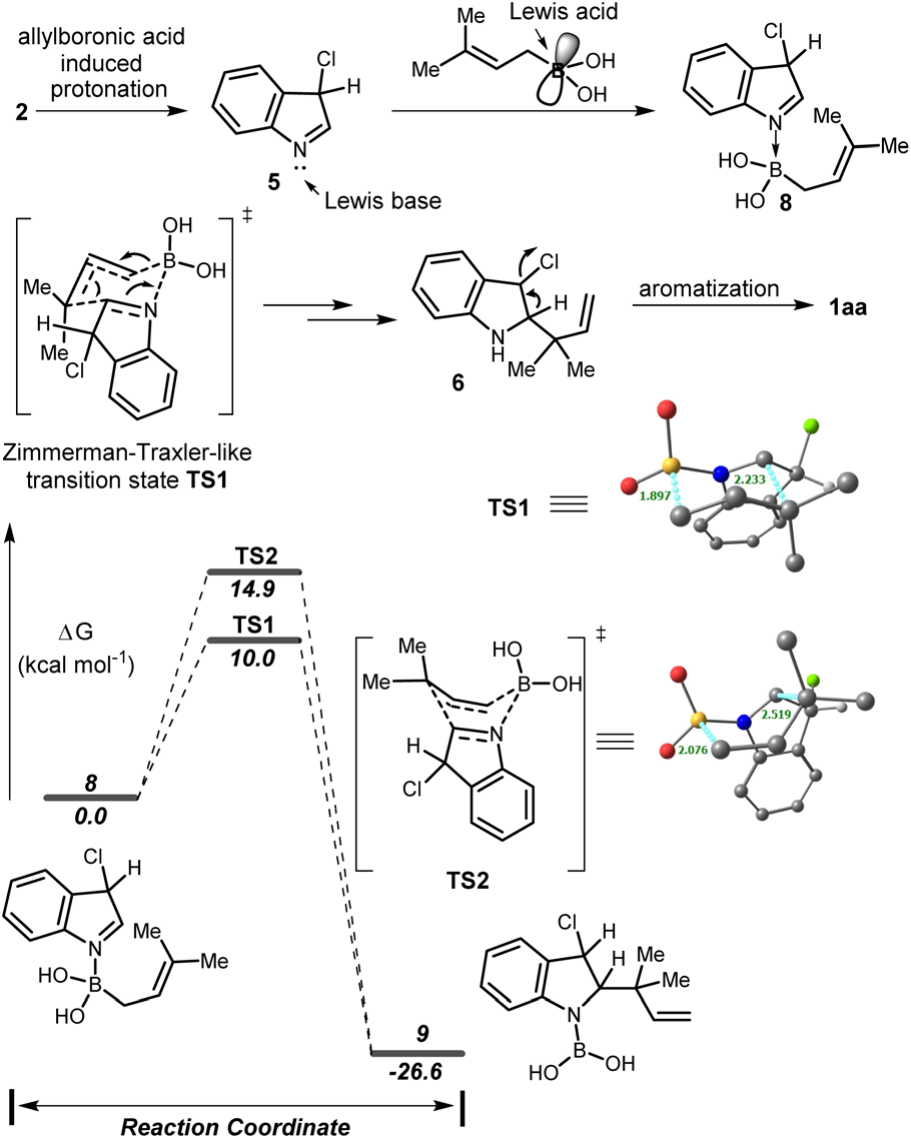
Plausible mechanism of reverse allylation and relative Gibbs free energy profile at the B3LYP(SMD)/Def2-TZVP//B3LYP/TZVP level.

Based on the previous report^7*b*^ we proposed that, initially, 3-chloroindole 2 underwent allylboronic acid induced tautomerization resulting in an indolenine intermediate 5 which coordinates with the electrophilic boron center of the allyl boronic acid to generate the adduct 8. Subsequently, intermediate 8 was transformed into an allylated indoline 6 through the Zimmerman–Traxler-like chair TS. Finally, aromatization provided the desired product 1aa. To support our hypothesis, the DFT calculations were performed using the B3LYP(SMD)/Def2-TZVP//B3LYP/TZVP level of theory. The allylboration proceeds *via* the chair-like transition state TS1 with a low activation barrier (10.0 kcal mol^−1^) compared to the corresponding boat-like transition state TS2 (14.9 kcal mol^−1^) providing intermediate 9 with a significant stabilization in energy. It is also well known that the boat form is unfavourable due to 1,4-diaxial strain. The bond distances in complex 8 [N–B, 1.68 Å & B–C, 1.64 Å], chair TS [N–B, 1.53 Å; B–C, 1.90 Å; & C–C, 2.23 Å] and complex 9 [N–B, 1.43 Å & C–C, 1.57 Å] suggested that B–C bond cleavage and C–C bond formation occurred through a concerted pathway and the product formation in this sequence was a thermodynamically downhill process (Scheme 4).

**Scheme 5:**
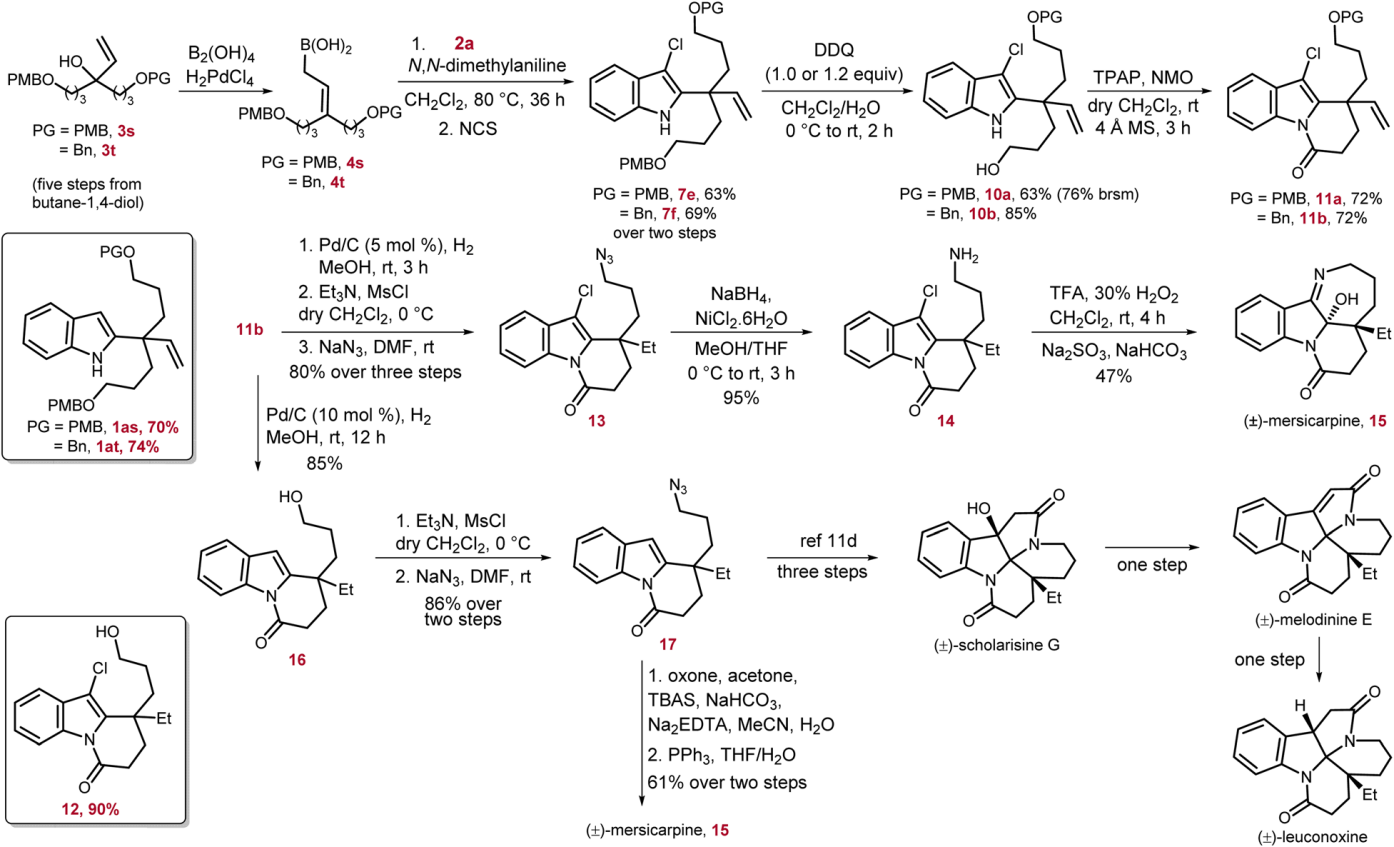
Total synthesis of (±)-mersicarpine and formal synthesis of (±)-scholarisine G, (±)-melodinine E, and (±)-leuconoxine alkaloids.

Facile generation of the indolyl-C2-quaternary center prompted us to apply this newly developed method to the total synthesis of indole-based alkaloids (±)-mersicarpine, (±)-scholarisine G, (±)-melodinine E, and (±)-leuconoxine. These alkaloids belong to the leuconolam–leuconoxine–mersicarpine triads, a subfamily of aspidosperma alkaloids, among 2000 known monoterpene indole alkaloids.^10^ Their unprecedented structural features, significant biological activities, and interesting biosynthetic relationships made them an attractive target for total synthesis.^1*h*,11,12^ A detailed overview of these polycyclic alkaloids suggested that despite having different ring connectivity they share a common core having an all-carbon quaternary center at the C2-position of the indole ring. Starting from indole derivatives, this key quaternary center is generated mainly by intramolecular radical,^12*b*–*d*^ Pd-catalyzed reductive Heck,^12*e*^ and cationic cyclization^12*f*^ of indole tethered alkenyl or alcohol moieties. In another strategy, the indole ring is constructed at a later stage on the properly assembled advanced precursors.^11*a*–*e*,12*g*,*h*^ In our approach, we aim to generate 1 through a more challenging intermolecular amalgamation of the indole moiety and properly designed allyl unit possessing the required functional groups (Scheme 5).

Mersicarpine was chosen as the first target for this venture owing to its intriguing structural features consisting of three heterocycles *i.e.*, indoline, seven-membered cyclic imine, and δ-lactam, fused with each other around a hemiaminal group adjacent to the quaternary carbon center. In addition, in 2021, Usui and co-authors studied the biological activity of mersicarpine on the human leukemia cell line HL60, and the results suggested that it is a novel protein translation inhibitor.^13^ In our retrosynthetic analysis, along with vinyl substitution which will provide the ethyl group after hydrogenation, two more terminally functionalized three-carbon moieties were required to construct the cyclic imine and δ-lactam ring.

At the outset of the synthesis of mersicarpine, we devised a rapid synthetic route to access the key tertiary alcohol 3s, having two 4-methoxy-benzyl (PMB) protected three-carbon units, from commercially available 1,4-butanediol.^14^ The desired γ,γ-disubstituted allyl boronic acid 4s was readily prepared from 3s which upon treatment with 3-chloroindole 2a under the optimized conditions resulted in the desired indole derivative 1as possessing a quaternary center in 70% yield. Our attempts to selectively remove one of the PMB groups was unsuccessful as treatment of 1as with DDQ resulted in a complex reaction mixture. We speculated this issue might have arisen due to the reaction of electron-rich and highly nucleophilic 1as and oxidized side product of the PMB protecting group or DDQ itself. To prevent this, 1as was converted to 7e in one pot in 63% yield. To our delight, 7e underwent selective mono PMB-deprotection to furnish 10a in 63% overall yield, and 10% of diol by removal of both PMB groups was also isolated. The alcohol 10a was subjected to Ley oxidation conditions to construct the required δ-lactam ring by an oxidation–cyclization–oxidation sequence to obtain 11a in 72% yield. As the selective mono-deprotection of the PMB group in 7e was relatively low yielding and less selective, to increase the efficiency of our synthesis, one PMB group was replaced by a benzyl group in 3t. The same reaction sequence was then repeated with 3t to reach the intermediate 7f with a slight increase in overall yields. The selective mono-PMB deprotection of 7f proceeded smoothly and produced 10b with much higher yield of 85%, and no diol was detected. The δ-lactam ring formation on 10b also occurred efficiently to provide 11b in 72% yield. The intermediate 11b has four functional groups, namely, alkene, OBn group, C–Cl bond and indole nucleus, which can potentially be reduced under hydrogenation conditions. To achieve the total synthesis of mersicarpine, first alkene and OBn groups were selectively hydrogenated by 5 mol% Pd/C and running the reaction over 3 h to afford 12 in 90% yield. Interestingly, another key intermediate 16 was also obtained in 85% yield by increasing the Pd/C catalyst loading to 10 mol% and prolonging the reaction time to 12 h. Of note, increasing the catalyst loading further in order to decrease the reaction time led to over-reduction of 16 to the corresponding indoline product. Although the intermediate 12 was isolated and then subjected to mesylation followed by azidation, the same azide product 13 was also synthesized from 11b by a three step protocol in 80% overall yield. The reduction of azide to amine was extremely facile to produce 14 in 95% yield. Finally, the total synthesis of (±)-mersicarpine 15 was accomplished in 47% yield by oxidation of 14 with a mixture of trifluoroacetic acid and hydrogen peroxide followed by imine formation at an appropriate pH.^12*f*^

The total synthesis of mersicarpine was also achieved *via* a second strategy starting from intermediate 16. In this route, first the alcohol functional group was mesylated and converted to azide 17. Following Wang's route, treatment of 17 with dimethyl dioxirane, prepared *in situ* from acetone and oxone, followed by PPh_3_-mediated Staudinger–aza-Wittig reduction facilitated the intramolecular cyclization to furnish (±)-mersicarpine in 61% yield over two steps.^11*d*^ The NMR spectrum of mersicarpine was recorded in base-washed CDCl_3_ due to the presence of an acid sensitive hemiaminal group. The intermediate 17 has been used as an advanced synthetic intermediate for the total synthesis of other leuconoxine family alkaloids by Wang *et al.* Thus, the formal synthesis of (±)-scholarisine G, (±)-melodinine E, and (±)-leuconoxine was also achieved.

## Conclusions

We developed an indole C2-reverse allylation strategy for the construction of an indolyl-C2-quaternary center employing allylboronic acids under mild conditions starting from readily available 3-chloroindole derivatives. The reaction shows excellent functional group tolerance as a wide range of allyl boronic acids and indoles took part in this reaction. As allylboronic acids can be accessed from simple allyl alcohol starting precursors, this method holds enormous potential for the total synthesis of indole-based alkaloids. According to our hypothesis, the nucleophilic addition of allylboronic acids occurred on the 3*H*-indole-imine tautomer. DFT calculations were carried out to support the proposed six-membered chair-like TS (Zimmerman–Traxler model) for the allylation step. The applicability of this strategy is demonstrated by total synthesis of the (±)-mersicarpine alkaloid *via* two routes in a divergent way from 3-chloroindole. Moreover, this strategy is also applied to the formal synthesis of other *Aspidosperma* alkaloids, such as (±)-scholarisine G, (±)-melodinine E, and (±)-leuconoxine, from a common intermediate.

## Data availability

The detailed experimental procedures, compound characterization data and related spectra are provided in the ESI.†

## Author contributions

M. G. and S. Sahu performed all the experiments in the laboratory. All the authors contributed to the design of the experiments, discussion, and preparation of the manuscript. S. Saha performed the DFT calculations.

## Conflicts of interest

There are no conflicts to declare.

## Supplementary Material

SC-015-D3SC04732F-s001

## References

[cit1] Li S.-M. (2010). Nat. Prod. Rep..

[cit2] Schkeryantz J. M., Woo J. C. G., Siliphaivanh P., Depew K. M., Danishefsky S. J. (1999). J. Am. Chem. Soc..

[cit3] Baran P. S., Maimone T. J., Richter J. M. (2007). Nature.

[cit4] Kramer G. W., Brown H. C. (1977). J. Organomet. Chem..

[cit5] Mishra N. K., Sharma S., Park J., Han S., Kim I. S. (2017). ACS Catal..

[cit6] Raducan M., Alam R., Szabó K. J. (2012). Angew. Chem., Int. Ed..

[cit7] Nowrouzi F., Batey R. A. (2013). Angew. Chem., Int. Ed..

[cit8] Sahu S., Das B., Maji M. S. (2018). Org. Lett..

[cit9] Chen T., Foo T. J. Y., Yeung Y.-Y. (2015). ACS Catal..

[cit10] Hájíček J. (2011). Collect. Czech. Chem. Commun..

[cit11] Xu Z., Wang Q., Zhu J. (2013). J. Am. Chem. Soc..

[cit12] Kam T.-S., Subramaniam G., Lim K.-H., Choo Y.-M. (2004). Tetrahedron Lett..

[cit13] Shiobara T., Nagumo Y., Nakajima R., Fukyama T., Yokoshima S., Usui T. (2021). Biosci., Biotechnol., Biochem..

[cit14] See ESI† for details

