# Supplementary material for: Construction of C2-indolyl-quaternary centers by branch-selective allylation: enabling concise total synthesis of the (±)-mersicarpine alkaloid
Source: Chem Sci. 2023 Dec 18;15(5):1789–95. doi: 10.1039/d3sc04732f (PMC10829033; doi:10.1039/d3sc04732f)
Supplement: SC-015-D3SC04732F-s001 [file SC-015-D3SC04732F-s001.pdf]

Electronic Supplementary Material (ESI) for Chemical Science.
This journal is © The Royal Society of Chemistry 2024

Construction of C2-indolyl-quaternary center by branch-selective allylation: enabling concise total synthesis of the (±)-mersicarpine alkaloid

Minakshi Ghosh, Samrat Sahu, Shuvendu Saha and Modhu Sudan Maji*

Department of Chemistry, Indian Institute of Technology Kharagpur, Kharagpur-721302, India.

Email: msm@chem.iitkgp.ac.in

**Table of Contents:**	**Page No.**
**Experimental Section:**	**S1**
**General Procedure (GP I) for the Synthesis of 3-Chloroindoles and characterization data (2f, 2h, 2j-2m, 2o)**	**S2-S4**
**Preparation of the Allylboronic Acids 4**	**S4-S5**
**General Procedure (GP III) for the Branch-Selective Indole C2-Allylation and Compound Characterization Data (1aa-1ar, 1ba-1na)**	**S5-S17**
**One-Pot Synthesis of Reverse-Allylated Products from Unsubstituted Indole**	**S17**
**General Procedure (GP IV) for One-Pot Sequential Electrophilic Substitution and Compound Characterization Data (7a-7d)**	**S17-S19**
**Total Synthesis of (±)-Mersicarpine and Related Compound Characterization Data**	**S20-S32**
**Comparison of NMR Data of Mersicarpine 15**	**S33-S34**
**Computational Studies**	**S35-S37**
**References**	**S38**
**^{1}H and ^{13}C NMR Spectra of all compounds and HPLC data of 1ha**	**S39-S155**

Experimental Section:

General: All reactions involving air or moisture-sensitive reagents were carried out in flame-dried glassware under nitrogen or argon atmosphere. All solvents were obtained from Merck India and dried according to the standard literature procedure. All the solvents used to conduct reactions were degassed by purging argon gas under sonication for 1 hour. Reactions were monitored by thin layer chromatography (TLC) using Merck silica gel 60 F254 pre-coated plates (0.25 mm), and visualized under UV light or dipping into $KMnO_4$ or p-anisaldehyde stain. Silica gel (particle size 100-200 mesh) were purchased from SRL India for performing column chromatography by using a mixture of hexanes and ethyl acetate or CH_2Cl_2 and MeOH eluent. The ^{1}H NMR spectroscopic data were recorded with a Bruker 400, 500 or 600 MHz NMR instruments. ^{13}C NMR spectra were similarly recorded at 101, 126 or 151 MHz NMR instruments by broadband decoupled mode. Proton and carbon NMR chemical shifts (δ) are reported in parts per million (ppm) relative to residual proton or carbon signals in $CDCl_3$ (δ = 7.26, 77.16) or DMSO-d_6 (δ = 2.50, 39.52). Coupling constants (J) are reported in Hertz (Hz) and refer to apparent multiplicities. The following abbreviations are used for the multiplicities: s: singlet, d: doublet, t: triplet, q: quartet, quint: quintet, sep: septet, dd: doublet of doublets, dt: doublet of triplets, dq: doublet of quartets, ddd: doublet of doublet of doublets, td: triplet of doublets, m: multiplet, br: broad. Infrared (IR) spectra were recorded by Perkin Elmer ATIR spectrometer, and reported in terms of wave number (cm^{-1}). High-resolution mass spectrometry (HRMS) spectra were recorded by the electrospray ionization (ESI) (+Ve) method using a time-of-flight mass analyzer. Optical rotations were measured on a Rudolph Autopol II Automatic Polarimeter and have the unit deg cm^3 dm^{-1} g^{-1}. Concentration in bracket is given in g/100 mL. The enantiomeric excesses were determined by HPLC analysis using a chiral stationary phase (column, CHIRALPACK ID; eluent: n-hexane/2- propanol). The chiral HPLC methods were calibrated with the corresponding racemic mixtures.

General Procedure (GP I) for the Synthesis of 3-Chloroindoles:

All the 3-chloroindole derivatives were synthesized according to the known literature procedures.[1]

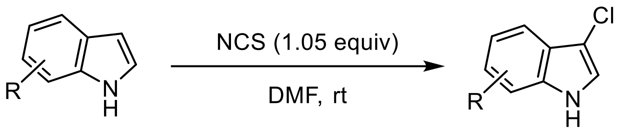

2a-2o

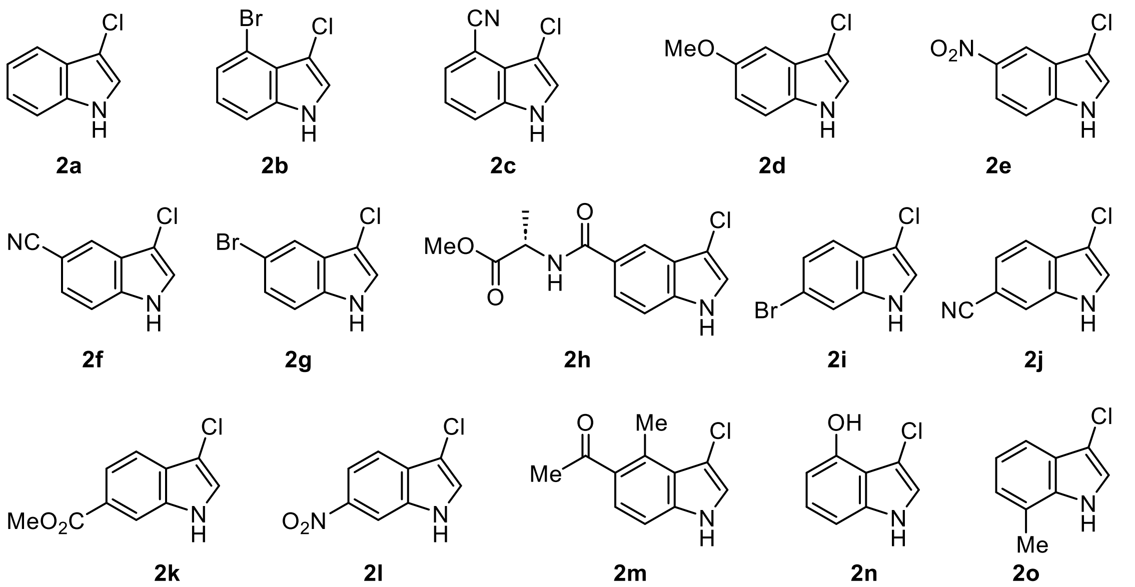

To a solution of indole derivatives (1.0 mmol, 1.0 equiv) in DMF (5 ml), *N*-chlorosuccinamide (NCS, 140 mg, 1.05 mmol, 1.05 equiv) was added and stirred at room temperature in a dark place. After completion of the reaction as indicated by TLC (1 to 2 hours), brine (10 mL) was added to the reaction mixture, and the aqueous layer was extracted with ethyl acetate (3 × 15 mL). The combined organic layers were washed with cold water (2 × 15 mL), dried over anhydrous Na_2SO_4 and evaporated under reduced pressure. The crude product was purified by column chromatography on silica gel using ethyl acetate in hexane as eluent to afford the desired 3-chloroindoles **2a-2o**.

The analytical data of 3-chloroindoles **2a-2e**, **2g**, and **2i** were matched with their reported data.[1]

3-Chloro-1*H*-indole-5-carbonitrile (2f):

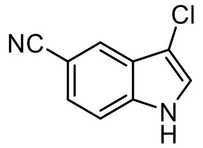

The titled compound **2f** was synthesized according to **GP I** and isolated as a white solid after column chromatography on silica gel using 15% ethyl acetate in hexane as eluent (169 mg, 96%). **^{1}H NMR** (500 MHz, DMSO-*d$_6$*): δ = 11.92 (s, 1H), 7.99 (s, 1H), 7.74 (s, 1H), 7.60 (d, *J* = 8.0 Hz, 1H), 7.52 (d, *J* = 8.5 Hz, 1H) ppm. **^{13}C**

NMR (126 MHz, DMSO-d_6): δ = 136.65, 125.32, 124.92, 124.48, 122.89, 120.13, 113.63, 104.21, 102.13 ppm. **FTIR**: ν_{max} (neat)/cm^{-1} = 3291, 2923, 2224, 1621, 1473, 1217, 798, 626. **HRMS (ESI)**: calculated for $C_9H_6ClN_2$ ([M+H]$^+$) 177.0214; found 177.0210.

Methyl (3-chloro-1*H*-indole-5-carbonyl)-*L*-alaninate (2h):

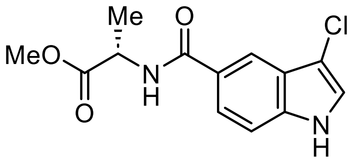

The titled compound **2h** was synthesized according to **GP I** and isolated as a white solid after column chromatography on silica gel using 35% ethyl acetate in hexane as eluent (224 mg, 80%). **^{1}H NMR** (500 MHz, DMSO-d_6): δ = 11.60 (s, 1H), 8.77 (d, *J* = 7.0 Hz, 1H), 8.16 (s, 1H), 7.75 (dd, *J* = 8.5, 1.0 Hz, 1H), 7.62 (d, *J* = 2.5 Hz, 1H), 7.47 (d, *J* = 9.0 Hz, 1H), 4.52 (p, *J* = 7.3 Hz, 1H), 3.65 (s, 3H), 1.43 (d, *J* = 7.5 Hz, 3H) ppm. **^{13}C NMR** (126 MHz, DMSO-d_6): δ = 173.39, 166.72, 136.50, 125.51, 123.98, 123.81, 122.00, 117.28, 111.77, 104.31, 51.75, 48.26, 16.79 ppm. **FTIR**: ν_{max} (neat)/cm^{-1} = 3297, 2924, 1732, 1636, 1535, 1351, 1208, 748. **HRMS (ESI)**: calculated for $C_{13}H_{14}ClN_2O_3$ ([M+H]$^+$) 281.0687; found 281.0687. **$[\alpha]_D^{25}$**: +9.08 (c 0.233, MeOH).

3-Chloro-1*H*-indole-6-carbonitrile (2j):

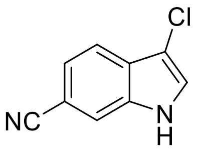

The titled compound **2j** was synthesized according to **GP I** and isolated as a white solid after column chromatography on silica gel using 18% ethyl acetate in hexane as eluent (148 mg, 84%). **^{1}H NMR** (500 MHz, DMSO-d_6): δ = 11.94 (s, 1H), 7.95 (s, 1H), 7.83 (d, *J* = 2.5 Hz, 1H), 7.64 (d, *J* = 8.5 Hz, 1H), 7.43 (dd, *J* = 8.5, 1.0 Hz, 1H) ppm. **^{13}C NMR** (126 MHz, DMSO-d_6): δ = 133.66, 127.36, 127.01, 122.33, 120.04, 118.29, 117.29, 104.01, 103.83 ppm. **FTIR**: ν_{max} (neat)/cm^{-1} = 3298, 2922, 2223, 1458, 1220, 807. **HRMS (ESI)**: calculated for $C_9H_5ClN_2Na$ ([M+Na]$^+$) 199.0033; found 199.0034.

Methyl 3-chloro-1*H*-indole-6-carboxylate (2k):

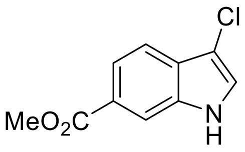

The titled compound **2k** was synthesized according to **GP I** and isolated as a white solid after column chromatography on silica gel using 15% ethyl acetate in hexane as eluent (153 mg, 73%). **^{1}H NMR** (500 MHz, $CDCl_3$): δ = 8.55 (br s, 1H), 8.14 (s, 1H), 7.88 (d, *J* = 8.5 Hz, 1H), 7.66 (d, *J* = 8.5 Hz, 1H), 7.33 (d, *J* = 2.5 Hz, 1H), 3.95 (s, 3H) ppm. **^{13}C NMR** (126 MHz, $CDCl_3$): δ = 168.04, 134.50, 128.95, 125.11, 124.35, 121.54, 118.21, 114.14, 107.16, 52.24 ppm. **FTIR**: ν_{max} (neat)/cm^{-1} = 3303, 2949, 1695, 1435, 1317, 1113, 764. **HRMS (ESI)**: calculated for $C_{10}H_9ClNO_2$ ([M+H]$^+$) 210.0316; found 210.0313.

3-Chloro-6-nitro-1*H*-indole (2l):

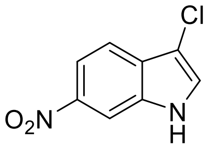

The titled compound **2l** was synthesized according to **GP I** and isolated as a yellow solid after column chromatography on silica gel using 12% ethyl acetate in hexane as eluent (159 mg, 81%). **^{1}H NMR** (400 MHz, DMSO-d_6): δ = 12.14 (s, 1H), 8.36 (d, *J* = 2.0 Hz, 1H), 7.97 (dd, *J* = 8.4, 2.4 Hz, 2H), 7.66 (d, *J* = 8.8 Hz, 1H) ppm. **^{13}C NMR** (126 MHz, DMSO-d_6): δ = 142.76, 133.20, 129.12, 128.81, 117.48, 114.67, 108.96, 104.19 ppm. **FTIR**: ν_{max} (neat)/cm^{-1} = 3336, 2919, 1510, 1339, 1208, 1065, 751. **HRMS (ESI)**: calculated for $C_8H_6ClN_2O_2$ ([M+H]$^+$) 197.0112; found 197.0101.

1-(3-Chloro-4-methyl-1*H*-indol-5-yl)ethan-1-one (2m):

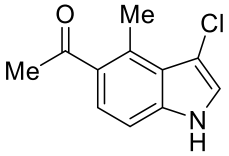

The titled compound **2m** was synthesized according to **GP I** and isolated as a white solid after column chromatography on silica gel using 12% ethyl acetate in hexane as eluent (178 mg, 86%). **^{1}H NMR** (500 MHz, DMSO-d_6): δ = 11.60 (s, 1H), 7.55-7.53 (m, 2H), 7.30 (d, *J* = 8.5 Hz, 1H), 2.84 (s, 3H), 2.55 (s, 3H) ppm. **^{13}C NMR** (126 MHz, DMSO-d_6): δ = 201.80, 136.33, 130.81, 130.47, 124.58, 123.57, 122.59, 109.71, 104.85, 30.48, 15.60 ppm. **FTIR**: ν_{max} (neat)/cm^{-1} = 3293, 2925, 1718, 1657, 1355, 1033, 794. **HRMS (ESI)**: calculated for $C_{11}H_{11}ClNO$ ([M+H]$^+$) 208.0524; found 208.0519.

3-Chloro-7-methyl-1*H*-indole (2o):

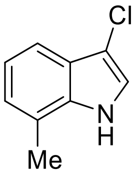

The titled compound **2o** was synthesized according to **GP I** and isolated as a white solid after column chromatography on silica gel using 3% ethyl acetate in hexane as eluent (157 mg, 95%). **^{1}H NMR** (500 MHz, DMSO-d_6): δ = 11.31 (s, 1H), 7.48 (d, *J* = 2.5 Hz, 1H), 7.31 (d, *J* = 7.5 Hz, 1H), 7.03-6.97 (m, 2H), 2.46 (s, 3H) ppm. **^{13}C NMR** (126 MHz, DMSO-d_6): δ = 134.46, 124.33, 122.65, 122.02, 121.41, 119.91, 114.66, 103.62, 16.37 ppm. **FTIR**: ν_{max} (neat)/cm^{-1} = 3391, 2924, 1723, 1328, 1052, 779. **HRMS (ESI)**: calculated for C_9H_9ClN ([M+H]$^+$) 166.0418; found 166.0419.

Preparation of the Allylboronic acids 4:

Synthesis of Allyl Alcohols: All the allyl alcohols were synthesized according to known literature procedures.[2]

General Procedure (GP II) for the preparation of Allylboronic Acids:[3]

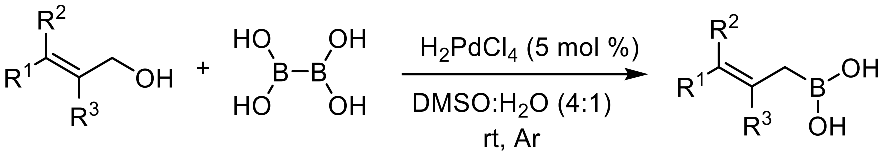

3a-3r 1.2 equiv **4a-4r**

A 10 mL two neck round bottom flask containing allyl alcohol (1.0 mmol) was evacuated under vacuo and then backfilled with argon. This process is repeated thrice. Then DMSO (1.6 mL) and H_2O (0.4 mL) were added and the resulting solution was stirred for 2 minutes. Next, freshly prepared H_2PdCl_4 (0.3 M, 0.17 mL, 0.05 mmol) and diboronic acid (108 mg, 1.2 mmol, 1.2 equiv) were added sequentially and the reaction mixture was stirred at room temperature for another 2 to 24 hours. Upon complete consumption of allyl alcohol as evidenced by TLC (*p*-anisaldehyde stain), reaction mixture was allowed to settle down over 1 to 2 hours for the complete sedimentation of Pd. Then the crude mixture was transferred using a syringe to a 25 mL round bottom flask under argon and was diluted with 2 mL degassed CH_2Cl_2 and combined solution was washed with 4 mL of degassed 16% aqueous NaCl solution. After separation of the two layers, the organic layer was taken into a 5 mL syringe and the aqueous layer was discarded. The organic layer was syringed back into the same round bottom flask was similarly washed twice more with 16% aqueous NaCl. Finally, the organic layer containing allyl-boronic acid was taken off and transferred into another 10 mL round bottom flask containing anhydrous Na_2SO_4 under argon, and the crude allylboronic acid solution was used immediately for the indole-C2-reverse-allylation reaction.

Note: [a] All the solvents were degassed for 3 hours with Ar (99.999%) under sonication. [b] In few cases, when all allyl alcohols were not consumed even after prolonged reaction time, then additional 1-2 mol% H_2PdCl_4 was added along with 0.2-0.4 equiv of $B_2(OH)_4$ to the reaction mixture. The stirring was continued further until complete consumption of allyl alcohols.

General Procedure (GP III) for the Branch-Selective Indole C2-Allylation:

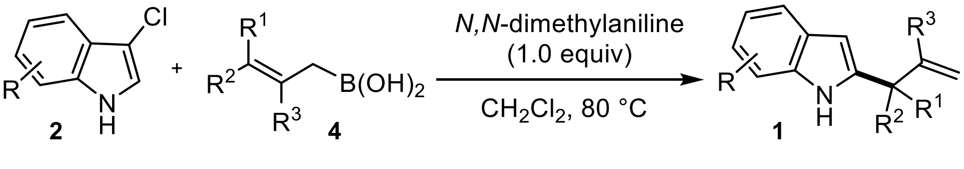

A 10 mL screw cap seal reaction tube was charged with 3-chloroindole derivatives **2** (0.2 mmol, 1.0 equiv). To it, solution of allylboronic acid **4** in 2 mL CH_2Cl_2 (prepared from 1.0 mmol of

allyl alcohol) and *N*,*N*-dimethylaniline (26 µL, 0.2 mmol, 1.0 equiv) were added at room temperature. The seal tube was flushed with argon, and the reaction mixture was stirred at 80 °C in a pre-heated oil bath for 24-36 h. Upon consumption of the starting material as indicated by TLC, reaction mixture was cooled down to room temperature, diluted with CH_2Cl_2 (15 mL) and washed with 1 (N) HCl (2 × 10 mL). The organic layer was washed with saturated aqueous $NaHCO_3$ solution once, dried over anhydrous Na_2SO_4, concentrated under reduced pressure and the crude residue was purified by silica gel column chromatography using ethyl acetate in hexane as eluent to obtain the pure desired allylated products **1**.

2-(2-Methylbut-3-en-2-yl)-1*H*-indole (1aa):

The titled compound **1aa** was synthesized according to **GP III**. Column chromatography on silica gel using 0.5% ethyl acetate in hexane as eluent provided the product **1aa** (31 mg, 83%) as a colorless oil.

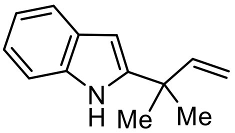

^{1}H NMR (600 MHz, $CDCl_3$): δ = 7.90 (br s, 1H), 7.57 (d, *J* = 7.8 Hz, 1H), 7.31 (d, *J* = 7.8 Hz, 1H), 7.16-7.13 (m, 1H), 7.09 (t, *J* = 7.5 Hz, 1H), 6.33 (d, *J* = 1.2 Hz, 1H), 6.06 (dd, *J* = 17.4, 10.2 Hz, 1H), 5.15-5.12 (m, 2H), 1.50 (s, 6H) ppm. **^{13}C NMR** (151 MHz, $CDCl_3$): δ = 146.21, 145.88, 136.00, 128.66, 121.41, 120.23, 119.73, 112.34, 110.57, 98.08, 38.31, 27.54 ppm. **FTIR**: ν_{max} (neat)/cm^{-1} = 3421, 2967, 2925, 1542, 1459, 1289, 998, 735. **HRMS (ESI)**: calculated for $C_{13}H_{16}N$ ($[M+H]^+$) 186.1277; found 186.1279.

2-(3-Methylhex-1-en-3-yl)-1*H*-indole (1ab):

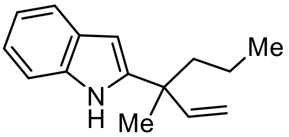

The titled compound **1ab** was synthesized according to the **GP III**. Column chromatography on silica gel using 0.5% ethyl acetate in hexane as eluent provided the product **1ab** (35 mg, 81%) as a colorless oil. **^{1}H NMR** (400 MHz, $CDCl_3$): δ = 7.89 (br s, 1H), 7.58 (d, *J* = 7.6 Hz, 1H), 7.32 (d, *J* = 8.0 Hz, 1H), 7.17-7.08 (m, 2H), 6.34 (d, *J* = 1.2 Hz, 1H), 6.05 (dd, *J* = 17.4, 10.6 Hz, 1H), 5.20-5.11 (m, 2H), 1.87-1.72 (m, 2H), 1.46 (s, 3H), 1.36-1.23 (m, 2H), 0.93 (t, *J* = 7.4 Hz, 3H) ppm. **^{13}C NMR** (126 MHz, $CDCl_3$): δ = 145.52, 145.09, 136.00, 128.72, 121.32, 120.21, 119.69, 113.07, 110.55, 99.02, 42.90, 41.84, 24.53, 17.79, 14.78 ppm. **FTIR**: ν_{max} (neat)/cm^{-1} = 3419, 2957, 1634, 1457, 1290, 916, 735. **HRMS (ESI)**: calculated for $C_{15}H_{20}N$ ($[M+H]^+$) 214.1590; found 214.1585.

2-(3,4-Dimethylpent-1-en-3-yl)-1*H*-indole (1ac):

The titled compound **1ac** was synthesized according to the **GP III**. Column chromatography on silica gel using 0.5% ethyl acetate in hexane as eluent provided the product **1ac** (30 mg, 71%)

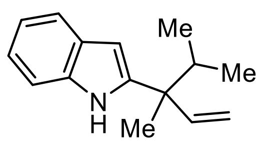

as a colorless oil. **^{1}H NMR** (400 MHz, $CDCl_3$): δ = 7.91 (br s, 1H), 7.55 (d, *J* = 7.6 Hz, 1H), 7.31 (d, *J* = 8.0 Hz, 1H), 7.14-7.05 (m, 2H), 6.31 (d, *J* = 1.6 Hz, 1H), 6.13 (dd, *J* = 17.6, 10.8 Hz, 1H), 5.22 (d, *J* = 10.8 Hz, 1H), 5.12 (d, *J* = 17.6 Hz, 1H), 2.14 (sep, *J* = 6.8 Hz, 1H), 1.40 (s, 3H), 0.93 (d, *J* = 6.8 Hz, 3H), 0.86 (d, *J* = 6.8 Hz, 3H) ppm. **^{13}C NMR** (101 MHz, $CDCl_3$): δ = 145.07, 143.30, 135.72, 128.57, 121.18, 120.12, 119.65, 114.37, 110.53, 99.53, 45.26, 36.24, 20.62, 18.11 ppm. **FTIR**: ν_{max} (neat)/cm^{-1} = 3415, 2964, 2875, 1718, 1457, 1408, 1287, 916. **HRMS (ESI)**: calculated for $C_{15}H_{20}N$ ($[M+H]^+$) 214.1590; found 214.1591.

2-(3,4,4-Trimethylpent-1-en-3-yl)-1*H*-indole (1ad):

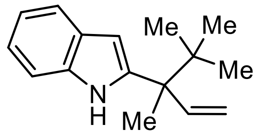

The titled compound **1ad** was synthesized according to the **GP III**. Column chromatography on silica gel using hexane as eluent provided the product **1ad** (17 mg, 38%, 63% brsm) as a colorless oil along with recovered starting material (12 mg). **^{1}H NMR** (500 MHz, $CDCl_3$): δ = 7.95 (br s, 1H), 7.57 (d, *J* = 7.5 Hz, 1H), 7.32 (d, *J* = 8.0 Hz, 1H), 7.14-7.11 (m, 1H), 7.09-7.06 (m, 1H), 6.52 (dd, *J* = 17.3, 10.8 Hz, 1H), 6.36 (d, *J* = 1.5 Hz, 1H), 5.24 (dd, *J* = 10.8, 1.3 Hz, 1H), 5.09 (dd, *J* = 17.5, 1.0 Hz, 1H), 1.50 (s, 3H), 0.98 (s, 9H) ppm. **^{13}C NMR** (126 MHz, $CDCl_3$): δ = 143.65, 142.24, 135.21, 128.18, 121.15, 120.04, 119.62, 114.98, 110.49, 101.71, 47.62, 36.90, 26.77, 20.64 ppm. **FTIR**: ν_{max} (neat)/cm^{-1} = 3436, 2924, 2854, 1457, 1373, 1290, 1009, 918. **HRMS (ESI)**: calculated for $C_{16}H_{22}N$ ($[M+H]^+$) 228.1747; found 228.1745.

2-(1-Vinylcyclopentyl)-1*H*-indole (1ae):

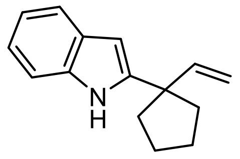

The titled compound **1ae** was synthesized according to the **GP III**. Column chromatography on silica gel using hexane as eluent provided the product **1ae** (31 mg, 73%) as a colorless oil. **^{1}H NMR** (400 MHz, $CDCl_3$): δ = 7.87 (br s, 1H), 7.55 (d, *J* = 7.6 Hz, 1H), 7.30 (d, *J* = 8.0 Hz, 1H), 7.15-7.11 (m, 1H), 7.09-7.05 (m, 1H), 6.34 (d, *J* = 2.0 Hz, 1H), 6.03 (dd, *J* = 17.4, 10.6 Hz, 1H), 5.11 (dd, *J* = 10.4, 1.2 Hz, 1H), 5.03 (dd, *J* = 17.4, 1.0 Hz, 1H), 2.10-2.05 (m, 2H), 2.01-1.95 (m, 2H), 1.78-1.75 (m, 4H) ppm. **^{13}C NMR** (101 MHz, $CDCl_3$): δ = 144.59, 144.26, 136.16, 128.62, 121.33, 120.16, 119.69, 113.01, 110.52, 98.96, 50.54, 37.65, 23.71 ppm. **FTIR**: ν_{max} (neat)/ cm^{-1} = 3415, 2956, 2872, 1634, 1455, 1406, 1287, 997. **HRMS (ESI)**: calculated for $C_{15}H_{18}N$ ($[M+H]^+$) 212.1434; found 212.1428.

2-(1-Vinylcyclohexyl)-1*H*-indole (1af):

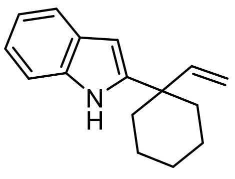

The titled compound **1af** was synthesized according to the **GP III**. Column chromatography on silica gel using hexane as eluent provided the product **1af** (41 mg, 91%) as a colorless oil. **^{1}H NMR** (500 MHz, $CDCl_3$): δ = 7.89 (br s, 1H), 7.56 (d, *J* = 7.5 Hz, 1H), 7.31 (d, *J* = 7.5 Hz, 1H), 7.14-7.11 (m, 1H), 7.09-7.06 (m, 1H), 6.36 (d, *J* = 2.0 Hz, 1H), 5.90 (dd, *J* = 17.5, 10.5 Hz, 1H), 5.13 (d, *J* = 10.5 Hz, 1H), 4.99 (d, *J* = 17.5 Hz, 1H), 2.05-2.00 (m, 2H), 1.90-1.85 (m, 2H), 1.63-1.57 (m, 4H), 1.49-1.45 (m, 2H) ppm. **^{13}C NMR** (126 MHz, $CDCl_3$): δ = 145.83, 144.43, 135.92, 128.75, 121.28, 120.13, 119.67, 113.56, 110.60, 99.47, 42.44, 35.95, 26.22, 22.53 ppm. **FTIR**: ν_{max} (neat)/ cm^{-1} = 3424, 2928, 2854, 1457, 1405, 1343, 1290, 997. **HRMS (ESI)**: calculated for $C_{16}H_{20}N$ ($[M+H]^+$) 226.1590; found 226.1586.

2-(3,7-Dimethylocta-1,6-dien-3-yl)-1*H*-indole (1ag):

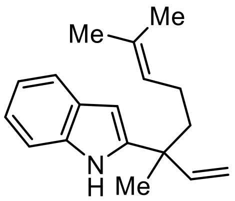

The titled compound **1ag** was synthesized according to **GP III**. Column chromatography on silica gel using 0.5% ethyl acetate in hexane as eluent provided the product **1ag** (42 mg, 82%) as a colorless oil. **^{1}H NMR** (400 MHz, $CDCl_3$): δ = 7.89 (br s, 1H), 7.56 (d, *J* = 7.6 Hz, 1H), 7.31 (dd, *J* = 8.0, 0.8 Hz, 1H), 7.15-7.11 (m, 1H), 7.08 (td, *J* = 7.4, 1.2 Hz, 1H), 6.34-6.33 (m, 1H), 6.05 (dd, *J* = 17.4, 10.6 Hz, 1H), 5.18 (dd, *J* = 10.6, 1.0 Hz, 1H), 5.15-5.09 (m, 2H), 2.01-1.95 (m, 1H), 1.91-1.79 (m, 3H), 1.67 (s, 3H), 1.55 (s, 3H), 1.47 (s, 3H) ppm. **^{13}C NMR** (101 MHz, $CDCl_3$): δ = 145.22, 144.79, 135.96, 131.86, 128.64, 124.38, 121.33, 120.20, 119.66, 113.28, 110.55, 99.06, 41.73, 40.35, 25.82, 24.39, 23.24, 17.77 ppm. **FTIR**: ν_{max} (neat)/cm^{-1} = 3418, 2924, 1619, 1457, 1375, 1290, 917. **HRMS (ESI)**: calculated for $C_{18}H_{24}N$ ($[M+H]^+$) 254.1903; found 254.1899.

2-(3,7,11-Trimethyldodeca-1,6,10-trien-3-yl)-1*H*-indole (1ah):

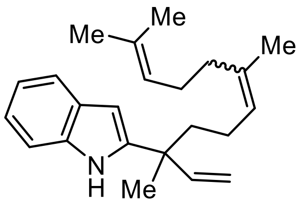

The titled compound **1ah** was synthesized according to the **GP III**. Column chromatography on silica gel using hexane as eluent provided the product **1ah** (52 mg, 81%, mixture of *E*/*Z* as obtained from the commercially available farnesol) as a colorless oil. **^{1}H NMR (major isomer)** (400 MHz, DMSO-d_6): δ = 10.78 (br s, 1H), 7.41 (d, *J* = 8.0 Hz, 1H), 7.29 (d, *J* = 8.0 Hz, 1H), 6.99 (t, *J* = 7.6 Hz, 1H), 6.91 (t, *J* = 7.4 Hz, 1H), 6.16-6.08 (m, 2H), 5.08-4.99 (m, 4H), 2.02-1.97 (m, 2H), 1.91-1.83 (m, 4H), 1.77-1.70 (m, 2H), 1.62 (s, 3H), 1.54 (s, 3H), 1.47 (s, 3H), 1.42 (d, *J* = 2.8 Hz, 3H) ppm. **^{13}C NMR (major isomer)** (101 MHz, DMSO-d_6):

δ= 145.22, 136.37, 134.33, 130.64, 127.67, 125.05, 124.15, 124.10, 120.20, 119.31, 118.47, 112.12, 110.77, 97.60, 41.18, 31.45, 26.15, 25.47, 23.07, 22.76, 17.53, 17.25, 15.68 ppm. **FTIR**: ν_{max} (neat)/ cm^{-1} = 3452, 2923, 2855, 1457, 1375, 1288, 1001, 785. **HRMS (ESI)**: calculated for $C_{23}H_{32}N$ ($[M+H]^+$) 322.2529; found 322.2525.

2-(1-(Benzyloxy)-2-methylbut-3-en-2-yl)-1*H*-indole (1ai):

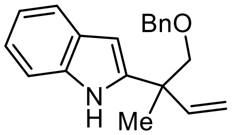

The titled compound **1ai** was synthesized according to the **GP III**. Column chromatography on silica gel using 1% ethyl acetate in hexane as eluent provided the product **1ai** (54 mg, 92%) as a colorless oil. **^{1}H NMR** (400 MHz, $CDCl_3$): δ = 8.91 (br s, 1H), 7.58 (d, *J* = 7.6 Hz, 1H), 7.42-7.34 (m, 5H), 7.31 (d, *J* = 8.0 Hz, 1H), 7.15 (t, *J* = 7.4 Hz, 1H), 7.08 (t, *J* = 7.4 Hz, 1H), 6.33-6.32 (m, 1H), 6.13 (dd, *J* = 17.4, 10.6 Hz, 1H), 5.19 (d, *J* = 10.4 Hz, 1H), 5.04 (d, *J* = 17.6 Hz, 1H), 4.62 (s, 2H), 3.71 (d, *J* = 8.8 Hz, 1H), 3.63 (d, *J* = 8.8 Hz, 1H), 1.49 (s, 3H) ppm. **^{13}C NMR** (101 MHz, $CDCl_3$): δ = 143.64, 142.85, 138.02, 135.94, 128.69, 128.02, 127.96, 127.89, 121.34, 120.21, 119.47, 114.60, 110.76, 99.03, 78.39, 73.88, 42.93, 22.91 ppm. **FTIR**: ν_{max} (neat)/cm^{-1} = 3425, 2854, 1456, 1291, 1219, 1075, 919,775. **HRMS (ESI)**: calculated for $C_{20}H_{22}NO$ ($[M+H]^+$) 292.1696; found 292.1704.

2-(1-((4-Methoxybenzyl)oxy)-4-vinyloctan-4-yl)-1*H*-indole (1aj):

The titled compound **1aj** was synthesized according to the **GP III**. Column chromatography on

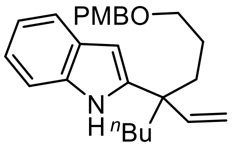

silica gel using 3% ethyl acetate in hexane as eluent provided the product **1aj** (42 mg, 53%) as a colorless oil. **^{1}H NMR** (400 MHz, $CDCl_3$): δ = 8.05 (br s, 1H), 7.58 (d, *J* = 7.6 Hz, 1H), 7.30-7.26 (m, 3H), 7.15 (t, *J* = 7.4 Hz, 1H), 7.10 (t, *J* = 7.2 Hz, 1H), 6.92 (d, *J* = 8.4 Hz, 2H), 6.36 (s, 1H), 6.00 (dd, *J* = 17.6, 10.8 Hz, 1H), 5.27 (d, *J* = 10.8 Hz, 1H), 5.19 (d, *J* = 17.6 Hz, 1H), 4.45 (s, 2H), 3.85 (s, 3H), 3.46 (t, *J* = 6.4 Hz, 2H), 1.94-1.89 (m, 2H), 1.86-1.75 (m, 2H), 1.61-1.52 (m, 2H), 1.35-1.21 (m, 4H), 0.91 (t, *J* = 7.2 Hz, 3H) ppm. **^{13}C NMR** (101 MHz, $CDCl_3$): δ = 159.28, 144.44, 143.70, 135.86, 130.72, 129.42, 128.59, 121.20, 120.11, 119.55, 114.19, 113.91, 110.61, 100.07, 72.69, 70.53, 55.43, 44.89, 37.07, 33.00, 26.17, 24.55, 23.43, 14.19 ppm. **FTIR**: ν_{max} (neat)/ cm^{-1} = 2932, 1612, 1512, 1246, 1033, 919, 736. **HRMS (ESI)**: calculated for $C_{26}H_{34}NO_2$ ($[M+H]^+$) 392.2584; found 392.2585.

2-(6-Methoxy-3-methylhex-1-en-3-yl)-1*H*-indole (1ak):

The titled compound **1ak** was synthesized according to the **GP III**. Column chromatography on silica gel using 2% ethyl acetate in hexane as eluent provided the product **1ak** (35 mg, 71%)

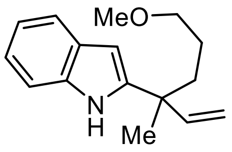

as a colorless oil. **^{1}H NMR** (500 MHz, $CDCl_3$): δ = 7.98 (br s, 1H), 7.54 (d, *J* = 7.5 Hz, 1H), 7.29 (d, *J* = 8.0 Hz, 1H), 7.12 (t, *J* = 7.5 Hz, 1H), 7.06 (t, *J* = 7.5 Hz, 1H), 6.32 (s, 1H), 6.03 (dd, *J* = 17.5, 10.5 Hz, 1H), 5.16 (d, *J* = 11.0 Hz, 1H), 5.12 (d, *J* = 17.5 Hz, 1H), 3.35 (t, *J* = 6.5 Hz, 2H), 3.31 (s, 3H), 1.94-1.81 (m, 2H), 1.62-1.58 (m, 1H), 1.52-1.48 (m, 1H), 1.45 (s, 3H) ppm. **^{13}C NMR** (126 MHz, $CDCl_3$): δ = 145.25, 144.67, 136.05, 128.69, 121.37, 120.22, 119.70, 113.31, 110.61, 99.28, 73.15, 58.70, 41.57, 36.71, 24.88, 24.64 ppm. **FTIR**: ν_{max} (neat)/ cm^{-1} = 3419, 3318, 2929, 1465, 1410, 1289, 1115, 919. **HRMS (ESI)**: calculated for $C_{16}H_{22}NO$ ($[M+H]^+$) 244.1696; found 244.1692.

4-(1*H*-indol-2-yl)-4-methylhex-5-en-1-yl 4-methylbenzenesulfonate (1al):

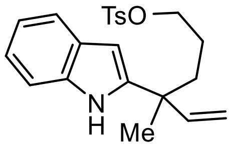

The titled compound **1al** was synthesized according to the **GP III**. Column chromatography on silica gel using 8% ethyl acetate in hexane as eluent provided the product **1al** (50 mg, 65%) as a colorless oil. **^{1}H NMR** (400 MHz, $CDCl_3$): δ = 7.88 (br s, 1H), 7.74 (d, *J* = 8.4 Hz, 2H), 7.53 (d, *J* = 7.6 Hz, 1H), 7.31-7.28 (m, 3H), 7.15-7.11 (m, 1H), 7.09-7.05 (m, 1H), 6.24 (s, 1H), 5.96 (dd, *J* = 17.4, 10.6 Hz, 1H), 5.16 (d, *J* = 10.4 Hz, 1H), 5.09 (d, *J* = 17.6 Hz, 1H), 4.01-3.97 (m, 2H), 2.43 (s, 3H), 1.86-1.74 (m, 2H), 1.69-1.61 (m, 1H), 1.54-1.48 (m, 1H), 1.40 (s, 3H) ppm. **^{13}C NMR** (101 MHz, $CDCl_3$): δ = 144.89, 144.59, 143.80, 136.05, 133.21, 129.97, 128.49, 128.00, 121.60, 120.24, 119.82, 113.77, 110.70, 99.35, 71.04, 41.28, 35.94, 24.44, 24.34, 21.76 ppm. **FTIR**: ν_{max} (neat)/ cm^{-1} = 3409, 2927, 1458, 1355, 1180, 1097, 917, 662. **HRMS (ESI)**: calculated for $C_{22}H_{26}NO_3S$ ($[M+H]^+$) 384.1628; found 384.1630.

(*E*)-2-(3,7-Dimethyl-8-(phenylthio)octa-1,6-dien-3-yl)-1*H*-indole (1am):

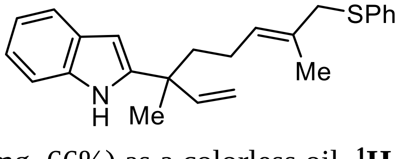

The titled compound **1am** was synthesized according to the **GP III**. Column chromatography on silica gel using 0.2% ethyl acetate in hexane as eluent provide the product **1am** (48 mg, 66%) as a colorless oil. **^{1}H NMR** (400 MHz, $CDCl_3$): δ = 7.82 (br s, 1H), 7.55 (d, *J* = 8.0 Hz, 1H), 7.34-7.24 (m, 5H), 7.21-7.19 (m, 1H), 7.15-7.11 (m, 1H), 7.09-7.05 (m, 1H), 6.28 (d, *J* = 1.6 Hz, 1H), 5.99 (dd, *J* = 17.4, 10.6 Hz, 1H), 5.20-5.15 (m, 2H), 5.11-5.07 (m, 1H), 3.45 (s, 2H), 1.99-1.88 (m, 1H), 1.87-1.81 (m, 1H), 1.74-1.69 (m, 1H), 1.66 (s, 3H), 1.64-1.59 (m, 1H), 1.42 (s, 3H) ppm. **^{13}C NMR** (101 MHz, $CDCl_3$): δ = 145.04, 144.52, 136.49, 135.97, 130.92, 130.63, 128.86, 128.78, 128.62, 126.45, 121.43, 120.22, 119.74, 113.40, 110.56, 99.14, 44.41, 41.66, 39.80, 24.38, 23.33, 15.25 ppm. **FTIR**: ν_{max} (neat)/ cm^{-1} = 3424, 2924, 1456,

1407, 1288, 1088, 919, 690. **HRMS (ESI)**: calculated for $C_{24}H_{28}NS$ ([M+H]$^+$) 362.1937; found 362.1938.

Methyl 4-(1*H*-indol-2-yl)-4-methylhex-5-enoate (1an):

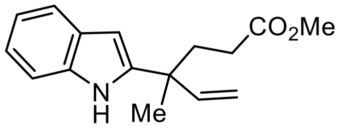

The titled compound **1an** was synthesized according to the **GP III**. Column chromatography on silica gel using 2% ethyl acetate in hexane as eluent provided the product **1an** (44 mg, 86%) as a colorless oil. **^{1}H NMR** (400 MHz, $CDCl_3$): δ = 7.94 (br s, 1H), 7.55 (d, *J* = 7.6 Hz, 1H), 7.31 (d, *J* = 8.0 Hz, 1H), 7.14 (t, *J* = 7.4 Hz, 1H), 7.07 (t, *J* = 7.4 Hz, 1H), 6.33 (s, 1H), 6.01 (dd, *J* = 17.6, 10.8 Hz, 1H), 5.22-5.14 (m, 2H), 3.61 (s, 3H), 2.35-2.29 (m, 1H), 2.26-2.18 (m, 2H), 2.16-2.11 (m, 1H), 1.45 (s, 3H) ppm. **^{13}C NMR** (101 MHz, $CDCl_3$): δ = 174.32, 144.37, 143.41, 136.10, 128.48, 121.63, 120.29, 119.81, 113.97, 110.68, 99.61, 51.78, 41.26, 34.71, 29.71, 24.41 ppm. **FTIR**: ν_{max} (neat)/ cm^{-1} = 3391, 2925, 1719, 1457, 1291, 1170, 997, 775. **HRMS (ESI)**: calculated for $C_{16}H_{20}NO_2$ ([M+H]$^+$) 258.1489; found 258.1489.

Ethyl 2-((1*H*-indol-2-yl)methyl)acrylate (1ao):

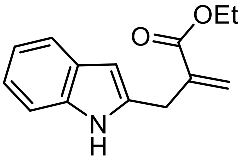

The titled compound **1ao** was synthesized according to the **GP III** in presence of diphenyl phosphate (2.5 mg, 0.01 mmol, 0.05 equiv). Column chromatography on silica gel using 1% ethyl acetate in hexane as eluent provided the product **1ao** (39 mg, 85%) as a colorless oil. **^{1}H NMR** (500 MHz, $CDCl_3$): δ = 8.44 (br s, 1H), 7.53 (d, *J* = 7.5 Hz, 1H), 7.31 (d, *J* = 8.0 Hz, 1H), 7.13 (t, *J* = 7.5 Hz, 1H), 7.07 (t, *J* = 7.3 Hz, 1H), 6.29 (s, 1H), 6.26 (s, 1H), 5.72 (d, *J* = 1.0 Hz, 1H), 4.26 (q, J = 7.2 Hz, 2H), 3.76 (s, 2H), 1.32 (t, *J* = 7.3 Hz, 3H) ppm. **^{13}C NMR** (126 MHz, $CDCl_3$): δ = 167.67, 138.52, 136.57, 136.27, 128.75, 127.01, 121.46, 120.12, 119.75, 110.74, 100.74, 61.33, 31.69, 14.29 ppm. **FTIR**: ν_{max} (neat)/ cm^{-1} = 3397, 2981, 1699, 1631, 1456, 1295, 1128, 1023, 732. **HRMS (ESI)**: calculated for $C_{14}H_{16}NO_2$ ([M+H]$^+$) 230.1176; found 230.1173.

Ethyl 2-((1*H*-indol-2-yl)(*p*-tolyl)methyl)acrylate (1ap):

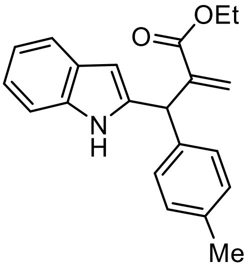

The titled compound **1ap** was synthesized according to the **GP III** in presence of diphenylphosphate (7.5 mg, 0.03 mmol, 0.15 equiv) at 100 °C. Column chromatography on silica gel using 3% ethyl acetate in hexane as eluent provided the product **1ap** (27 mg, 42%) as a colorless oil. **^{1}H NMR** (400 MHz, $CDCl_3$): δ = 8.08 (br s, 1H), 7.53 (d, *J* = 7.6 Hz, 1H), 7.27 (d, *J* = 7.2 Hz, 1H), 7.15-7.09 (m, 5H), 7.09-7.05 (m, 1H), 6.44 (s, 1H), 6.22 (d, *J* = 1.6 Hz, 1H), 5.50 (s, 1H), 5.47 (s, 1H), 4.22-4.13 (m, 2H), 2.34 (s, 3H),

1.23 (t, *J* = 7.0 Hz, 3H) ppm. **^{13}C NMR** (101 MHz, $CDCl_3$): δ = 166.89, 142.50, 139.14, 137.12, 136.90, 136.41, 129.48, 128.53, 127.49, 121.68, 120.37, 119.82, 110.80, 102.32, 61.21, 46.53, 21.19, 14.22 ppm. **FTIR**: ν_{max} (neat)/ cm^{-1} = 3415, 2985, 1702, 1513, 1457, 1249, 1169, 1022. **HRMS (ESI)**: calculated for $C_{21}H_{22}NO_2$ ($[M+H]^+$) 320.1645; found 320.1662.

2-((1*H*-indol-2-yl)(*p*-tolyl)methyl)acrylonitrile (1aq):

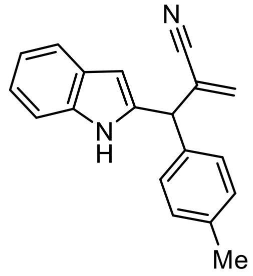

The titled compound **1aq** was synthesized according to the **GP III** in presence of diphenylphosphate (7.5 mg, 0.03 mmol, 0.15 equiv) at 100 °C. Column chromatography using 10% ethyl acetate in hexane as eluent provided the product **1aq** (31 mg, 57%) as a colorless oil. **^{1}H NMR** (500 MHz, $CDCl_3$): δ = 7.83 (br s, 1H), 7.58 (d, *J* = 8.0 Hz, 1H), 7.27 (d, *J* = 8.0 Hz, 1H), 7.20 (d, *J* = 8.0 Hz, 2H), 7.18-7.15 (m, 3H), 7.11 (td, *J* = 7.4, 0.8 Hz, 1H), 6.42 (d, *J* = 1.0 Hz, 1H), 6.13 (s, 1H), 5.77 (d, *J* = 1.0 Hz, 1H), 5.08 (s, 1H), 2.37 (s, 3H) ppm. **^{13}C NMR** (126 MHz, $CDCl_3$): δ = 138.31, 136.59, 135.95, 134.41, 132.46, 130.01, 128.61, 128.36, 124.87, 122.40, 120.73, 120.26, 118.35, 110.94, 103.16, 49.60, 21.23 ppm. **FTIR**: ν_{max} (neat)/ cm^{-1} = 3450, 2928, 2201, 1546, 1298, 1135, 1010. **HRMS (ESI)**: calculated for $C_{19}H_{17}N_2$ ($[M+H]^+$) 273.1386; found 273.1395.

2-(1-Methylcyclohex-2-en-1-yl)-1*H*-indole (1ar):

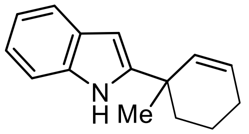

The titled compound **1ar** was synthesized according to the **GP III**. Column chromatography on silica gel using 1% ethyl acetate in hexane as eluent provided the product **1ar** (28 mg, 66%) as a colorless oil. **^{1}H NMR** (500 MHz, $CDCl_3$): δ = 7.97 (br s, 1H), 7.54 (d, *J* = 8.0 Hz, 1H), 7.31 (d, *J* = 8.0 Hz, 1H), 7.12 (td, *J* = 7.5, 1.0 Hz, 1H), 7.08-7.05 (m, 1H), 6.29 (d, *J* = 1.5 Hz, 1H), 5.95 (dt, *J* = 10.0, 3.8 Hz, 1H), 5.73 (d, *J* = 10.0 Hz, 1H), 2.11-2.06 (m, 2H), 1.99-1.94 (m, 1H), 1.76-1.72 (m, 1H), 1.68-1.64 (m, 1H), 1.62-1.58 (m, 1H), 1.47 (s, 3H) ppm. **^{13}C NMR** (126 MHz, $CDCl_3$): δ = 147.23, 135.59, 133.19, 128.96, 128.67, 121.14, 120.13, 119.70, 110.58, 98.12, 38.27, 36.51, 28.40, 25.09, 19.57 ppm. **FTIR**: ν_{max} (neat)/ cm^{-1} = 3421, 3016, 2929, 1458, 1290, 735. **HRMS (ESI)**: calculated for $C_{15}H_{18}N$ ($[M+H]^+$) 212.1434; found 212.1434.

4-Bromo-2-(2-methylbut-3-en-2-yl)-1*H*-indole (1ba):

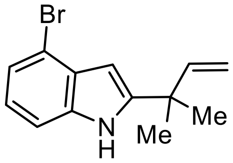

The titled compound **1ba** was synthesized according to the **GP III**. Column chromatography on silica gel using 1% ethyl acetate in hexane as eluent provided the product **1ba** (36 mg, 69%) as a colorless oil. **^{1}H NMR** (500 MHz, $CDCl_3$): δ = 8.02 (br s, 1H), 7.24 (d, *J* = 8.0 Hz, 2H), 6.98 (t, *J*

= 7.8 Hz, 1H), 6.37 (d, *J* = 2.0 Hz, 1H), 6.04 (dd, *J* = 17.5, 10.0 Hz, 1H), 5.15-5.12 (m, 2H), 1.50 (s, 6H) ppm. **^{13}C NMR** (126 MHz, $CDCl_3$): δ = 146.72, 145.83, 136.21, 129.37, 122.65, 122.36, 114.19, 112.76, 109.75, 98.56, 38.43, 27.51 ppm. **FTIR**: ν_{max} (neat)/ cm^{-1} = 3467, 2968, 1572, 1431, 1323, 1179, 913, 755. **HRMS (ESI)**: calculated for $C_{13}H_{15}BrN$ ($[M+H]^+$) 264.0382; found 264.0379.

2-(2-Methylbut-3-en-2-yl)-1*H*-indole-4-carbonitrile (1ca):

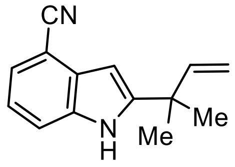

The titled compound **1ca** was synthesized according to the **GP III**. Column chromatography on silica gel using 1% ethyl acetate in hexane as eluent provided the product **1ca** (32 mg, 77%) as a colorless oil. **^{1}H NMR** (500 MHz, $CDCl_3$): δ = 8.20 (br s, 1H), 7.51 (d, *J* = 8.0 Hz, 1H), 7.42 (d, *J* = 7.5 Hz, 1H), 7.15 (t, *J* = 7.8 Hz, 1H), 6.55 (d, *J* = 2.0 Hz, 1H), 6.04 (dd, *J* = 17.5, 10.5 Hz, 1H), 5.18-5.13 (m, 2H), 1.51 (s, 6H). **^{13}C NMR** (126 MHz, $CDCl_3$): δ = 149.24, 145.41, 135.72, 130.38, 125.06, 121.06, 119.07, 115.22, 113.17, 102.47, 97.57, 38.54, 27.51 ppm. **FTIR**: ν_{max} (neat)/ cm^{-1} = 3317, 2972, 2220, 1535, 1436, 1359, 1278, 918. **HRMS (ESI)**: calculated for $C_{14}H_{15}N_2$ ($[M+H]^+$) 211.1230; found 211.1227.

5-Methoxy-2-(2-methylbut-3-en-2-yl)-1*H*-indole (1da):

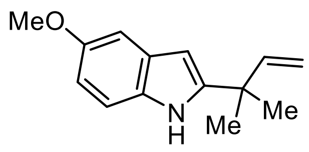

The titled compound **1da** was synthesized according to the **GP III**. Column chromatography on silica gel using 1% ethyl acetate in hexane as eluent provided the product **1da** (34 mg, 79%) as a colorless oil. **^{1}H NMR** (600 MHz, $CDCl_3$): δ = 7.77 (br s, 1H), 7.18 (d, *J* = 9.0 Hz, 1H), 7.03 (d, *J* = 1.8 Hz, 1H), 6.78 (dd, *J* = 9.0, 2.4 Hz, 1H), 6.25 (d, *J* = 1.8 Hz, 1H), 6.04 (dd, *J* = 17.1, 10.5 Hz, 1H), 5.13-5.10 (m, 2H), 3.84 (s, 3H), 1.47 (s, 6H) ppm. **^{13}C NMR** (151 MHz, $CDCl_3$): δ= 154.27, 146.76, 146.21, 131.06, 129.10, 112.31, 111.38, 111.24, 102.39, 97.96, 56.11, 38.37, 27.55 ppm. **FTIR**: ν_{max} (neat)/ cm^{-1} = 3417, 2968, 1624, 1485, 1208, 1150, 1030, 730. **HRMS (ESI)**: calculated for $C_{14}H_{18}NO$ ($[M+H]^+$) 216.1383; found 216.1380.

2-(2-Methylbut-3-en-2-yl)-5-nitro-1*H*-indole (1ea):

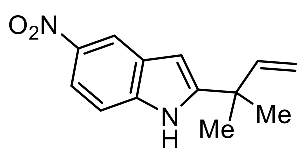

The titled compound **1ea** was synthesized according to the **GP III** and reaction was conducted at 100 °C. Column Chromatography on silica gel using 3% ethyl acetate in hexane as eluent provided the product **1ea** (40 mg, 86%) as a yellow oil. **^{1}H NMR** (600 MHz, $CDCl_3$): δ = 8.50 (d, *J* = 1.8 Hz, 1H), 8.29 (br s, 1H), 8.05 (dd, *J* = 8.7, 2.1 Hz, 1H), 7.32 (d, *J* = 9.0 Hz, 1H), 6.47 (d, *J* = 1.8 Hz, 1H), 6.03 (dd, *J* = 17.4, 10.2 Hz, 1H), 5.18-5.14 (m, 2H), 1.51 (s, 6H) ppm. **^{13}C NMR**

(151 MHz, $CDCl_3$): δ = 149.42, 145.30, 141.91, 139.16, 128.07, 117.39, 117.34, 113.24, 110.46, 100.35, 38.49, 27.36 ppm. **FTIR**: ν_{max} (neat)/ cm^{-1} = 3369, 2969, 1618, 1472, 1317, 1071, 919. **HRMS (ESI)**: calculated for $C_{13}H_{15}N_2O_2$ ($[M+H]^+$) 231.1128; found 231.1128.

2-(2-Methylbut-3-en-2-yl)-1*H*-indole-5-carbonitrile (1fa):

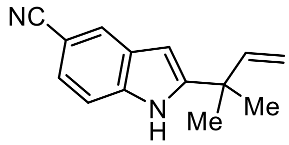

The titled compound **1fa** was synthesized according to the **GP III**. Column chromatography on silica gel using 3% ethyl acetate in hexane as eluent provided the product **1fa** (32 mg, 77%) as a colorless oil. **^{1}H NMR** (600 MHz, $CDCl_3$): δ = 8.20 (br s, 1H), 7.88 (s, 1H), 7.37-7.33 (m, 2H), 6.38 (d, *J* = 1.8 Hz, 1H), 6.03 (dd, *J* = 17.4, 10.8 Hz, 1H), 5.17-5.12 (m, 2H), 1.50 (s, 6H) ppm. **^{13}C NMR** (151 MHz, $CDCl_3$): δ = 148.45, 145.47, 137.79, 128.60, 125.70, 124.61, 121.06, 113.11, 111.41, 102.83, 98.92, 38.41, 27.44 ppm. **FTIR**: ν_{max} (neat)/ cm^{-1} = 3331, 2978, 2219, 1613, 1473, 1318, 916, 784. **HRMS (ESI)**: calculated for $C_{14}H_{15}N_2$ ($[M+H]^+$) 211.1230; found 211.1239.

5-Bromo-2-(2-methylbut-3-en-2-yl)-1*H*-indole (1ga):

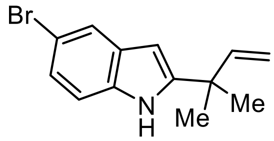

The titled compound **1ga** was synthesized according to the **GP III**. Column chromatography on silica gel using 1% ethyl acetate in hexane as eluent provided the product **1ga** (40 mg, 75%) as a colorless oil. **^{1}H NMR** (500 MHz, $CDCl_3$): δ = 7.91 (br s, 1H), 7.66 (s, 1H), 7.20 (dd, *J* = 8.5, 2.0 Hz, 1H), 7.16 (d, *J* = 8.5 Hz, 1H), 6.26 (d, *J* = 2.0 Hz, 1H), 6.03 (dd, *J* = 17.3, 10.8 Hz, 1H), 5.14-5.10 (m, 2H), 1.48 (s, 6H) ppm. **^{13}C NMR** (126 MHz, $CDCl_3$): δ = 147.32, 145.88, 134.66, 130.53, 124.21, 122.75, 112.91, 112.67, 111.97, 97.86, 38.37, 27.52 ppm. **FTIR**: ν_{max} (neat)/ cm^{-1} = 3432, 3055, 1599, 1455, 1341, 1135, 736. **HRMS (ESI)**: calculated for $C_{13}H_{15}BrN$ ($[M+H]^+$) 264.0382; found 264.0382.

Methyl (2-(2-methylbut-3-en-2-yl)-1*H*-indole-5-carbonyl)-*L*-alaninate (1ha):

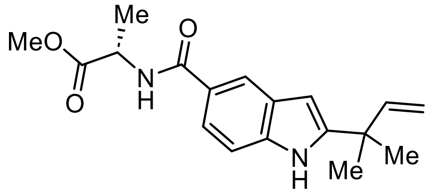

The titled compound **1ha** was synthesized according to the **GP III**. Column chromatography on silica gel using 30% ethyl acetate in hexane as eluent provided the product **1ha** (38 mg, 60%) as a colorless oil. **^{1}H NMR** (500 MHz, $CDCl_3$): δ = 8.26 (br s, 1H), 8.04 (d, *J* = 1.5 Hz, 1H), 7.60 (dd, *J* = 8.5, 1.5 Hz, 1H), 7.29 (d, *J* = 8.5 Hz, 1H), 6.74 (d, *J* = 6.5 Hz, 1H), 6.36 (d, *J* = 1.0 Hz, 1H), 6.04 (dd, *J* = 17.3, 10.8 Hz, 1H), 5.13-5.09 (m, 2H), 4.85 (quint, *J* = 7.3 Hz, 1H), 3.78 (s, 3H), 1.53 (d, *J* = 7.0 Hz, 3H), 1.49 (s, 6H) ppm. **^{13}C NMR** (126 MHz, $CDCl_3$): δ = 174.15, 168.15, 147.59, 145.92, 138.11, 128.43,

125.71, 120.63, 119.93, 112.63, 110.52, 99.13, 52.56, 48.65, 38.41, 27.50, 18.96 ppm. **FTIR**: ν_{max} (neat)/ cm^{-1} = 3289, 2968, 1736, 1635, 1524, 1317, 1217, 790. **HRMS (ESI)**: calculated for $C_{18}H_{23}N_2O_3$ ($[M+H]^+$) 315.1703; found 315.1705. **$[\alpha]_D^{25}$**: +2.12 (c 0.233, MeOH).

HPLC conditions: ID column, n-hexane/2-propanol = 80/20, flow rate = 0.5 mL/min, one enantiomer: t_R = 25.81 min; another enantiomer: t_R = 37.67 min (*ee* 100%). The HPLC analysis confirmed that the stereointegrity of the chiral center in **1ha** has been retained during the reaction as no racemization was observed.

6-Bromo-2-(2-methylbut-3-en-2-yl)-1*H*-indole (1ia):

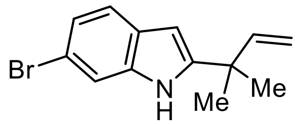

The titled compound **1ia** was synthesized according to the **GP III**. Column chromatography on silica gel using 3% ethyl acetate in hexane as eluent provided the product **1ia** (34 mg, 65%) as a colorless oil. **^{1}H NMR** (500 MHz, $CDCl_3$): δ = 7.87 (br s, 1H), 7.45 (s, 1H), 7.40 (d, *J* = 8.5 Hz, 1H), 7.17 (dd, *J* = 8.0, 1.0 Hz, 1H), 6.29 (d, *J* = 1.0 Hz, 1H), 6.03 (dd, *J* = 17.3, 10.8 Hz, 1H), 5.14-5.10 (m, 2H), 1.48 (s, 6H) ppm. **^{13}C NMR** (126 MHz, $CDCl_3$): δ = 146.68, 145.91, 136.88, 127.63, 123.03, 121.44, 114.74, 113.56, 112.65, 98.34, 38.36, 27.51 ppm. **FTIR**: ν_{max} (neat)/ cm^{-1} = 3431, 2968, 1611, 1453, 1379, 1218, 905, 808. **HRMS (ESI)**: calculated for $C_{13}H_{15}BrN$ ($[M+H]^+$) 264.0382; found 264.0377.

2-(2-Methylbut-3-en-2-yl)-1*H*-indole-6-carbonitrile (1ja):

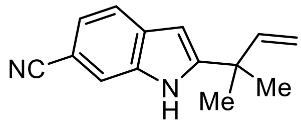

The titled compound **1ja** was synthesized according to the **GP III**. Column chromatography on silica gel using 5% ethyl acetate in hexane as eluent provided the product **1ja** (22 mg, 52%) as a colorless oil. **^{1}H NMR** (400 MHz, $CDCl_3$): δ = 8.21 (br s, 1H), 7.63 (s, 1H), 7.58 (d, *J* = 8.4 Hz, 1H), 7.31 (d, *J* = 8.0 Hz, 1H), 6.40 (d, *J* = 0.8 Hz, 1H), 6.03 (dd, *J* = 17.4, 10.6 Hz, 1H), 5.19-5.13 (m, 2H), 1.50 (s, 6H) ppm. **^{13}C NMR** (101 MHz, $CDCl_3$): δ = 150.33, 145.26, 134.75, 132.08, 122.93, 121.09, 120.90, 115.33, 113.21, 103.49, 99.12, 38.50, 27.42 ppm. **FTIR**: ν_{max} (neat)/ cm^{-1} = 3330, 2970, 2218, 1465, 1307, 1231, 817. **HRMS (ESI)**: calculated for $C_{14}H_{15}N_2$ ($[M+H]^+$) 211.1230; found 211.1221.

Methyl 2-(2-methylbut-3-en-2-yl)-1*H*-indole-6-carboxylate (1ka):

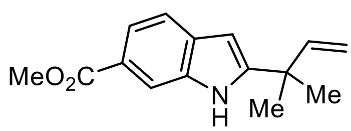

The titled compound **1ka** was synthesized according to the **GP III**. Column chromatography on silica gel using 6% ethyl acetate in hexane as eluent provided the product **1ka** (26 mg, 53%) as a colorless oil. **^{1}H NMR** (500 MHz, $CDCl_3$): δ = 8.24 (br s, 1H), 8.09 (s, 1H), 7.78 (dd, *J* = 8.3,

1.3 Hz, 1H), 7.55 (d, *J* = 8.5 Hz, 1H), 6.37 (d, *J* = 1.5 Hz, 1H), 6.06 (dd, *J* = 17.3, 10.8 Hz, 1H), 5.15-5.12 (m, 2H), 3.93 (s, 3H), 1.51 (s, 6H) ppm. **^{13}C NMR** (126 MHz, $CDCl_3$): δ = 168.43, 149.73, 145.70, 135.39, 132.59, 123.08, 121.03, 119.69, 113.03, 112.81, 98.76, 51.96, 38.53, 27.52 ppm. **FTIR**: ν_{max} (neat)/ cm^{-1} = 3356, 2969, 1694, 1532, 1318, 1215, 1091. **HRMS (ESI)**: calculated for $C_{15}H_{18}NO_2$ ($[M+H]^+$) 244.1332; found 244.1328.

2-(2-Methylbut-3-en-2-yl)-6-nitro-1*H*-indole (1la):

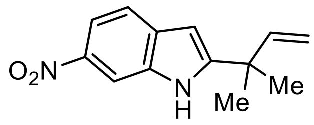

The titled compound **1la** was synthesized according to the **GP III**. Column chromatography on silica gel using 5% ethyl acetate in hexane as eluent provided the product **1la** (16 mg, 35%) as a yellow oil along with recovered starting material (17 mg). **^{1}H NMR** (500 MHz, $CDCl_3$): δ = 8.39 (br s, 1H), 8.28 (s, 1H), 7.99 (dd, *J* = 8.8, 1.8 Hz, 1H), 7.56 (d, *J* = 9.0 Hz, 1H), 6.43 (s, 1H), 6.05 (dd, *J* = 17.5, 10.5 Hz, 1H), 5.20-5.15 (m, 2H), 1.52 (s, 6H) ppm. **^{13}C NMR** (126 MHz, $CDCl_3$): δ = 152.57, 145.09, 142.82, 134.44, 133.87, 119.90, 115.62, 113.40, 107.60, 99.43, 38.71, 27.43 ppm. **FTIR**: ν_{max} (neat)/ cm^{-1} = 3376, 2970, 1504, 1335, 1292, 1070, 823. **HRMS (ESI)**: calculated for $C_{13}H_{15}N_2O_2$ ($[M+H]^+$) 231.1128; found 231.1125.

1-(4-Methyl-2-(2-methylbut-3-en-2-yl)-1*H*-indol-5-yl)ethan-1-one (1ma):

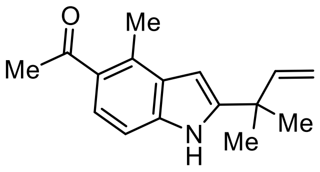

The titled compound **1ma** was synthesized according to the **GP III**. Column chromatography on silica gel using 6% ethyl acetate in hexane as eluent provided the product **1ma** (31 mg, 64%) as a colorless oil. **^{1}H NMR** (500 MHz, $CDCl_3$): δ = 8.15 (br s, 1H), 7.57 (d, *J* = 8.5 Hz, 1H), 7.16 (d, *J* = 8.5 Hz, 1H), 6.47 (d, *J* = 1.5 Hz, 1H), 6.05 (dd, *J* = 17.0, 11.0 Hz, 1H), 5.14-5.11 (m, 2H), 2.78 (s, 3H), 2.63 (s, 3H), 1.50 (s, 6H). **^{13}C NMR** (126 MHz, $CDCl_3$): δ = 201.77, 146.66, 145.93, 137.21, 132.64, 129.91, 129.25, 124.04, 112.67, 107.65, 98.43, 38.42, 30.10, 27.52, 17.58 ppm. **FTIR**: ν_{max} (neat)/ cm^{-1} = 3324, 2969, 1652, 1599, 1354, 1282, 1135, 789. **HRMS (ESI)**: calculated for $C_{16}H_{20}NO$ ($[M+H]^+$) 242.1539; found 242.1530.

2-(2-Methylbut-3-en-2-yl)-1*H*-indol-4-ol (1na):

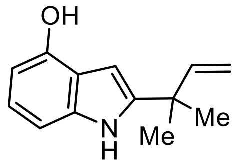

The titled compound **1na** was synthesized according to the **GP III**. Column chromatography on silica gel using 10% ethyl acetate in hexane as eluent provided the product **1na** (25 mg, 62%) as a colorless oil. **^{1}H NMR** (400 MHz, $CDCl_3$): δ = 7.94 (br s, 1H), 6.99 (t, *J* = 7.8 Hz, 1H), 6.93 (d, *J* = 8.0 Hz, 1H), 6.50 (d, *J* = 7.2 Hz, 1H), 6.37 (d, *J* = 2.0 Hz, 1H), 6.04 (dd, *J* = 17.6, 10.4 Hz, 1H), 5.15-5.11 (m, 3H), 1.48 (s, 6H) ppm. **^{13}C NMR** (101 MHz, $CDCl_3$): δ = 148.59,

146.14, 144.82, 137.87, 122.31, 117.92, 112.41, 104.23, 103.88, 94.16, 38.27, 27.52 ppm. **FTIR**: ν_{max} (neat)/ cm^{-1} = 3414, 2969, 1592, 1454, 1245, 1037, 767. **HRMS (ESI)**: calculated for $C_{13}H_{16}NO$ ($[M+H]^+$) 202.1226; found 202.1227.

One-Pot Synthesis of Reverse-Allylated Products from Unsubstituted Indole:

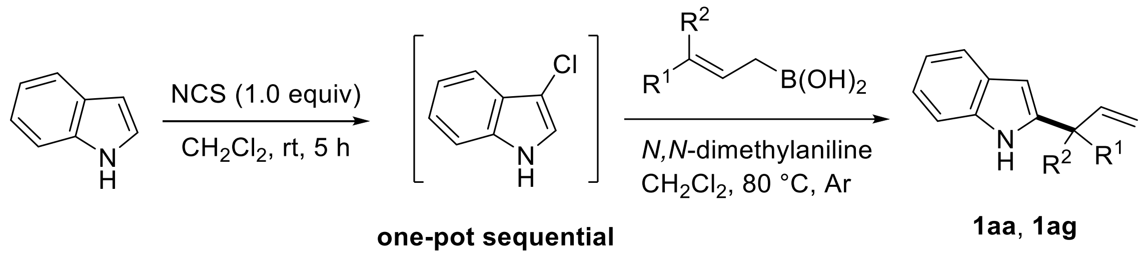

In a 10 mL screw cap seal reaction tube, to a solution of indole (23 mg, 0.2 mmol, 1.0 equiv) in CH_2Cl_2 (1 mL), *N*-chlorosuccinamide (NCS, 27 mg, 0.2 mmol, 1.0 equiv) was added and stirred at room temperature in a dark place. After completion of the reaction as indicated by TLC (5 hour), allyl boronic acid **4a** or **4g** in 1.5 mL CH_2Cl_2 (prepared from 1.0 mmol of allyl alcohol) and *N,N*-dimethylaniline (26 μL, 0.2 mmol, 1.0 equiv) were added. The seal tube was flushed with argon, and the reaction mixture was stirred at 80 °C oil bath temperature for 24 h. Upon consumption of the intermediate as indicated by TLC, reaction mixture was cooled down to room temperature, diluted with CH_2Cl_2 (15 mL) and washed with 1 (N) HCl (2 × 10 mL). The organic layer was washed with saturated aqueous $NaHCO_3$ solution once, dried over anhydrous Na_2SO_4, concentrated under reduced pressure and the crude residue was purified by silica gel column chromatography using ethyl acetate in hexane as eluent to obtain the pure desired allylated products **1aa** (27.5 mg, 74% over two steps) or **1ag** (31.5 mg, 62% over two steps), respectively.

General Procedure (GP IV) for One-Pot Sequential Electrophilic Substitution to Access 3-Substituted-2-Prenylated Indoles:

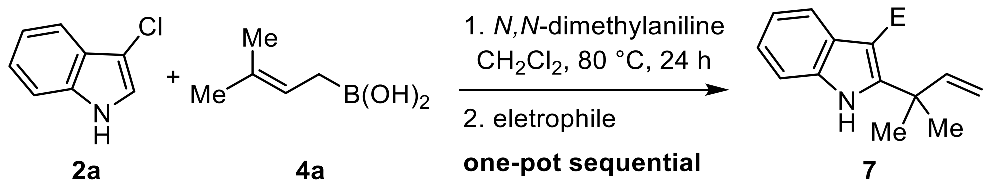

As mentioned in **GP III**, a 10 mL screw cap seal reaction tube was charged with 3-chloroindole **2a** (30 mg, 0.2 mmol, 1.0 equiv). To it, prenyl boronic acid **4a** in 2 mL CH_2Cl_2 (prepared from 1.0 mmol of prenyl alcohol) and *N,N*-dimethylaniline (26 μL, 0.2 mmol, 1.0 equiv) were added, and the resulting reaction mixture was stirred at 80 °C for 24 h. Upon consumption of the

starting material as indicated by TLC, the reaction mixture was cooled down to room temperature and treated with an electrophile.[1a,4] Upon complete consumption of the intermediate **1aa** as indicated by TLC, the reaction mixture was diluted with CH_2Cl_2 (20 mL) and washed with 1 (N) HCl (2 × 15 mL). The organic layer was washed with saturated aqueous $NaHCO_3$ solution once, dried over anhydrous Na_2SO_4, concentrated and purified by silica gel column chromatography using ethyl acetate in hexane as eluent to obtain the pure desired 3-substituted-2-prenylated indoles **7**.

3-Chloro-2-(2-methylbut-3-en-2-yl)-1*H*-indole (7a):

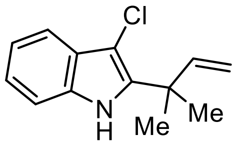

The titled compound **7a** was synthesized according to the **GP IV**. After the formation of intermediate **1aa**, *N*-chlorosuccinamide (NCS, 27 mg, 0.2 mmol, 1.0 equiv) was added and stirred at room temperature in a dark place for 4 h. Column chromatography on silica gel using 1% ethyl acetate in hexane as eluent provided the product **7a** (34 mg, 77% over two steps) as a colorless oil. **^{1}H NMR** (500 MHz, $CDCl_3$): δ = 7.93 (br s, 1H), 7.60 (d, *J* = 7.0 Hz, 1H), 7.30-7.29 (m, 1H), 7.22-7.16 (m, 2H), 6.18 (dd, *J* = 17.5, 11.0 Hz, 1H), 5.26-5.20 (m, 2H), 1.64 (s, 6H) ppm. **^{13}C NMR** (126 MHz, $CDCl_3$): δ = 144.45, 138.17, 132.92, 127.59, 122.50, 120.41, 117.75, 113.43, 110.92, 102.19, 38.86, 26.36 ppm. **FTIR**: ν_{max} (neat)/ cm^{-1} = 3438, 2969, 1456, 1234, 1035, 922, 740. **HRMS (ESI)**: calculated for $C_{13}H_{15}ClN$ ($[M+H]^+$) 220.0888; found 220.0886.

3-Bromo-2-(2-methylbut-3-en-2-yl)-1*H*-indole (7b):

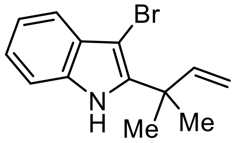

The titled compound **7b** was synthesized according to the **GP IV**. After the formation of intermediate **1aa**, *N*-bromosuccinamide (NBS, 53 mg, 0.3 mmol, 1.5 equiv) was added and stirred at room temperature in a dark place for 12 h.[4a] Column chromatography on silica gel using 0.5% ethyl acetate in hexane as eluent provided the product **7b** (32 mg, 61% over two steps) as a colorless oil. **^{1}H NMR** (400 MHz, $CDCl_3$): δ = 8.09 (br s, 1H), 7.55 (d, *J* = 7.2 Hz, 1H), 7.30 (d, *J* = 7.6 Hz, 1H), 7.22-7.15 (m, 2H), 6.18 (dd, *J* = 17.4, 10.6 Hz, 1H), 5.27-5.21 (m, 2H), 1.65 (s, 6H) ppm. **^{13}C NMR** (101 MHz, $CDCl_3$): δ = 144.37, 139.60, 133.44, 129.15, 122.59, 120.58, 118.75, 113.67, 110.86, 88.00, 39.08, 26.49 ppm. **FTIR**: ν_{max} (neat)/ cm^{-1} = 3434, 2969, 1454, 1231, 1020, 923, 740. **HRMS (ESI)**: calculated for $C_{13}H_{15}BrN$ ($[M+H]^+$) 264.0382; found 264.0379.

2-(2-Methylbut-3-en-2-yl)-3-(phenylthio)-1*H*-indole (7c):

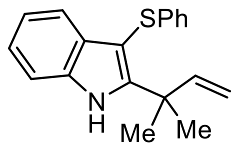

The titled compound **7c** was synthesized according to the **GP IV**. After the formation of intermediate **1aa**, sodium sulfinate (49 mg, 0.3 mmol, 1.5 equiv) and triphenylphosphine (79 mg, 0.3 mmol, 1.5 equiv) were added. To the reaction mixture, iodine (51 mg, 0.2 mmol, 1.0 equiv) was added and refluxed at 60 °C for 5 h.[4b] Column chromatography on silica gel using 3% ethyl acetate in hexane as eluent provided the product **7c** (44 mg, 75% over two steps) as white solid. **^{1}H NMR** (500 MHz, $CDCl_3$): δ = 8.30 (br s, 1H), 7.51 (d, *J* = 7.5 Hz, 1H), 7.36 (d, *J* = 8.5 Hz, 1H), 7.19 (t, *J* = 7.8 Hz, 1H), 7.14 (t, *J* = 7.8 Hz, 2H), 7.10 (t, *J* = 7.5 Hz, 1H), 7.04-7.01 (m, 3H), 6.24 (dd, *J* = 17.5, 10.5 Hz, 1H), 5.24-5.21 (m, 2H), 1.64 (s, 6H) ppm. **^{13}C NMR** (126 MHz, $CDCl_3$): δ = 148.92, 145.15, 139.86, 134.27, 131.77, 128.73, 125.42, 124.38, 122.49, 120.89, 119.24, 113.61, 110.96, 97.47, 39.70, 27.19 ppm. **FTIR**: ν_{max} (neat)/ cm^{-1} = 3429, 2964, 1581, 1453, 1260, 1023, 737. **HRMS (ESI)**: calculated for $C_{19}H_{20}NS$ ($[M+H]^+$) 294.1311; found 294.1310.

Bis(2-(2-methylbut-3-en-2-yl)-1*H*-indol-3-yl)methane (7d):

The titled compound **7d** was synthesized according to the **GP IV**. A solution of Me_2NH (37 μL, 40% solution in H_2O, 0.21 mmol, 1.05 equiv) and a solution of CH_2O (21 μL, 37% solution in H_2O, 0.21 mmol, 1.05 equiv) were sequentially added to AcOH (70 μL). Then the resulting mixture was stirred for 1 h and transferred dropwise to the intermediate **1aa** and final mixture was stirred at rt for 12 h.[4c] Column chromatography on silica gel using 4% ethyl acetate in hexane as eluent provided the product **7d** (20 mg, 51% over two steps) as a yellow oil. **^{1}H NMR** (500 MHz, $CDCl_3$): δ = 7.94 (br s, 2H), 7.25 (d, *J* = 8.5 Hz, 2H), 6.98 (t, *J* = 7.5 Hz, 2H), 6.86 (d, *J* = 8.0 Hz, 2H), 6.71 (t, *J* = 7.5 Hz, 2H), 6.21 (dd, *J* = 17.5, 10.5 Hz, 2H), 5.21 (d, *J* = 17.5 Hz, 2H), 5.17 (d, *J* = 10.5 Hz, 2H), 4.42 (s, 2H), 1.59 (s, 12H) ppm. **^{13}C NMR** (126 MHz, $CDCl_3$): δ = 146.00, 138.54, 134.25, 130.26, 120.90, 120.02, 118.98, 112.10, 110.16, 110.05, 39.11, 27.41, 23.07 ppm. **FTIR**: ν_{max} (neat)/ cm^{-1} = 3384, 2970, 1618, 1459, 1301, 1012, 916, 746. **HRMS (ESI)**: calculated for $C_{27}H_{31}N_2$ ($[M+H]^+$) 383.2482; found 383.2484.

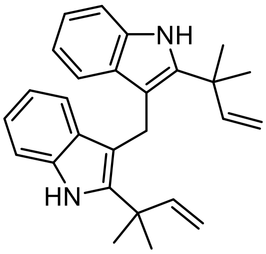

Total Synthesis of (±)-Mersicarpine:

General Procedure (GP V) to Access required Allylboronic Acid 4s-4t Used in the Synthesis of Mersicarpine:

First, a 250 mL two-neck round bottom flask was evacuated and backfilled with nitrogen. To it, butane-1,4-diol **A** (50 mmol, 4.5 g, 1.0 equiv) was taken and 100 mL dry THF was added. Then the reaction mixture was cooled down to 0 °C, and to the stirring solution, NaH (60% dispersion in mineral oil, 50 mmol, 2.0 g, 1.0 equiv) was added in portion under positive nitrogen pressure. The resulting mixture was stirred at room temperature for 30 min and again cooled down to 0 °C. After that, tetrabutylammonium iodide (TBAI, 5 mmol, 1.8 g, 0.1 equiv) was added followed by *p*-methoxybenzyl bromide or benzyl bromide (50 mmol, 1.0 equiv) and the resulting solution was stirred at room temperature for 12 h.[5a] After complete consumption of **A**, as indicated by TLC, the reaction mixture was quenched with water (60 mL) and the aqueous layer was extracted with ethyl acetate (3 x 60 mL). The combined organic layers were dried over anhydrous Na_2SO_4 and evaporated under reduced pressure. The crude residue was purified by silica gel column chromatography using 30% ethyl acetate in hexane as eluent to afford **B1** (9.6 g, 91%) or **B2** (8.6 g, 95%), respectively, as pale yellow oil.

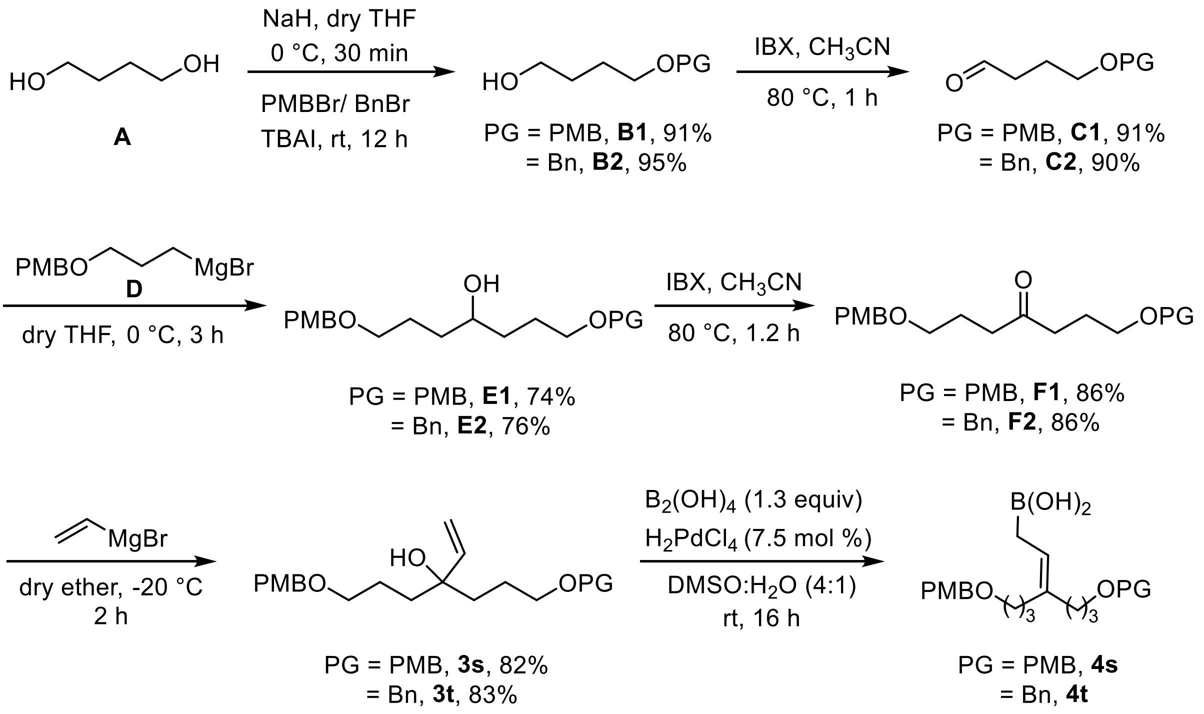

The mono-protected diol **B1** or **B2** (45 mmol, 1.0 equiv) was taken in a 250 mL round bottom flask and 140 mL acetonitrile was added. To this reaction mixture, IBX (50 mmol, 14 g, 1.1 equiv) was added and refluxed for 1 h at 80 °C. After complete consumption of starting material

as indicated by TLC, the reaction mixture was filtered through a short pad of celite and washed with ethyl acetate. The filtrates were then concentrated under reduced pressure and purified by silica gel column chromatography using 5% ethyl acetate in hexane as eluent to afford **C1** (8.4 g, 91%) or **C2** (7.2 g, 90%), respectively, as a colorless liquid.

Preparation of **E1** and **E2**: A two-neck round bottom flask was evacuated and backfilled with nitrogen. The process is repeated twice. Then, the flask was charged with aldehyde **C1** or **C2** (20 mmol, 1.0 equiv) and dry THF (60 mL) was added. The resulting solution was cooled down to 0 °C and freshly prepared (3-((4-methoxybenzyl)oxy)propyl)magnesium bromide **D** (24.0 mmol, 1.0 M in THF, 24 mL, 1.2 equiv) was added dropwise while maintaining constant stirring.[5b] The stirring was continued at the same temperature for another 3 h. After completion of the reaction as indicated by TLC, saturated aqueous NH_4Cl solution (40 mL) was added to the reaction mixture. The aqueous layer was extracted with ethyl acetate (3 x 40 mL), the combined organic layers were dried over anhydrous Na_2SO_4, and concentrated under reduced pressure. The crude residue was purified by silica gel column chromatography using 25% ethyl acetate in hexane as eluent to afford **E1** (5.7 g, 74%) or **E2** (5.4 g, 76%), respectively, as a colorless liquid.

The alcohol containing **E1** or **E2** (15 mmol, 1.0 equiv) was taken in a 100 mL round bottom flask and 50 mL of acetonitrile was added. To the resulting solution, IBX (18 mmol, 5 g, 1.2 equiv) was added and refluxed for 1.2 h at 80 °C. After complete consumption of starting material as indicated by TLC, the reaction mixture was filtered through a short pad of celite and washed with ethyl acetate. The filtrates were then concentrated under reduced pressure and purified by silica gel column chromatography using 10% ethyl acetate in hexane as eluent to afford ketone **F1** (5.0 g, 86%) or F**2** (4.6 g, 86%), respectively, as a pale yellow liquid.

Synthesis of quaternary allylic alcohols (3s and 3t): A long two-neck round bottom flask was evacuated and backfilled with nitrogen, charged with ketone **F1** or **F2** (10 mmol, 1.0 equiv) and dry ether (20 mL). The resulting solution was cooled down to -20 °C and vinylmagnesium bromide (15.0 mmol, 1.0 M in THF, 15 mL, 1.5 equiv) was added dropwise while maintaining constant stirring. The reaction mixture was stirred at same temperature for another 2 h. After completion of the reaction as indicated by TLC, saturated aqueous NH_4Cl solution (15 mL) was added to the reaction mixture and the aqueous layer was extracted with ethyl acetate (3 x 20 mL). The combined organic layers were dried over anhydrous Na_2SO_4 and concentrated under reduced pressure. The crude residue was purified by silica gel column chromatography using

15% ethyl acetate in hexane as eluent to afford **3s** (3.4 g, 82%) or **3t** (3.2 g, 76%), respectively, as a colorless liquid.

After the synthesis of allylic alcohols **3s** and **3t**, were subjected to **GP II** to prepare desired allylic boronic acids **4s** and **4t** by using H_2PdCl_4 catalyst (7.5 mol%) and diboronic acid (1.3 equiv).

1,7-Bis((4-methoxybenzyl)oxy)-4-vinylheptan-4-ol (3s):

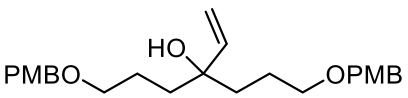

The titled compound **3s** was synthesized according to the **GP V**. Column chromatography on silica gel using 15% ethyl acetate in hexane as eluent provided the product **3s** (43% yield over five steps starting from **A**) as a colorless liquid. **^{1}H NMR** (500 MHz, $CDCl_3$): δ = 7.25 (d, *J* = 9.0 Hz, 4H), 6.87 (d, *J* = 9.0 Hz, 4H), 5.76 (dd, *J* = 17.3, 10.8 Hz, 1H), 5.21 (dd, *J* = 17.5, 1.5 Hz, 1H), 5.10 (dd, *J* = 11.0, 1.5 Hz, 1H), 4.43 (s, 4H), 3.80 (s, 6H), 3.47-3.41 (m, 4H), 2.95 (s, 1H), 1.71-1.59 (m, 8H) ppm. **^{13}C NMR** (126 MHz, $CDCl_3$): δ = 159.31, 143.88, 130.62, 129.34, 113.92, 113.06, 74.69, 72.63, 70.67, 55.37, 38.26, 24.11 ppm. **FTIR**: ν_{max} (neat)/ cm^{-1} = 3407, 2935, 2855, 1612, 1511, 1244, 1091, 818. **HRMS (ESI)**: calculated for $C_{25}H_{34}NaO_5$ ($[M+Na]^+$) 437.2298; found 437.2294.

1-(Benzyloxy)-7-((4-methoxybenzyl)oxy)-4-vinylheptan-4-ol (3t):

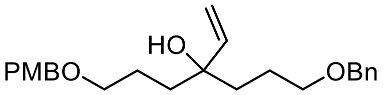

The titled compound **3t** was synthesized according to the **GP V**. Column chromatography on silica gel using 15% ethyl acetate in hexane as eluent provided the product **3t** (46% yield over five steps starting from **A**) as a colorless liquid. **^{1}H NMR** (500 MHz, $CDCl_3$): δ = 7.26-7.25 (m, 4H), 7.20 (dd, *J* = 6.0, 2.5 Hz, 1H), 7.17 (d, *J* = 8.5 Hz, 2H), 6.79 (d, *J* = 8.5 Hz, 2H), 5.68 (dd, *J* = 17.3, 10.8 Hz, 1H), 5.14 (dd, *J* = 17.3, 0.8 Hz, 1H), 5.03 (dd, *J* = 11.0, 1.0 Hz, 1H), 4.42 (s, 2H), 4.35 (s, 2H), 3.72 (s, 3H), 3.40-3.35 (m, 4H), 1.61-1.53 (m, 8H) ppm. **^{13}C NMR** (126 MHz, $CDCl_3$): δ = 13C NMR (126 MHz, CDCl3) δ 159.30, 143.84, 138.52, 130.57, 129.36, 128.47, 127.74, 127.65, 113.92, 113.10, 74.72, 72.98, 72.65, 70.97, 70.66, 55.38, 38.29, 38.22, 24.11 ppm. **FTIR**: ν_{max} (neat)/ cm^{-1} = 3412, 2934, 2856, 1612, 1513, 1247, 1096, 820. **HRMS (ESI)**: calculated for $C_{24}H_{33}O_4$ ($[M+H]^+$) 385.2373; found 385.2376.

2-(1,7-Bis((4-methoxybenzyl)oxy)-4-vinylheptan-4-yl)-1*H*-indole (1as):

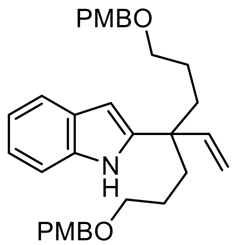

The titled compound **1as** was synthesized according to the **GP III**. Column chromatography on silica gel using 12% ethyl acetate in hexane as eluent provided the product **1as** (72 mg, 70%) as a colorless oil. **^{1}H NMR** (400 MHz, $CDCl_3$): δ = 8.16 (br s, 1H), 7.53 (d, *J* = 7.6 Hz, 1H), 7.24 (d, *J* = 8.4 Hz, 4H), 7.16 (d, *J* = 7.6 Hz, 1H), 7.11-7.03 (m, 2H), 6.88 (d, *J* = 8.8 Hz, 4H), 6.32 (s, 1H), 5.97 (dd, *J* = 17.6, 10.8 Hz, 1H), 5.22 (d, *J* = 10.8 Hz, 1H), 5.16 (d, *J* = 17.2 Hz, 1H), 4.40 (s, 4H), 3.81 (s, 6H), 3.41 (t, *J* = 6.2 Hz, 4H), 1.90-1.86 (m, 4H), 1.58-1.46 (m, 4H) ppm. **^{13}C NMR** (126 MHz, $CDCl_3$): δ = 159.36, 144.06, 143.30, 135.97, 130.83, 129.38, 128.66, 121.22, 120.11, 119.58, 114.39, 113.98, 110.69, 100.39, 72.70, 70.49, 55.43, 44.91, 33.81, 24.65 ppm. **FTIR**: ν_{max} (neat)/ cm^{-1} = 3375, 2933, 2857, 1612, 1513, 1457, 1247, 1095, 819. **HRMS (ESI)**: calculated for $C_{33}H_{40}NO_4$ ($[M+H]^+$) 514.2952; found 514.2953.

2-(1-(Benzyloxy)-7-((4-methoxybenzyl)oxy)-4-vinylheptan-4-yl)-1*H*-indole (1at):

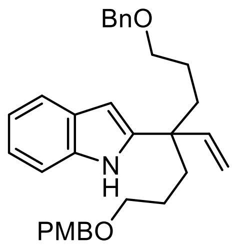

The titled compound **1at** was synthesized according to the **GP III**. Column chromatography on silica gel using 10% ethyl acetate in hexane as eluent provided the product **1at** (72 mg, 74%) as a colorless oil. **^{1}H NMR** (400 MHz, $CDCl_3$): δ = 8.15 (br s, 1H), 7.54 (d, *J* = 7.6 Hz, 1H), 7.37-7.32 (m, 5H), 7.25 (d, *J* = 8.8 Hz, 2H), 7.17 (d, *J* = 7.6 Hz, 1H), 7.12-7.04 (m, 2H), 6.88 (d, *J* = 8.8 Hz, 2H), 6.33 (s, 1H), 5.98 (dd, *J* = 17.6, 10.8 Hz, 1H), 5.24 (d, *J* = 10.8 Hz, 1H), 5.17 (d, *J* = 17.6 Hz, 1H), 4.48 (s, 2H), 4.41 (s, 2H), 3.81 (s, 3H), 3.46-3.41 (m, 4H), 1.94-1.85 (m, 4H), 1.61-1.51 (m, 4H) ppm. **^{13}C NMR** (101 MHz, $CDCl_3$): δ = 159.27, 144.01, 143.25, 138.62, 135.89, 130.70, 129.41, 128.56, 128.52, 127.80, 127.69, 121.21, 120.09, 119.55, 114.43, 113.90, 110.68, 100.31, 73.03, 72.68, 70.75, 70.44, 55.41, 44.82, 33.60, 33.57, 24.57 ppm. **FTIR**: ν_{max} (neat)/ cm^{-1} = 3352, 2925, 2855, 1613, 1512, 1457, 1246, 1095, 786. **HRMS (ESI)**: calculated for $C_{32}H_{38}NO_3$ ($[M+H]^+$) 484.2846; found 484.2859.

2-(1,7-Bis((4-methoxybenzyl)oxy)-4-vinylheptan-4-yl)-3-chloro-1*H*-indole (7e):

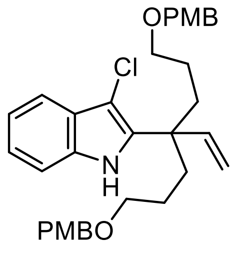

The titled compound **7e** was synthesized according to the **GP IV**. After the formation of intermediate **1as**, *N*-chlorosuccinamide (NCS, 27 mg, 0.2 mmol, 1.0 equiv) was added and stirred at room temperature in a dark place for 3 h. Column chromatography on silica gel using 10% ethyl acetate in hexane as eluent provided the product **7e** (69 mg, 63% over two steps) as a colorless oil. **^{1}H NMR** (500 MHz, $CDCl_3$): δ = 9.05 (s, 1H), 7.53 (d, *J* = 7.5 Hz, 1H), 7.27 (d,

J = 8.0 Hz, 4H), 7.11-7.08 (m, 1H), 7.07-7.04 (m, 1H), 6.88 (d, *J* = 9.0 Hz, 4H), 6.75 (d, *J* = 8.0 Hz, 1H), 6.03 (dd, *J* = 17.8, 10.8 Hz, 1H), 5.25 (d, *J* = 11.0 Hz, 1H), 5.12 (d, *J* = 17.5 Hz, 1H), 4.44 (s, 4H), 3.81 (s, 6H), 3.51-3.42 (m, 4H), 2.14-2.08 (m, 2H), 2.07-2.01 (m, 2H), 1.65-1.60 (m, 2H), 1.56-1.49 (m, 2H) ppm. **^{13}C NMR** (126 MHz, $CDCl_3$): δ = 159.50, 141.40, 136.12, 133.41, 130.65, 129.57, 127.20, 122.04, 120.01, 117.47, 114.84, 114.07, 111.10, 103.09, 72.97, 70.54, 55.46, 46.07, 33.65, 25.16 ppm. **FTIR**: ν_{max} (neat)/ cm^{-1} = 3003, 2834, 1610, 1510, 1245, 1176, 1035, 810. **HRMS (ESI)**: calculated for $C_{33}H_{39}ClNO_4$ ($[M+H]^+$) 548.2562; found 548.2544.

2-(1-(Benzyloxy)-7-((4-methoxybenzyl)oxy)-4-vinylheptan-4-yl)-3-chloro-1*H*-indole (7f):

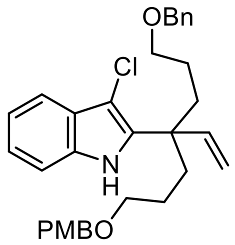

The titled compound **7f** was synthesized according to the **GP IV**. After the formation of intermediate **1at**, *N*-chlorosuccinamide (NCS, 27 mg, 0.2 mmol, 1.0 equiv) was added and stirred at room temperature in a dark place for 3 h. Column chromatography on silica gel using 8% ethyl acetate in hexane as eluent provided the product **7f** (71 mg, 69% over two steps) as a colorless oil. **^{1}H NMR** (400 MHz, $CDCl_3$): δ = 9.07 (br s, 1H), 7.55 (d, *J* = 7.6 Hz, 1H), 7.37-7.33 (m, 5H), 7.29 (d, *J* = 8.4 Hz, 2H), 7.13-7.05 (m, 2H), 6.90 (d, *J* = 8.4 Hz, 2H), 6.76 (d, *J* = 7.6 Hz, 1H), 6.05 (dd, *J* = 17.6, 10.8 Hz, 1H), 5.27 (d, *J* = 10.8 Hz, 1H), 5.14 (d, *J* = 17.2 Hz, 1H), 4.52 (s, 2H), 4.46 (s, 2H), 3.82 (s, 3H), 3.54-3.45 (m, 4H), 2.17-2.01 (m, 4H), 1.64-1.54 (m, 4H) ppm. **^{13}C NMR** (126 MHz, $CDCl_3$): δ = 159.45, 141.33, 138.47, 136.07, 133.36, 130.57, 129.56, 128.57, 127.94, 127.79, 127.15, 122.03, 119.99, 117.44, 114.86, 114.03, 111.06, 103.04, 73.28, 72.95, 70.80, 70.49, 55.42, 46.02, 33.63, 33.54, 25.13 ppm. **FTIR**: ν_{max} (neat)/ cm^{-1} = 3298, 2934, 2858, 1613, 1513, 1248, 1096, 743. **HRMS (ESI)**: calculated for $C_{32}H_{37}ClNO_3$ ($[M+H]^+$) 518.2456; found 518.2476.

4-(3-Chloro-*1H*-indol-2-yl)-7-((4-methoxybenzyl)oxy)-4-vinylheptan-1-ol (10a):

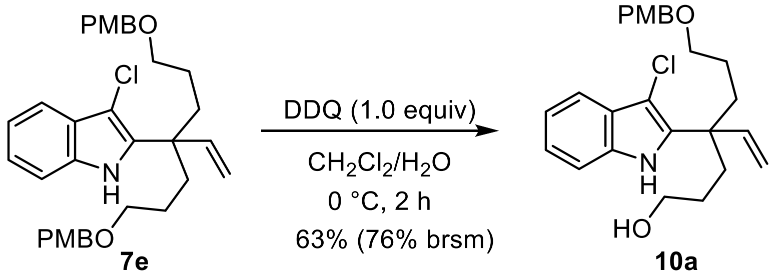

To a solution of **7e** (274 mg, 0.5 mmol, 1.0 equiv) in the mixture of CH_2Cl_2 (10 mL) and H_2O (0.4 mL) was added DDQ (114 mg, 0.5 mmol, 1.0 equiv) at 0 °C and the resulting mixture was stirred for 2 h at the same temperature. The reaction was quenched with saturated $NaHCO_3$ (10

mL), and extracted with CH_2Cl_2 (3 x 10 mL). The combined organic layers were washed with brine and dried over anhydrous Na_2SO_4, and concentrated under reduced pressure. Column chromatography on silica gel using 30% ethyl acetate in hexane as eluent provided the product **10a** (135 mg, 63%) as a pale yellow oil along with recovered starting material **7e** (46 mg). **^{1}H NMR** (500 MHz, $CDCl_3$): δ = 8.86 (br s, 1H), 7.56-7.54 (m, 1H), 7.28 (d, *J* = 8.5 Hz, 2H), 7.12-7.10 (m, 2H), 6.98-6.96 (m, 1H), 6.89 (d, *J* = 9.0 Hz, 2H), 6.05 (dd, *J* = 17.5, 10.5 Hz, 1H), 5.29 (dd, *J* = 11.0, 0.5 Hz, 1H), 5.16 (dd, *J* = 17.5, 0.5 Hz, 1H), 4.46 (s, 2H), 3.81 (s, 3H), 3.60 (td, *J* = 6.4, 1.7 Hz, 2H), 3.54-3.50 (m, 1H), 3.49-3.45 (m, 1H), 2.19-2.13 (m, 1H), 2.12-2.02 (m, 2H), 2.01-1.96 (m, 1H), 1.57-1.49 (m, 2H), 1.46-1.38 (m, 2H) ppm. **^{13}C NMR** (126 MHz, $CDCl_3$): δ = 159.55, 141.40, 136.03, 133.39, 130.54, 129.63, 127.23, 122.21, 120.14, 117.57, 115.09, 114.11, 111.07, 103.06, 73.06, 70.48, 63.28, 55.48, 45.97, 33.58, 33.33, 27.79, 25.23 ppm. **FTIR**: ν_{max} (neat)/ cm^{-1} = 3297, 2927, 2859, 1613, 1513, 1248, 1034, 771. **HRMS (ESI)**: calculated for $C_{25}H_{31}ClNO_3$ ($[M+H]^+$) 428.1987; found 428.1993.

7-(Benzyloxy)-4-(3-chloro-1*H*-indol-2-yl)-4-vinylheptan-1-ol (10b):

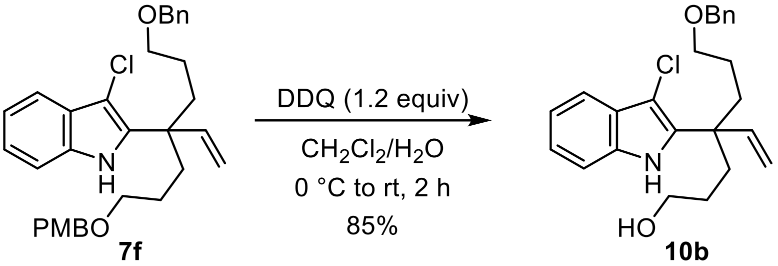

To a solution of **7f** (259 mg, 0.5 mmol, 1.0 equiv) in the mixture of CH_2Cl_2 (10 mL) and H_2O (0.4 mL) was added DDQ (136 mg, 0.6 mmol, 1.2 equiv) at 0 °C, and the mixture was slowly warmed to room temperature and stirred for 2 h. The reaction was quenched with saturated $NaHCO_3$ (10 mL), and extracted with CH_2Cl_2 (3 x 10 mL). The combined organic layers were washed with brine and dried over anhydrous Na_2SO_4, and concentrated under reduced pressure. Column chromatography on silica gel using 27% ethyl acetate in hexane as eluent provided the product **10b** (169 mg, 85%) as a pale yellow oil. **^{1}H NMR** (400 MHz, $CDCl_3$): δ = 8.83 (br s, 1H), 7.56-7.54 (m, 1H), 7.37-7.30 (m, 5H), 7.14-7.08 (m, 2H), 6.97-6.95 (m, 1H), 6.05 (dd, *J* = 17.6, 10.8 Hz, 1H), 5.30 (d, *J* = 10.8 Hz, 1H), 5.17 (d, *J* = 17.6 Hz, 1H), 4.53 (s, 2H), 3.61 (t, *J* = 6.4 Hz, 2H), 3.56-3.47 (m, 2H), 2.22-2.14 (m, 1H), 2.12-2.05 (m, 2H), 2.03-1.97 (m, 1H), 1.58-1.39 (m, 4H) ppm. **^{13}C NMR** (101 MHz, $CDCl_3$): δ = 141.30, 138.26, 135.99, 133.30, 128.63, 128.03, 127.91, 127.12, 122.18, 120.09, 117.50, 115.08, 111.05, 102.92, 73.36, 70.74, 63.16, 45.85, 33.42, 33.21, 27.65, 25.12 ppm. **FTIR**: ν_{max} (neat)/ cm^{-1} = 3312, 2925, 2856,

1457, 1055, 918, 743. **HRMS (ESI)**: calculated for $C_{24}H_{29}ClNO_2$ ($[M+H]^+$) 398.1881; found 398.1890.

10-Chloro-9-(3-((4-methoxybenzyl)oxy)propyl)-9-vinyl-8,9-dihydropyrido[1,2-*a*]indol-6(7*H*)-one (11a):

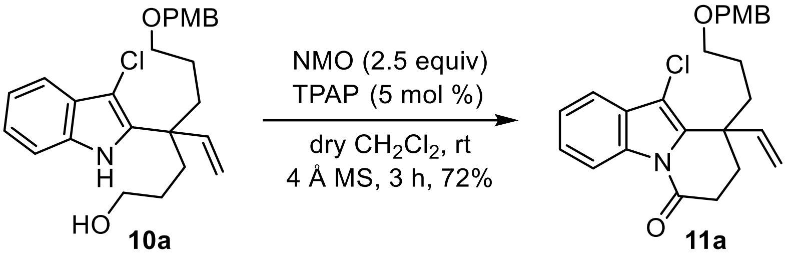

A two-neck round bottom flask was evacuated, backfilled with nitrogen, and charged with activated powdered 4Å molecular sieves (300 mg), alcohol **10a** (214 mg, 0.5 mmol, 1.0 equiv), and 10 mL of anhydrous CH_2Cl_2. To the resulting solution, *N*-methylmorpholine-*N*-oxide (NMO, 146 mg, 1.25 mmol, 2.5 equiv) was added and stirred for 10 min. Then tetrapropylammonium perruthenate (TPAP, 9 mg, 0.025 mmol, 0.05 equiv) was added and reaction mixture was stirred at room temperature for 3 h. After complete consumption of starting material as indicated by TLC, the reaction mixture was filtered through a short pad of celite and washed with a large amount of CH_2Cl_2. The filtrates were then washed with 10% Na_2SO_3 (20 mL) and brine, and then dried over anhydrous Na_2SO_4, concentrated under reduced pressure and purified by silica gel column chromatography using 10% ethyl acetate in hexane as eluent to afford **11a** (153 mg, 72%) as a colorless oil. **^{1}H NMR** (400 MHz, $CDCl_3$): δ = 8.50 (dd, *J* = 7.2, 1.2 Hz, 1H), 7.58-7.55 (m, 1H), 7.41-7.33 (m, 2H), 7.24 (d, *J* = 8.4 Hz, 2H), 6.87 (d, *J* = 8.4 Hz, 2H), 5.88 (dd, *J* = 17.6, 10.4 Hz, 1H), 5.19 (d, *J* = 10.4 Hz, 1H), 4.82 (d, *J* = 17.2 Hz, 1H), 4.43 (s, 2H), 3.80 (s, 3H), 3.54-3.44 (m, 2H), 2.85-2.69 (m, 2H), 2.52 (ddd, *J* = 14.0, 12.0, 4.6 Hz, 1H), 2.31 (td, *J* = 13.1, 4.3 Hz, 1H), 1.96-1.83 (m, 2H), 1.72-1.61 (m, 2H) ppm. **^{13}C NMR** (101 MHz, $CDCl_3$): δ = 168.99, 159.27, 140.48, 134.59, 133.64, 130.67, 129.34, 128.08, 126.03, 124.50, 117.81, 116.77, 115.66, 113.90, 111.37, 72.75, 70.25, 55.42, 43.74, 34.51, 30.75, 24.69 ppm. **FTIR**: ν_{max} (neat)/ cm^{-1} = 2931, 2857, 1713, 1513, 1454, 1345, 1247, 1181, 1034. **HRMS (ESI)**: calculated for $C_{25}H_{27}ClNO_3$ ($[M+H]^+$) 424.1674; found 424.1681.

9-(3-(Benzyloxy)propyl)-10-chloro-9-vinyl-8,9-dihydropyrido[1,2-*a]*indol-6(7*H*)-one (11b):

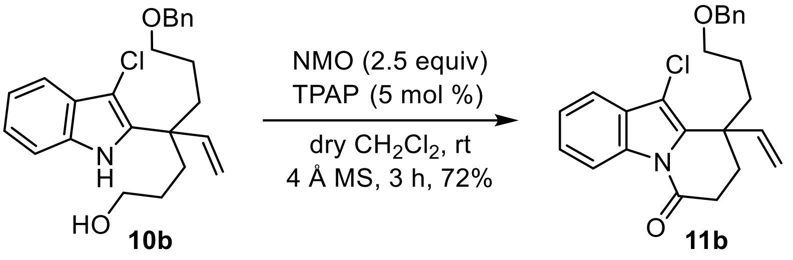

A two-neck round bottom flask was evacuated, backfilled with nitrogen and charged with activated powdered 4Å molecular sieves (300 mg), alcohol **10b** (199 mg, 0.5 mmol, 1.0 equiv), and 10 mL of anhydrous CH_2Cl_2. To the resulting solution, *N*-methylmorpholine-*N*-oxide (NMO, 146 mg, 1.25 mmol, 2.5 equiv) was added and stirred for 10 min. Then tetrapropylammonium perruthenate (TPAP, 9 mg, 0.025 mmol, 0.05 equiv) was added and reaction mixture was stirred at room temperature for 3 h. After complete consumption of starting material as indicated by TLC, the reaction mixture was filtered through a short pad of celite and washed with a large amount of CH_2Cl_2. The filtrates were then washed with 10% Na_2SO_3 (20 mL) and brine, dried over anhydrous Na_2SO_4, concentrated under reduced pressure and purified by silica gel column chromatography using 7% ethyl acetate in hexane as eluent to afford **11b** (142 mg, 72%) as a colorless oil. **^{1}H NMR** (500 MHz, $CDCl_3$): δ = 8.52 (d, *J* = 8.0 Hz, 1H), 7.58 (d, *J* = 7.5 Hz, 1H), 7.41-7.34 (m, 6H), 7.30-7.27 (m, 1H), 5.91 (dd, *J* = 17.5, 10.5 Hz, 1H), 5.20 (d, *J* = 10.5 Hz, 1H), 4.85 (d, *J* = 17.5 Hz, 1H), 4.51 (s, 2H), 3.58-3.50 (m, 2H), 2.85-2.79 (m, 1H), 2.74 (dt, *J* = 17.3, 4.1 Hz, 1H), 2.58-2.52 (m, 1H), 2.32 (td, *J* = 13.1, 4.3 Hz, 1H), 1.96 (td, *J* = 13.0, 4.8 Hz, 1H), 1.88 (dt, *J* = 13.7, 4.5 Hz, 1H), 1.70-1.62 (m, 2H) ppm. **^{13}C NMR** (126 MHz, $CDCl_3$): δ = 168.87, 140.47, 138.61, 134.64, 133.65, 128.47, 128.08, 127.68, 127.65, 126.00, 124.47, 117.78, 116.76, 115.59, 111.31, 73.07, 70.53, 43.67, 34.50, 30.73, 30.72, 24.70 ppm. **FTIR**: ν_{max} (neat)/ cm^{-1} = 2924, 2856, 1715, 1455, 1346, 1184, 1100, 751. **HRMS (ESI)**: calculated for $C_{24}H_{25}ClNO_2$ ($[M+H]^+$) 394.1568; found 394.1574.

9-(3-Azidopropyl)-10-chloro-9-ethyl-8,9-dihydropyrido[1,2-*a*]indol-6(7*H*)-one (13):

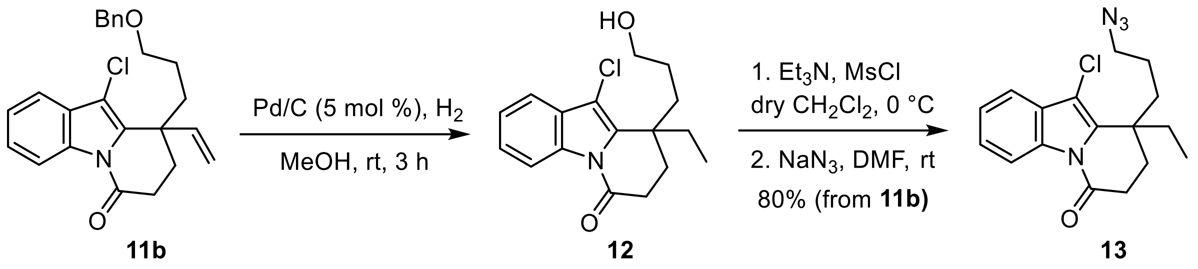

To a solution of **11b** (158 mg, 0.4 mmol, 1.0 equiv) in MeOH (10 mL) was added Pd/C (21 mg, 0.02 mmol, 0.05 equiv). The reaction mixture was purged with H_2 gas and stirred under an atmosphere of H_2 (applied through balloon filled with hydrogen gas) for 3 h. After complete consumption of starting material as indicated by TLC, the reaction mixture was filtered through a pad of celite, and washed with ethyl acetate (20 mL). The filtrates were then concentrated under reduced pressure and the crude residue of **12** was used directly in the next step without further purification.

The analytically pure sample **12** was obtained by silica gel column chromatography using 25% ethyl acetate in hexane as eluent as a colorless oil (110 mg, 90%). **^{1}H NMR** (400 MHz, $CDCl_3$): δ = 8.51-8.49 (m, 1H), 7.53 (dd, *J* = 6.0, 1.6 Hz, 1H), 7.39-7.32 (m, 2H), 3.68 (t, *J* = 6.2 Hz, 2H), 2.83 (t, *J* = 6.6 Hz, 2H), 2.27-2.11 (m, 2H), 2.03 (t, *J* = 6.6 Hz, 2H), 1.89-1.77 (m, 2H), 1.60-1.56 (m, 2H), 0.95 (t, *J* = 7.6 Hz, 3H) ppm. **^{13}C NMR** (126 MHz, $CDCl_3$): δ = 168.89, 137.34, 133.69, 128.25, 125.88, 124.51, 117.69, 116.81, 110.12, 63.33, 39.43, 33.36, 30.40, 30.19, 29.00, 27.73, 8.70 ppm. **FTIR**: ν_{max} (neat)/ cm^{-1} = 3415, 2936, 1711, 1455, 1345, 1185, 1060, 768. **HRMS (ESI)**: calculated for $C_{17}H_{21}ClNO_2$ ($[M+H]^+$) 306.1255; found 306.1253.

Synthesis of (13): A solution of crude **12** in dry CH_2Cl_2 (5 mL) was cooled down to 0 °C, and Et_3N (84 µL, 0.6 mmol, 1.5 equiv) followed by MsCl (37 µL, 0.48 mmol, 1.2 equiv) were added dropwise. The resulting solution was stirred at the same temperature for 1 h, then quenched with saturated $NaHCO_3$ solution and extracted with CH_2Cl_2 (3 x 10 mL). The combined organic layers were washed with brine and dried over anhydrous Na_2SO_4, and concentrated under reduced pressure to obtain the crude mesylated product, which was used directly for the next step without further purification.

To the solution of the crude mesylated product in DMF (5 mL) was added NaN_3 (130 mg, 2.0 mmol, 5.0 equiv). The reaction was covered with aluminum foil and stirred at room temperature for 12 h. After completion of the reaction as indicated by TLC, the reaction was quenched with water and extracted with EtOAc (3 x 15 mL). The combined organic layers were washed with

brine and cold water, dried over anhydrous Na_2SO_4, and concentrated under reduced pressure. Column chromatography on silica gel using 6% ethyl acetate in hexane as eluent provided the product **13** (106 mg, 80% over three steps) as a colorless oil. **^{1}H NMR** (400 MHz, $CDCl_3$): δ = 8.50 (dd, *J* = 6.8, 1.6 Hz, 1H), 7.56-7.53 (m, 1H), 7.40-7.33 (m, 2H), 3.33 (t, *J* = 6.6 Hz, 2H), 2.85-2.82 (m, 2H), 2.30-2.22 (m, 1H), 2.13 (dq, *J* = 14.8, 7.2 Hz, 1H), 2.03 (t, *J* = 6.8 Hz, 2H), 1.89-1.75 (m, 2H), 1.65-1.60 (m, 2H), 0.96 (t, *J* = 7.6 Hz, 3H) ppm. **^{13}C NMR** (101 MHz, $CDCl_3$): δ = 168.74, 136.83, 133.61, 128.09, 125.98, 124.55, 117.73, 116.78, 110.23, 51.96, 39.35, 34.09, 30.27, 30.17, 28.93, 24.10, 8.65 ppm. **FTIR**: ν_{max} (neat)/ cm^{-1} = 2925, 2856, 2096, 1714, 1455, 1346, 1185, 752. **HRMS (ESI)**: calculated for $C_{17}H_{20}ClN_4O$ ($[M+H]^+$) 331.1320; found 331.1317.

9-(3-Aminopropyl)-10-chloro-9-ethyl-8,9-dihydropyrido[1,2-*a*]indol-6(7*H*)-one (14):

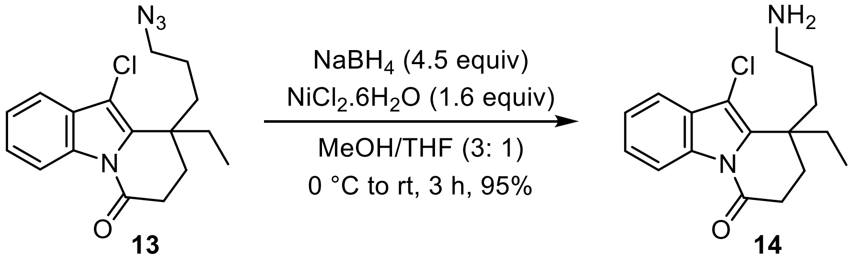

Compound **13** (66 mg, 0.2 mmol, 1.0 equiv) was dissolved in a mixture of MeOH (3 mL) and THF (1 mL), cooled down to 0 °C. Then, $NiCl_2{\cdot}6H_2O$ (76 mg, 0.32 mmol, 1.6 equiv) and $NaBH_4$ (34 mg, 0.9 mmol, 4.5 equiv) were added sequentially and stirred at 0 °C for 10 min. Then the reaction mixture was warmed to room temperature and stirred for another 3 h. After complete consumption of starting material as indicated by TLC, the reaction mixture was filtered through a short pad of celite and washed with a large amount of ethyl acetate. The filtrates were then washed with brine, dried over Na_2SO_4, concentrated under reduced pressure and purified by silica gel column chromatography using 8% MeOH in CH_2Cl_2 as eluent to afford **14** (58 mg, 95%) as a colorless oil. **^{1}H NMR** (500 MHz, $CDCl_3$): δ = 8.42 (d, *J* = 8.0 Hz, 1H), 8.03 (br s, 2H), 7.46 (d, *J* = 7.0 Hz, 1H), 7.30-7.24 (m, 2H), 2.88-2.83 (m, 3H), 2.70-2.67 (m, 1H), 2.12-2.06 (m, 2H), 1.93-1.86 (m, 2H), 1.79-1.69 (m, 4H), 0.88 (t, *J* = 7.5 Hz, 3H) ppm. **^{13}C NMR** (126 MHz, $CDCl_3$): δ = 168.98, 136.69, 133.59, 128.03, 125.98, 124.55, 117.73, 116.76, 110.10, 40.34, 39.33, 33.85, 30.27, 29.89, 28.63, 22.45, 8.66 ppm. **FTIR**: ν_{max} (neat)/ cm^{-1} = 3410, 2925, 1709, 1455, 1345, 1188, 752. **HRMS (ESI)**: calculated for $C_{17}H_{22}ClN_2O$ ($[M+H]^+$) 305.1415; found 305.1421.

(±)-Mersicarpine (15):

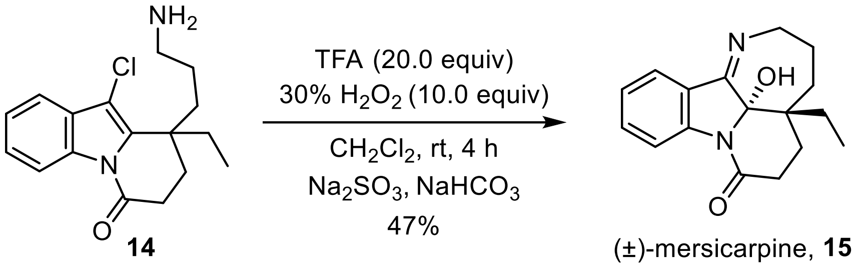

A solution of **14** (30 mg, 0.1 mmol, 1.0 equiv) in CH_2Cl_2 (2.5 mL) was cooled down to 0 °C, CF_3CO_2H (0.15 mL, 2.0 mmol, 20.0 equiv) was added followed by 30% H_2O_2 (0.1 mL, 1.0 mmol, 10.0 equiv). The resulting mixture was stirred for 4 h at room temperature, then quenched with a saturated aqueous Na_2SO_3 solution. The pH of the mixture was adjusted to 4~5 by addition of saturated aqueous $NaHCO_3$ solution and stirred vigorously for 15 min, and then extracted with CH_2Cl_2 (2 x 10 mL). The combined organic layers were washed with brine, dried over anhydrous Na_2SO_4, and concentrated under reduced pressure. Column chromatography on silica gel using 25% ethyl acetate in hexane as eluent provided **(±)-mersicarpine** (13.5 mg, 47%) as a white solid. **^{1}H NMR** (600 MHz, base-washed $CDCl_3$): δ = 8.16 (d, *J* = 7.8 Hz, 1H), 7.60 (d, *J* = 7.2 Hz, 1H), 7.38 (t, *J* = 7.5 Hz, 1H), 7.08 (t, *J* = 6.9 Hz, 1H), 4.74 (br s, 1H), 3.94-3.85 (m, 2H), 2.60 (dd, *J* = 18.3, 9.3 Hz, 1H), 2.44-2.38 (m, 1H), 2.07 (t, *J* = 12.9 Hz, 1H), 1.95-1.90 (m, 1H), 1.77-1.67 (m, 4H), 1.37-1.32 (m, 1H), 1.15-1.10 (m, 1H), 0.75 (t, *J* = 6.6 Hz, 3H) ppm. **^{13}C NMR** (151 MHz, base-washed $CDCl_3$): δ = 169.59, 168.72, 146.67, 133.43, 124.52, 124.50, 122.30, 116.96, 93.94, 50.78, 39.42, 34.47, 29.32, 25.57, 23.09, 21.28, 6.99 ppm. **FTIR**: ν_{max} (neat)/ cm^{-1} = 3225, 2926, 1661, 1468, 1391, 1070, 916, 755. **HRMS (ESI)**: calculated for $C_{17}H_{21}N_2O_2$ ($[M+H]^+$) 285.1598; found 285.1603.

9-Ethyl-9-(3-hydroxypropyl)-8,9-dihydropyrido[1,2-*a*]indol-6(7*H*)-one (16):

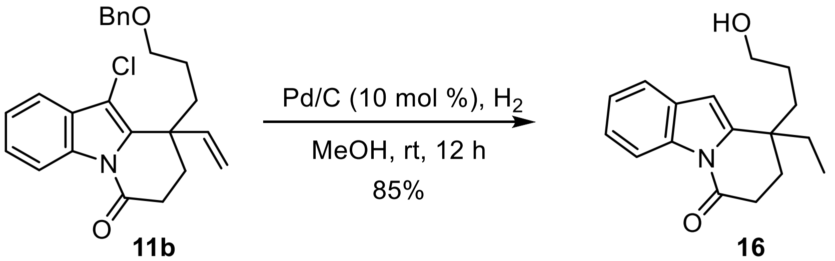

To a solution of **11b** (158 mg, 0.4 mmol, 1.0 equiv) in MeOH (8 mL) was added Pd/C (43 mg, 0.04 mmol, 0.1 equiv). The reaction mixture was purged with H_2 gas and stirred under an atmosphere of H_2 (applied through balloon filled with hydrogen gas) for 12 h. After complete consumption of starting material as indicated by TLC, the reaction mixture was filtered through

a pad of celite, and washed with ethyl acetate (20 mL). The filtrates were then concentrated under reduced pressure and purified by silica gel column chromatography using 25% ethyl acetate in hexane as eluent to afford **16** (92 mg, 85%) as a colorless oil. **^{1}H NMR** (500 MHz, $CDCl_3$): δ = 8.48 (d, *J* = 8.0 Hz, 1H), 7.47 (d, *J* = 7.5 Hz, 1H), 7.30-7.23 (m, 2H), 6.31 (s, 1H), 3.64 (t, *J* = 6.3 Hz, 2H), 2.86 (t, *J* = 6.5 Hz, 2H), 1.98 (t, *J* = 6.5 Hz, 2H), 1.83-1.70 (m, 4H), 1.61-1.50 (m, 2H), 1.41 (br s, 1H), 0.92 (t, *J* = 7.3 Hz, 3H) ppm. **^{13}C NMR** (126 MHz, $CDCl_3$): δ = 169.23, 144.19, 135.39, 129.69, 124.38, 124.07, 119.98, 116.66, 105.29, 63.25, 37.55, 33.15, 30.48, 30.19, 30.16, 27.12, 8.18 ppm. **FTIR**: ν_{max} (neat)/ cm^{-1} = 3419, 2971, 1704, 1455, 1351, 1185, 1061, 755. **HRMS (ESI)**: calculated for $C_{17}H_{22}NO_2$ ($[M+H]^+$) 272.1645; found 272.1645.

9-(3-Azidopropyl)-9-ethyl-8,9-dihydropyrido[1,2-*a*]indol-6(7*H*)-one (17):

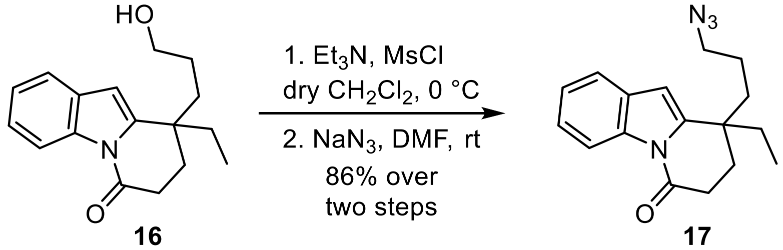

A solution of **16** (140 mg, 0.4 mmol, 1.0 equiv) in dry CH_2Cl_2 (5 mL) was cooled down to 0 °C, and Et_3N (84 µL, 0.6 mmol, 1.5 equiv) followed by MsCl (37 µL, 0.48 mmol, 1.2 equiv) were added. The resulting solution was stirred at the same temperature for 1 h, then quenched with saturated $NaHCO_3$ and extracted with CH_2Cl_2 (3 x 10 mL). The combined organic layers were washed with brine and dried over anhydrous Na_2SO_4, and concentrated under reduced pressure to obtain the crude mesylated product, which was used directly for the next step without further purification.

To the solution of the crude mesylated product in DMF (5 mL) was added NaN_3 (130 mg, 2.0 mmol, 5.0 equiv). The reaction was covered with aluminum foil and stirred at room temperature for 12 h. After completion of the reaction as indicated by TLC, the reaction was quenched with water and extracted with EtOAc (3 x 15 mL). The combined organic layers were washed with brine and cold water, dried over anhydrous Na_2SO_4, and concentrated under reduced pressure. Column chromatography on silica gel using 5% ethyl acetate in hexane as eluent provided **17** (101 mg, 85%) as a colorless oil. **^{1}H NMR** (500 MHz, $CDCl_3$): δ = 8.49 (d, *J* = 8.0 Hz, 1H), 7.49 (d, *J* = 7.5 Hz, 1H), 7.31-7.24 (m, 2H), 6.32 (s, 1H), 3.35-3.24 (m, 2H), 2.87 (t, *J* = 6.8 Hz, 2H), 1.99 (t, *J* = 6.5 Hz, 2H), 1.84-1.70 (m, 4H), 1.69-1.62 (m, 1H), 1.58-1.53 (m, 1H), 0.93 (t,

J = 7.5 Hz, 3H) ppm. **^{13}C NMR** (126 MHz, $CDCl_3$): δ = 169.00, 143.71, 135.40, 129.59, 124.50, 124.12, 120.05, 116.67, 105.35, 51.96, 37.61, 34.03, 30.42, 30.18, 23.60, 8.16 ppm. **FTIR**: ν_{max} (neat)/ cm^{-1} = 2969, 2093, 1703, 1455, 1349, 1184, 755. **HRMS (ESI)**: calculated for $C_{17}H_{21}N_4O$ ($[M+H]^+$) 297.1710; found 297.1710.

(±)-Mersicarpine (15):

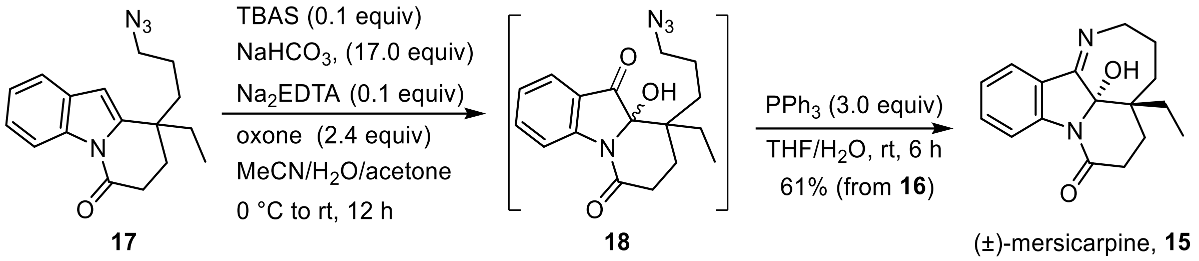

Compound **17** (30 mg, 0.1 mmol, 1.0 equiv) was dissolved in a mixture of acetonitrile (2 mL), water (1 mL) and acetone (2 mL). Tetrabutylammonium hydrogen sulfate (TBAS, 3.0 mg, 0.01 mmol, 0.1 equiv), sodium bicarbonate (142 mg, 1.7 mmol, 17.0 equiv) and disodium ethylenediaminetetraacetic acid (Na_2EDTA, 4.0 mg, 0.01 mmol, 0.1 equiv) were then added to the solution and the resulting mixture was cooled down to 0 ºC. Next, a solution of oxone (148 mg, 0.24 mmol, 2.4 equiv) in water (1 mL) was added dropwise over 5 min with vigorous stirring. The reaction mixture was slowly warmed up to room temperature and stirred for 12 h. After complete consumption of starting material as indicated by TLC, the reaction mixture was diluted with ethyl acetate (15 mL), washed with brine, dried over anhydrous Na_2SO_4, and concentrated under reduced pressure to afford the crude **18** as a brown liquid, which was used directly in the next step without further purification.

To a solution of crude **18** in THF (3.0 mL) and water (0.2 mL), triphenylphosphine (79 mg, 0.3 mmol, 3.0 equiv) was added at room temperature and stirred for 6 h. After complete consumption of intermediate **18** as indicated by TLC, the reaction mixture was concentrated directly under reduced pressure to afford a yellow oil, which was purified by silica gel column chromatography using 25% ethyl acetate in hexane as eluent to afford **(±)-mersicarpine** (17 mg, 61% over two steps) as a white solid.

Comparison of ^{1}H NMR Data of Mersicarpine 15:

Mersicarpine ^{1}H NMR<br>(this work)<br>(600 MHz, base-washed $CDCl_3$)	Mersicarpine ^{1}H NMR<br>Isolated[6a]<br>(400 MHz, $CDCl_3$)	Mersicarpine ^{1}H NMR<br>By Wang *et al.*[6b]<br>(400 MHz, $CDCl_3$)
8.16 (d, *J* = 7.8 Hz, 1H)	8.13 (br d, *J* = 8.0 Hz, 1H)	8.14 (d, *J* = 8.2 Hz, 1H)
7.60 (d, *J* = 7.2 Hz, 1H)	7.66 (br d, *J* = 8.0 Hz, 1H)	7.60 (d, *J* = 7.5 Hz, 1H)
7.38 (t, *J* = 7.5 Hz, 1H)	7.39 (td, *J* = 8.0, 1.0 Hz , 1H)	7.36 (t, *J* = 7.3 Hz, 1H)
7.08 (t, *J* = 6.9 Hz, 1H)	7.09 (td, *J* = 8.0, 1.0 Hz , 1H)	7.07 (t, *J* = 7.5 Hz, 1H)
4.74 (br s, 1H)	5.92 (br s, 1H)	
3.94-3.85 (m, 2H)	3.87 (m, 2H)	3.93-3.78 (m, 2H)
2.60 (dd, *J* = 18.3, 9.3 Hz, 1H)	2.48 (ddd, *J* = 18.5, 9.5, 3.5 Hz, 1H)	2.59 (ddd, *J* = 18.4, 9.6, 3.3 Hz, 1H)
2.44-2.38 (m, 1H)	2.35 (ddd, *J* = 18.5, 8.8, 7.8 Hz, 1H)	2.44-2.35 (m, 1H)
2.07 (t, *J* = 12.9 Hz, 1H)	2.07 (m, 1H)	2.10-2.02 (m, 1H)
1.95-1.90 (m, 1H)	1.89 (dddd, *J* = 14.0, 9.5, 7.8, 1.5 Hz, 1H)	1.96-1.85 (m, 1H)
1.77-1.67 (m, 4H)	1.75 (dt, *J* = 14.3, 3.5 Hz, 1H)	1.77-1.72 (m, 1H)
	1.65 (m, 3H)	1.70-1.62 (m, 3H)
1.37-1.32 (m, 1H)	1.32 (dqd, *J* = 14.5,7.5,1.5 Hz, 1H)	1.34-1.28 (m, 1H)
1.15-1.10 (m, 1H)	1.10 (dq, *J* = 14.5,7.5 Hz, 1H)	1.16-1.07 (m, 1H)
0.75 (t, *J* = 6.6 Hz, 3H)	0.74 (t, *J* = 7.5 Hz, 3H)	0.74 (t, *J* = 7.4 Hz, 3H)

Comparison of ^{13}C NMR Data of Mersicarpine 15:

Mersicarpine ^{13}C NMR (this work) (151 MHz, base-washed $CDCl_3$)	Mersicarpine ^{13}C NMR Isolated[6a] (101 MHz, $CDCl_3$)	Mersicarpine ^{13}C NMR Wang *et al.*[6b] (101 MHz, $CDCl_3$)
169.6	169.6	169.6
168.7	168.9	169.0
146.7	146.5	146.7
133.4	133.2	133.5
124.52	124.4	124.5
124.50	124.2	124.4
122.3	122.2	122.4
117.0	116.7	116.9
93.9	93.8	94.0
50.8	50.5	50.6
39.4	39.3	39.4
34.5	34.3	34.5
29.3	29.1	29.8, 29.3
25.6	25.4	25.6
23.1	22.9	23.1
21.3	21.1	21.3
7.0	6.8	7.0

Computational Studies:

All the calculations were performed with the Gaussian 16, Revision B.01 program package.[7] Geometry optimization and frequency analysis were conducted using B3LYP function.[8] During geometry optimization we used TZVP basis set for all atoms. In all cases, ultrafine integral grid was employed. To refine the computed energy, single point calculations were performed using the B3LYP functional with the Def2-TZVP basis set for all atoms with considering the Grimme's D3 (BJ-damping) dispersion effect. The SMD solvent model[9] was used to simulate the implicit solvent effect. Here, dichloromethane was utilized as the solvent. Transition state optimizations were performed starting from infinitely separated substrates. Single imaginary frequency for the transition states (TSs) and real frequencies for local minima were obtained. The connectivity of each TS was validated through a relaxed potential energy surface scan for the corresponding reaction coordinate, and was found to be the highest-energy point that connected the relevant reactant and product. The geometries were optimized without any symmetry constraints. The zero-point vibrational energies (ZPVE), thermal corrections were obtained by using the temperature 353.15 K and 1 atm pressure. Unless otherwise specified, the ΔG was used throughout the text. All calculated structures were visualized with ChemCraft.[10]

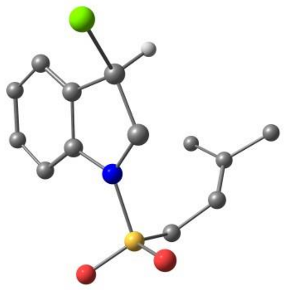

8

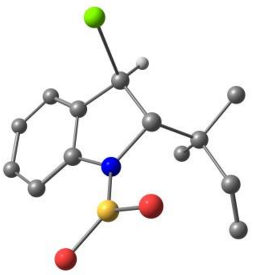

9

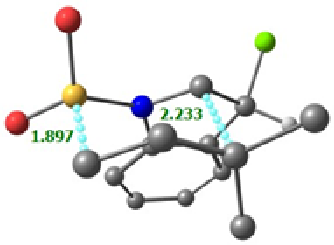

TS1

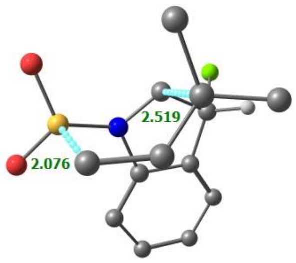

TS2

Figure S1. DFT optimized structures. Hydrogen atoms are removed for clarity in the absence of H-bonds.

Table S1. Electronic and Gibbs free energies (in Hartree) at the at B3LYP(SMD)/Def2-TZVP //B3LYP/TZVP level of theory.

Structures	*E*	*G*	Structures	*E*	*G*
**8**	-1196.045769	-1196.134386	**TS1**	-1196.037060	-1196.118469
**9**	-1196.094196	-1196.176841	**TS2**	-1196.030244	-1196.110678

G = Sum of electronic and thermal free energies.

Table S2. Cartesian coordinates (Å) of the optimized structures of all intermediates and transition states involved in the C2 allylation of 3-chloroindole at B3LYP/TZVP level of theory.

8

C	1.715071000	7.957099000	-3.315844000
C	1.153596000	6.807767000	-3.718009000
C	-0.264001000	6.349812000	-3.605439000
H	1.923334000	4.656028000	-1.297961000
H	1.825261000	6.085502000	-4.180533000
H	-0.623609000	6.051183000	-4.600392000
H	-0.931332000	7.153162000	-3.284995000
C	-0.278094000	7.333582000	1.936194000
C	0.173453000	6.565054000	0.880761000
C	-0.671340000	6.262541000	-0.190327000
C	-1.979633000	6.707566000	-0.248420000
C	-2.429853000	7.482903000	0.821967000
C	-1.597609000	7.791316000	1.896310000
C	1.505574000	5.943942000	0.581075000
C	1.230852000	5.257964000	-0.729406000
N	0.031792000	5.455289000	-1.142684000
H	0.363141000	7.563490000	2.777863000
H	-2.611509000	6.441280000	-1.083488000
H	-3.449441000	7.847321000	0.817093000
H	-1.979117000	8.390306000	2.713500000
B	-0.506482000	5.018973000	-2.679525000
O	0.356073000	3.881793000	-2.950881000
O	-1.911025000	4.718640000	-2.583758000
H	0.437804000	3.720407000	-3.893679000
H	-2.030931000	3.791273000	-2.355286000
C	3.186865000	8.217289000	-3.519607000
H	3.691948000	8.433192000	-2.570781000
H	3.687689000	7.366331000	-3.983662000
H	3.347116000	9.093381000	-4.158115000
C	0.966204000	9.096983000	-2.675630000
H	1.059568000	10.005424000	-3.281130000
H	-0.094268000	8.890401000	-2.545241000
H	1.385450000	9.340935000	-1.692803000
Cl	2.087659000	4.742040000	1.827902000
H	2.313823000	6.668936000	0.483765000

9

C	1.715973000	6.753258000	-2.352635000
C	1.450570000	6.260768000	-3.761227000
C	0.811745000	6.898804000	-4.735044000
H	1.574539000	4.755015000	-1.533095000
H	1.857279000	5.274933000	-3.969790000
H	0.710051000	6.451724000	-5.716777000
H	0.370046000	7.878653000	-4.605179000
C	-0.456094000	7.327006000	1.661702000
C	-0.071992000	6.619558000	0.533098000
C	-1.003378000	6.249242000	-0.443538000
C	-2.348339000	6.584689000	-0.292211000
C	-2.724724000	7.292400000	0.846458000
C	-1.796402000	7.668801000	1.817664000
C	1.271841000	6.125301000	0.130948000
C	1.093325000	5.711847000	-1.345924000
N	-0.373530000	5.556582000	-1.492399000
H	0.274450000	7.595538000	2.415617000
H	-3.075468000	6.301636000	-1.037704000
H	-3.766564000	7.561803000	0.973910000
H	-2.118774000	8.222522000	2.690170000
B	-0.973411000	4.588160000	-2.359240000
O	-0.135246000	3.697087000	-3.000055000
O	-2.348030000	4.575917000	-2.487391000

H	-0.550634000	3.142675000	-3.664724000
H	-2.708688000	3.822304000	-2.960943000
C	3.251602000	6.743447000	-2.166980000
H	3.549013000	7.098031000	-1.178542000
H	3.661323000	5.739611000	-2.300429000
H	3.717460000	7.396967000	-2.906252000
C	1.179488000	8.172921000	-2.130649000
H	1.622839000	8.859608000	-2.854162000
H	0.095791000	8.218618000	-2.239980000
H	1.430174000	8.543196000	-1.135994000
Cl	1.789844000	4.661735000	1.174462000
H	2.080400000	6.826595000	0.293773000

TS1

C	2.133795000	6.126038000	-2.347586000
C	1.398314000	6.068447000	-3.530629000
C	0.198552000	6.752220000	-3.802153000
H	0.788731000	4.013074000	-1.906331000
H	1.640756000	5.243984000	-4.197277000
H	-0.139187000	6.731115000	-4.835019000
H	0.031997000	7.717660000	-3.335245000
C	-0.290991000	6.652314000	1.803526000
C	-0.138115000	6.022233000	0.582356000
C	-0.988557000	6.312599000	-0.491770000
C	-2.012618000	7.241174000	-0.362215000
C	-2.164947000	7.865907000	0.876478000
C	-1.321339000	7.582223000	1.948547000
C	0.863256000	5.017275000	0.116205000
C	0.546848000	4.907258000	-1.356406000
N	-0.619610000	5.534442000	-1.619911000
H	0.362844000	6.417180000	2.634636000
H	-2.654623000	7.468650000	-1.199441000
H	-2.958719000	8.592303000	1.002145000
H	-1.466883000	8.082701000	2.897344000
B	-1.137733000	5.630693000	-3.056229000
O	-1.054577000	4.334961000	-3.659156000
O	-2.340947000	6.374988000	-3.233993000
H	-1.209813000	4.365680000	-4.606649000
H	-3.074576000	5.781417000	-3.422714000
C	3.369924000	5.265709000	-2.223584000
H	3.535797000	4.937884000	-1.193157000
H	3.312206000	4.377155000	-2.854299000
H	4.263202000	5.828125000	-2.516536000
C	2.175143000	7.401752000	-1.532528000
H	1.212125000	7.904040000	-1.472838000
H	2.527787000	7.225105000	-0.515016000
H	2.883766000	8.099443000	-1.992551000
Cl	0.627630000	3.355585000	0.916274000
H	1.896469000	5.253756000	0.347217000

TS2

C	0.371603000	-0.645247000	-1.628667000
B	-1.258764000	0.892617000	-0.483980000
C	0.002593000	2.463042000	-0.985694000
C	1.192264000	1.988343000	-1.561872000
C	1.355390000	1.364200000	-2.785274000
O	-1.772774000	1.213061000	0.780158000
O	-2.233777000	0.844993000	-1.503286000
H	-0.762248000	2.882736000	-1.630350000
H	0.098831000	2.974403000	-0.035296000
H	2.051187000	1.922696000	-0.896147000
H	-1.929343000	0.598274000	-2.377907000
H	-2.688838000	1.494829000	0.698276000
C	2.753231000	-1.699374000	0.983294000
C	1.758891000	-1.270723000	0.121291000
C	0.703082000	-0.477383000	0.580950000
C	0.616796000	-0.089900000	1.910220000
C	1.623575000	-0.525693000	2.771812000
C	2.679245000	-1.317371000	2.322082000
C	1.551078000	-1.512421000	-1.335608000
N	-0.183268000	-0.185580000	-0.500579000
H	3.560632000	-2.328867000	0.629995000
H	-0.202247000	0.523321000	2.253087000
H	1.579680000	-0.240036000	3.815612000
H	3.443560000	-1.640759000	3.017282000
H	0.712999000	2.211664000	-4.648472000
H	0.206816000	0.549333000	-4.435468000
H	-0.620414000	1.853334000	-3.563184000
C	0.350517000	1.494885000	-3.902441000
C	2.726582000	0.935215000	-3.235771000
H	3.425589000	0.857282000	-2.401670000
H	2.704184000	-0.026443000	-3.760125000
H	3.141224000	1.657875000	-3.948771000
H	-0.207821000	-0.741324000	-2.531980000
Cl	1.114775000	-3.303088000	-1.697286000
H	2.413029000	-1.351091000	-1.972816000

References:

1. (a) T. Guney, J. J. Lee, G. A. Kraus, *Org. Lett.* **2014**, *16*, 1124-1127; (b) J. Yana, T. Nib, F. Yan, *Tetrahedron Letters* **2015**, *56*, 1096-1098.

2. (a) M. Bernasconi, V. Ramella, P. Tosatti, A. Pfaltz, *Chem. Eur. J.* **2014**, *20*, 2440-2444; (b) Q. Tan, X. Wang, Y. Xiong, Z. Zhao, L. Li, P. Tang, M. Zhang, *Angew. Chem. Int. Ed.* **2017**, *56*, 4829-4833; (c) J. Qi, J. Zheng, S. Cui, *Org. Lett.* **2018**, *20*, 1355-1358.

3. (a) M. Raducan, R. Alam, K. J. Szabó, *Angew. Chem. Int. Ed.* **2012**, *51*, 13050-13053; (b) S. Sahu, G. Karan, L. Roy, M. S. Maji, *Chem. Sci.*, **2022**, *13*, 2355-2362.

4. (a) P. Huang, X. Peng, D. Hu, H. Liao, S. Tang, L. Liu, *Org. Biomol. Chem.* **2017**,*15*, 9622-9629; (b) P. Katrun, S. Hongthong, S. Hlekhlai, M. Pohmakotr, V. Reutrakul, D. Soorukram, T. Jaipetch, C. Kuhakarn, *RSC Adv.* **2014**, *4*, 18933-18938; (c) K. A. Miller, T. R. Welch, T. J. Greshock, Y. Ding, D. H. Sherman, R. M. Williams, *J. Org. Chem.* **2008**, *73*, 3116-3119.

5. (a) K. Rode, P. R. Narasimhamurthy, R. Rieger, F. Krätzschmar, A. Breder, *Eur. J. Org. Chem.* **2021**, 1720-1725; (b) K. Komine, K. M. Lambert, Q. R. Savage, J. B. Cox, J. L. Wood, *Chem. Sci.* **2020**, *11*, 9488-9493.

6. (a) T.-S. Kam, G. Subramaniam, K.-H. Lim, Y.-M. Choo, *Tetrahedron Letters* 2004, 45, 5995-5998; (b) Y. Liu, H. Wang, *Chem. Commun.* **2019**, *55*, 3544-3547.

7. (a) M. J. Frisch, G. W. Trucks, H. B. Schlegel, G. E. Scuseria, M. A. Robb, J. R. Cheeseman, G. Scalmani, V. Barone, G. A. Petersson, H. Nakatsuji, X. Li, M. Caricato, A. V. Marenich, J. Bloino, B. G. Janesko, R. Gomperts, B. Mennucci, H. P. Hratchian, J. V. Ortiz, A. F. Izmaylov, J. L. Sonnenberg, D. Williams-Young, F. Ding, F. Lipparini, F. Egidi, J. Goings, B. Peng, A. Petrone, T. Henderson, D. Ranasinghe, V. G. Zakrzewski, J. Gao, N. Rega, G. Zheng, W. Liang, M. Hada, M. Ehara, K. Toyota, R. Fukuda, J. Hasegawa, M. Ishida, T. Nakajima, Y. Honda, O. Kitao, H. Nakai, T. Vreven, K. Throssell, J. A. Jr. Montgomery, J. E. Peralta, F. Ogliaro, M. J. Bearpark, J. J. Heyd, E. N. Brothers, K. N. Kudin, V. N. Staroverov, T. A. Keith, R. Kobayashi, J. Normand, K. Raghavachari, A. P. Rendell, J. C. Burant, S. S. Iyengar, J. Tomasi, M. Cossi, J. M. Millam, M. Klene, C. Adamo, R. Cammi, J. W. Ochterski, R. L. Martin, K. Morokuma, O. Farkas, J. B. Foresman, D. J. Fox, *Gaussian 16* (Revision B.01): Gaussian, Inc.: Wallingford CT (**2016**).

8. (a) A. D. Becke, *J. Chem. Phys.* **1993**, *98*, 1372-1377; (b) C. Lee, W. Yang, R. G. Parr, *Phys. Rev. B* **1988**, *37*, 785-789; (c) A. D. Becke, *Phys. Rev. A* **1988**, *38*, 3098-3100.

9. A. V. Marenich, C. J. Cramer, D. G. Truhlar, *J. Phys. Chem. B* **2009**, *113*, 6378-6396.

10. D. A. Zhurko, G. A. Zhurko, *ChemCraft 1.5*; Plimus: San Diego, CA. Available at http://www.chemcraftprog.com.

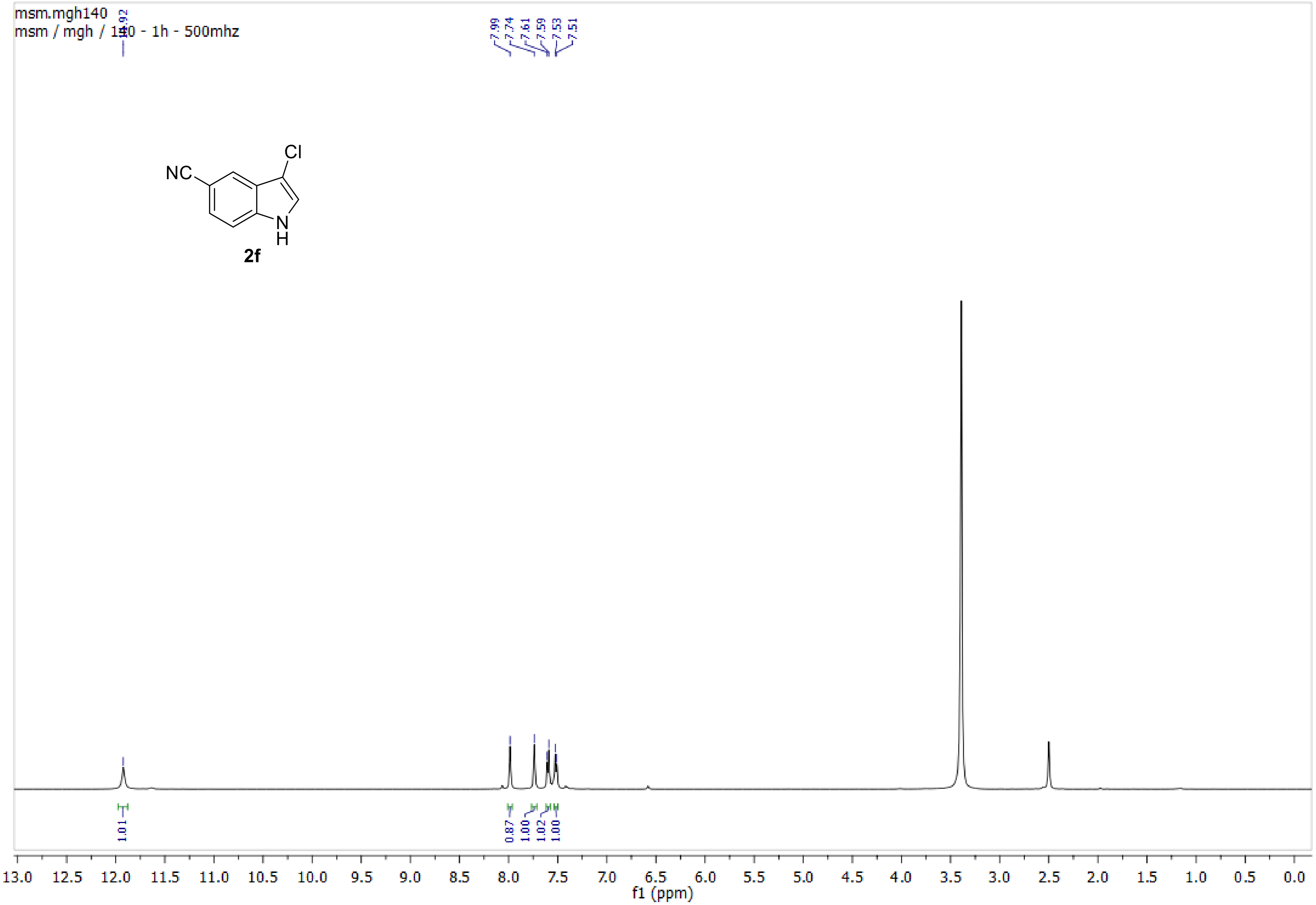
msm.mgh140
msm / mgh / 140 - 1h - 500mhz
11.92
7.99
7.74
7.61
7.59
7.53
7.51
NC
Cl
N
H
2f
1.01
0.87
1.00
1.02
1.00
13.0
12.5
12.0
11.5
11.0
10.5
10.0
9.5
9.0
8.5
8.0
7.5
7.0
6.5
6.0
5.5
5.0
4.5
4.0
3.5
3.0
2.5
2.0
1.5
1.0
0.5
0.0
f1 (ppm)

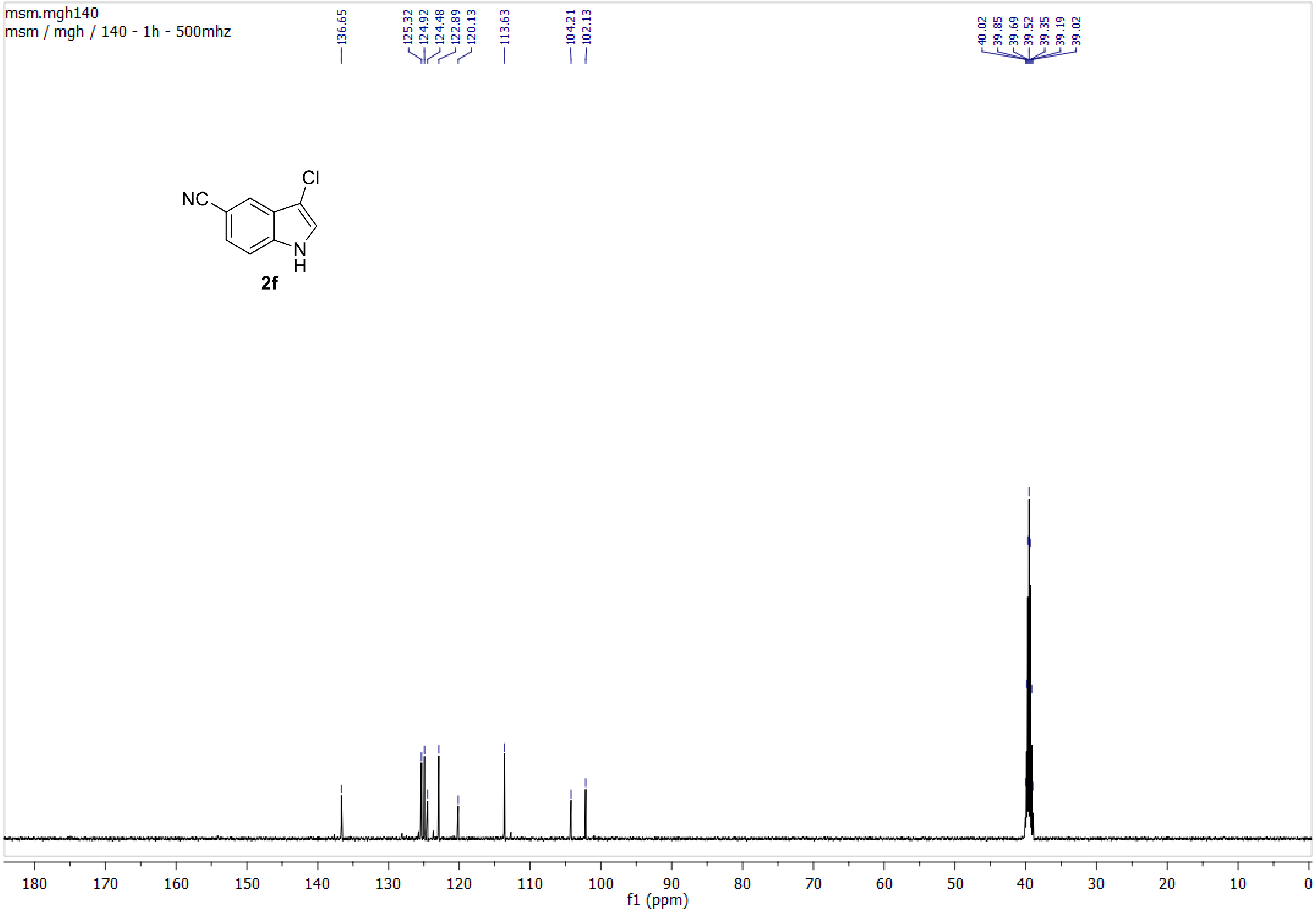
msm.mgh140
msm / mgh / 140 - 1h - 500mhz
136.65
125.32
124.92
124.48
122.89
120.13
113.63
104.21
102.13
40.02
39.85
39.69
39.52
39.35
39.19
39.02
NC
Cl
N
H
2f
180
170
160
150
140
130
120
110
100
90
80
70
60
50
40
30
20
10
0
f1 (ppm)

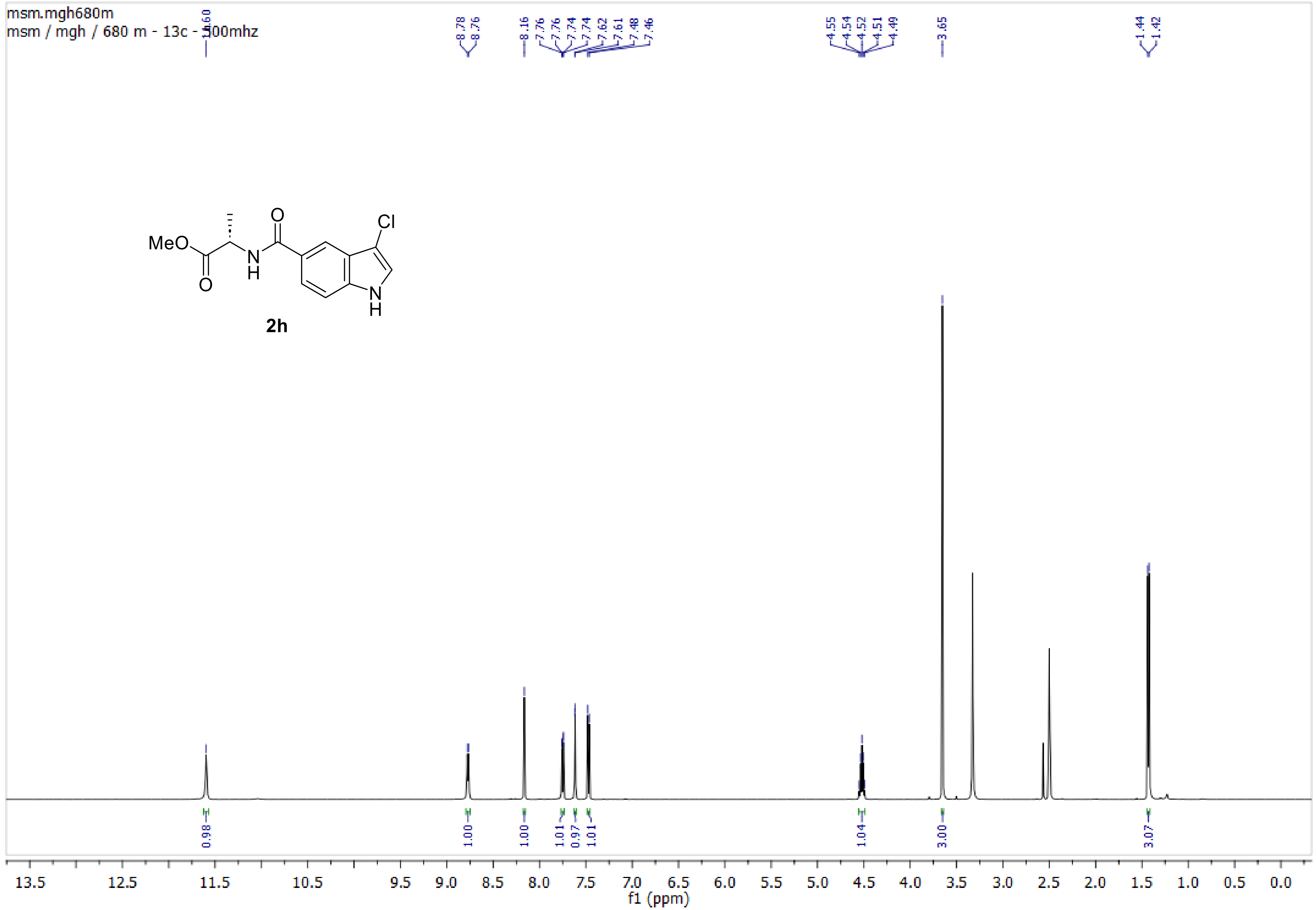
msm.mgh680m
msm / mgh / 680 m - 13c - 500mhz
11.60
8.78
8.76
8.16
7.76
7.76
7.74
7.74
7.62
7.61
7.48
7.46
4.55
4.54
4.52
4.51
4.49
3.65
1.44
1.42
MeO
O
N
H
O
Cl
N
H
2h
0.98
1.00
1.00
1.01
0.97
1.01
1.04
3.00
3.07
f1 (ppm)

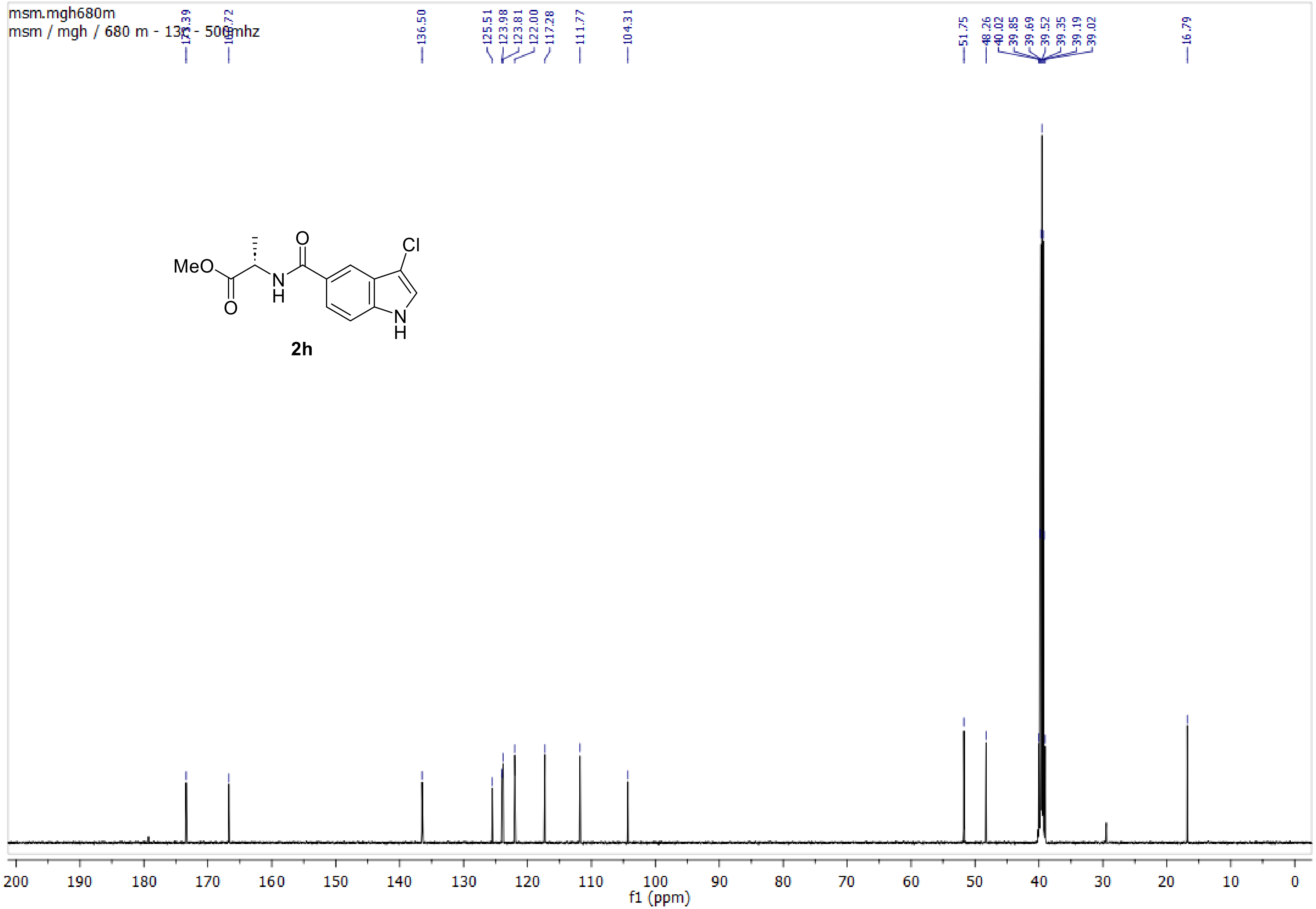
msm.mgh680m
msm / mgh / 680 m - 13c - 500mhz
173.39
166.72
136.50
125.51
123.98
123.81
122.00
117.28
111.77
104.31
51.75
48.26
40.02
39.85
39.69
39.52
39.35
39.19
39.02
16.79
MeO
O
N
H
O
Cl
N
H
2h
200 190 180 170 160 150 140 130 120 110 100 90 80 70 60 50 40 30 20 10 0
f1 (ppm)

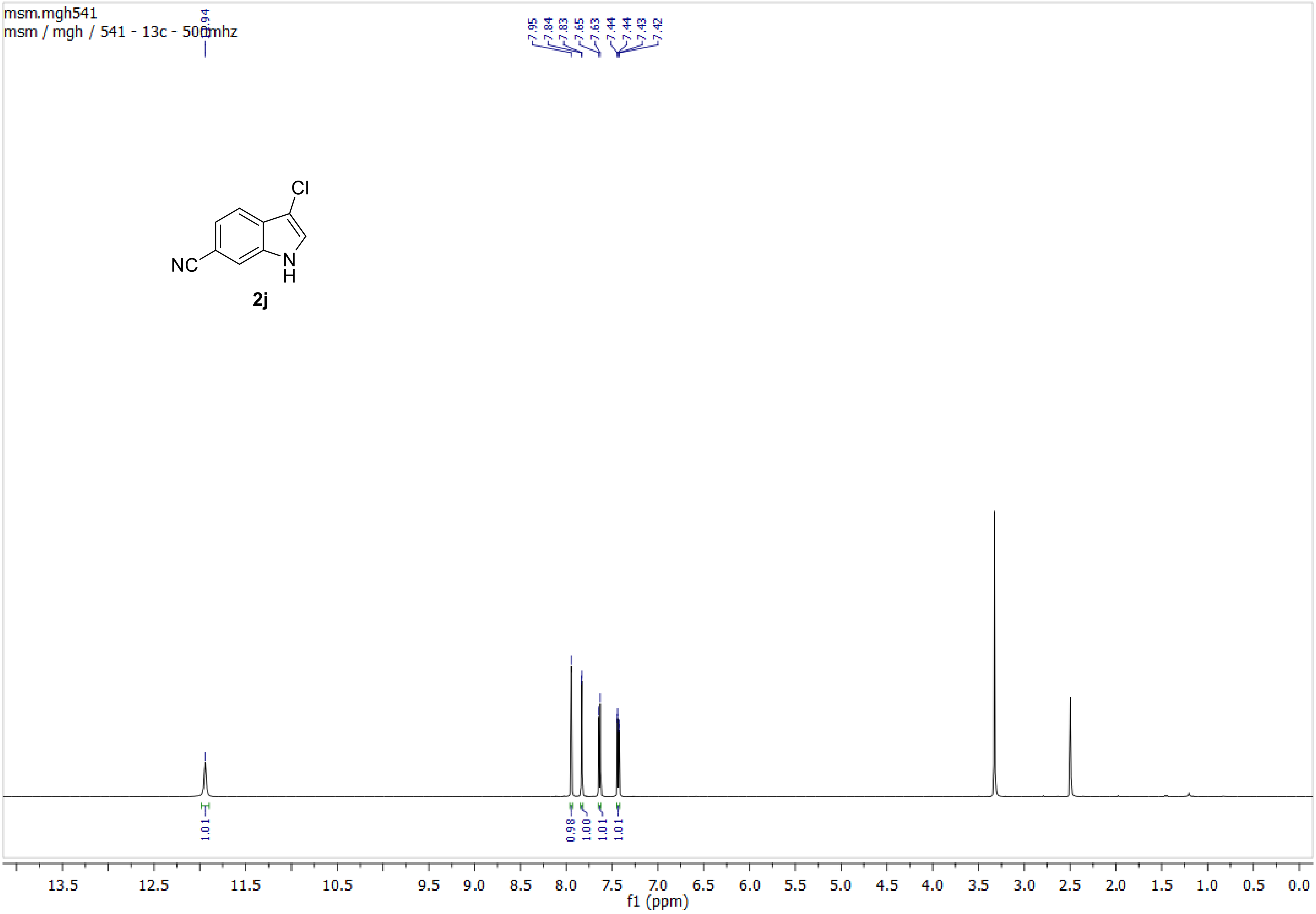
msm.mgh541
msm / mgh / 541 - 13c - 500mhz
11.94
7.95
7.84
7.83
7.65
7.63
7.44
7.44
7.43
7.42
Cl
NC
N
H
2j
1.01
0.98
1.00
1.01
1.01
13.5
12.5
11.5
10.5
9.5
9.0
8.5
8.0
7.5
7.0
6.5
6.0
5.5
5.0
4.5
4.0
3.5
3.0
2.5
2.0
1.5
1.0
0.5
0.0
f1 (ppm)

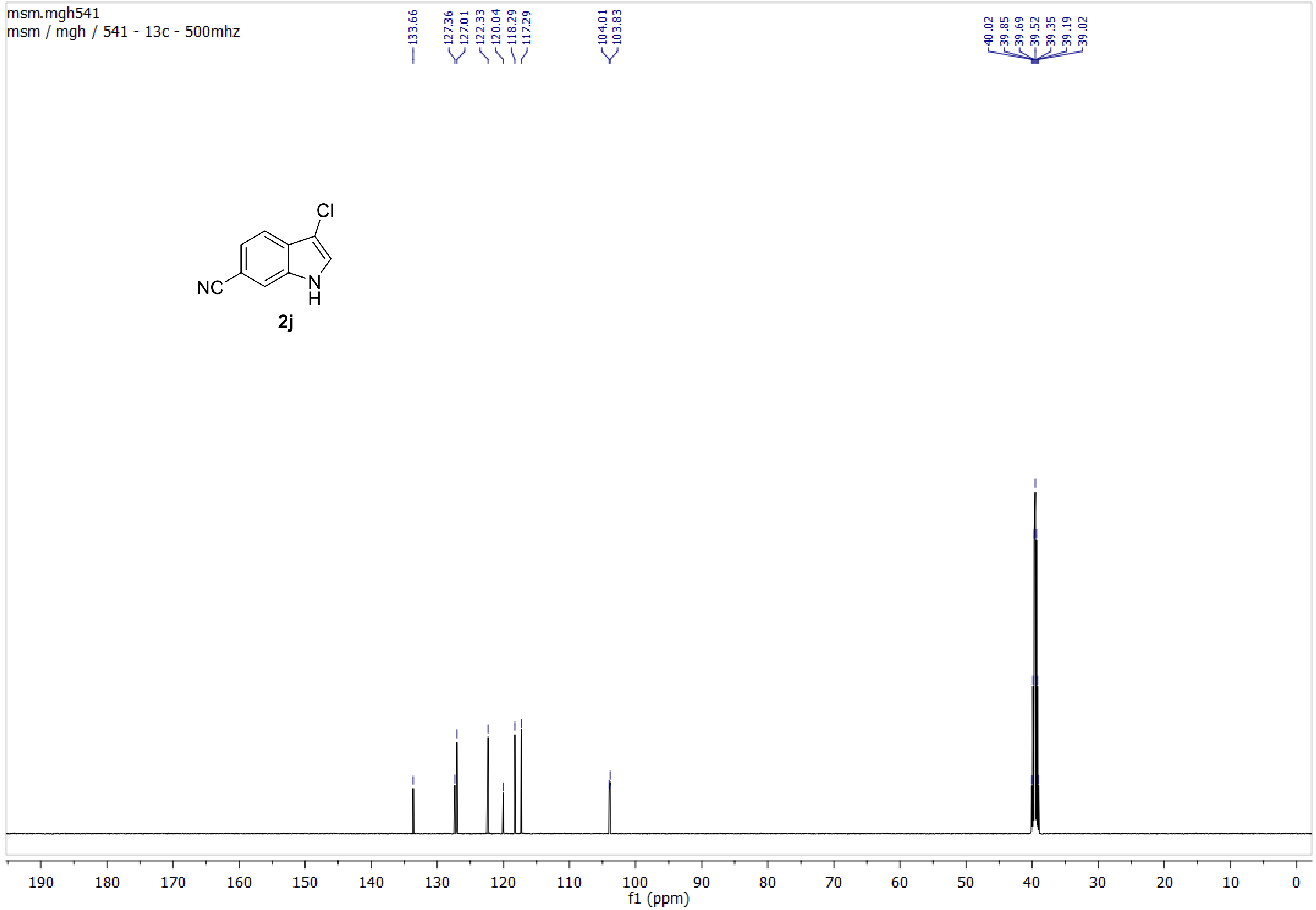
msm.mgh541
msm / mgh / 541 - 13c - 500mhz
133.66
127.36
127.01
122.33
120.04
118.29
117.29
104.01
103.83
40.02
39.85
39.69
39.52
39.35
39.19
39.02
Cl
NC
N
H
2j
190 180 170 160 150 140 130 120 110 100 90 80 70 60 50 40 30 20 10 0
f1 (ppm)

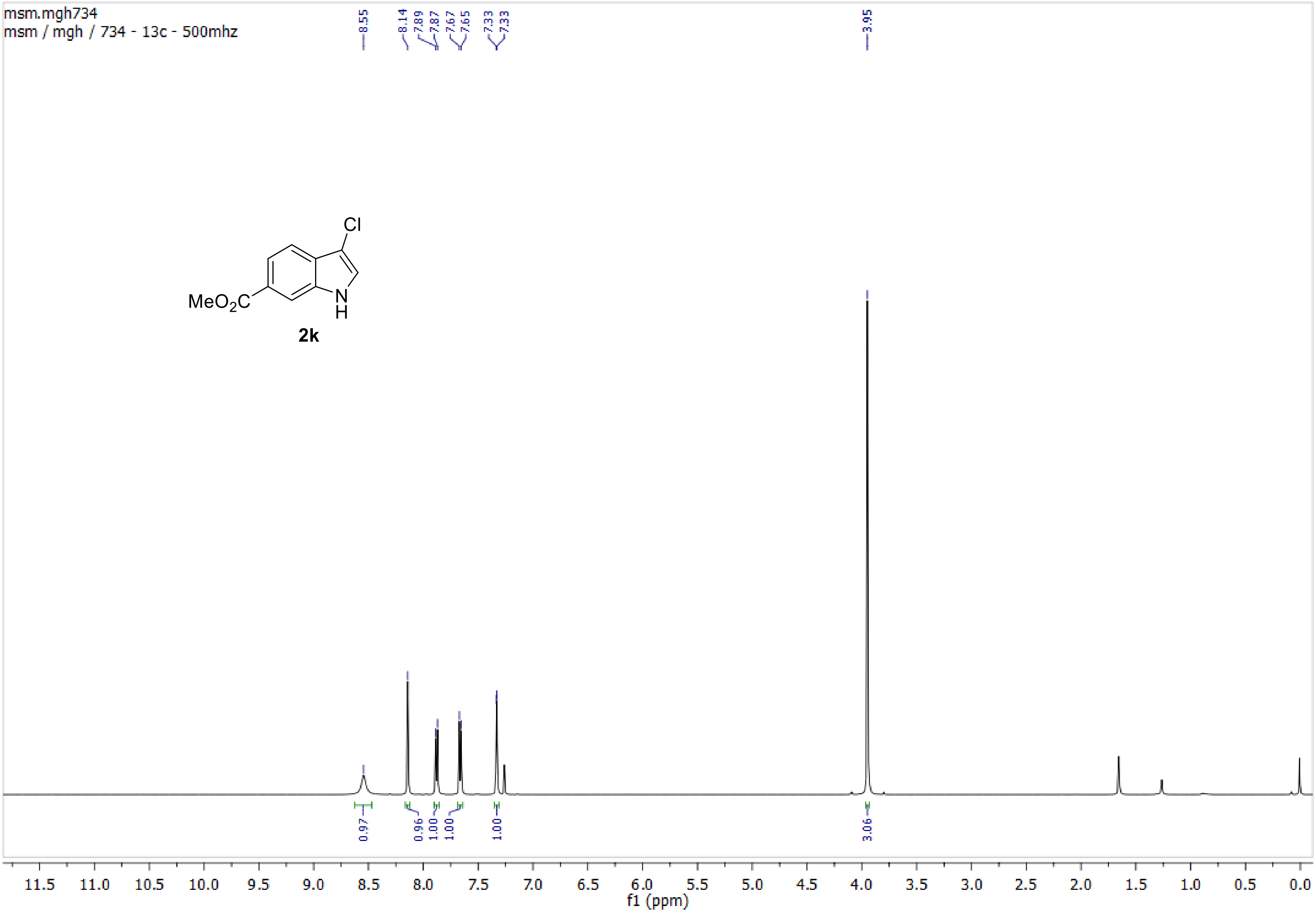
msm.mgh734
msm / mgh / 734 - 13c - 500mhz
8.55
8.14
7.89
7.87
7.67
7.65
7.33
7.33
3.95
Cl
MeO2C
N
H
2k
0.97
0.96
1.00
1.00
1.00
3.06
11.5 11.0 10.5 10.0 9.5 9.0 8.5 8.0 7.5 7.0 6.5 6.0 5.5 5.0 4.5 4.0 3.5 3.0 2.5 2.0 1.5 1.0 0.5 0.0
f1 (ppm)

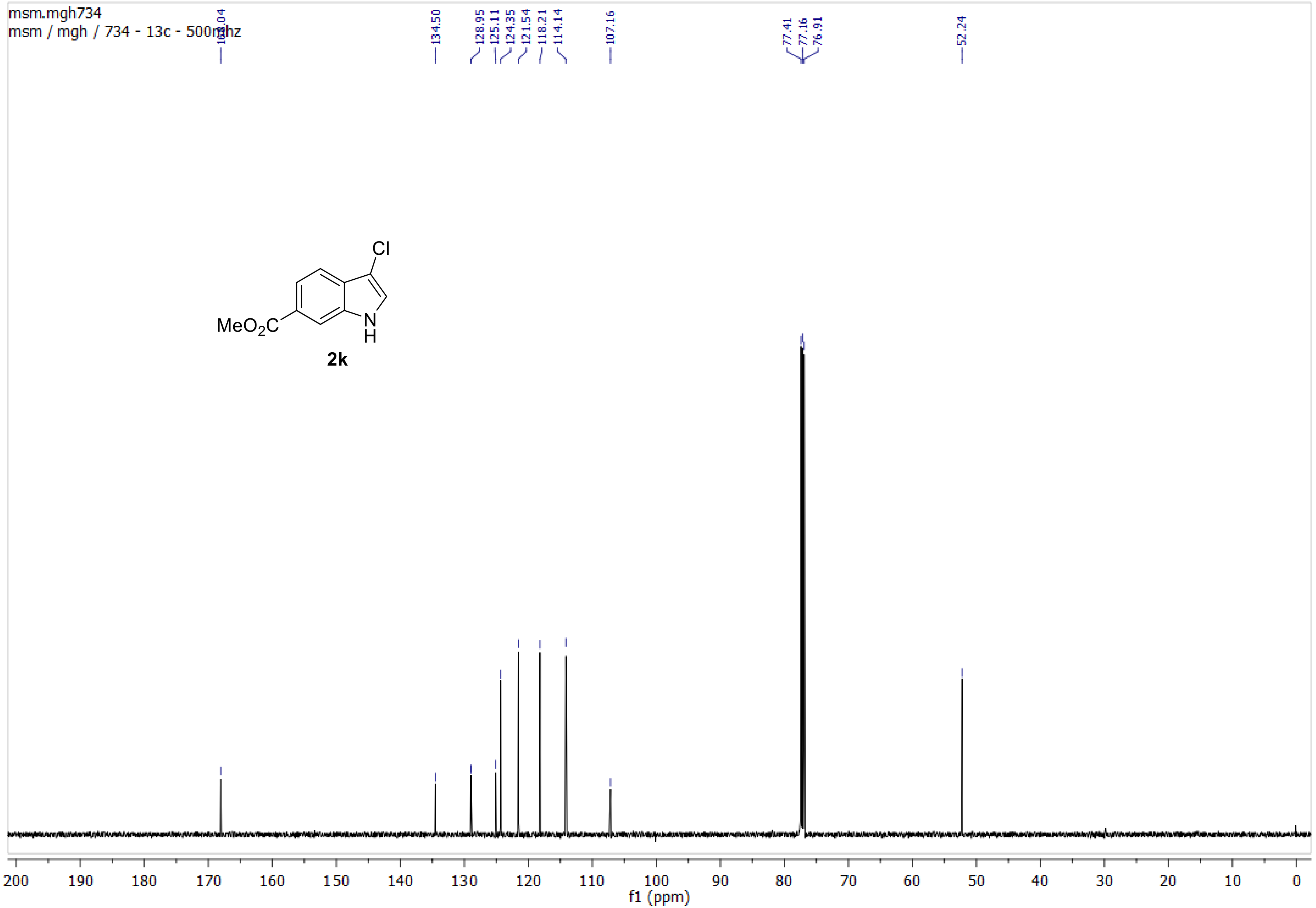
msm.mgh734
msm / mgh / 734 - 13c - 500mhz
134.50
128.95
125.11
124.35
121.54
118.21
114.14
107.16
77.41
77.16
76.91
52.24
Cl
MeO2C
N
H
2k
200 190 180 170 160 150 140 130 120 110 100 90 80 70 60 50 40 30 20 10 0
f1 (ppm)

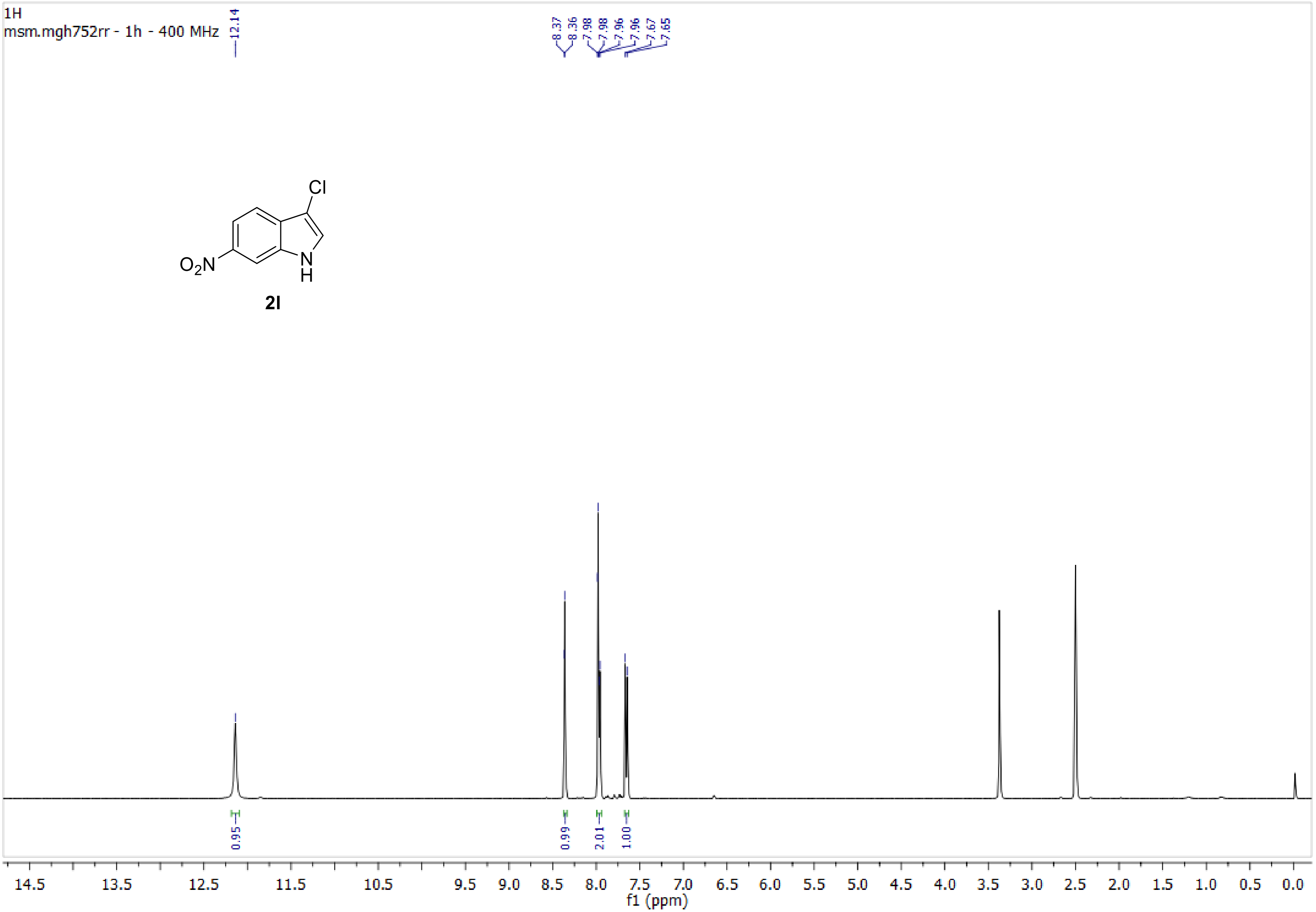
1H
msm.mgh752rr - 1h - 400 MHz
12.14
8.37
8.36
7.98
7.98
7.96
7.96
7.67
7.65
Cl
O_2N
N
H
2l
0.95
0.99
2.01
1.00
14.5 13.5 12.5 11.5 10.5 9.5 9.0 8.5 8.0 7.5 7.0 6.5 6.0 5.5 5.0 4.5 4.0 3.5 3.0 2.5 2.0 1.5 1.0 0.5 0.0
f1 (ppm)

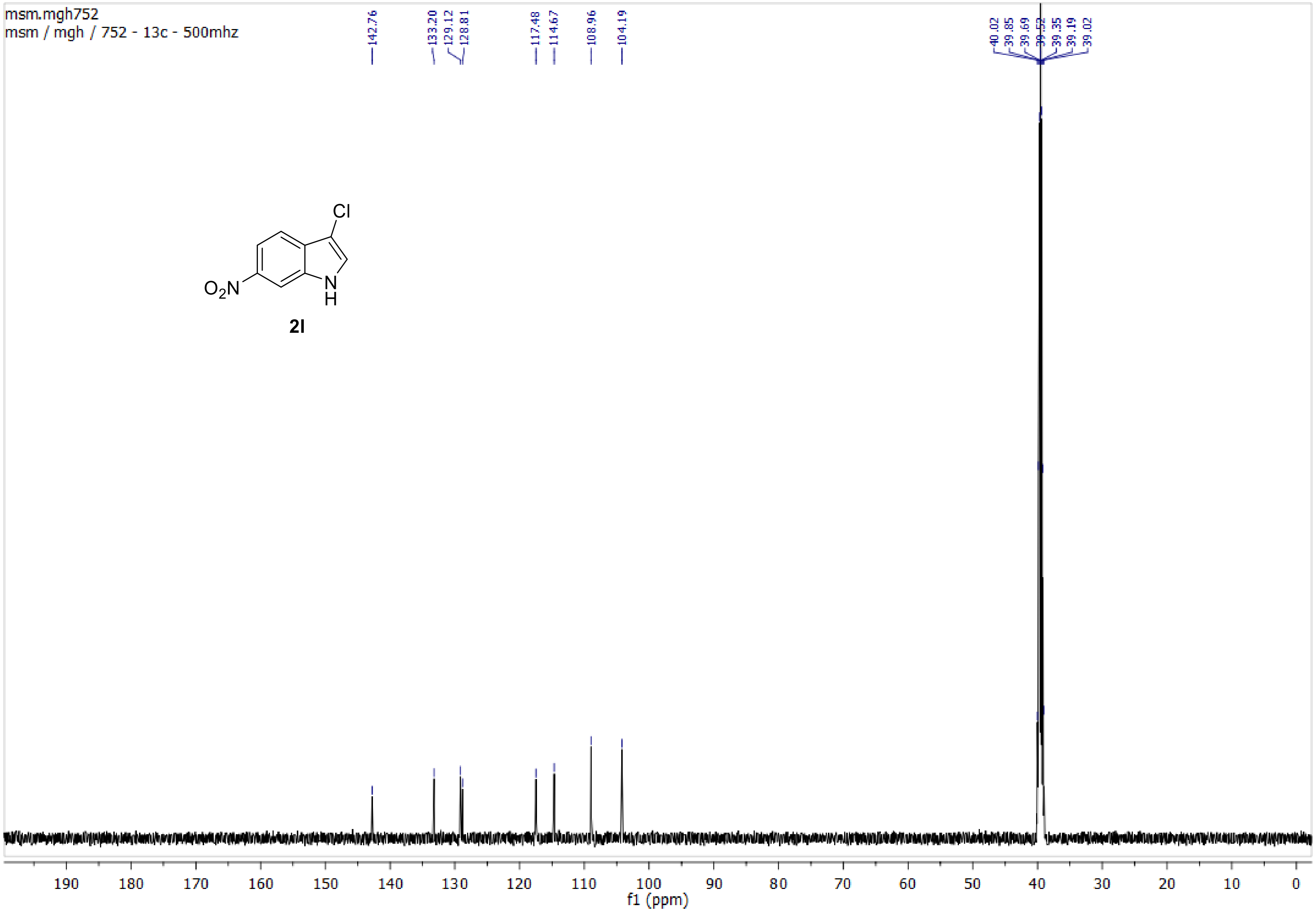
msm.mgh752
msm / mgh / 752 - 13c - 500mhz
142.76
133.20
129.12
128.81
117.48
114.67
108.96
104.19
40.02
39.85
39.69
39.52
39.35
39.19
39.02
Cl
O2N
N
H
2l
190
180
170
160
150
140
130
120
110
100
90
80
70
60
50
40
30
20
10
0
f1 (ppm)

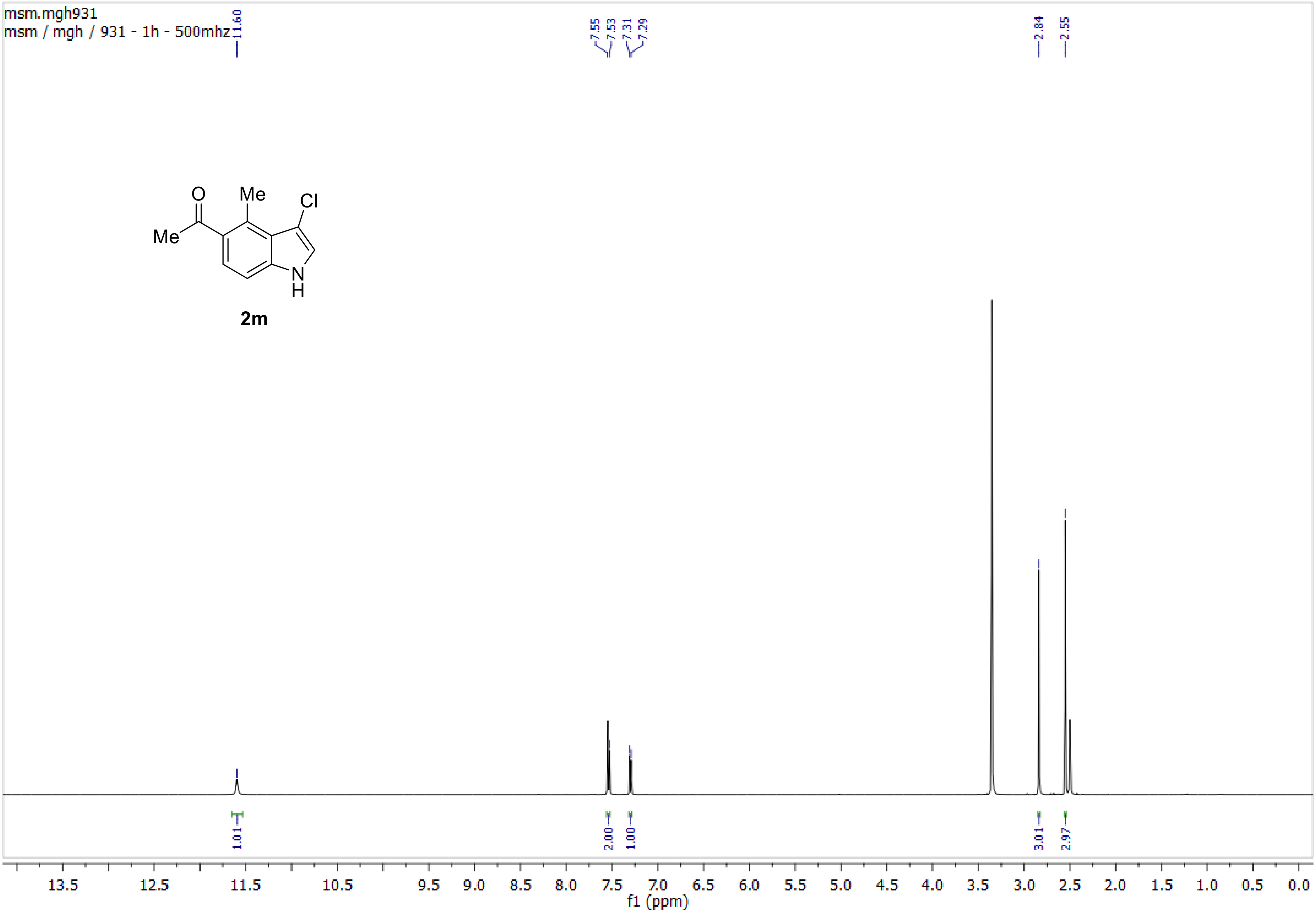
msm.mgh931
msm / mgh / 931 - 1h - 500mhz
11.60
7.55
7.53
7.31
7.29
2.84
2.55
O
Me
Cl
Me
N
H
2m
1.01
2.00
1.00
3.01
2.97
13.5
12.5
11.5
10.5
9.5
9.0
8.5
8.0
7.5
7.0
6.5
6.0
5.5
5.0
4.5
4.0
3.5
3.0
2.5
2.0
1.5
1.0
0.5
0.0
f1 (ppm)

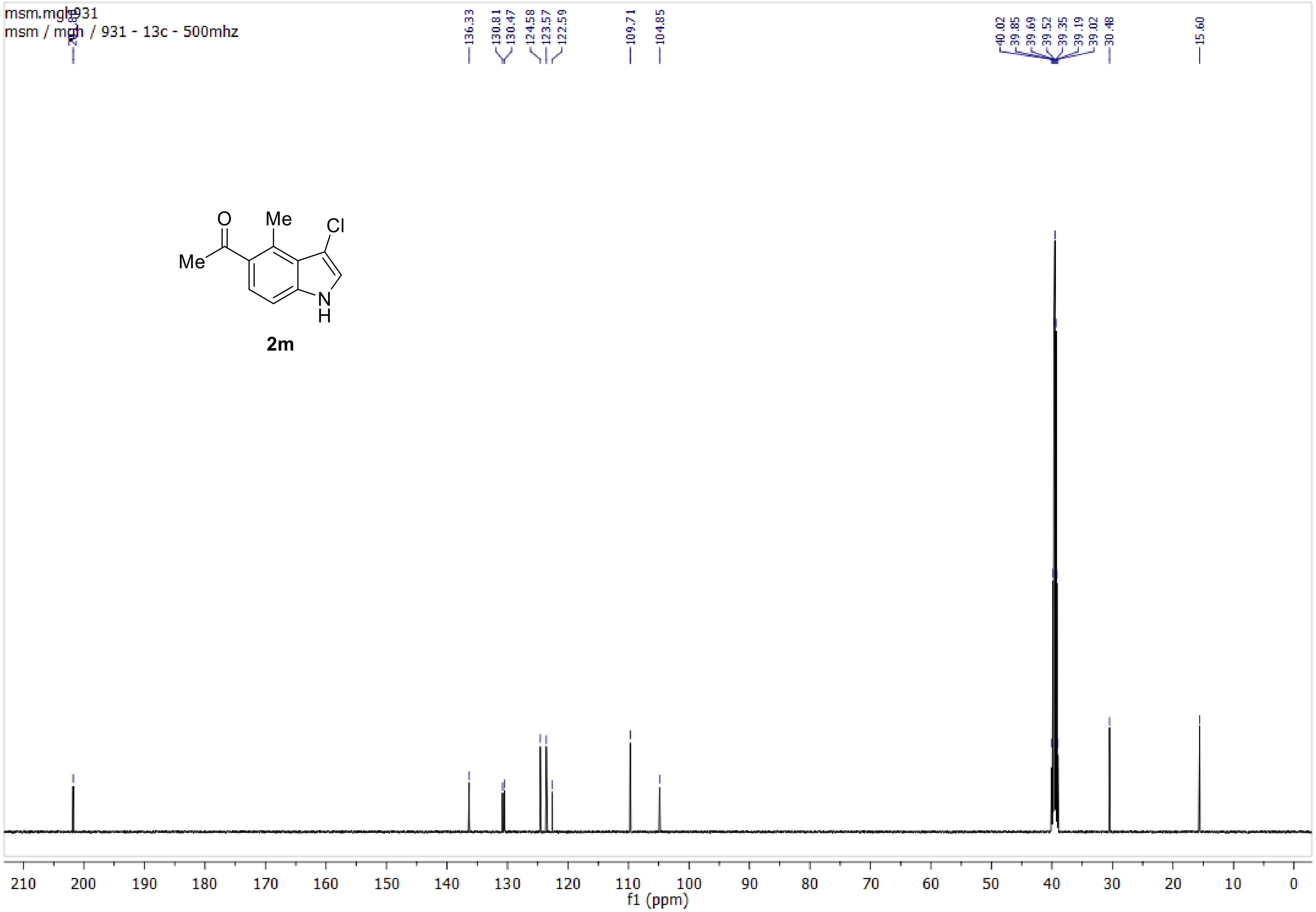
msm.mgh931
msm / mgh / 931 - 13c - 500mhz
136.33
130.81
130.47
124.58
123.57
122.59
109.71
104.85
40.02
39.85
39.69
39.52
39.35
39.19
39.02
30.48
15.60
O
Me
Cl
Me
N
H
2m
210 200 190 180 170 160 150 140 130 120 110 100 90 80 70 60 50 40 30 20 10 0
f1 (ppm)

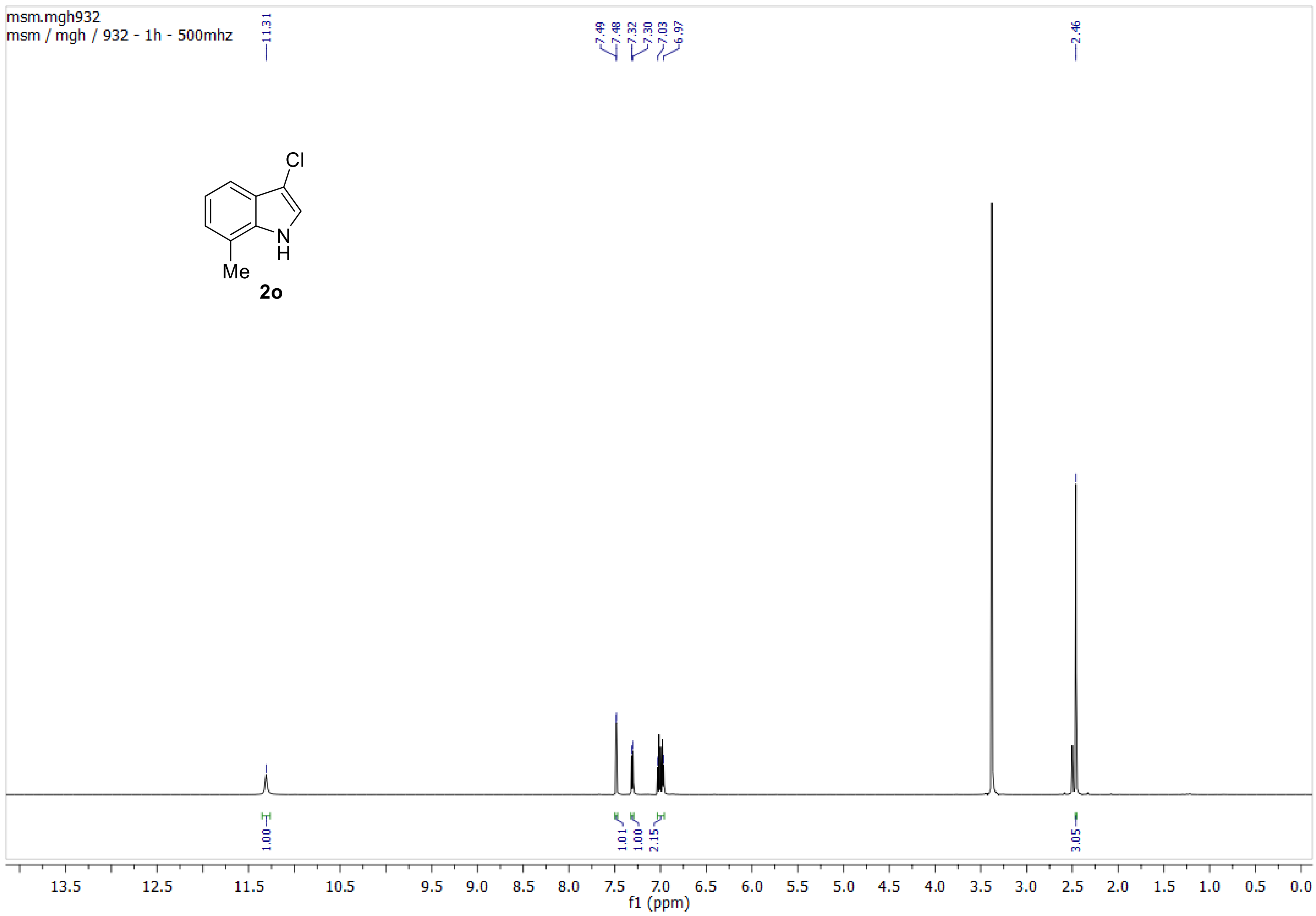
msm.mgh932
msm / mgh / 932 - 1h - 500mhz
11.31
7.49
7.48
7.32
7.30
7.03
6.97
2.46
Cl
N
H
Me
2o
1.00
1.01
1.00
2.15
3.05
13.5
12.5
11.5
10.5
9.5
9.0
8.5
8.0
7.5
7.0
6.5
6.0
5.5
5.0
4.5
4.0
3.5
3.0
2.5
2.0
1.5
1.0
0.5
0.0
f1 (ppm)

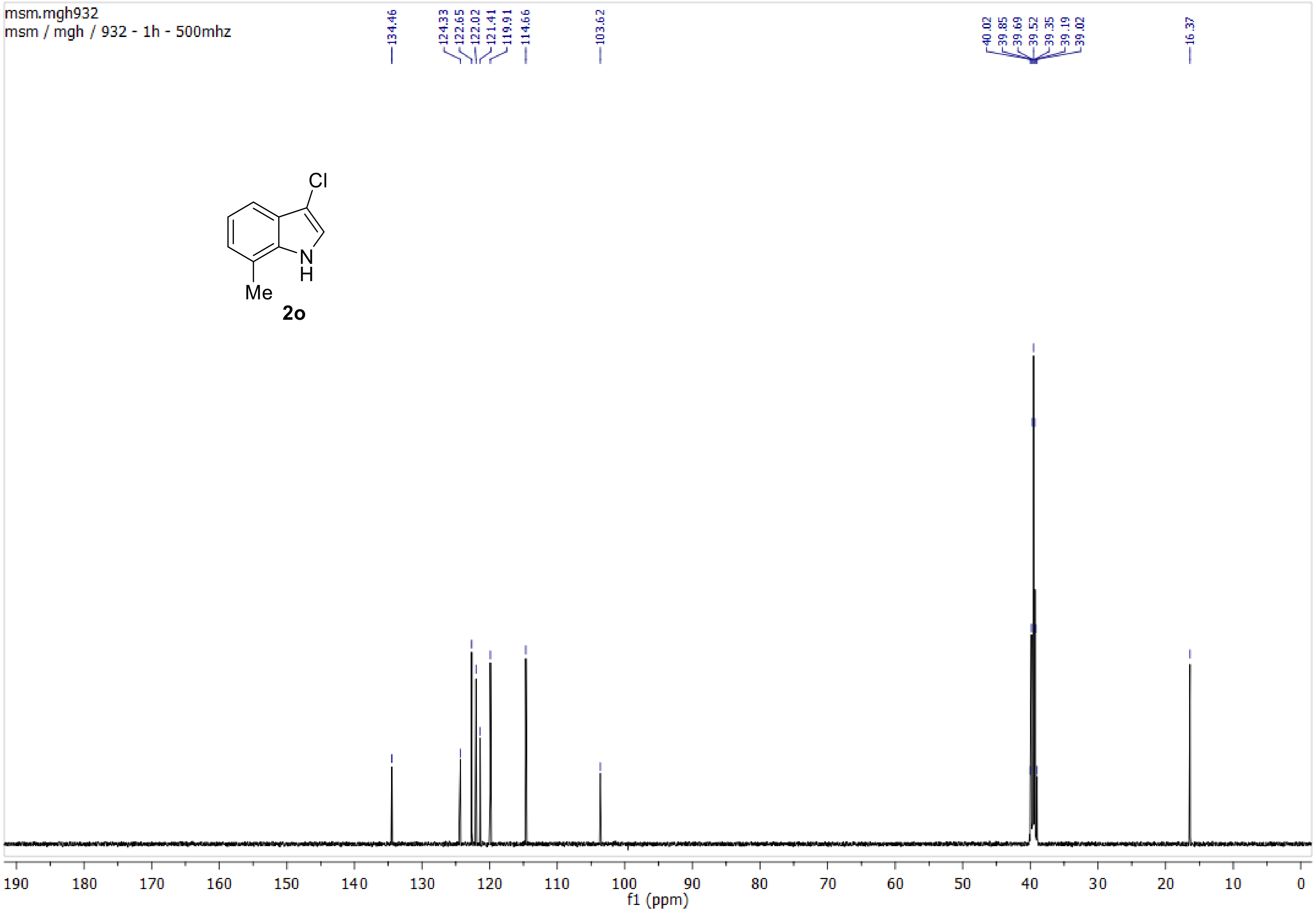
msm.mgh932
msm / mgh / 932 - 1h - 500mhz
134.46
124.33
122.65
122.02
121.41
119.91
114.66
103.62
40.02
39.85
39.69
39.52
39.35
39.19
39.02
16.37
Cl
N
H
Me
2o
190 180 170 160 150 140 130 120 110 100 90 80 70 60 50 40 30 20 10 0
f1 (ppm)

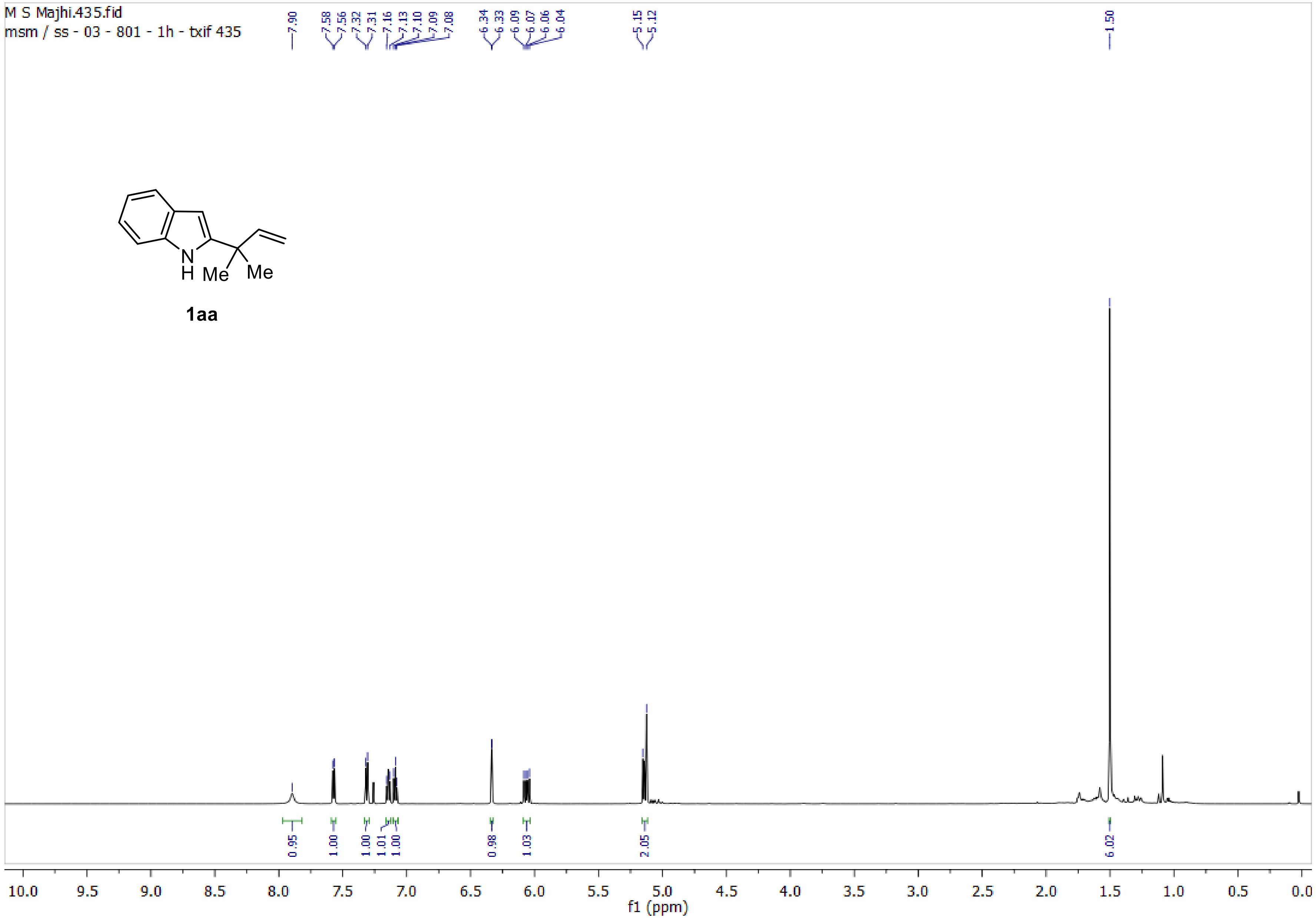
M S Majhi.435.fid
msm / ss - 03 - 801 - 1h - txif 435
7.90
7.58
7.56
7.32
7.31
7.16
7.13
7.10
7.09
7.08
6.34
6.33
6.09
6.07
6.06
6.04
5.15
5.12
1.50
N
H
Me
Me
1aa
0.95
1.00
1.00
1.01
1.00
0.98
1.03
2.05
6.02
10.0
9.5
9.0
8.5
8.0
7.5
7.0
6.5
6.0
5.5
5.0
4.5
4.0
3.5
3.0
2.5
2.0
1.5
1.0
0.5
0.0
f1 (ppm)

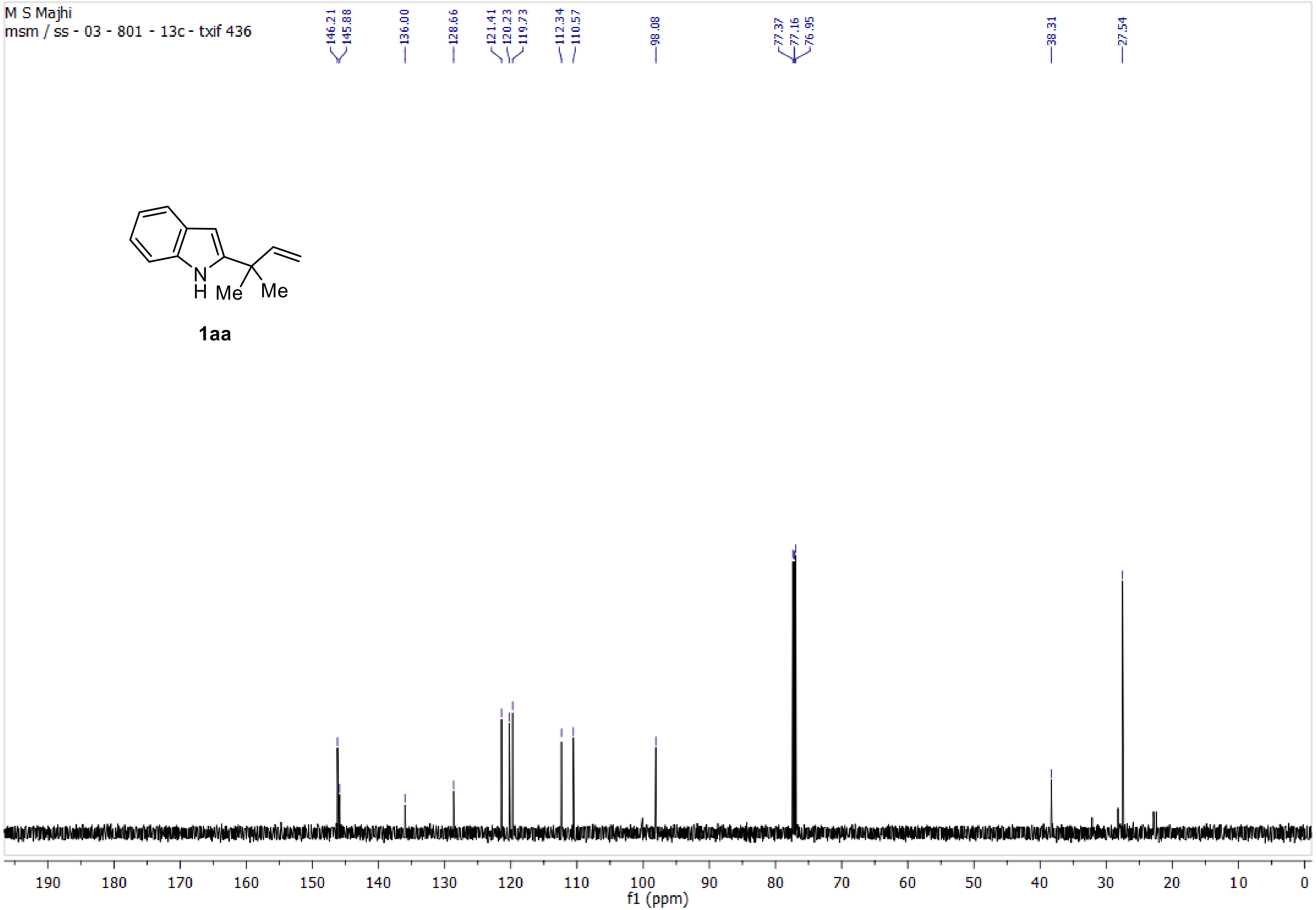
M S Majhi
msm / ss - 03 - 801 - 13c - txif 436
146.21
145.88
136.00
128.66
121.41
120.23
119.73
112.34
110.57
98.08
77.37
77.16
76.95
38.31
27.54
N
H
Me
Me
1aa
190
180
170
160
150
140
130
120
110
100
90
80
70
60
50
40
30
20
10
0
f1 (ppm)

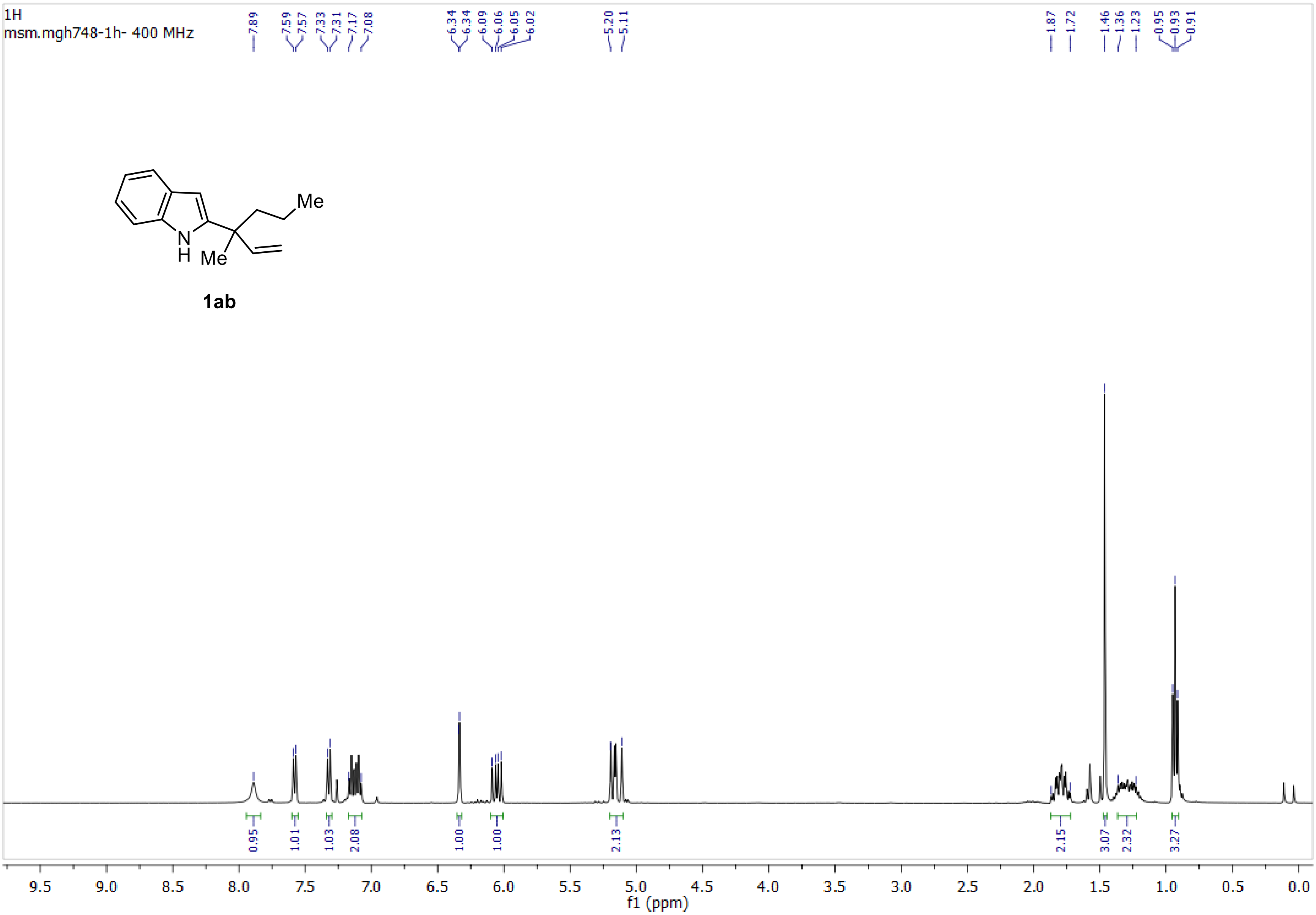
1H
msm.mgh748-1h- 400 MHz
7.89
7.59
7.57
7.33
7.31
7.17
7.08
6.34
6.34
6.09
6.06
6.05
6.02
5.20
5.11
1.87
1.72
1.46
1.36
1.23
0.95
0.93
0.91
Me
N
H
Me
1ab
0.95
1.01
1.03
2.08
1.00
1.00
2.13
2.15
3.07
2.32
3.27
9.5
9.0
8.5
8.0
7.5
7.0
6.5
6.0
5.5
5.0
4.5
4.0
3.5
3.0
2.5
2.0
1.5
1.0
0.5
0.0
f1 (ppm)

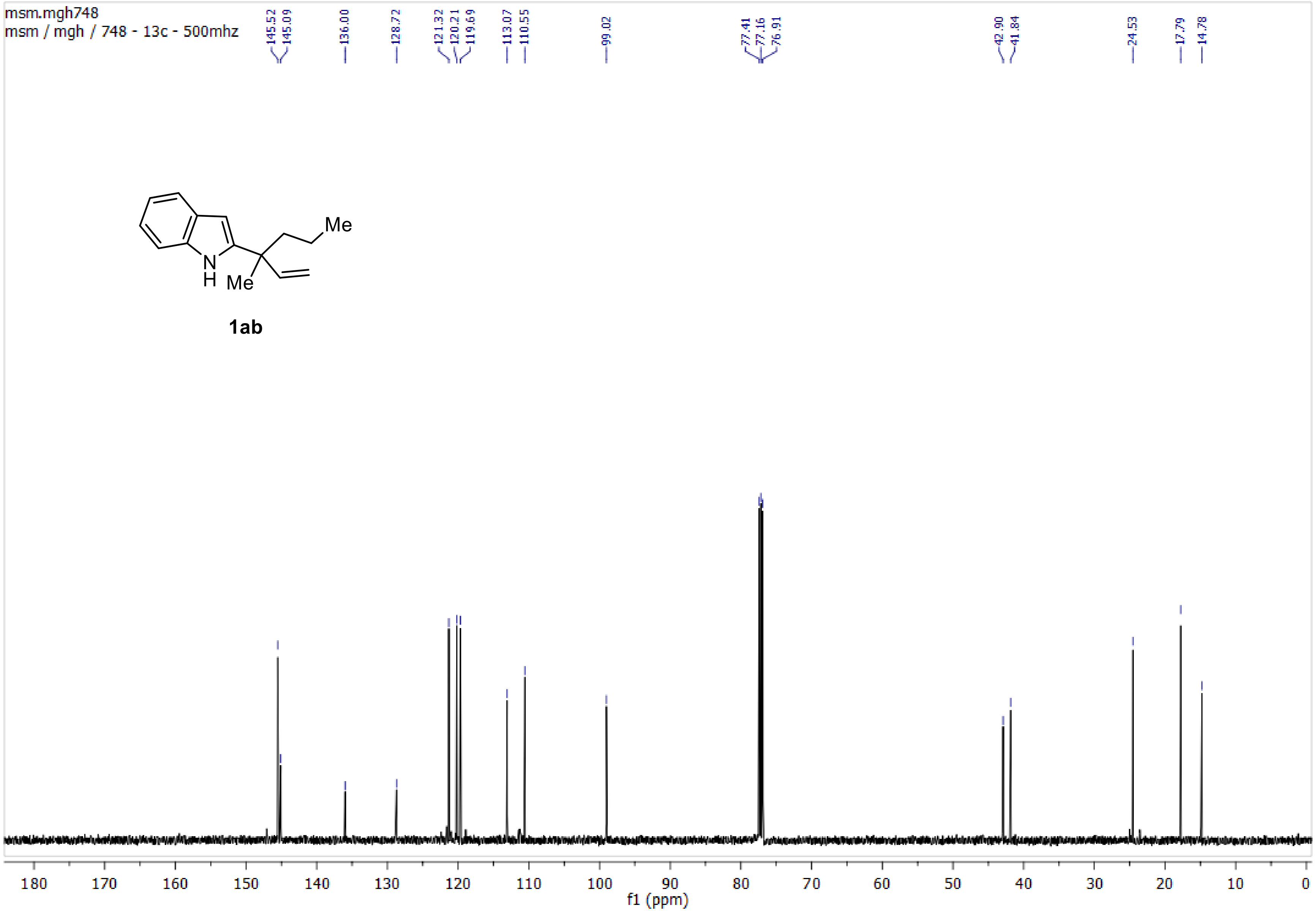

msm.mgh748
msm / mgh / 748 - 13c - 500mhz
145.52
145.09
136.00
128.72
121.32
120.21
119.69
113.07
110.55
99.02
77.41
77.16
76.91
42.90
41.84
24.53
17.79
14.78
Me
N
H
Me
1ab
180
170
160
150
140
130
120
110
100
90
80
70
60
50
40
30
20
10
0
f1 (ppm)

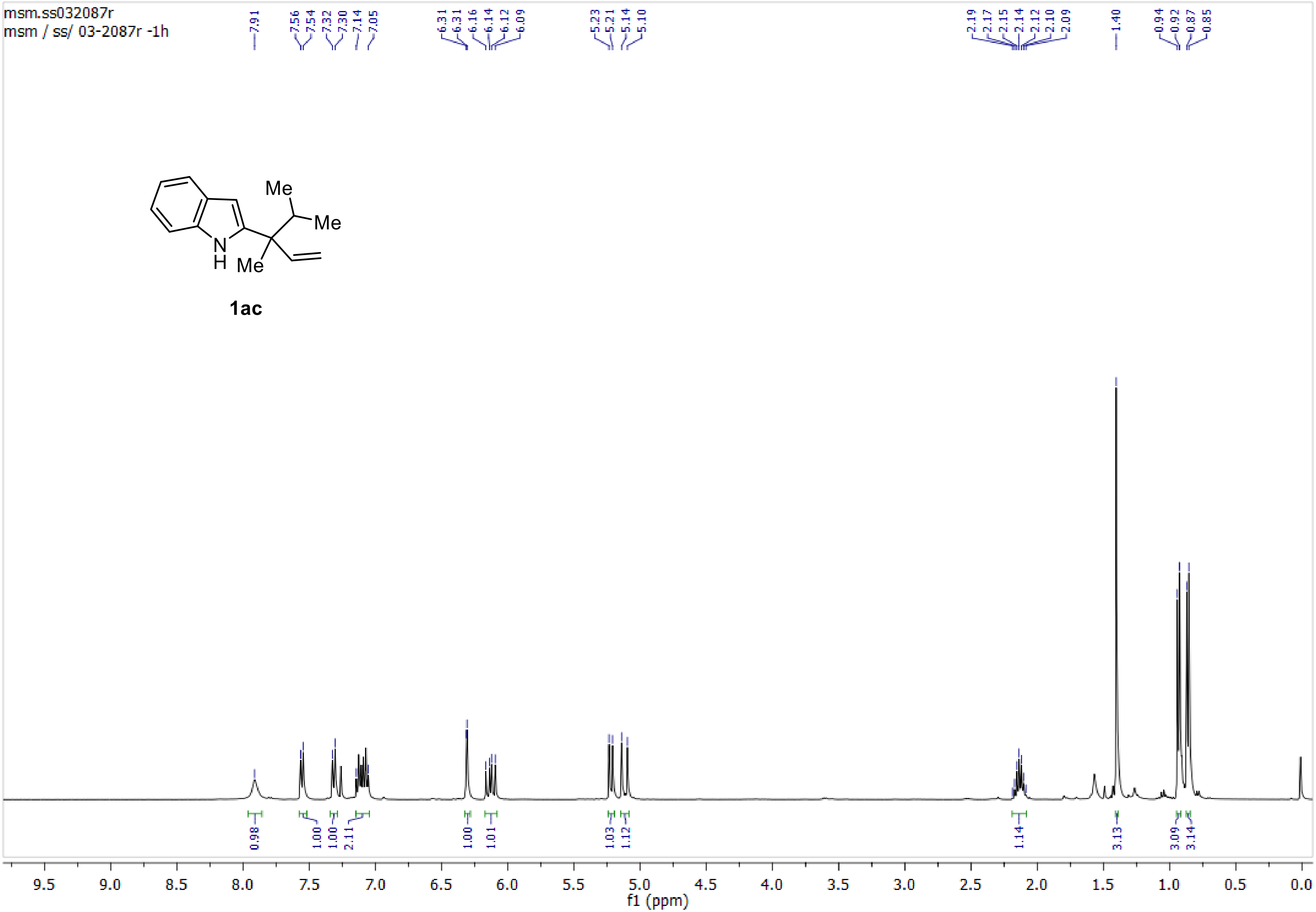
msm.ss032087r
msm / ss/ 03-2087r -1h
7.91
7.56 7.54 7.32 7.30 7.14 7.05
6.31 6.31 6.16 6.14 6.12 6.09
5.23 5.21 5.14 5.10
2.19 2.17 2.15 2.14 2.12 2.10 2.09
1.40
0.94 0.92 0.87 0.85
Me
Me
N
H
Me
1ac
0.98
1.00
1.00
2.11
1.00
1.01
1.03
1.12
1.14
3.13
3.09
3.14
9.5 9.0 8.5 8.0 7.5 7.0 6.5 6.0 5.5 5.0 4.5 4.0 3.5 3.0 2.5 2.0 1.5 1.0 0.5 0.0
f1 (ppm)

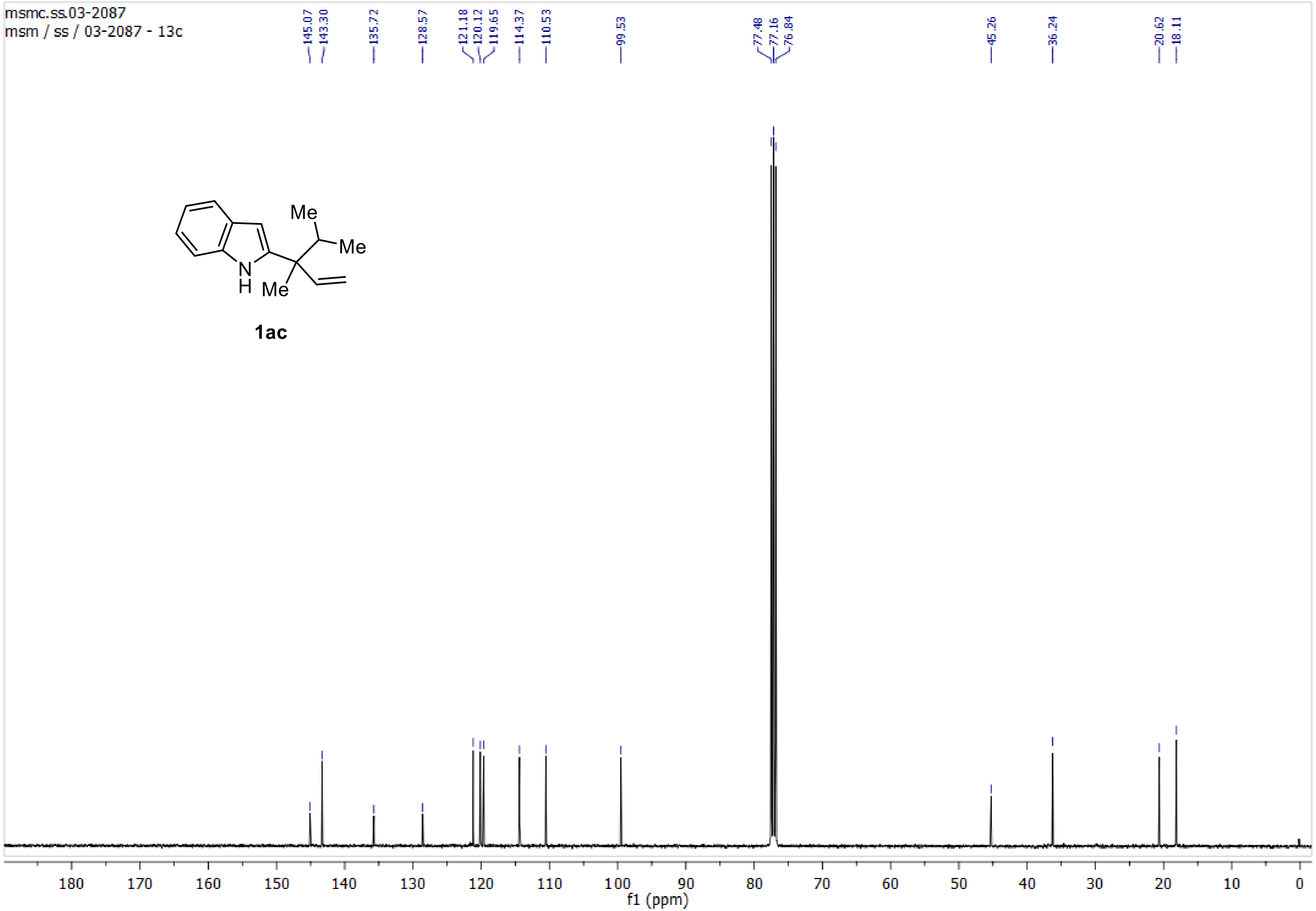
msmc.ss.03-2087
msm / ss / 03-2087 - 13c
145.07
143.30
135.72
128.57
121.18
120.12
119.65
114.37
110.53
99.53
77.48
77.16
76.84
45.26
36.24
20.62
18.11
Me
Me
N
H
Me
1ac
180
170
160
150
140
130
120
110
100
90
80
70
60
50
40
30
20
10
0
f1 (ppm)

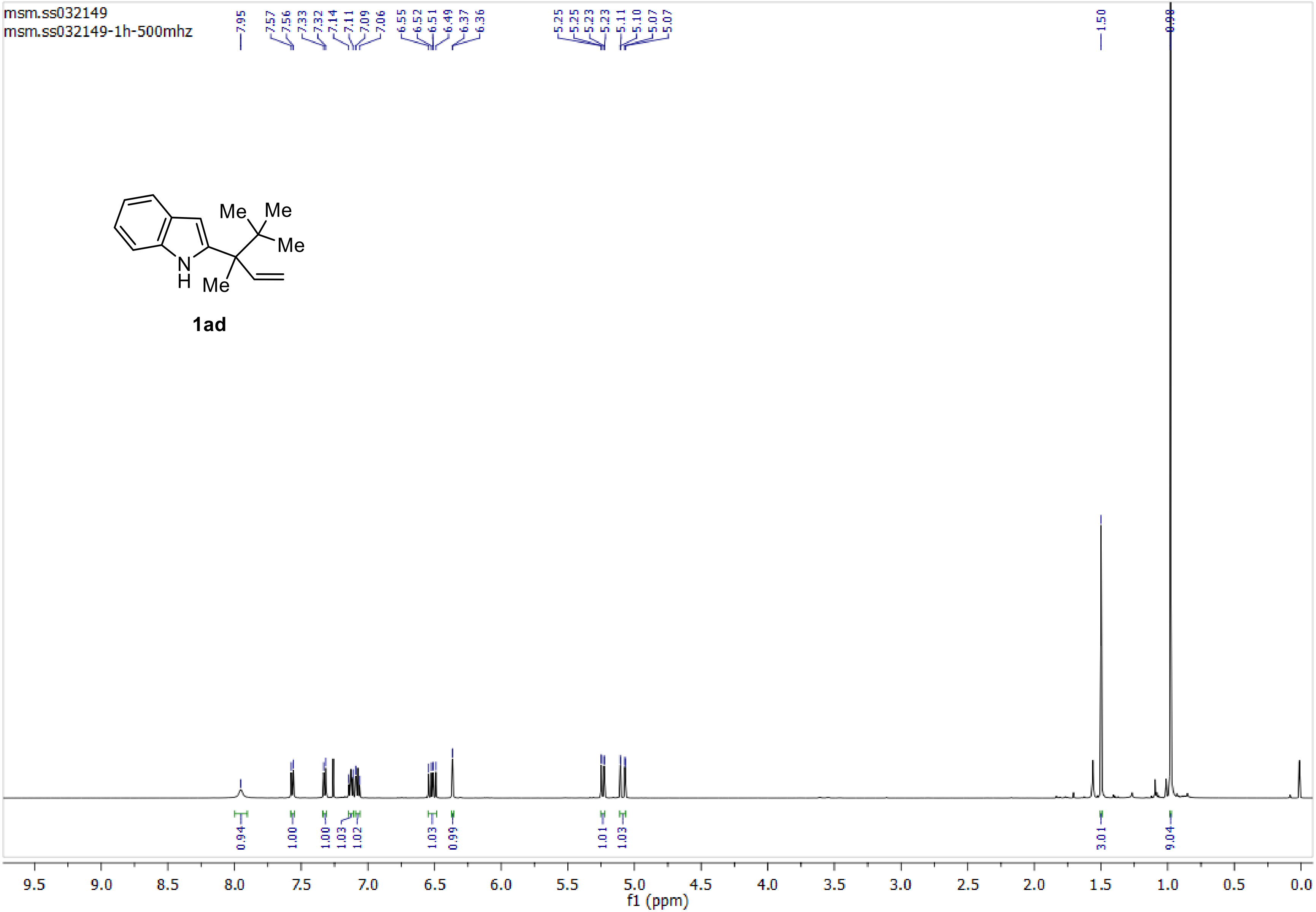
msm.ss032149
msm.ss032149-1h-500mhz
7.95
7.57
7.56
7.33
7.32
7.14
7.11
7.09
7.06
6.55
6.52
6.51
6.49
6.37
6.36
5.25
5.25
5.23
5.23
5.11
5.10
5.07
5.07
1.50
0.98
Me
Me
Me
Me
N
H
1ad
0.94
1.00
1.00
1.03
1.02
1.03
0.99
1.01
1.03
3.01
9.04
9.5
9.0
8.5
8.0
7.5
7.0
6.5
6.0
5.5
5.0
4.5
4.0
3.5
3.0
2.5
2.0
1.5
1.0
0.5
0.0
f1 (ppm)

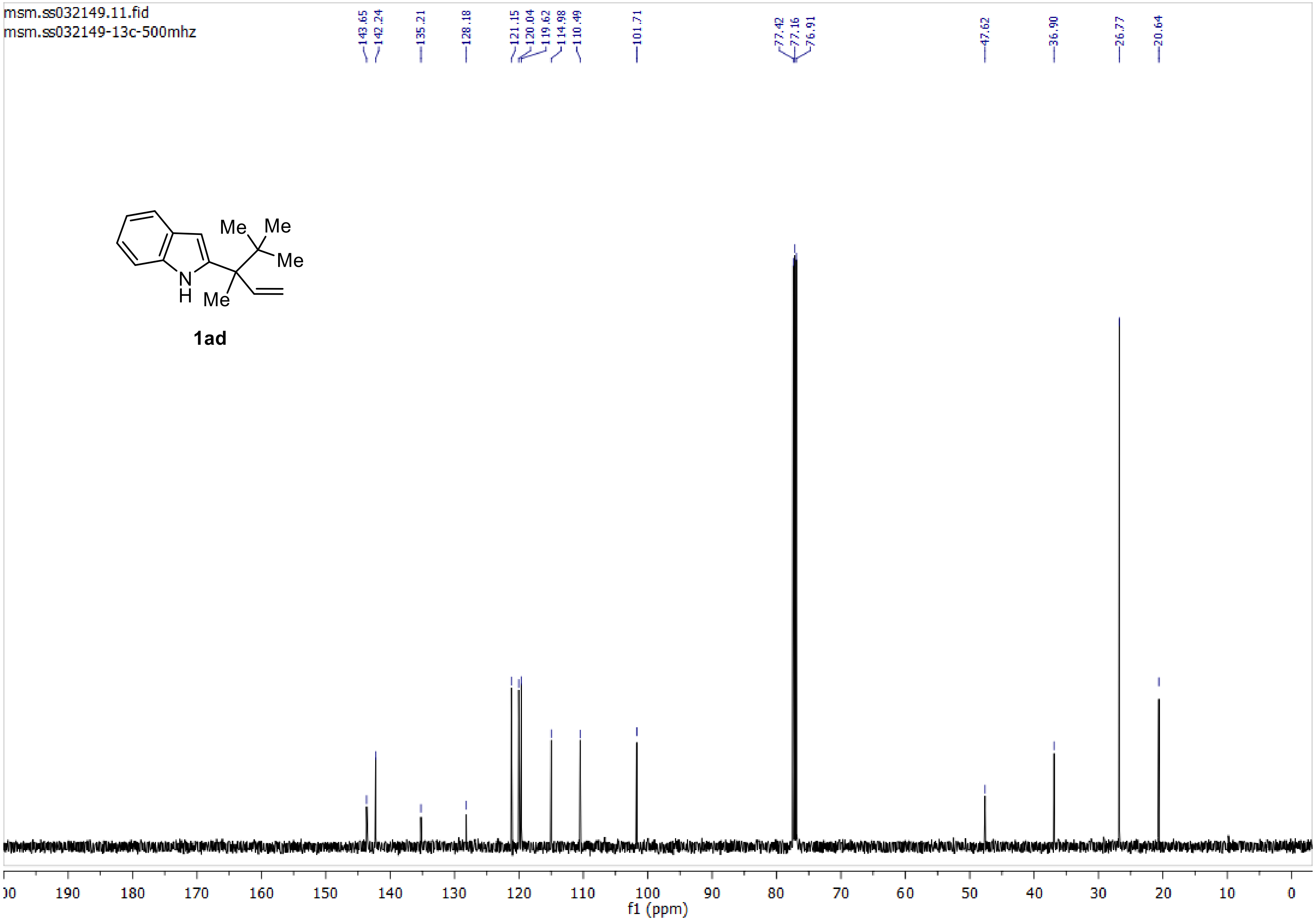

msm.ss032149.11.fid
msm.ss032149-13c-500mhz
143.65
142.24
135.21
128.18
121.15
120.04
119.62
114.98
110.49
101.71
77.42
77.16
76.91
47.62
36.90
26.77
20.64
Me
Me
Me
N
H
Me
1ad
190
180
170
160
150
140
130
120
110
100
90
80
70
60
50
40
30
20
10
0
f1 (ppm)

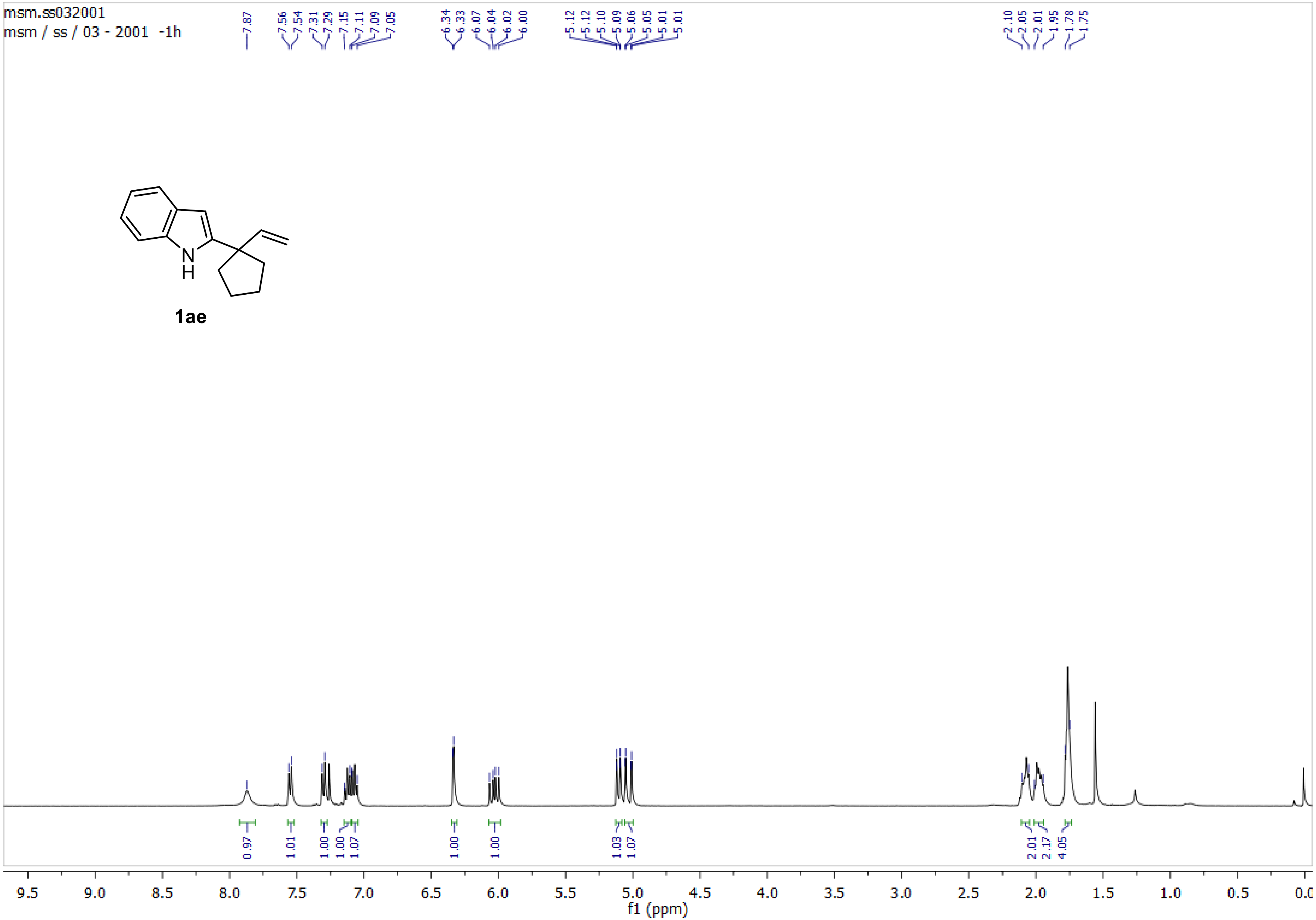
msm.ss032001
msm / ss / 03 - 2001 -1h
7.87
7.56 7.54 7.31 7.29 7.15 7.11 7.09 7.05
6.34 6.33 6.07 6.04 6.02 6.00
5.12 5.12 5.10 5.09 5.06 5.05 5.01 5.01
2.10 2.05 2.01 1.95 1.78 1.75
N
H
1ae
0.97 1.01 1.00 1.00 1.07 1.00 1.00 1.03 1.07 2.01 2.17 4.05
9.5 9.0 8.5 8.0 7.5 7.0 6.5 6.0 5.5 5.0 4.5 4.0 3.5 3.0 2.5 2.0 1.5 1.0 0.5 0.0
f1 (ppm)

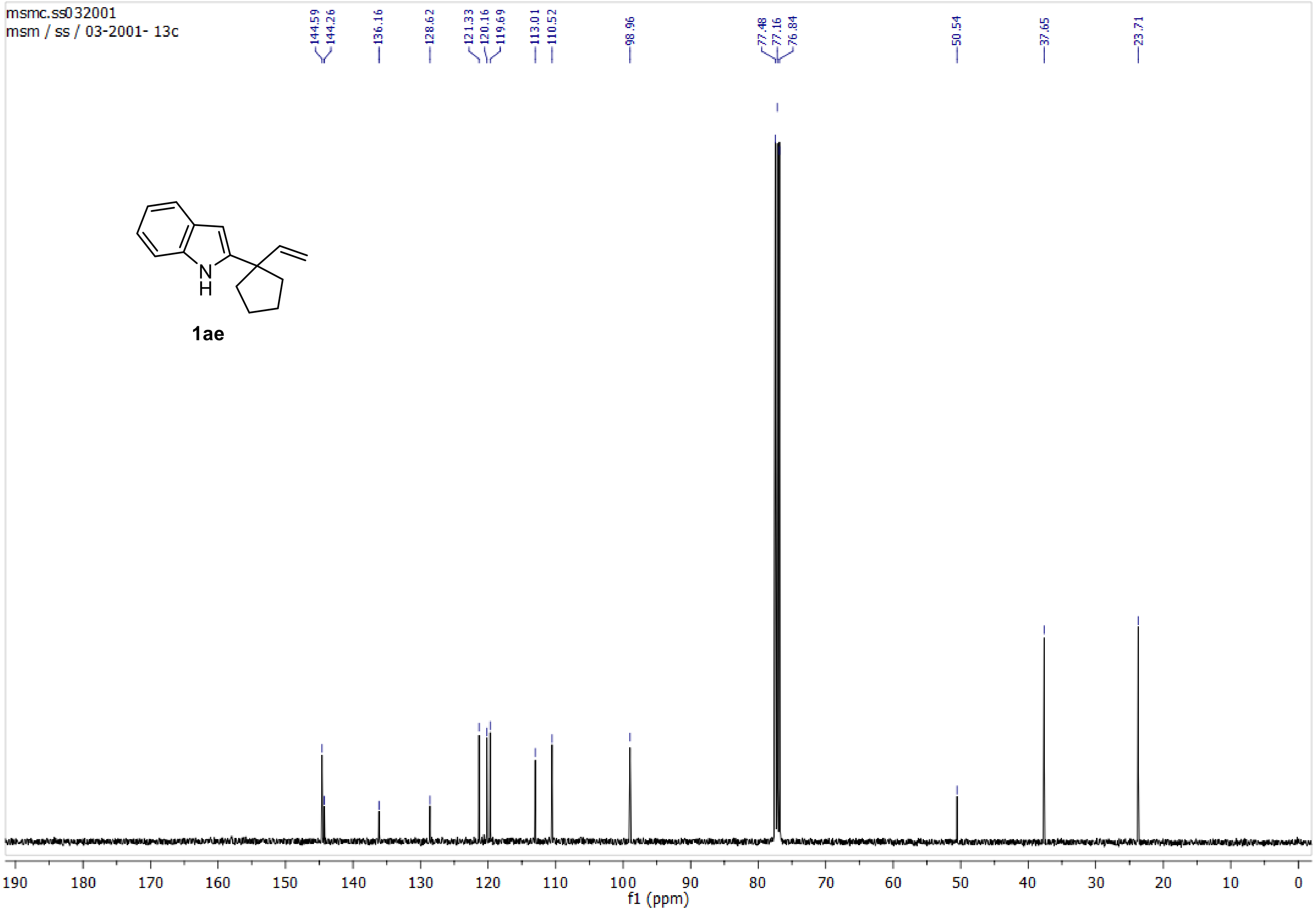
msmc.ss032001
msm / ss / 03-2001- 13c
144.59
144.26
136.16
128.62
121.33
120.16
119.69
113.01
110.52
98.96
77.48
77.16
76.84
50.54
37.65
23.71
1ae
190 180 170 160 150 140 130 120 110 100 90 80 70 60 50 40 30 20 10 0
f1 (ppm)

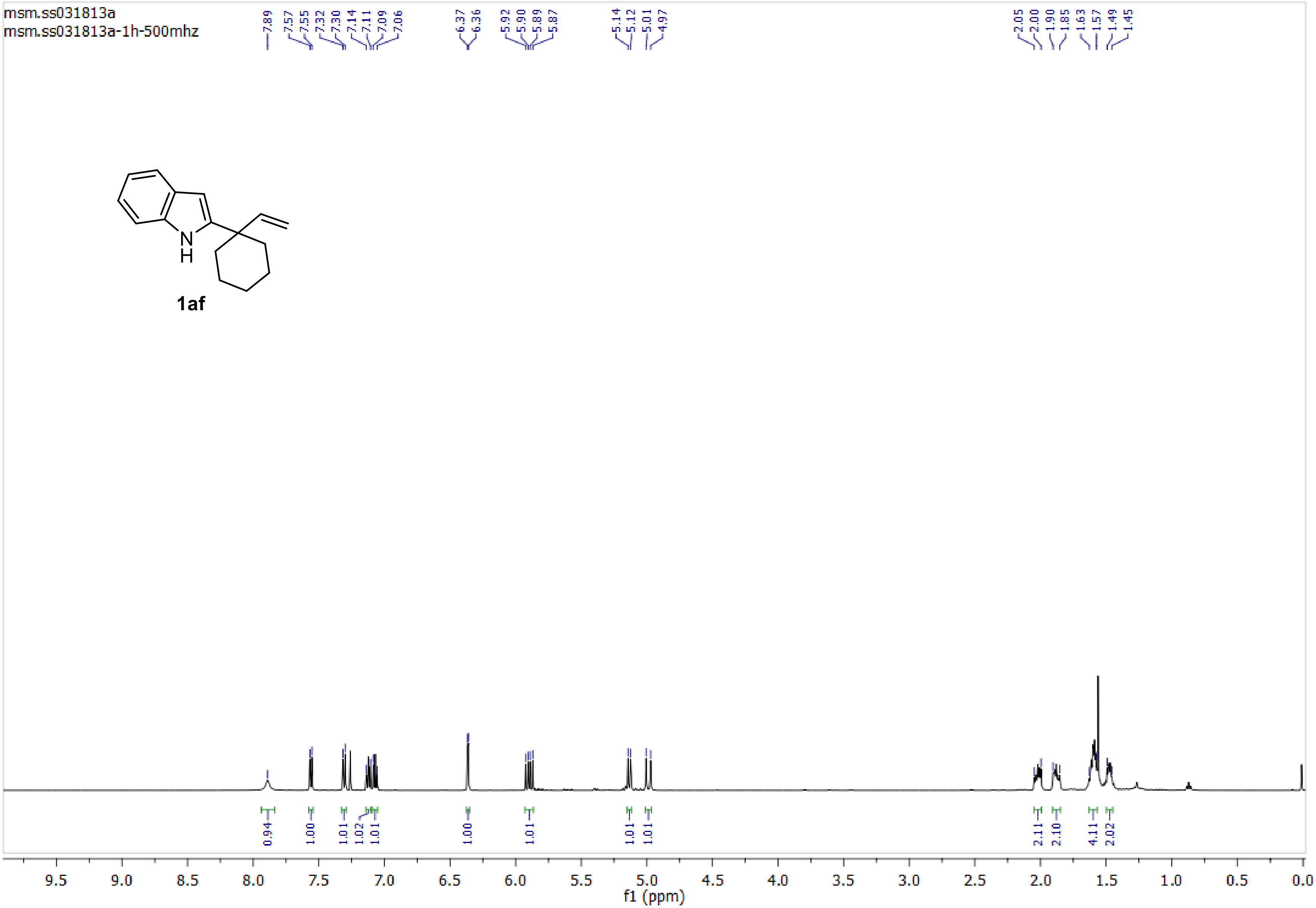
1af
msm.ss031813a
msm.ss031813a-1h-500mhz
7.89
7.57
7.55
7.32
7.30
7.14
7.11
7.09
7.06
6.37
6.36
5.92
5.90
5.89
5.87
5.14
5.12
5.01
4.97
2.05
2.00
1.90
1.85
1.63
1.57
1.49
1.45
0.94
1.00
1.01
1.02
1.01
1.00
1.01
1.01
1.01
2.11
2.10
4.11
2.02
9.5
9.0
8.5
8.0
7.5
7.0
6.5
6.0
5.5
5.0
4.5
4.0
3.5
3.0
2.5
2.0
1.5
1.0
0.5
0.0
f1 (ppm)

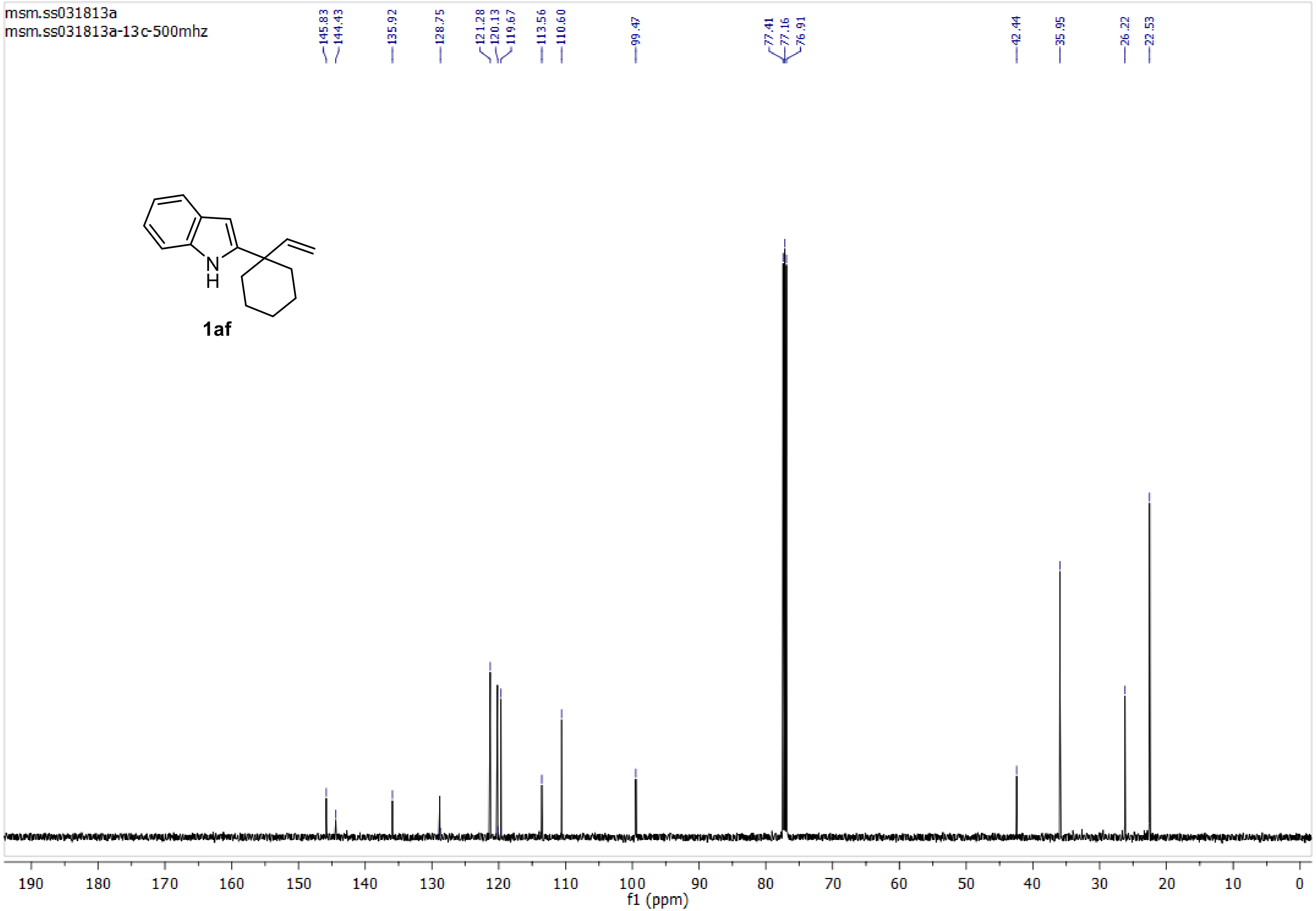
msm.ss031813a
msm.ss031813a-13c-500mhz
145.83
144.43
135.92
128.75
121.28
120.13
119.67
113.56
110.60
99.47
77.41
77.16
76.91
42.44
35.95
26.22
22.53
N
H
1af
190
180
170
160
150
140
130
120
110
100
90
80
70
60
50
40
30
20
10
0
f1 (ppm)

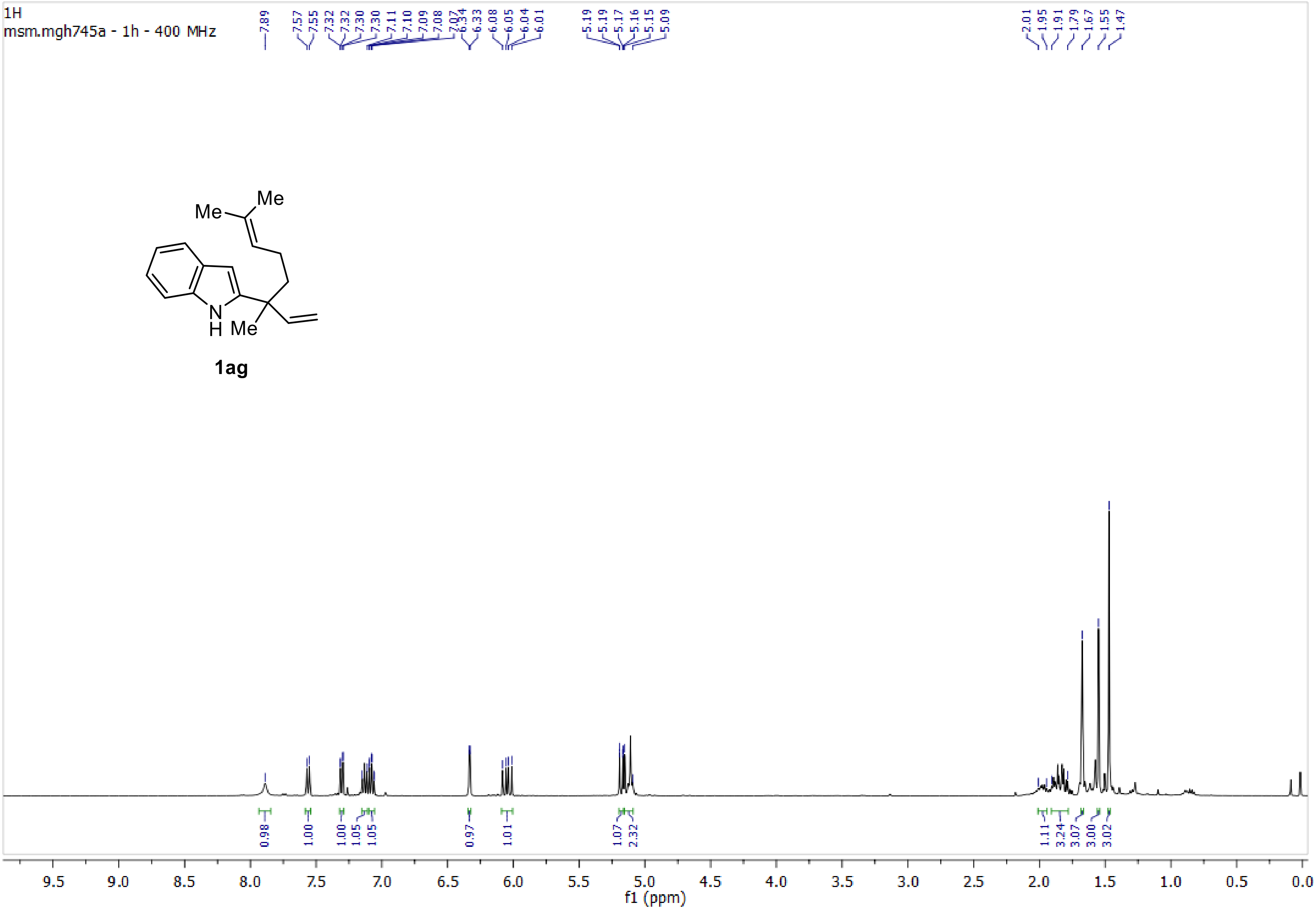
1H
msm.mgh745a - 1h - 400 MHz
7.89 7.57 7.55 7.32 7.32 7.30 7.30 7.11 7.10 7.09 7.08 7.07 6.34 6.33 6.08 6.05 6.04 6.01
5.19 5.19 5.17 5.16 5.15 5.09
2.01 1.95 1.91 1.79 1.67 1.55 1.47
Me
Me
N
H Me
1ag
0.98 1.00 1.00 1.05 1.05 0.97 1.01 1.07 2.32 1.11 3.24 3.07 3.00 3.02
9.5 9.0 8.5 8.0 7.5 7.0 6.5 6.0 5.5 5.0 4.5 4.0 3.5 3.0 2.5 2.0 1.5 1.0 0.5 0.0
f1 (ppm)

13C
msm.mgh745 13C NMR 400 MHz

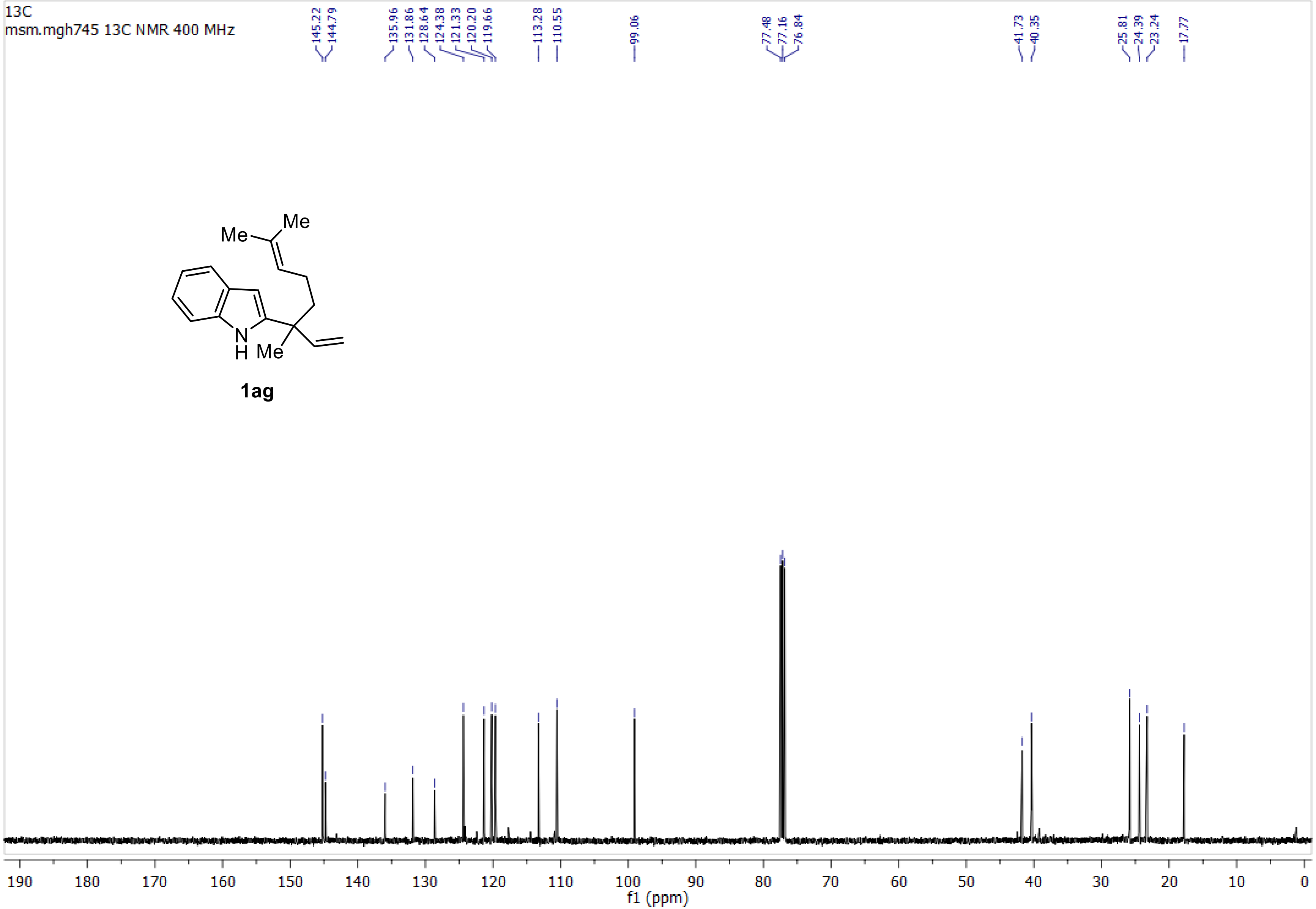

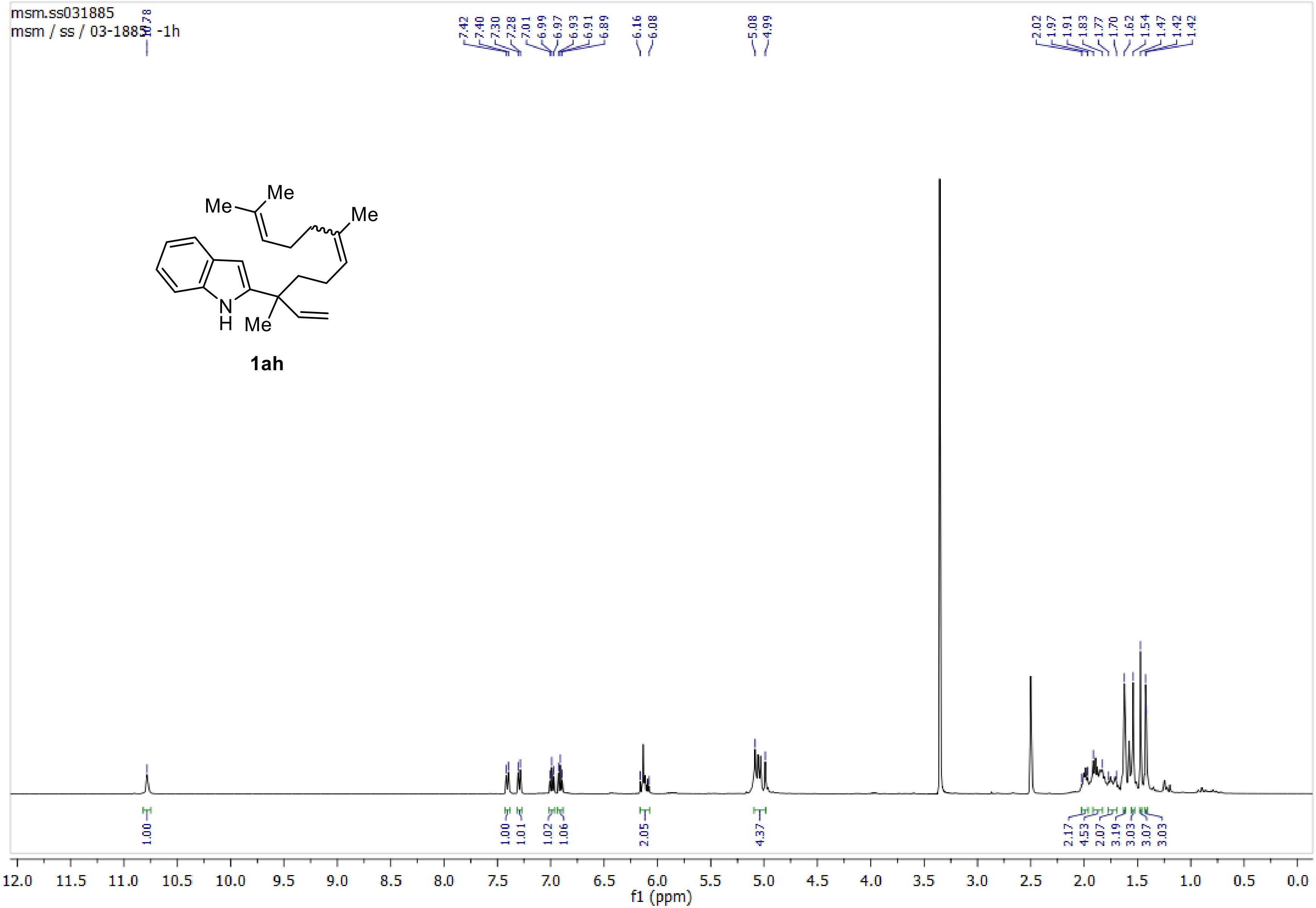
msm.ss031885
msm / ss / 03-1885 -1h
10.78
7.42
7.40
7.30
7.28
7.01
6.99
6.97
6.93
6.91
6.89
6.16
6.08
5.08
4.99
2.02
1.97
1.91
1.83
1.77
1.70
1.62
1.54
1.47
1.42
1.42
Me
Me
Me
N
H
Me
1ah
1.00
1.00
1.01
1.02
1.06
2.05
4.37
2.17
4.53
2.07
3.19
3.03
3.07
3.03
12.0 11.5 11.0 10.5 10.0 9.5 9.0 8.5 8.0 7.5 7.0 6.5 6.0 5.5 5.0 4.5 4.0 3.5 3.0 2.5 2.0 1.5 1.0 0.5 0.0
f1 (ppm)

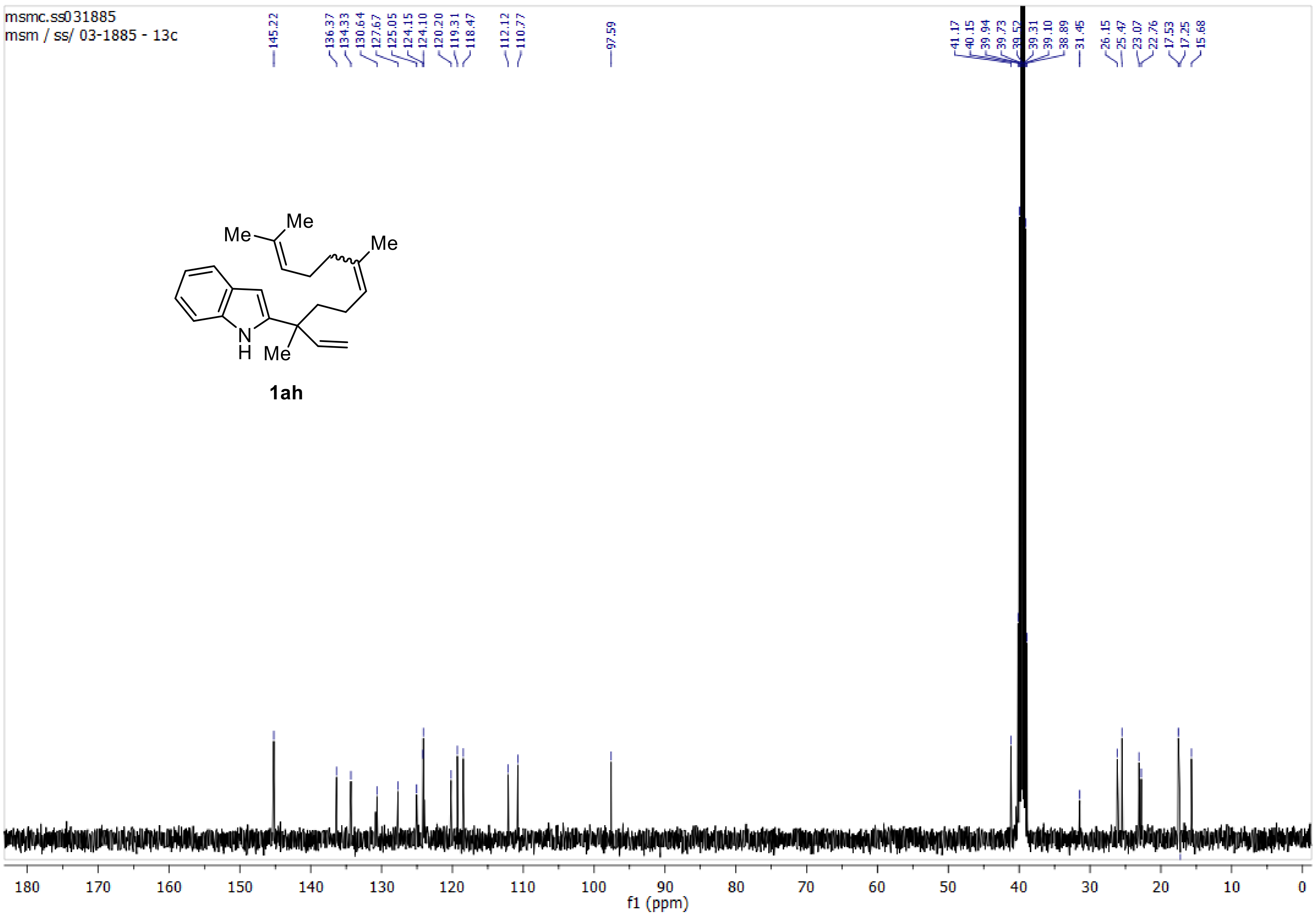
msmc.ss031885
msm / ss/ 03-1885 - 13c
145.22
136.37
134.33
130.64
127.67
125.05
124.15
124.10
120.20
119.31
118.47
112.12
110.77
97.59
41.17
40.15
39.94
39.73
39.52
39.31
39.10
38.89
31.45
26.15
25.47
23.07
22.76
17.53
17.25
15.68
Me
Me
Me
N
H
Me
1ah
180
170
160
150
140
130
120
110
100
90
80
70
60
50
40
30
20
10
0
f1 (ppm)

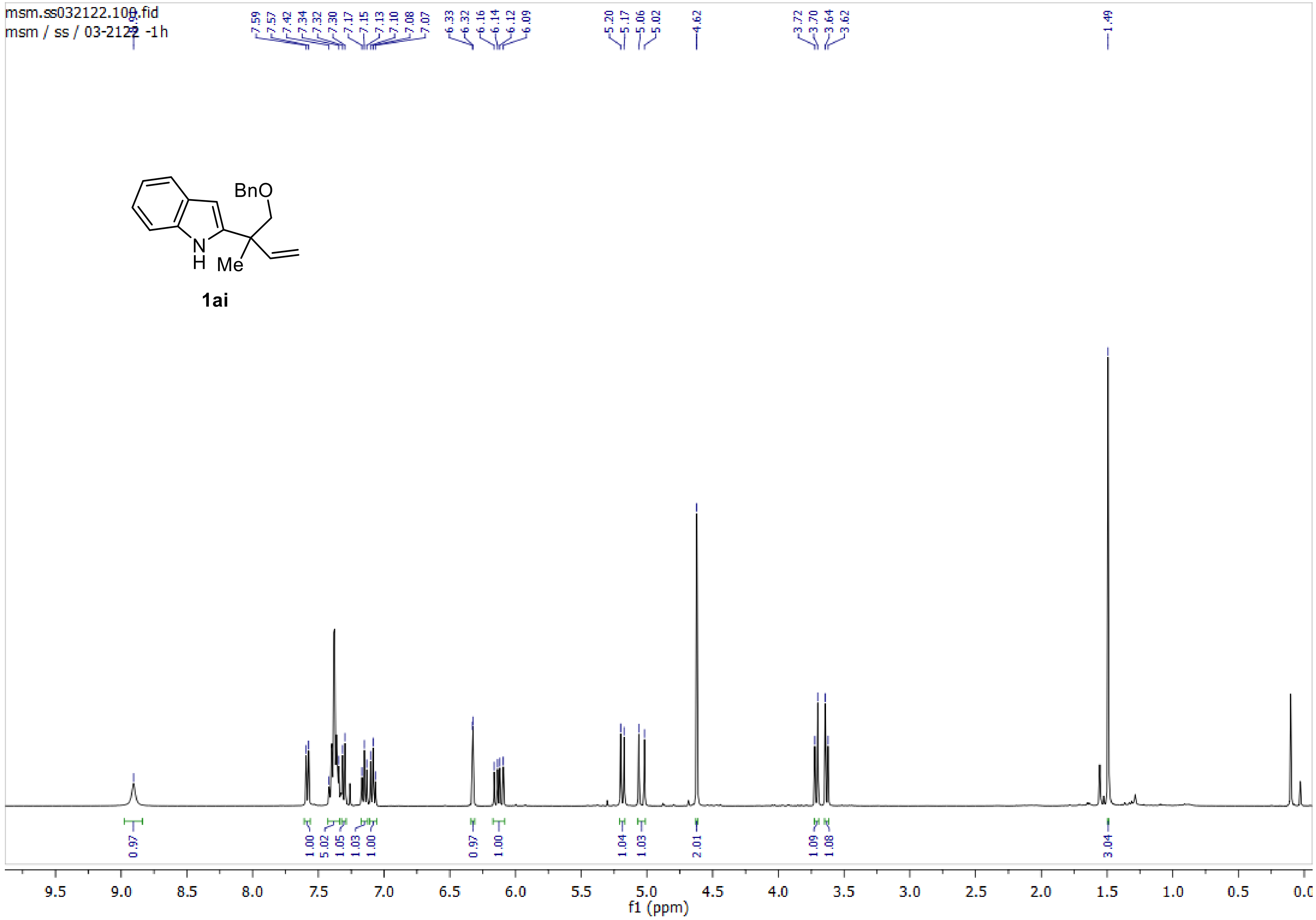

msm.ss032122.100.fid
msm / ss / 03-2122 -1h
BnO
N
H
Me
1ai
f1 (ppm)

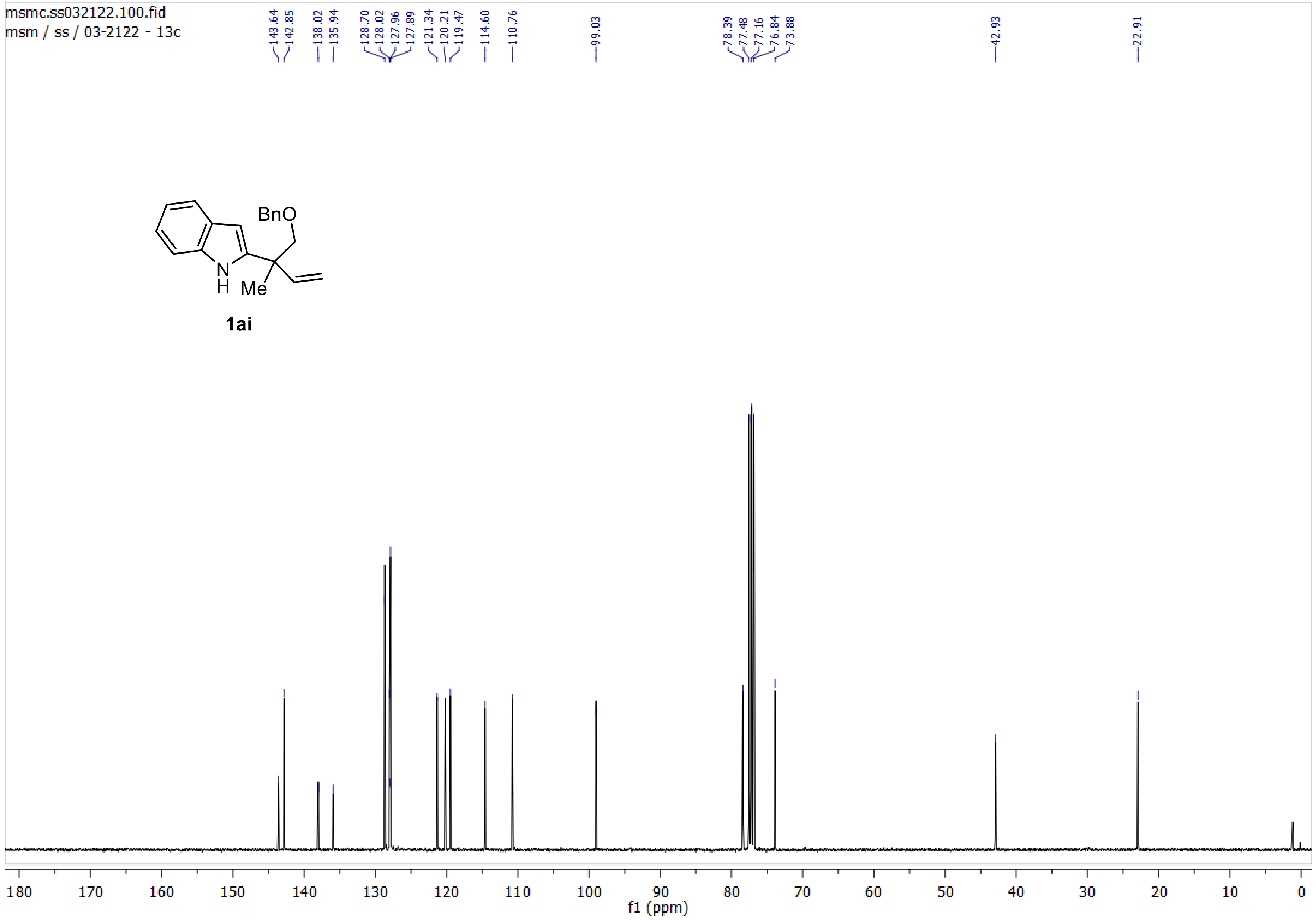
msmc.ss032122.100.fid
msm / ss / 03-2122 - 13c
143.64
142.85
138.02
135.94
128.70
128.02
127.96
127.89
121.34
120.21
119.47
114.60
110.76
99.03
78.39
77.48
77.16
76.84
73.88
42.93
22.91
BnO
N
H
Me
1ai
180
170
160
150
140
130
120
110
100
90
80
70
60
50
40
30
20
10
0
f1 (ppm)

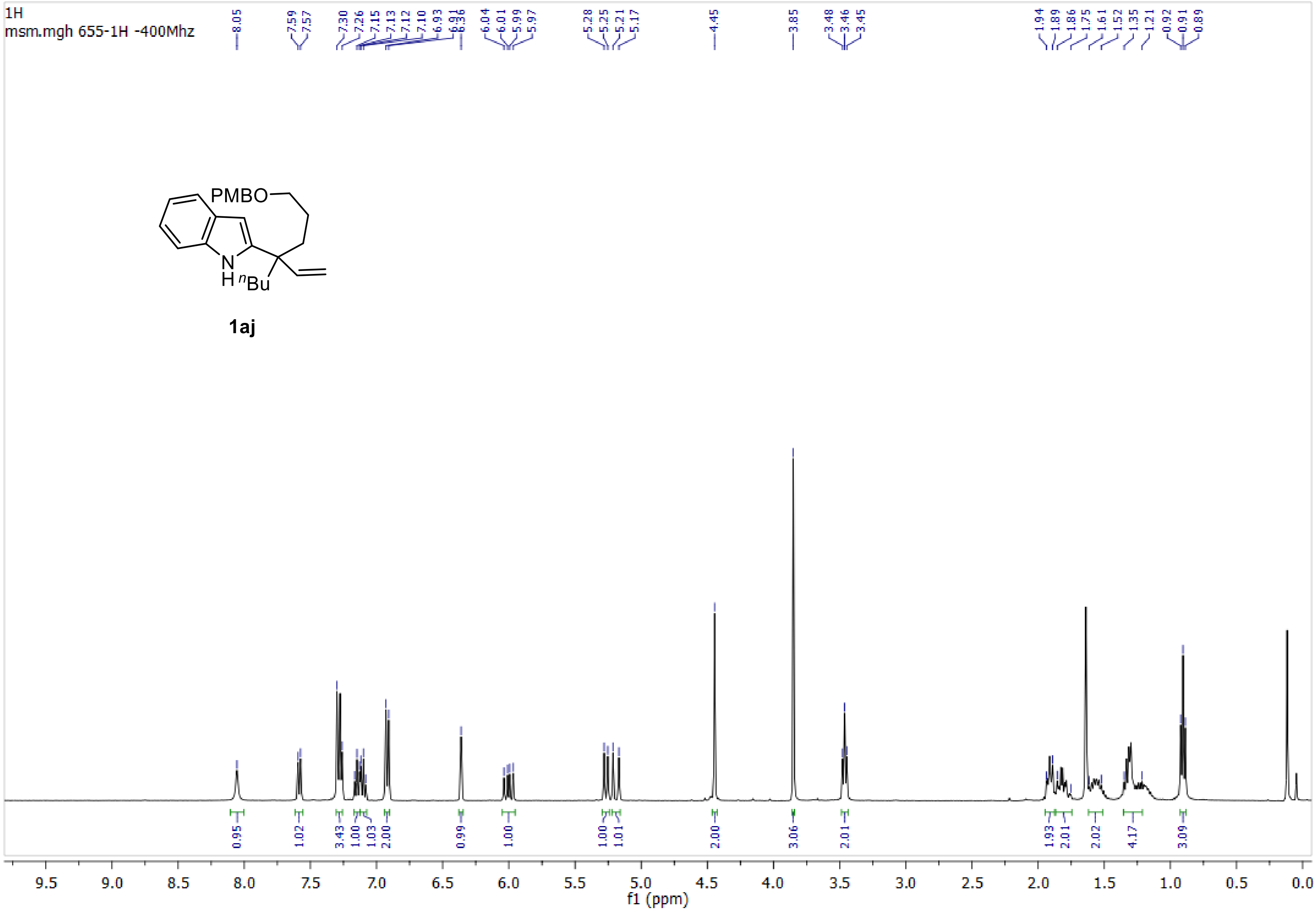
1H
msm.mgh 655-1H -400Mhz
PMBO
N
H
nBu
1aj
f1 (ppm)

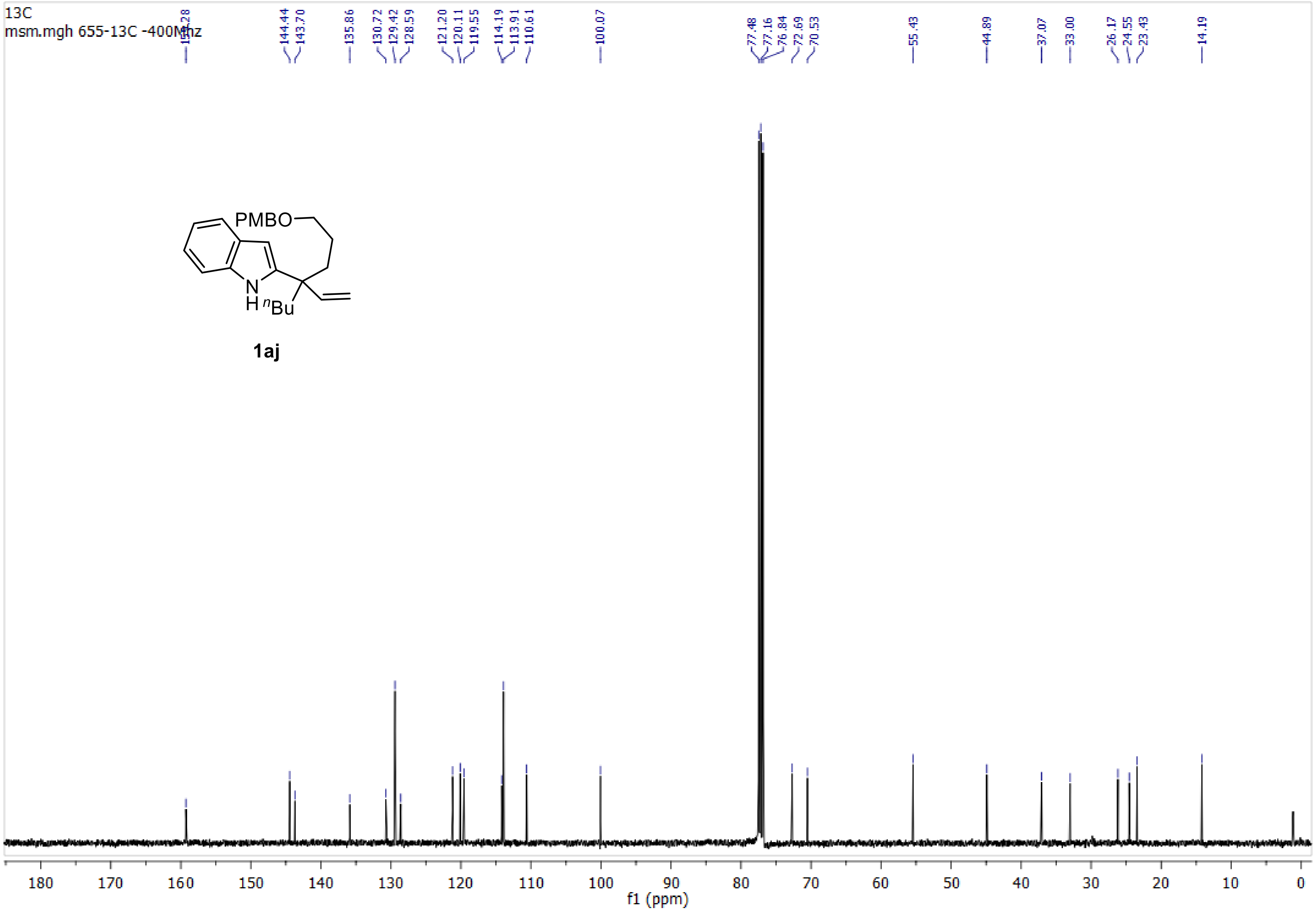
13C
msm.mgh 655-13C -400Mhz
144.44
143.70
135.86
130.72
129.42
128.59
121.20
120.11
119.55
114.19
113.91
110.61
100.07
77.48
77.16
76.84
72.69
70.53
55.43
44.89
37.07
33.00
26.17
24.55
23.43
14.19
PMBO
N
H
nBu
1aj
180
170
160
150
140
130
120
110
100
90
80
70
60
50
40
30
20
10
0
f1 (ppm)

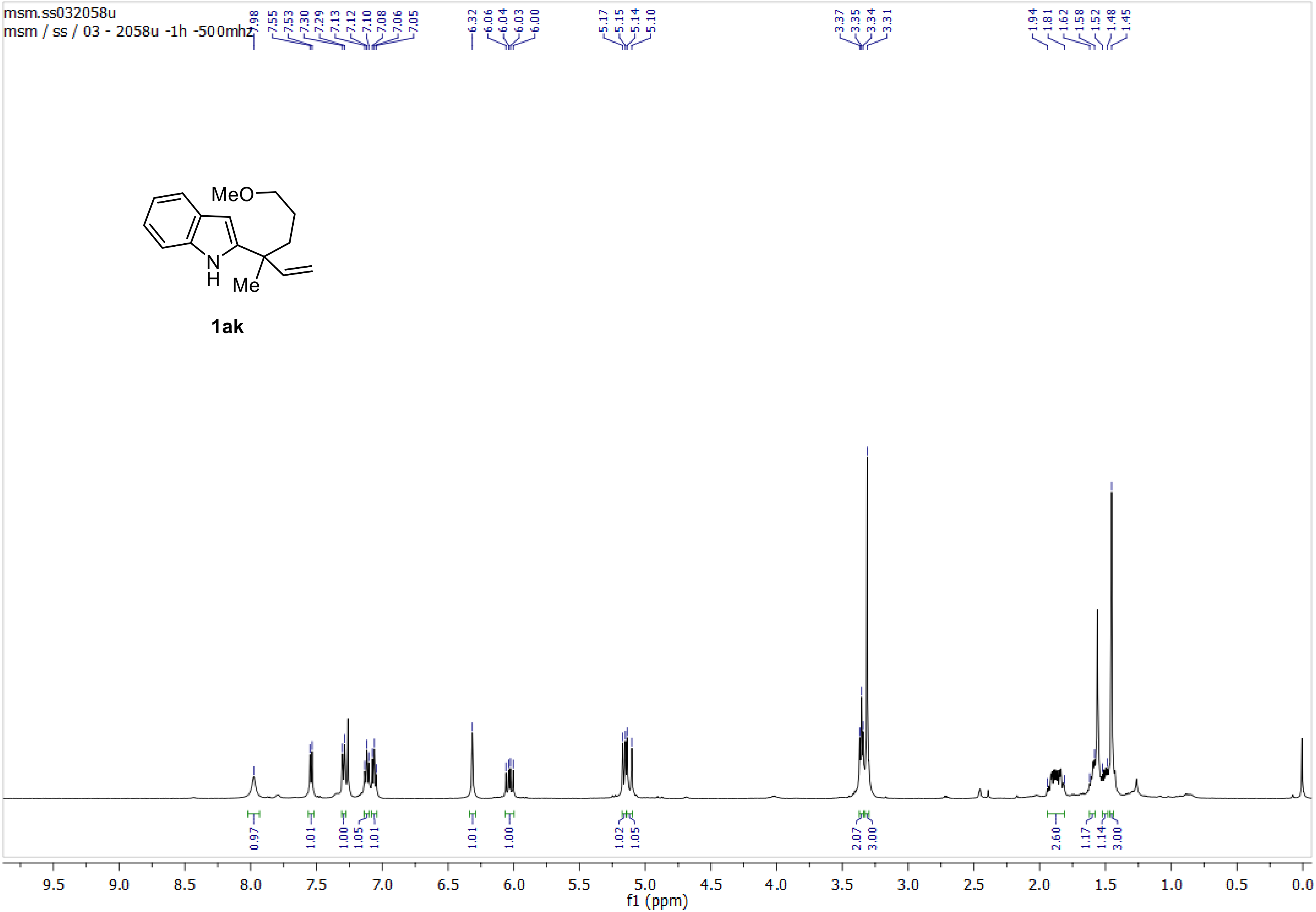
1ak
msm.ss032058u
msm / ss / 03 - 2058u -1h -500mhz
7.98 7.55 7.53 7.30 7.29 7.13 7.12 7.10 7.08 7.06 7.05
6.32 6.06 6.04 6.03 6.00
5.17 5.15 5.14 5.10
3.37 3.35 3.34 3.31
1.94 1.81 1.62 1.58 1.52 1.48 1.45
0.97 1.01 1.00 1.05 1.01 1.01 1.00 1.02 1.05 2.07 3.00 2.60 1.17 1.14 3.00
9.5 9.0 8.5 8.0 7.5 7.0 6.5 6.0 5.5 5.0 4.5 4.0 3.5 3.0 2.5 2.0 1.5 1.0 0.5 0.0
f1 (ppm)

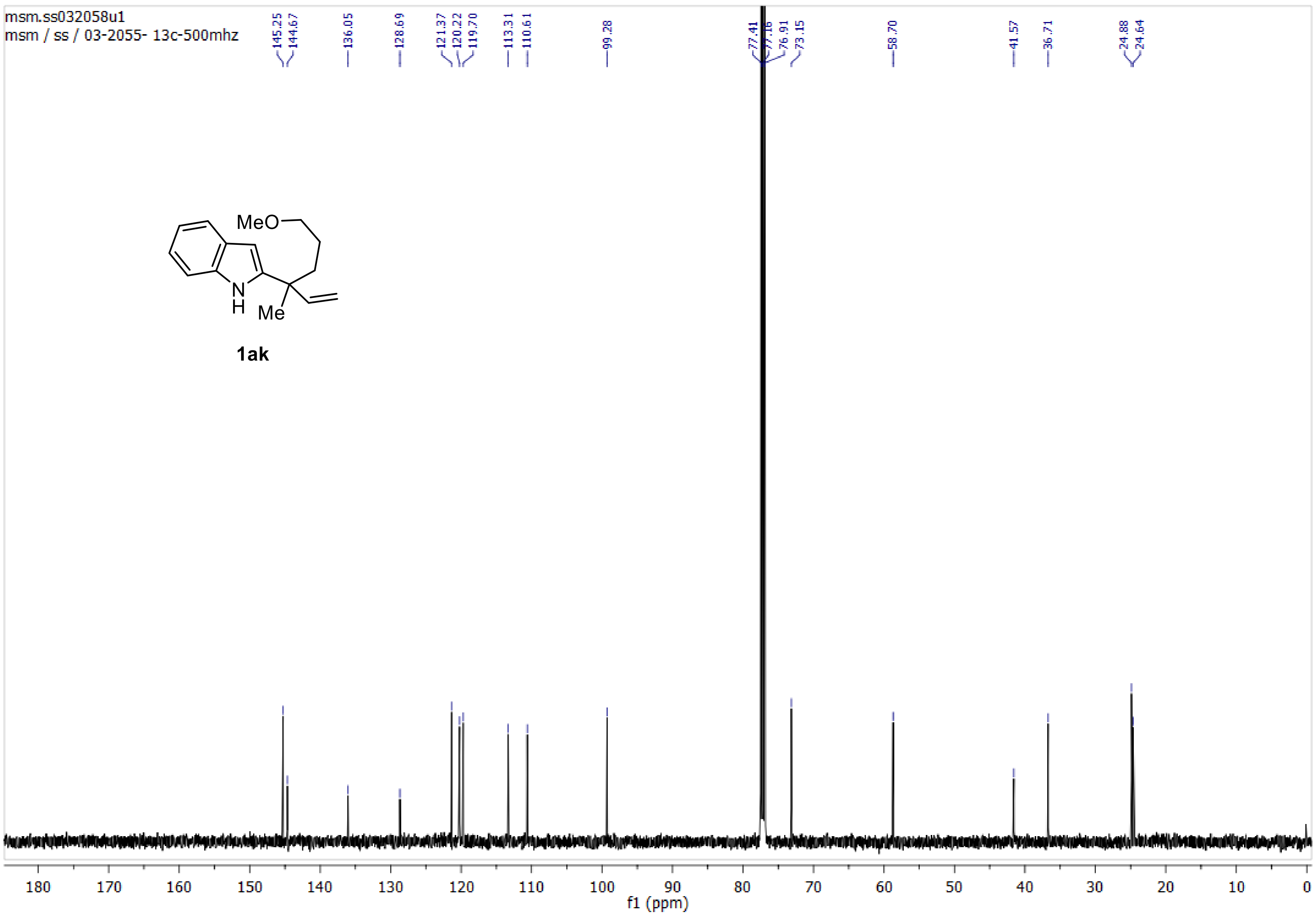
msm.ss032058u1
msm / ss / 03-2055- 13c-500mhz
145.25
144.67
136.05
128.69
121.37
120.22
119.70
113.31
110.61
99.28
77.41
77.16
76.91
73.15
58.70
41.57
36.71
24.88
24.64
MeO
N
H
Me
1ak
180
170
160
150
140
130
120
110
100
90
80
70
60
50
40
30
20
10
0
f1 (ppm)

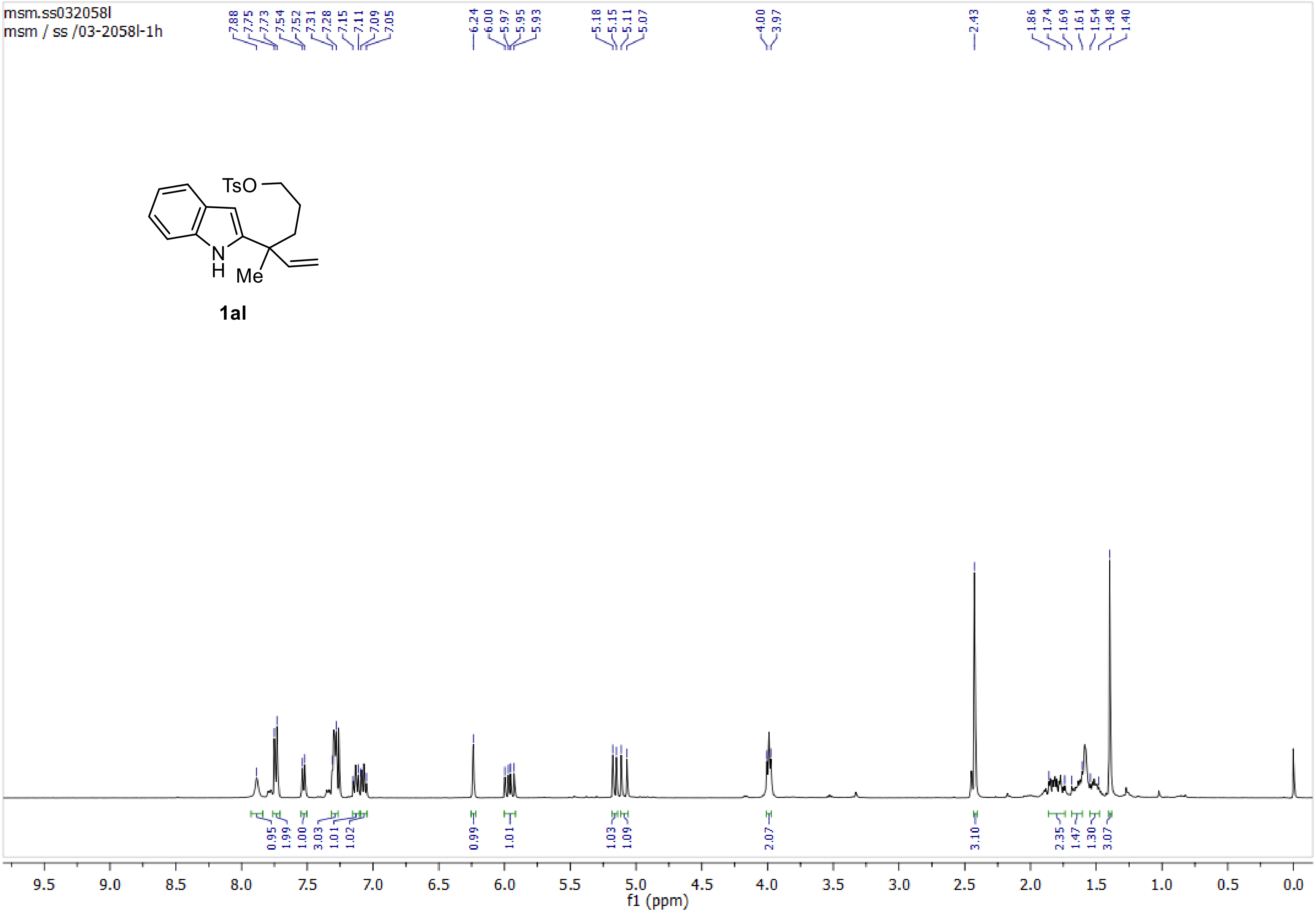
msm.ss032058l
msm / ss /03-2058l-1h
7.88
7.75
7.73
7.54
7.52
7.31
7.28
7.15
7.11
7.09
7.05
6.24
6.00
5.97
5.95
5.93
5.18
5.15
5.11
5.07
4.00
3.97
2.43
1.86
1.74
1.69
1.61
1.54
1.48
1.40
TsO
N
H
Me
1al
0.95
1.99
1.00
3.03
1.01
1.02
0.99
1.01
1.03
1.09
2.07
3.10
2.35
1.47
1.30
3.07
9.5
9.0
8.5
8.0
7.5
7.0
6.5
6.0
5.5
5.0
4.5
4.0
3.5
3.0
2.5
2.0
1.5
1.0
0.5
0.0
f1 (ppm)

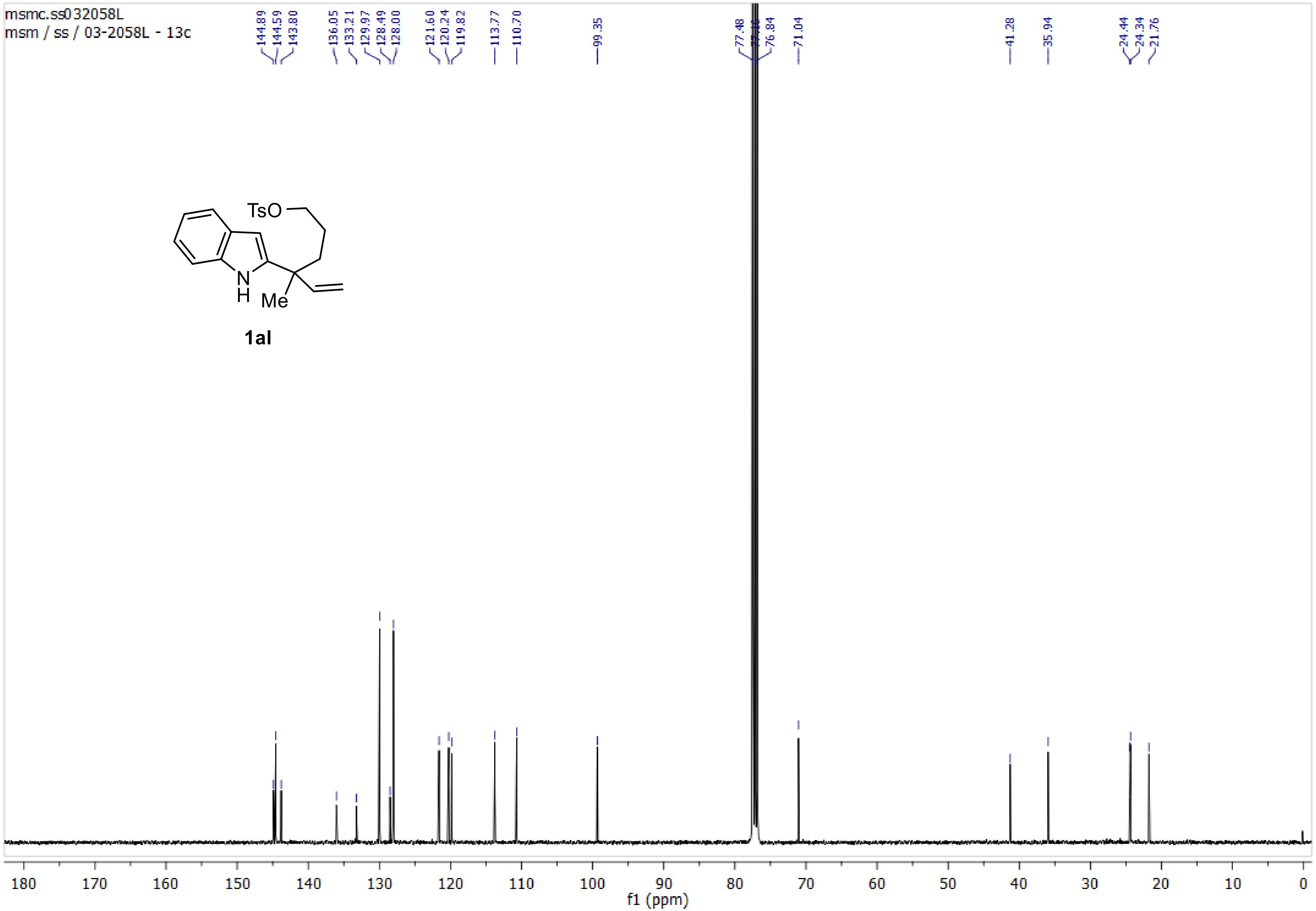
msmc.ss032058L
msm / ss / 03-2058L - 13c
144.89
144.59
143.80
136.05
133.21
129.97
128.49
128.00
121.60
120.24
119.82
113.77
110.70
99.35
77.48
76.84
71.04
41.28
35.94
24.44
24.34
21.76
TsO
N
H
Me
1al
180 170 160 150 140 130 120 110 100 90 80 70 60 50 40 30 20 10 0
f1 (ppm)

msm.ss031942a
msm / ss-03-1942a -1h

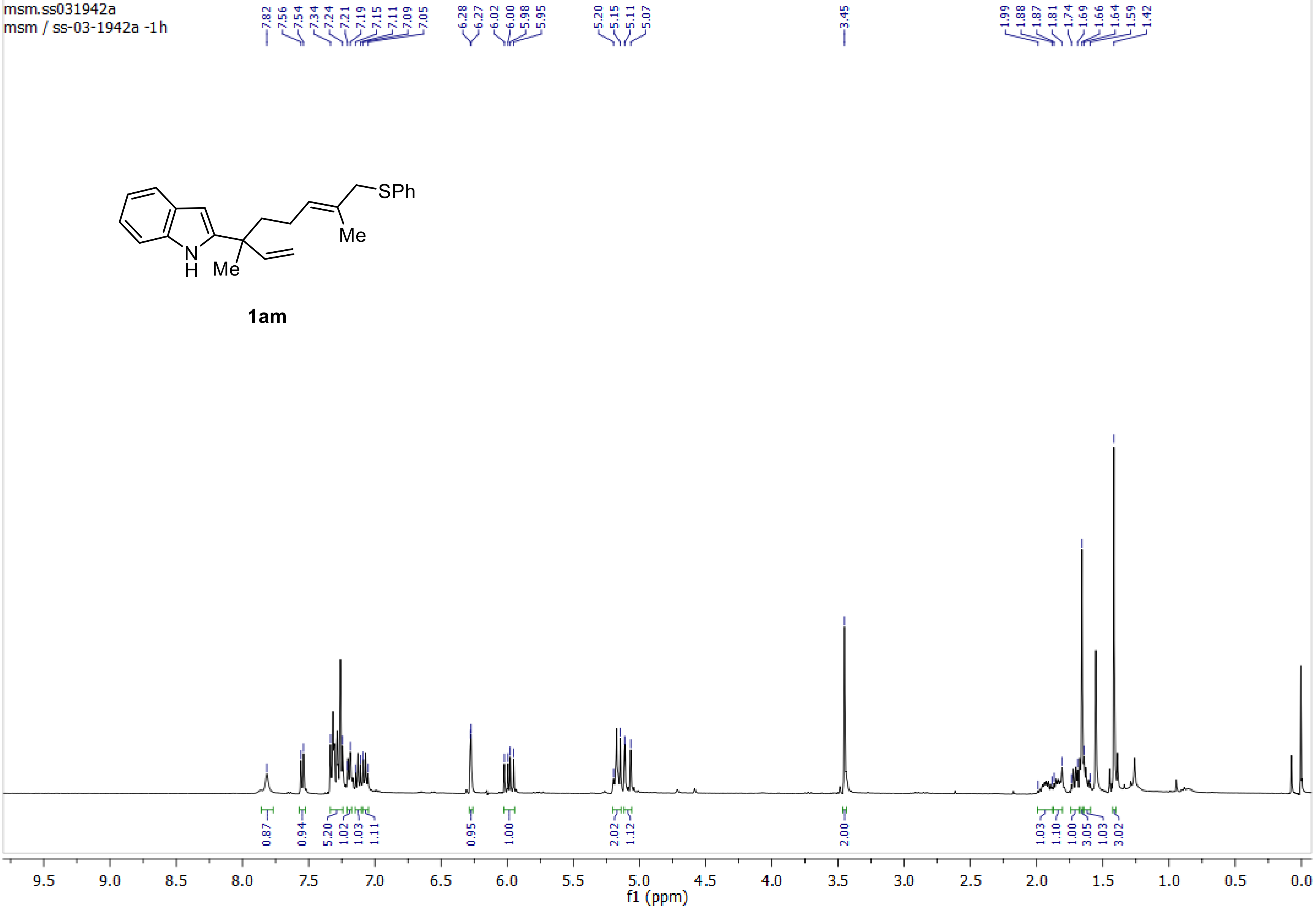

1am

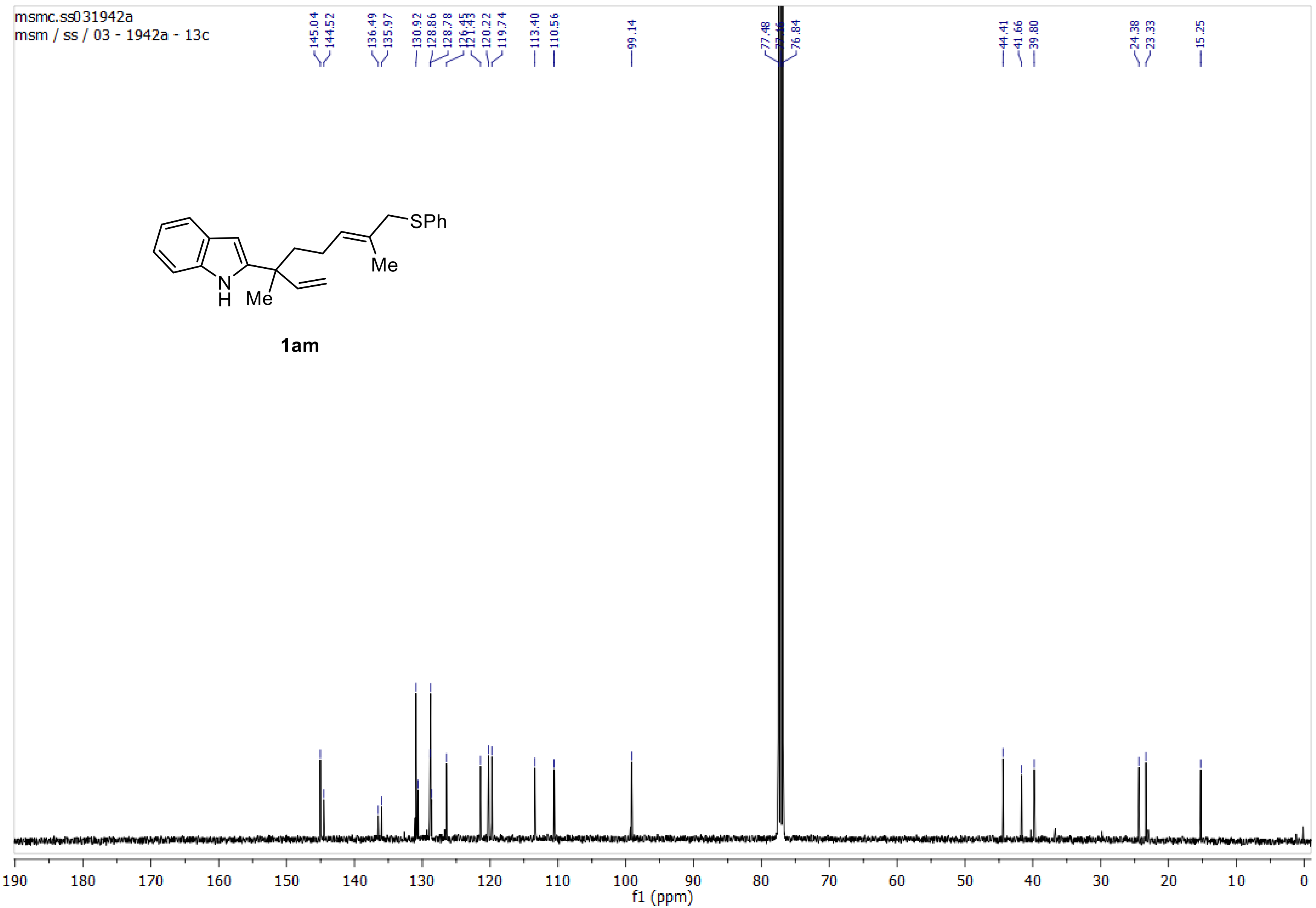

msmc.ss031942a
msm / ss / 03 - 1942a - 13c
145.04
144.52
136.49
135.97
130.92
128.86
128.78
126.45
121.43
120.22
119.74
113.40
110.56
99.14
77.48
76.84
44.41
41.66
39.80
24.38
23.33
15.25
SPh
Me
N
H
Me
1am
190
180
170
160
150
140
130
120
110
100
90
80
70
60
50
40
30
20
10
0
f1 (ppm)

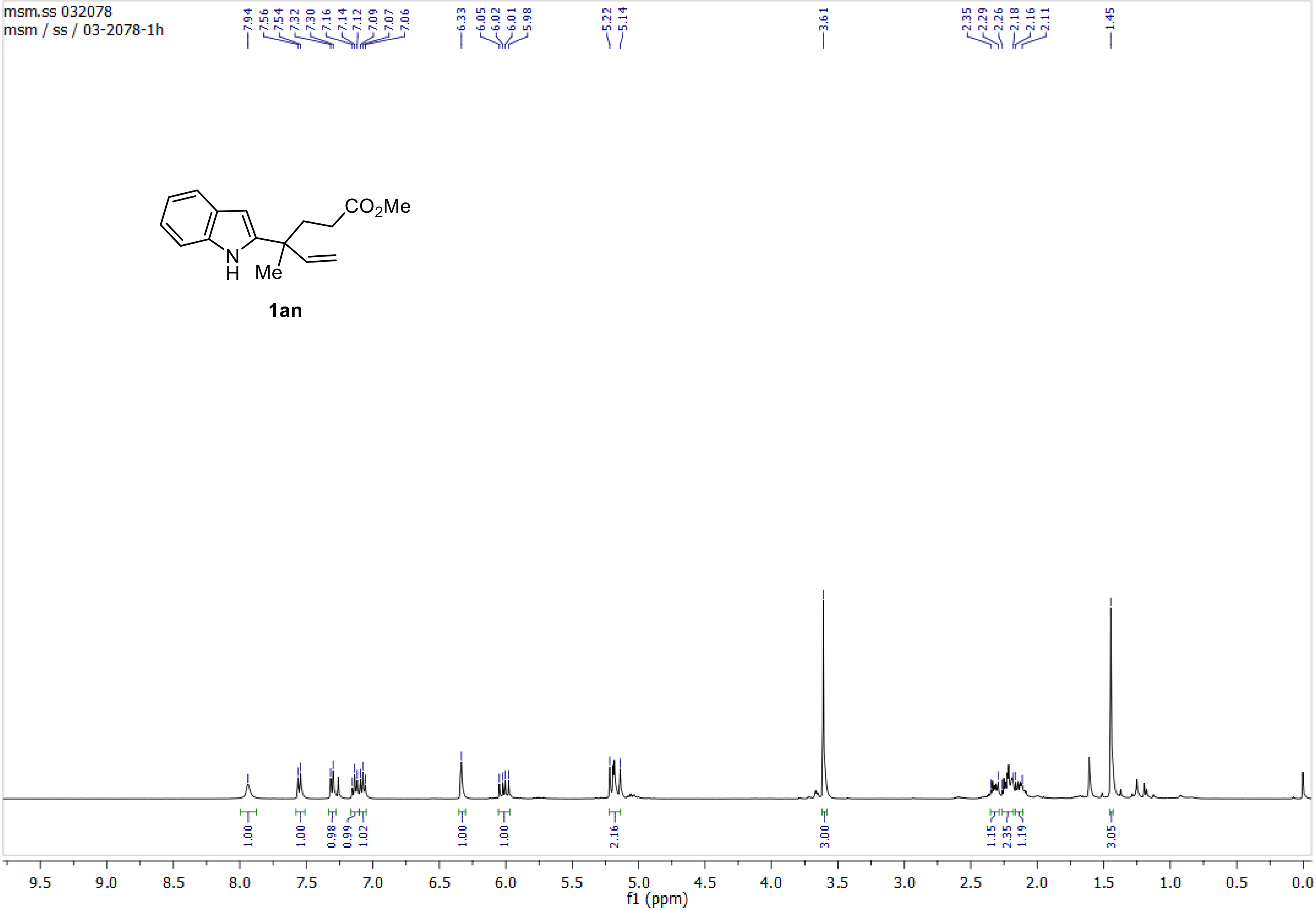
msm.ss 032078
msm / ss / 03-2078-1h
7.94
7.56
7.54
7.32
7.30
7.16
7.14
7.12
7.09
7.07
7.06
6.33
6.05
6.02
6.01
5.98
5.22
5.14
3.61
2.35
2.29
2.26
2.18
2.16
2.11
1.45
CO2Me
N
H
Me
1.00
1.00
0.98
0.99
1.02
1.00
1.00
2.16
3.00
1.15
2.35
1.19
3.05
9.5
9.0
8.5
8.0
7.5
7.0
6.5
6.0
5.5
5.0
4.5
4.0
3.5
3.0
2.5
2.0
1.5
1.0
0.5
0.0
f1 (ppm)

1an

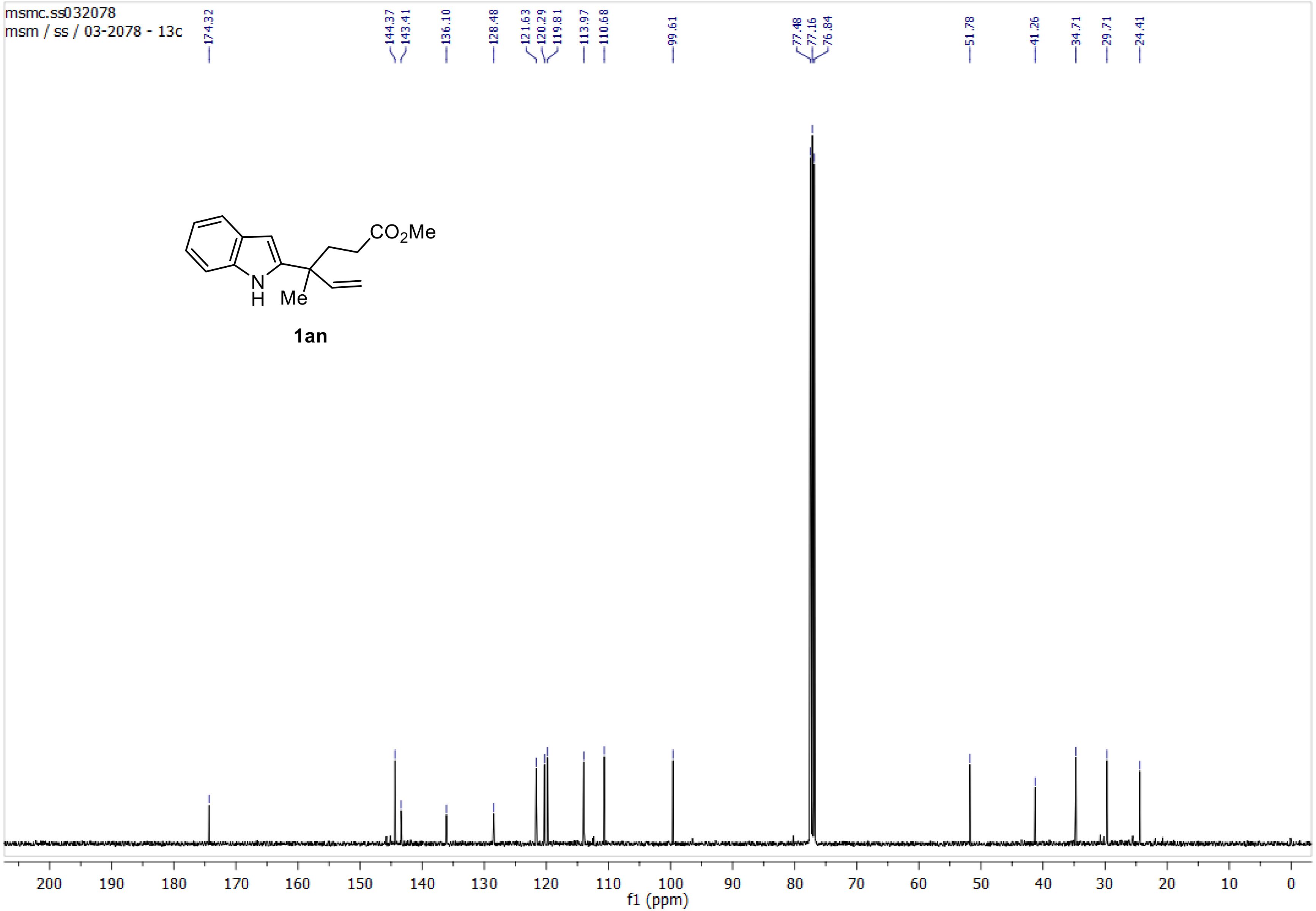

msmc.ss032078
msm / ss / 03-2078 - 13c
174.32
144.37
143.41
136.10
128.48
121.63
120.29
119.81
113.97
110.68
99.61
77.48
77.16
76.84
51.78
41.26
34.71
29.71
24.41
CO2Me
N
H
Me
1an
200 190 180 170 160 150 140 130 120 110 100 90 80 70 60 50 40 30 20 10 0
f1 (ppm)

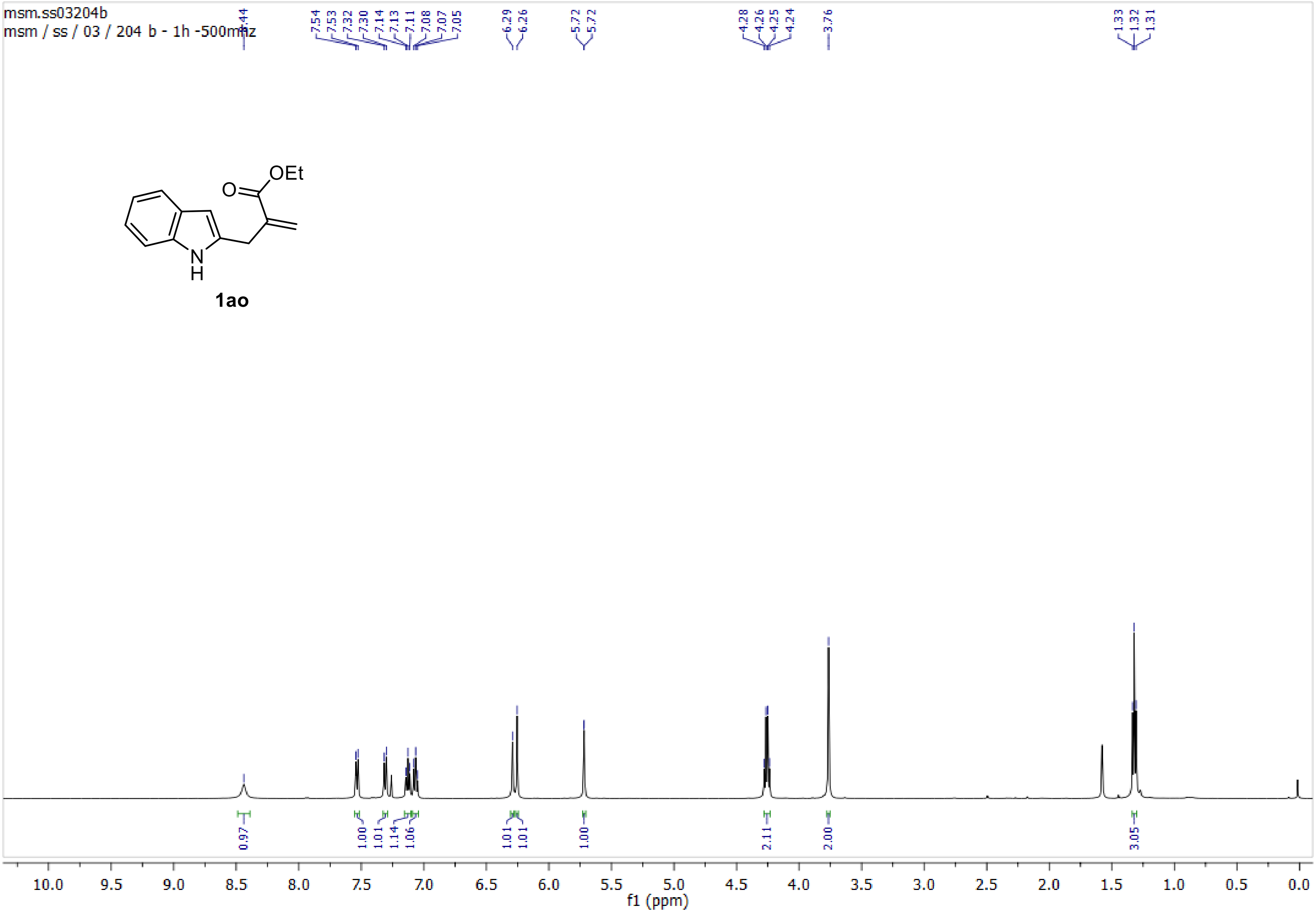
msm.ss03204b
msm / ss / 03 / 204 b - 1h -500mhz
OEt
O
N
H
1ao
8.44
7.54 7.53 7.32 7.30 7.14 7.13 7.11 7.08 7.07 7.05
6.29 6.26
5.72 5.72
4.28 4.26 4.25 4.24
3.76
1.33 1.32 1.31
0.97
1.00 1.01 1.14 1.06
1.01 1.01
1.00
2.11
2.00
3.05
10.0 9.5 9.0 8.5 8.0 7.5 7.0 6.5 6.0 5.5 5.0 4.5 4.0 3.5 3.0 2.5 2.0 1.5 1.0 0.5 0.0
f1 (ppm)

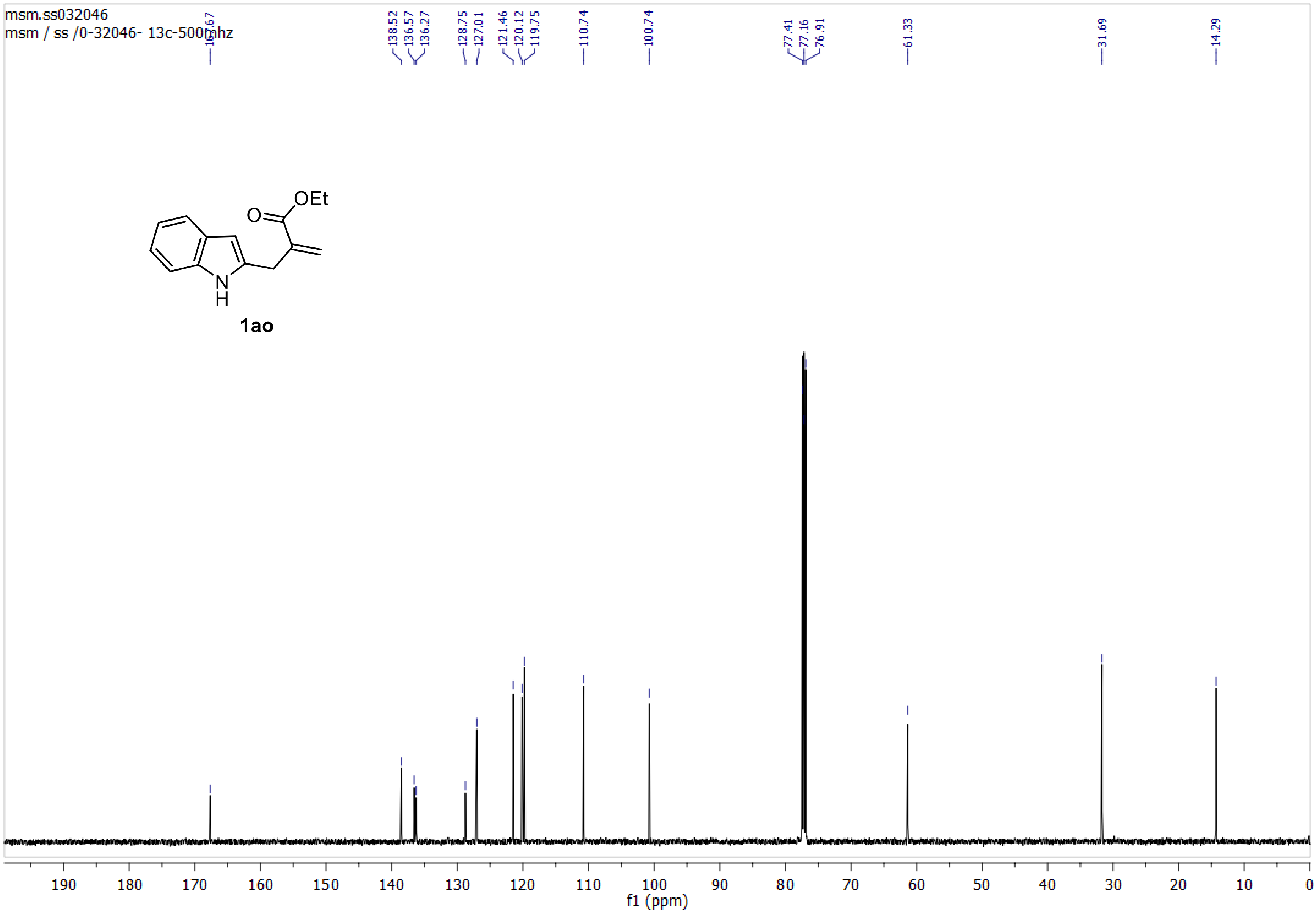
msm.ss032046
msm / ss /0-32046- 13c-500mhz
138.52
136.57
136.27
128.75
127.01
121.46
120.12
119.75
110.74
100.74
77.41
77.16
76.91
61.33
31.69
14.29
OEt
O
N
H
1ao
190
180
170
160
150
140
130
120
110
100
90
80
70
60
50
40
30
20
10
0
f1 (ppm)

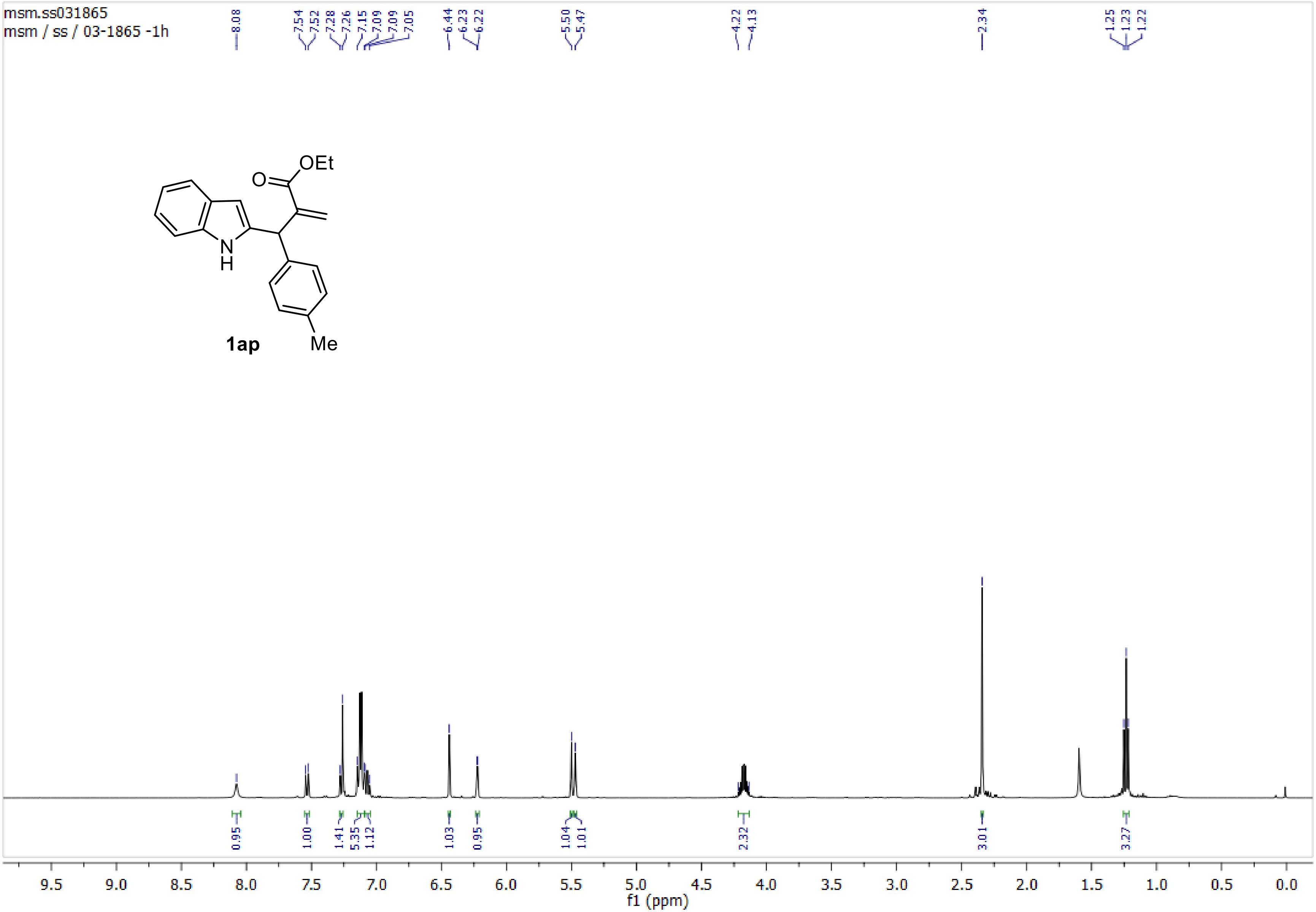
msm.ss031865
msm / ss / 03-1865 -1h
OEt
O
N
H
Me
1ap
8.08
7.54
7.52
7.28
7.26
7.15
7.09
7.09
7.05
6.44
6.23
6.22
5.50
5.47
4.22
4.13
2.34
1.25
1.23
1.22
0.95
1.00
1.41
5.35
1.12
1.03
0.95
1.04
1.01
2.32
3.01
3.27
9.5
9.0
8.5
8.0
7.5
7.0
6.5
6.0
5.5
5.0
4.5
4.0
3.5
3.0
2.5
2.0
1.5
1.0
0.5
0.0
f1 (ppm)

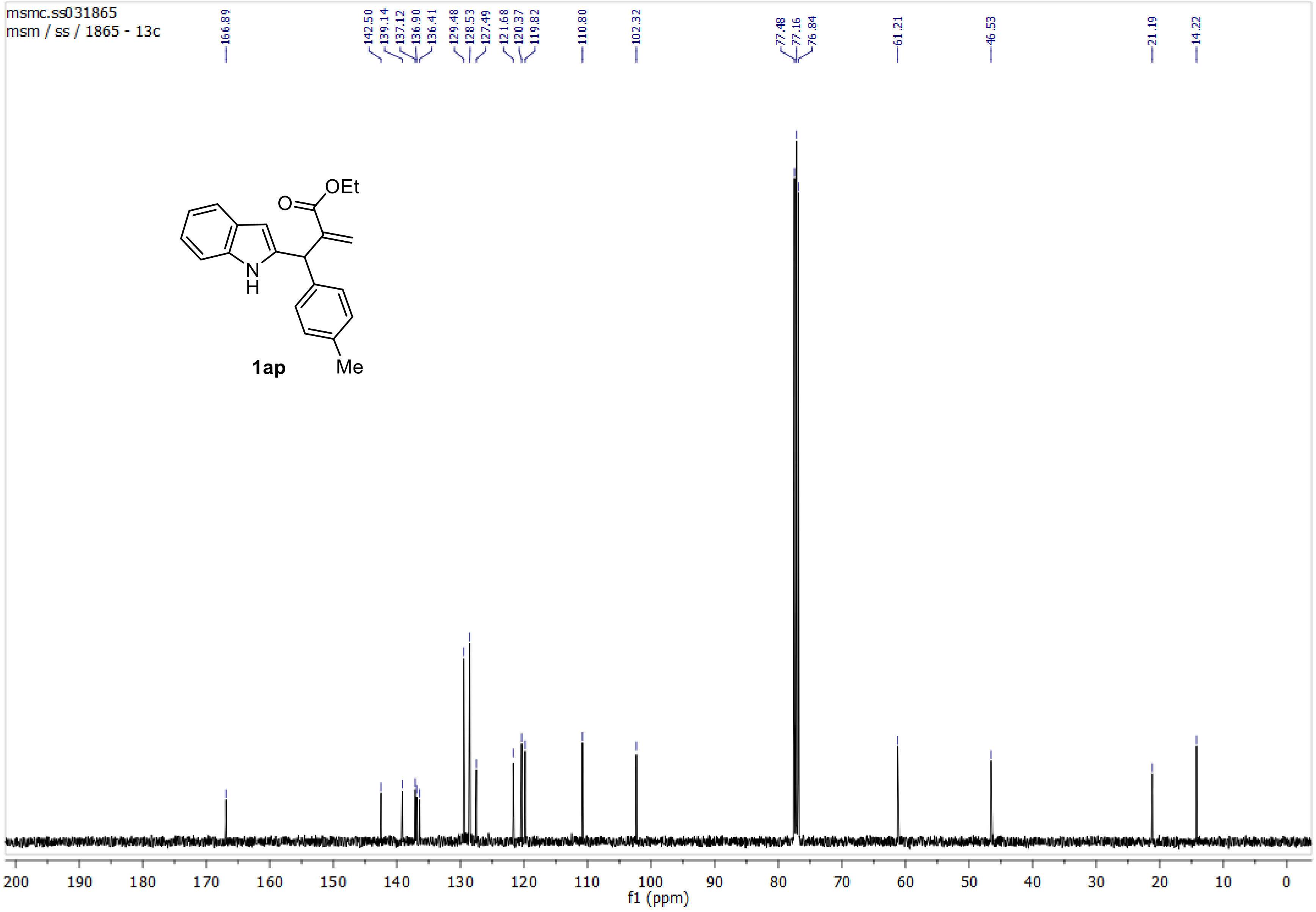

msmc.ss031865
msm / ss / 1865 - 13c
166.89
142.50
139.14
137.12
136.90
136.41
129.48
128.53
127.49
121.68
120.37
119.82
110.80
102.32
77.48
77.16
76.84
61.21
46.53
21.19
14.22
OEt
O
N
H
Me
1ap
200 190 180 170 160 150 140 130 120 110 100 90 80 70 60 50 40 30 20 10 0
f1 (ppm)

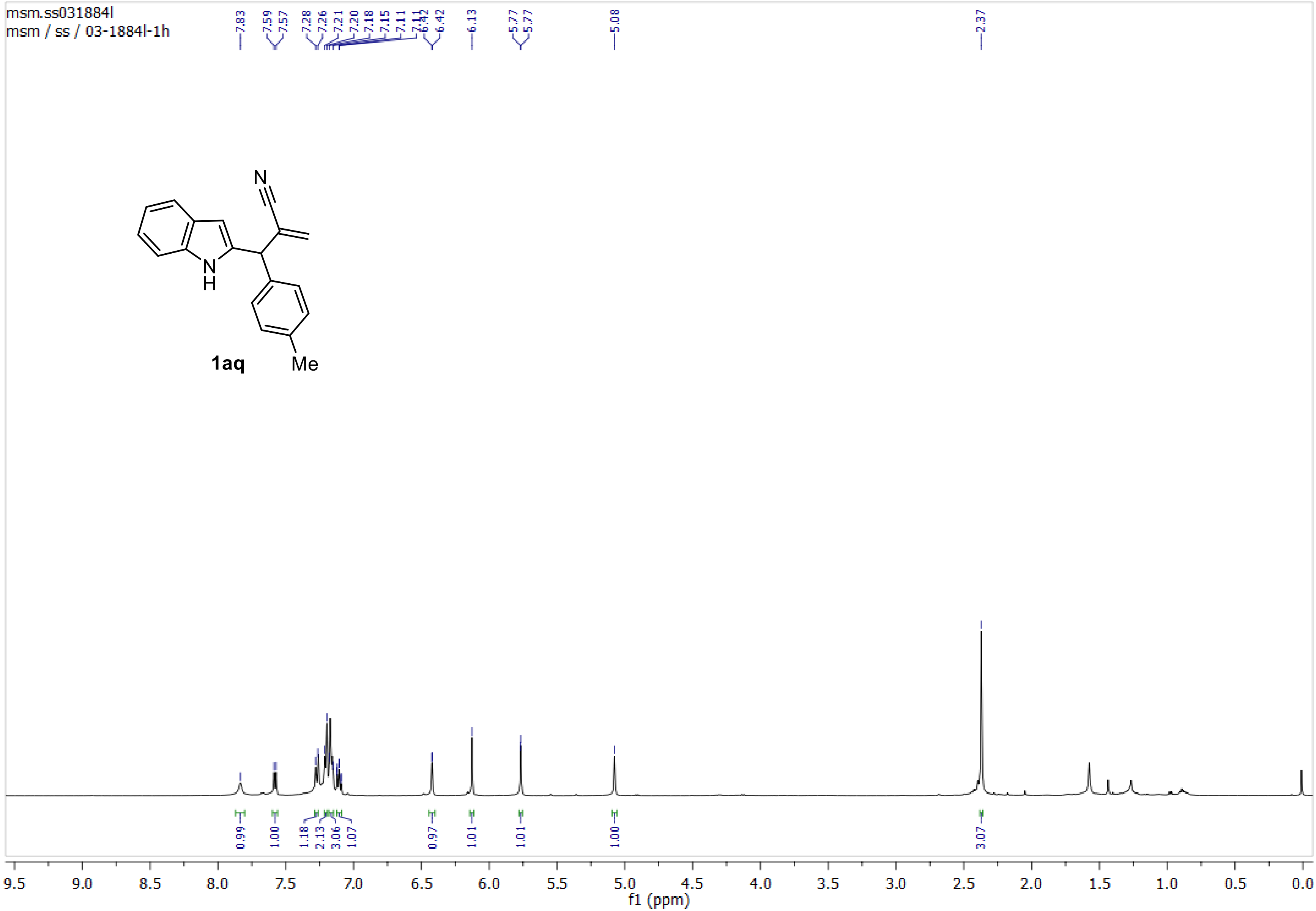
msm.ss031884l
msm / ss / 03-1884l-1h
7.83
7.59
7.57
7.28
7.26
7.21
7.20
7.18
7.15
7.11
7.11
6.42
6.42
6.13
5.77
5.77
5.08
2.37
N
N
H
Me
1aq
0.99
1.00
1.18
2.13
3.06
1.07
0.97
1.01
1.01
1.00
3.07
9.5
9.0
8.5
8.0
7.5
7.0
6.5
6.0
5.5
5.0
4.5
4.0
3.5
3.0
2.5
2.0
1.5
1.0
0.5
0.0
f1 (ppm)

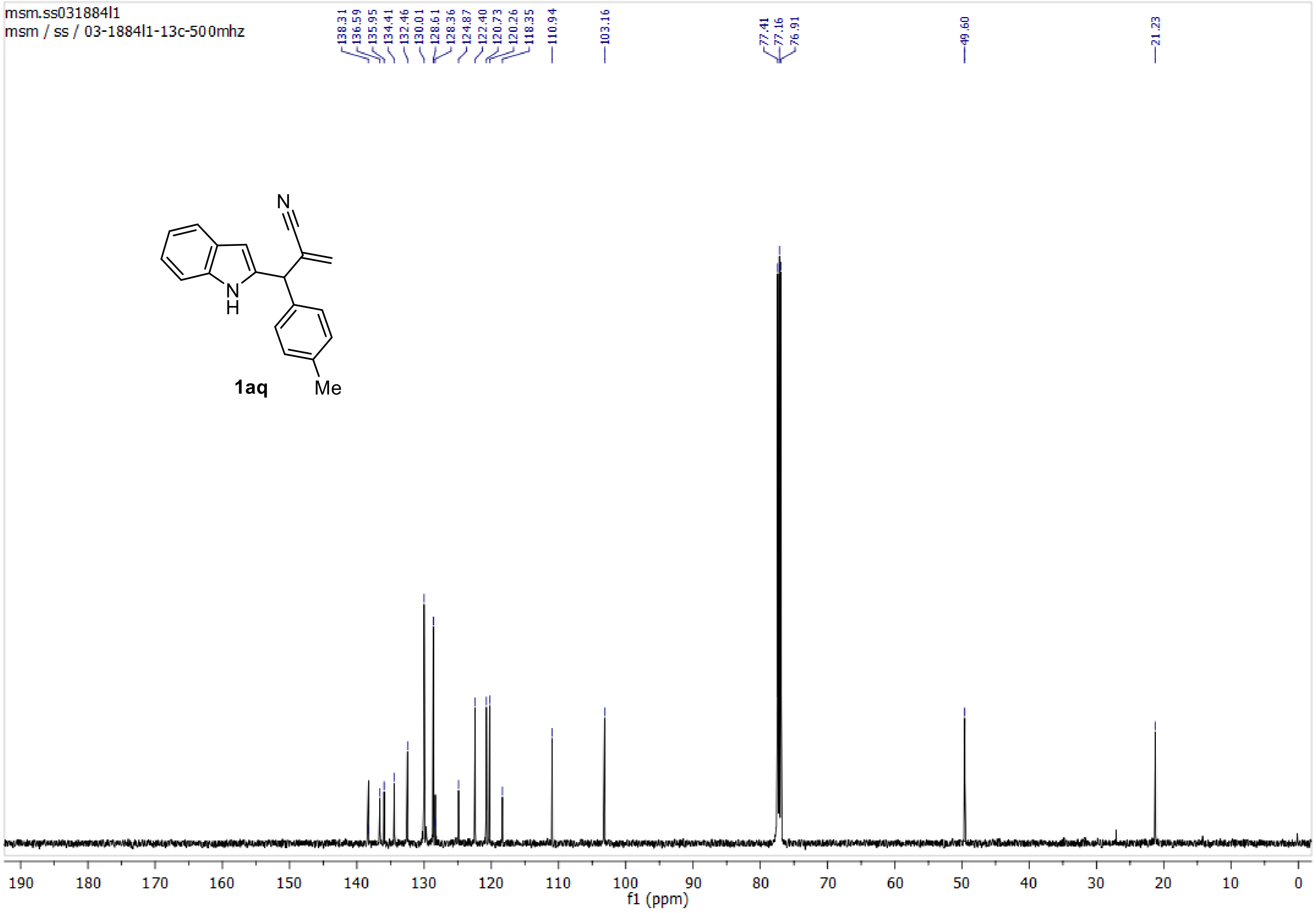
msm.ss031884l1
msm / ss / 03-1884l1-13c-500mhz
138.31
136.59
135.95
134.41
132.46
130.01
128.61
128.36
124.87
122.40
120.73
120.26
118.35
110.94
103.16
77.41
77.16
76.91
49.60
21.23
N
N
H
1aq
Me
190
180
170
160
150
140
130
120
110
100
90
80
70
60
50
40
30
20
10
0
f1 (ppm)

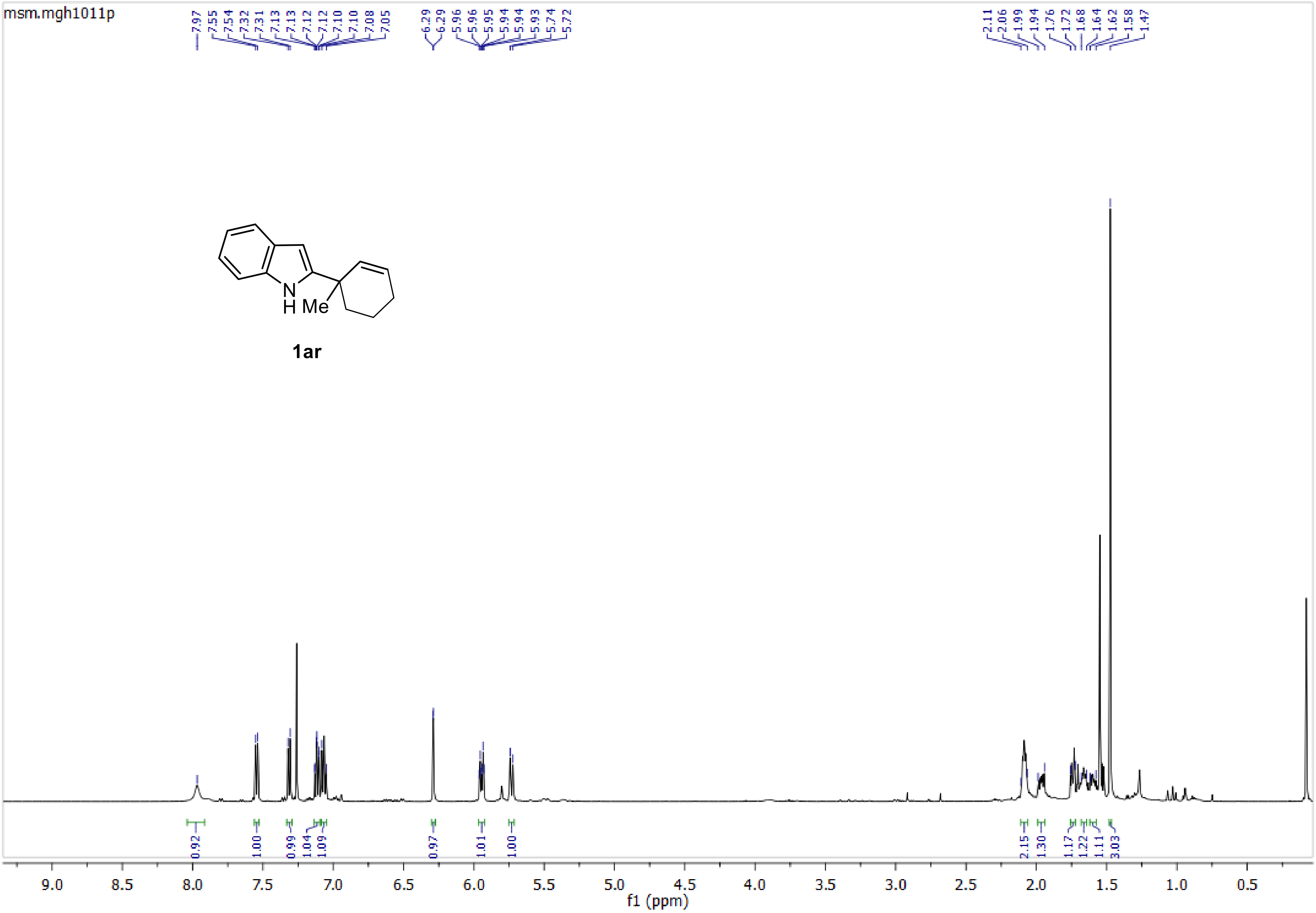
msm.mgh1011p
7.97 7.55 7.54 7.32 7.31 7.13 7.13 7.12 7.12 7.10 7.10 7.08 7.05
6.29 6.29 5.96 5.96 5.95 5.94 5.94 5.93 5.74 5.72
2.11 2.06 1.99 1.94 1.76 1.72 1.68 1.64 1.62 1.58 1.47
N
H Me
1ar
0.92 1.00 0.99 1.04 1.09 0.97 1.01 1.00
2.15 1.30 1.17 1.22 1.11 3.03
9.0 8.5 8.0 7.5 7.0 6.5 6.0 5.5 5.0 4.5 4.0 3.5 3.0 2.5 2.0 1.5 1.0 0.5
f1 (ppm)

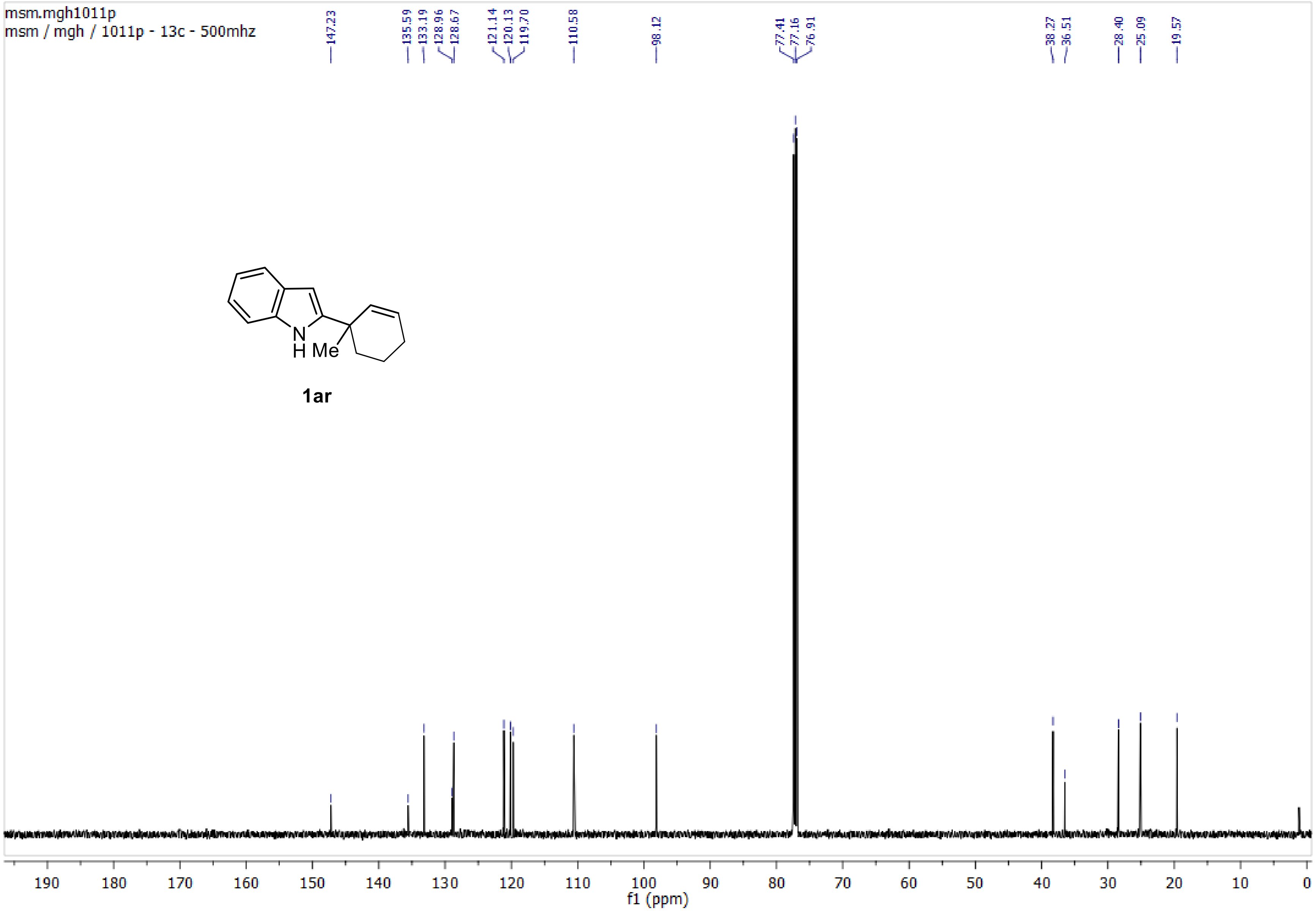
msm.mgh1011p
msm / mgh / 1011p - 13c - 500mhz
147.23
135.59
133.19
128.96
128.67
121.14
120.13
119.70
110.58
98.12
77.41
77.16
76.91
38.27
36.51
28.40
25.09
19.57
N
H Me
1ar
190 180 170 160 150 140 130 120 110 100 90 80 70 60 50 40 30 20 10 0
f1 (ppm)

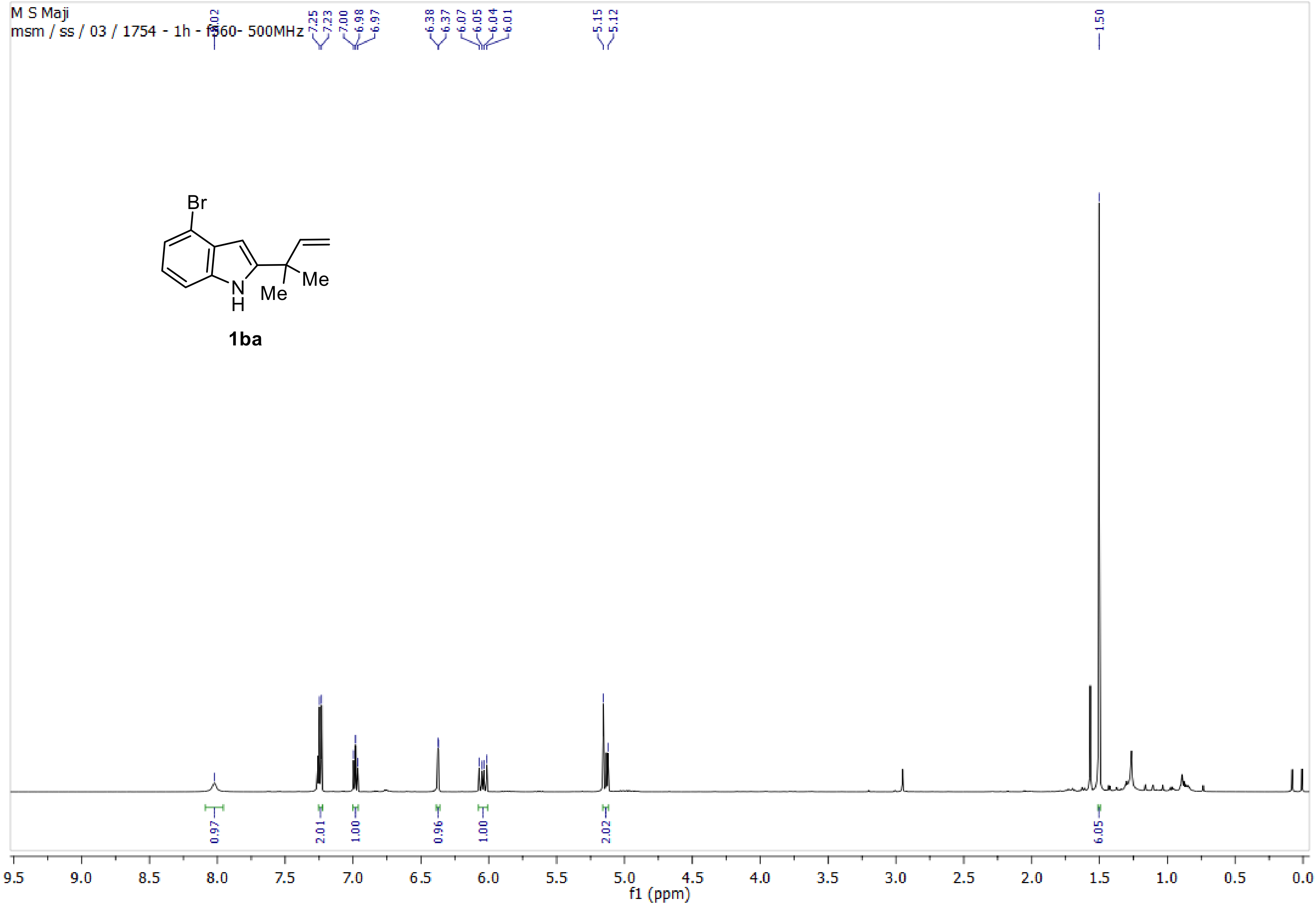
M S Maji
msm / ss / 03 / 1754 - 1h - f360- 500MHz
8.02
7.25
7.23
7.00
6.98
6.97
6.38
6.37
6.07
6.05
6.04
6.01
5.15
5.12
1.50
Br
N
H
Me
Me
1ba
0.97
2.01
1.00
0.96
1.00
2.02
6.05
9.5
9.0
8.5
8.0
7.5
7.0
6.5
6.0
5.5
5.0
4.5
4.0
3.5
3.0
2.5
2.0
1.5
1.0
0.5
0.0
f1 (ppm)

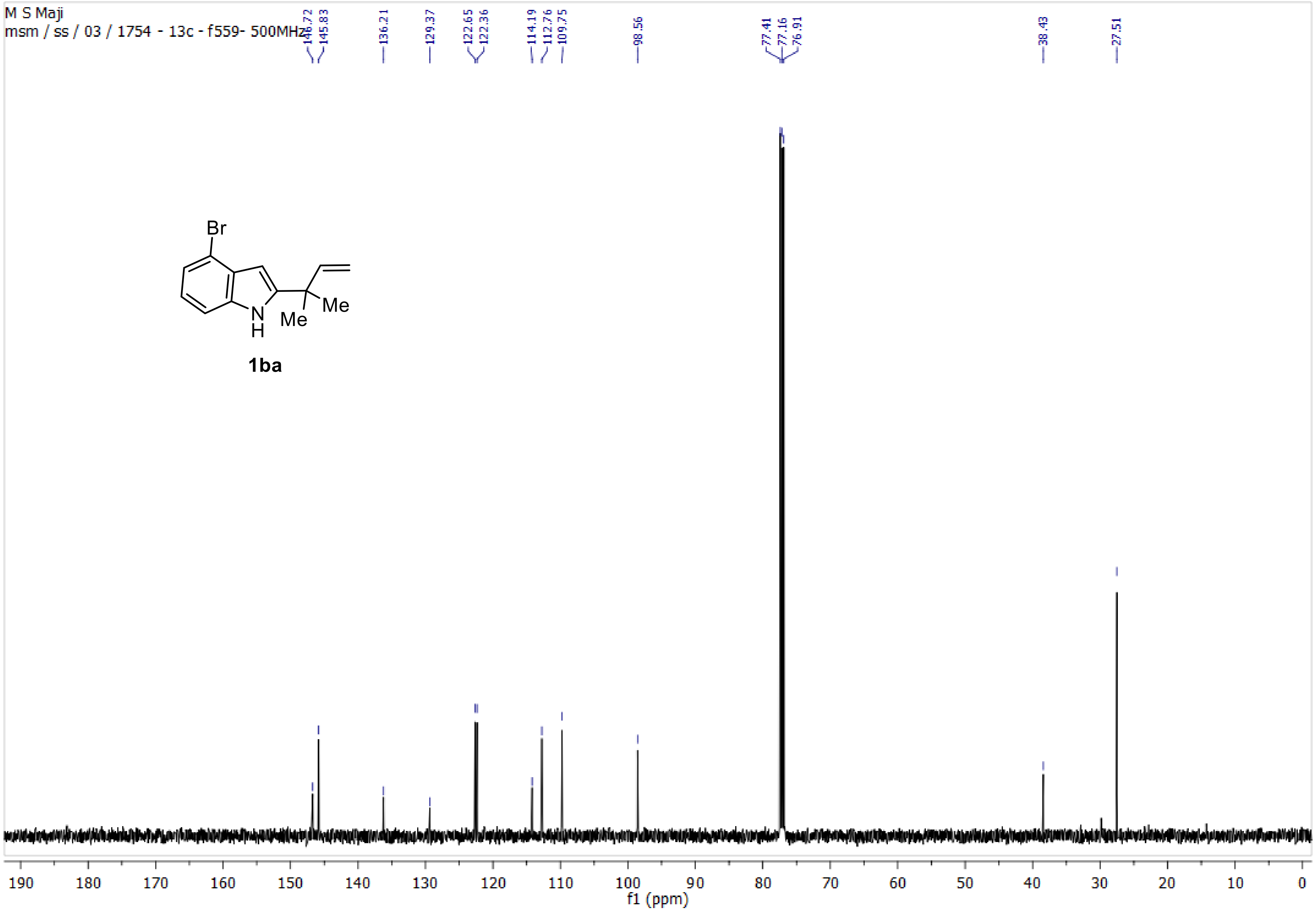
M S Maji
msm / ss / 03 / 1754 - 13c - f559- 500MHz
146.72
145.83
136.21
129.37
122.65
122.36
114.19
112.76
109.75
98.56
77.41
77.16
76.91
38.43
27.51
Br
N
H
Me
Me
1ba
190 180 170 160 150 140 130 120 110 100 90 80 70 60 50 40 30 20 10 0
f1 (ppm)

msm.ss032303.2.fid
msm / ss / 03 - 2203 - 1h - 500mhz

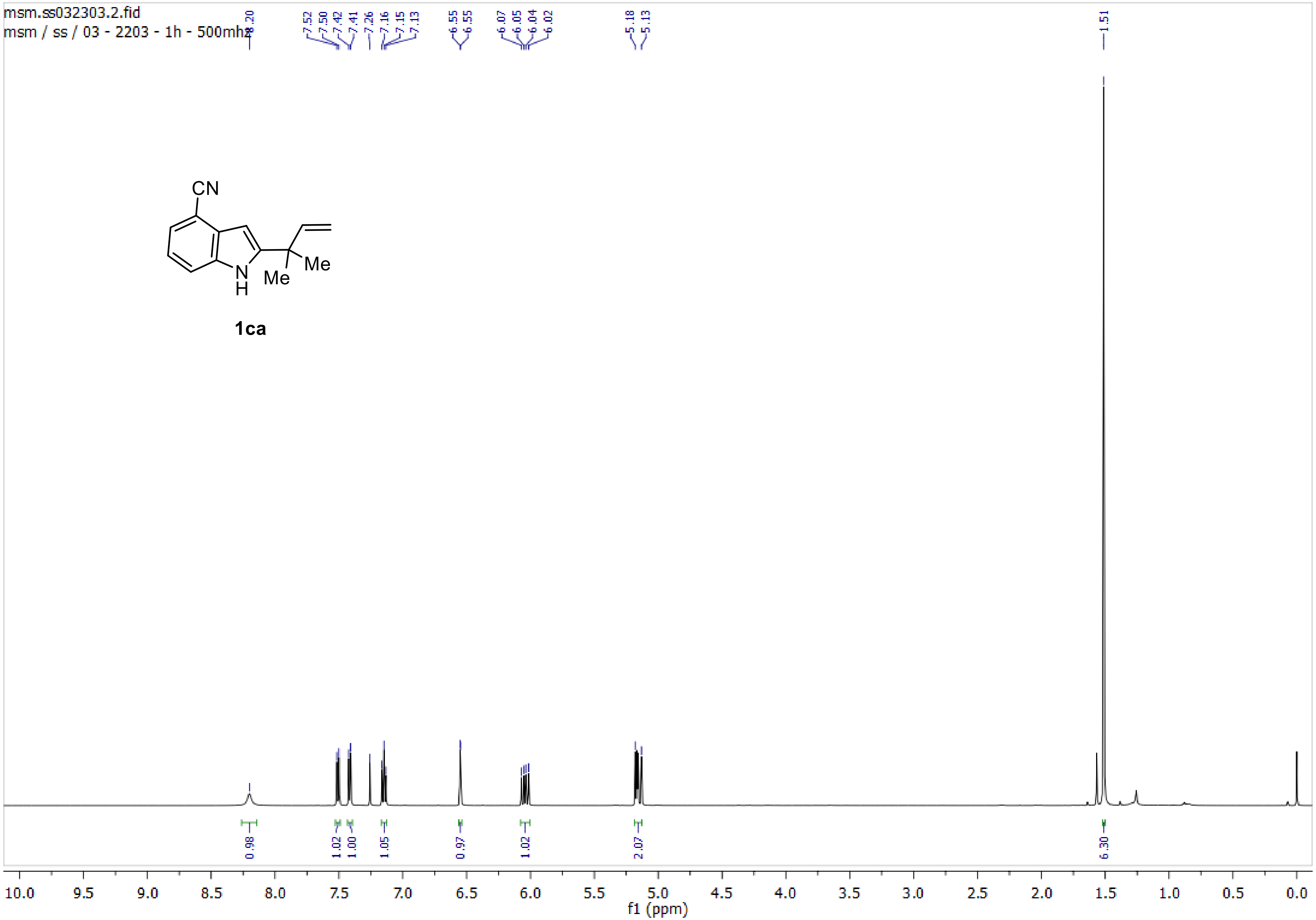

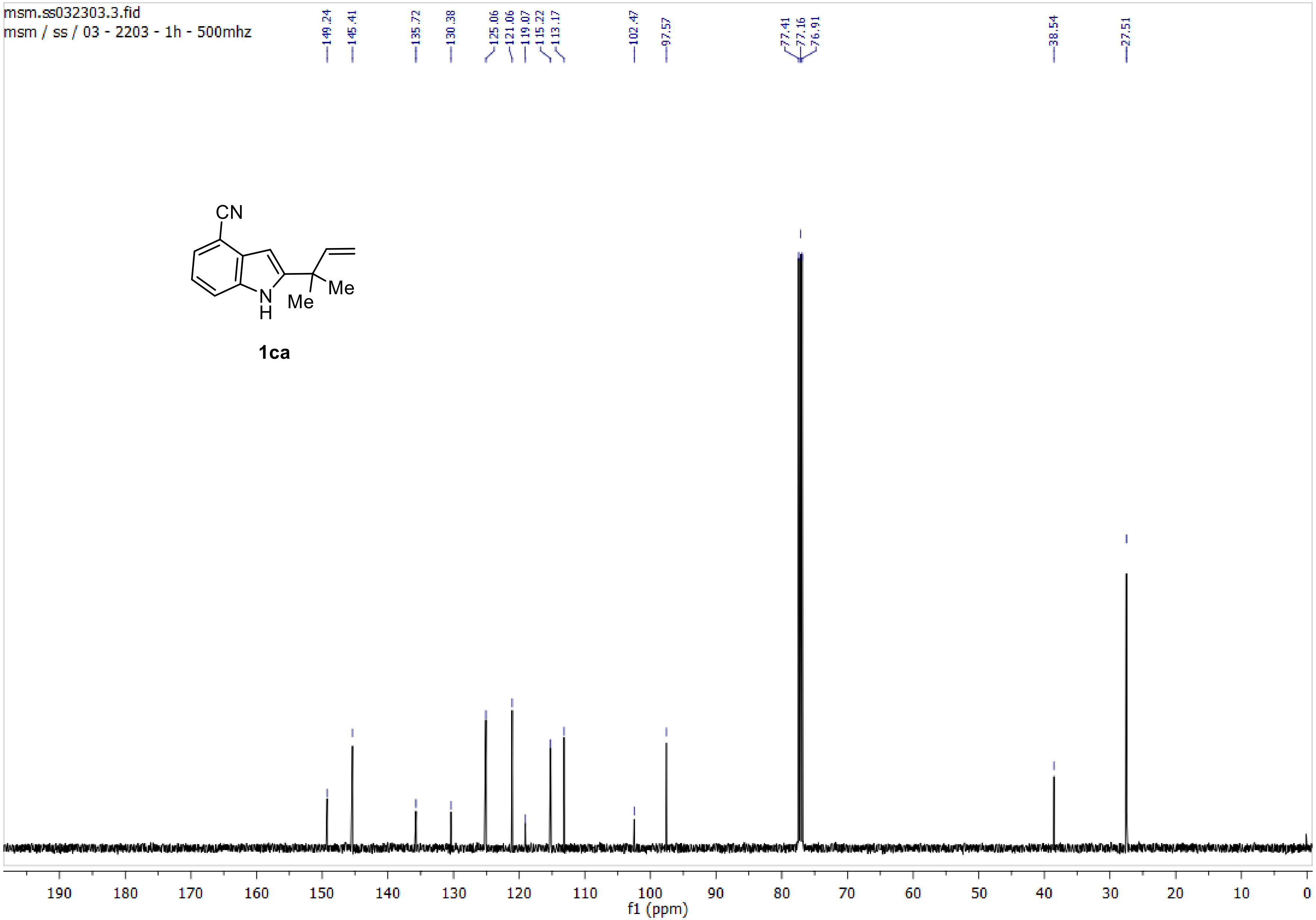
msm.ss032303.3.fid
msm / ss / 03 - 2203 - 1h - 500mhz
149.24
145.41
135.72
130.38
125.06
121.06
119.07
115.22
113.17
102.47
97.57
77.41
77.16
76.91
38.54
27.51
CN
N
H
Me
Me
190
180
170
160
150
140
130
120
110
100
90
80
70
60
50
40
30
20
10
0
f1 (ppm)

1ca

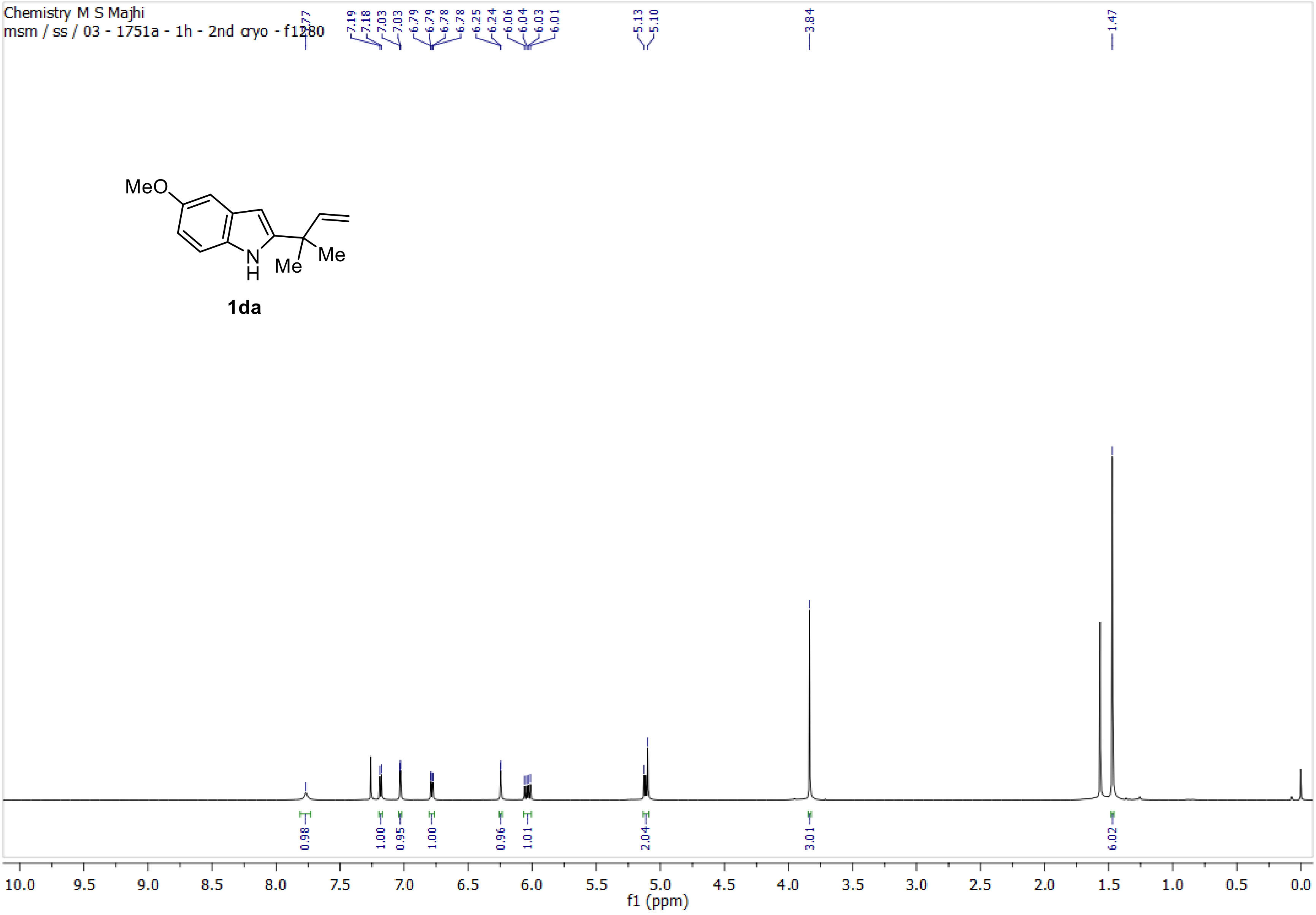
Chemistry M S Majhi
msm / ss / 03 - 1751a - 1h - 2nd cryo - f1280
7.77
7.19
7.18
7.03
7.03
6.79
6.79
6.78
6.78
6.25
6.24
6.06
6.04
6.03
6.01
5.13
5.10
3.84
1.47
MeO
N
H
Me
Me
1da
0.98
1.00
0.95
1.00
0.96
1.01
2.04
3.01
6.02
10.0 9.5 9.0 8.5 8.0 7.5 7.0 6.5 6.0 5.5 5.0 4.5 4.0 3.5 3.0 2.5 2.0 1.5 1.0 0.5 0.0
f1 (ppm)

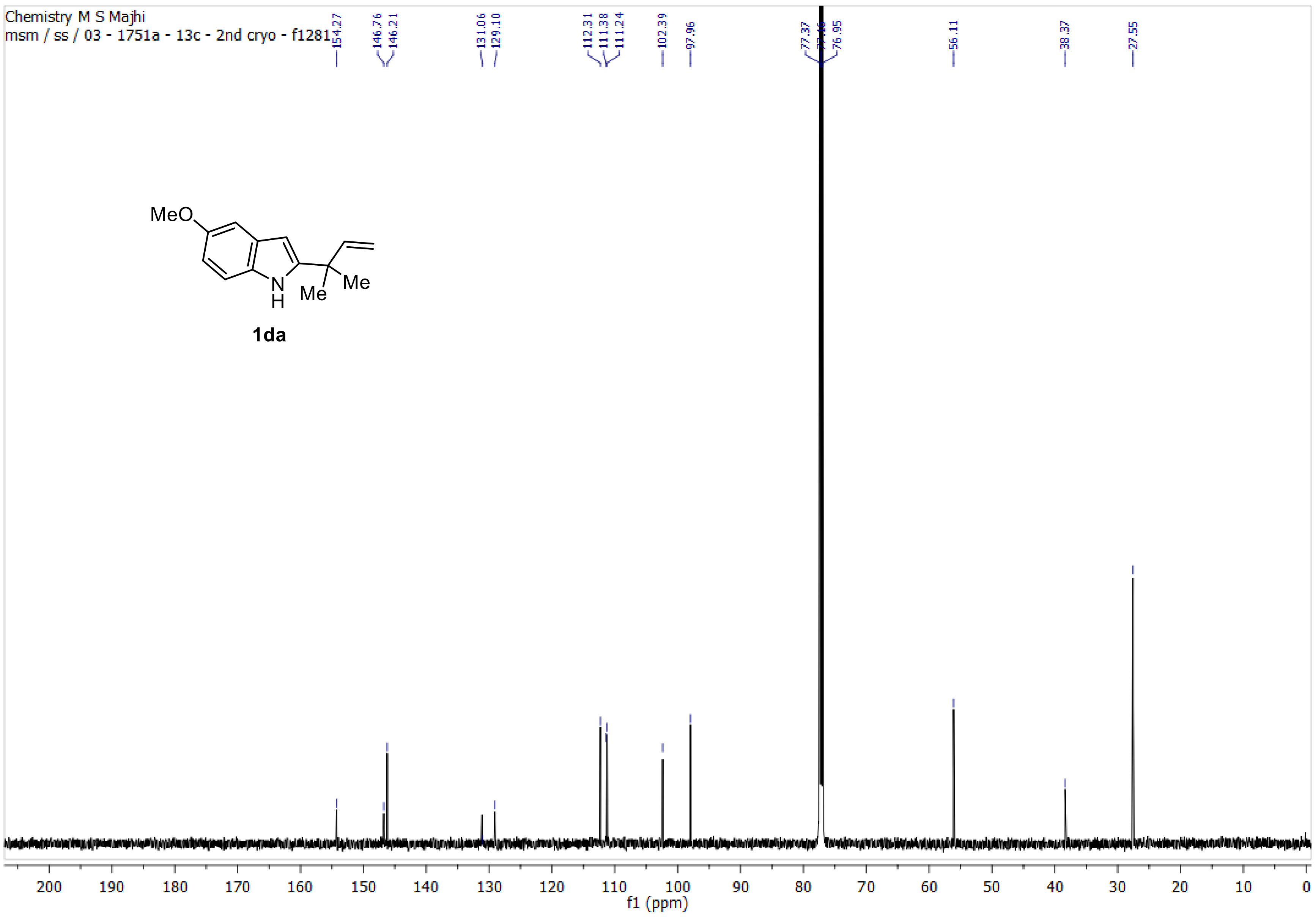

Chemistry M S Majhi
msm / ss / 03 - 1751a - 13c - 2nd cryo - f1281
154.27
146.76
146.21
131.06
129.10
112.31
111.38
111.24
102.39
97.96
77.37
76.95
56.11
38.37
27.55
MeO
N
H
Me
Me
1da
200 190 180 170 160 150 140 130 120 110 100 90 80 70 60 50 40 30 20 10 0
f1 (ppm)

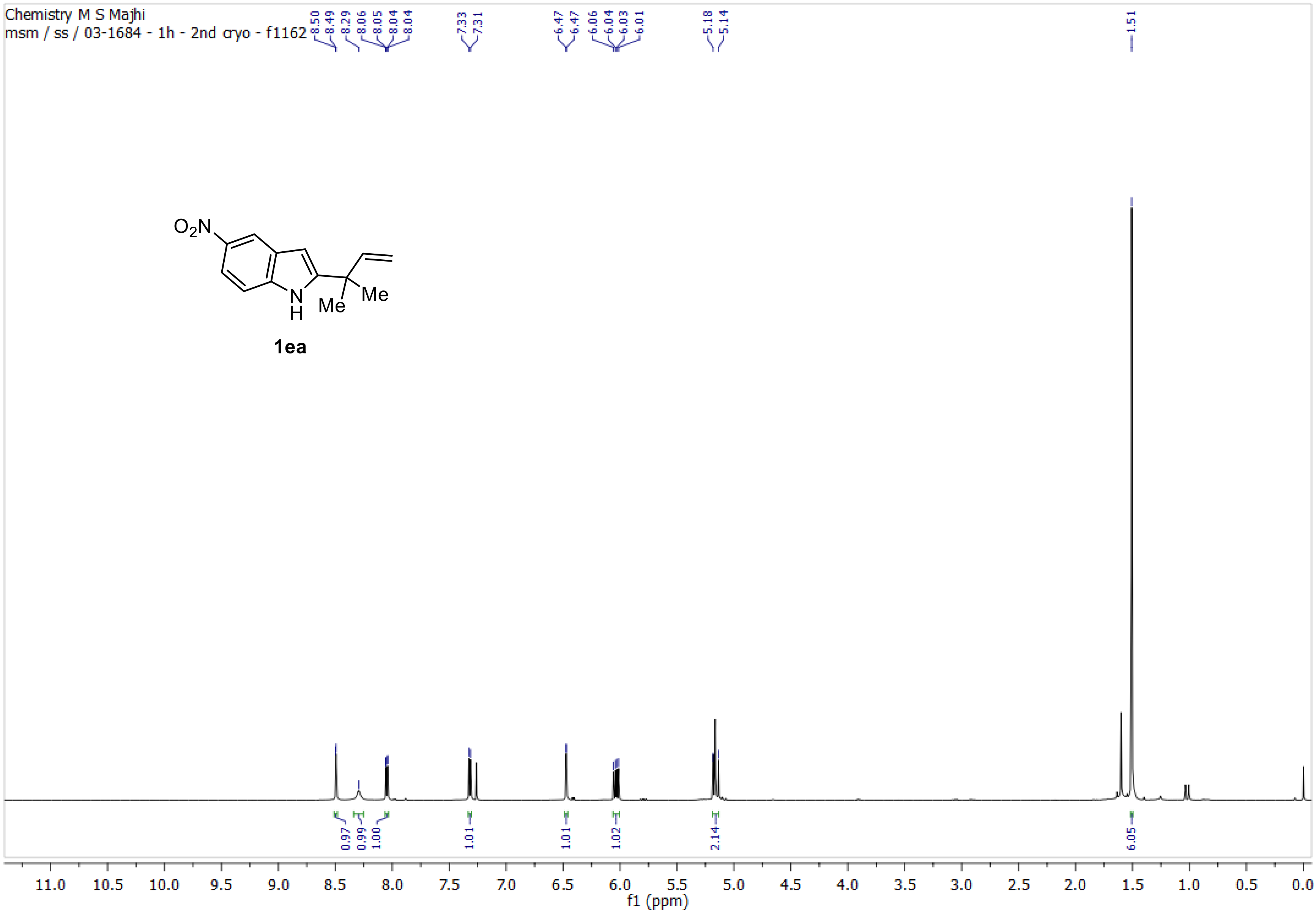
Chemistry M S Majhi
msm / ss / 03-1684 - 1h - 2nd cryo - f1162
8.50
8.49
8.29
8.06
8.05
8.04
8.04
7.33
7.31
6.47
6.47
6.06
6.04
6.03
6.01
5.18
5.14
1.51
O_2N
N
H
Me
Me
1ea
0.97
0.99
1.00
1.01
1.01
1.02
2.14
6.05
11.0
10.5
10.0
9.5
9.0
8.5
8.0
7.5
7.0
6.5
6.0
5.5
5.0
4.5
4.0
3.5
3.0
2.5
2.0
1.5
1.0
0.5
0.0
f1 (ppm)

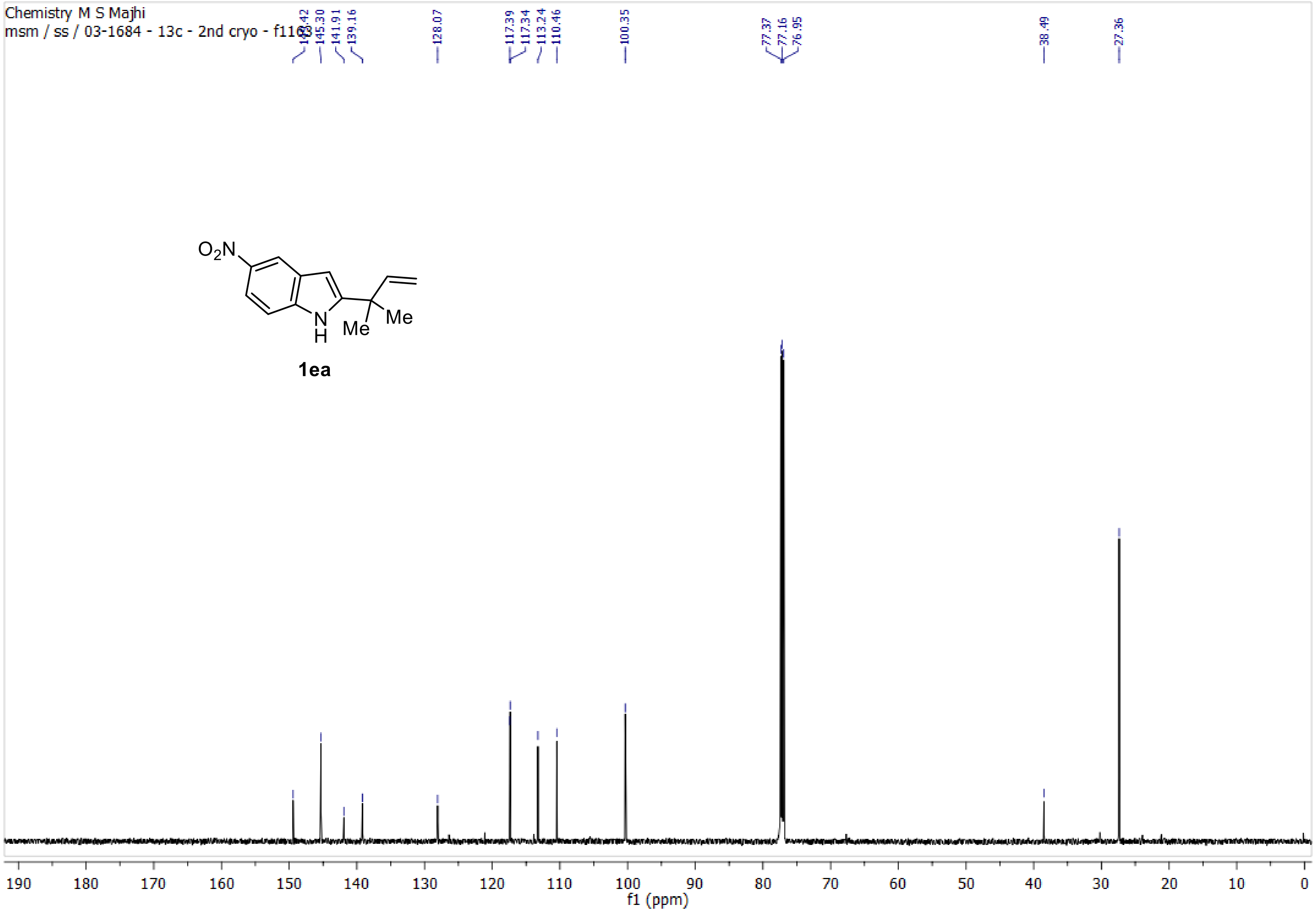
Chemistry M S Majhi
msm / ss / 03-1684 - 13c - 2nd cryo - f1163
149.42
145.30
141.91
139.16
128.07
117.39
117.34
113.24
110.46
100.35
77.37
77.16
76.95
38.49
27.36
O2N
N
H
Me
Me
190 180 170 160 150 140 130 120 110 100 90 80 70 60 50 40 30 20 10 0
f1 (ppm)

1ea

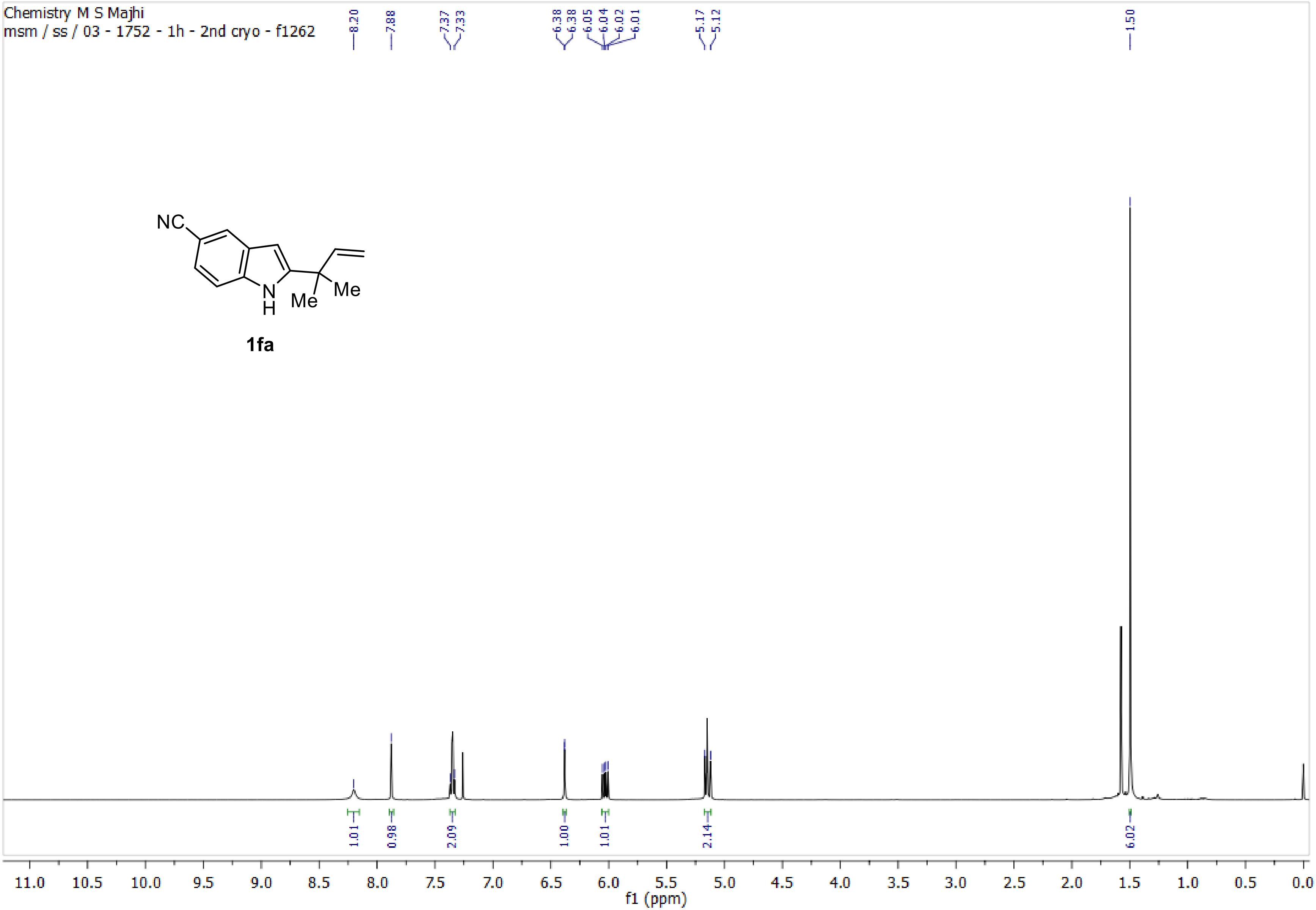
Chemistry M S Majhi
msm / ss / 03 - 1752 - 1h - 2nd cryo - f1262
8.20
7.88
7.37
7.33
6.38
6.38
6.05
6.04
6.02
6.01
5.17
5.12
1.50
NC
N
H
Me
Me
1fa
1.01
0.98
2.09
1.00
1.01
2.14
6.02
11.0
10.5
10.0
9.5
9.0
8.5
8.0
7.5
7.0
6.5
6.0
5.5
5.0
4.5
4.0
3.5
3.0
2.5
2.0
1.5
1.0
0.5
0.0
f1 (ppm)

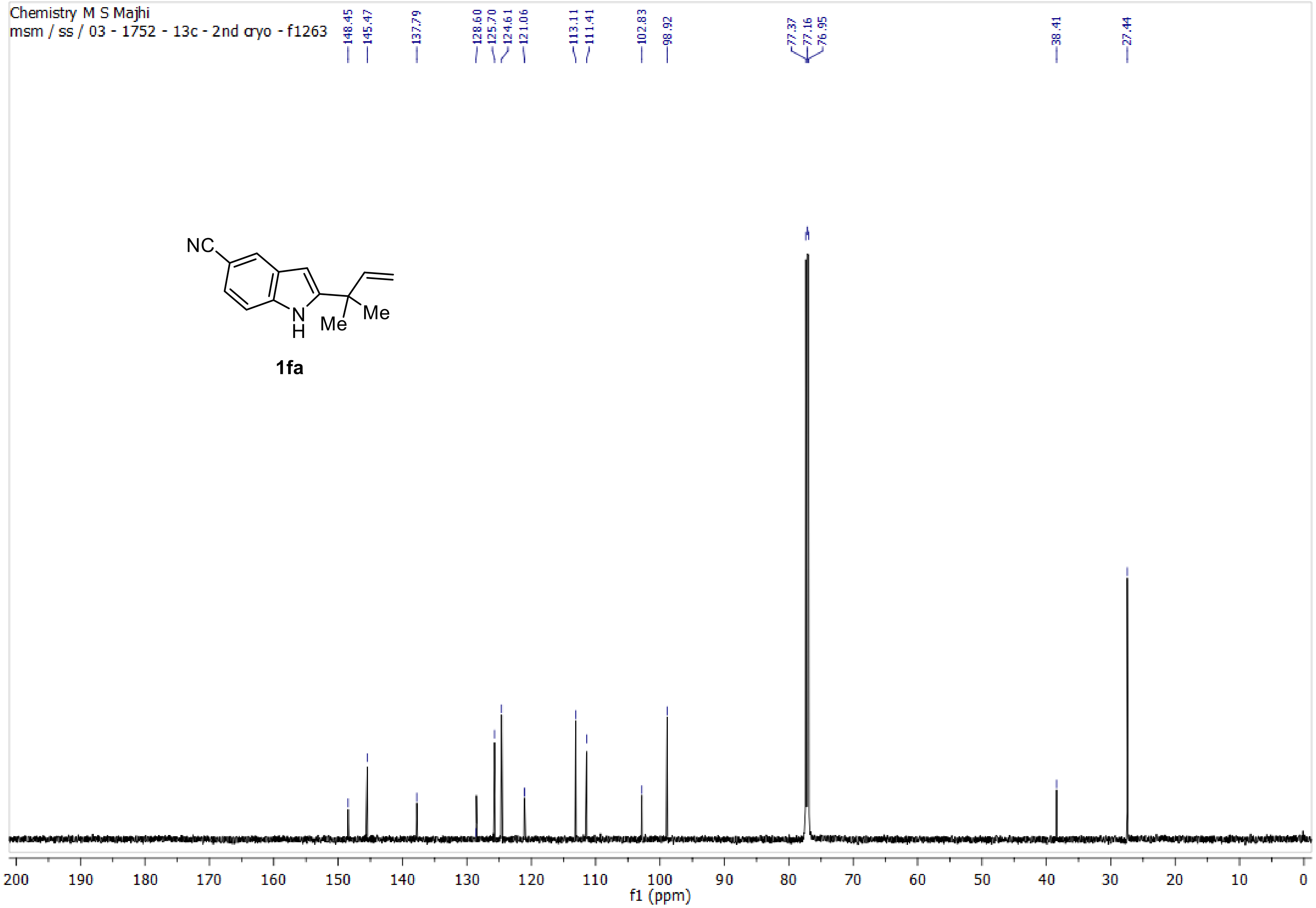
Chemistry M S Majhi
msm / ss / 03 - 1752 - 13c - 2nd cryo - f1263
148.45
145.47
137.79
128.60
125.70
124.61
121.06
113.11
111.41
102.83
98.92
77.37
77.16
76.95
38.41
27.44
NC
N
H
Me
Me
1fa
200 190 180 170 160 150 140 130 120 110 100 90 80 70 60 50 40 30 20 10 0
f1 (ppm)

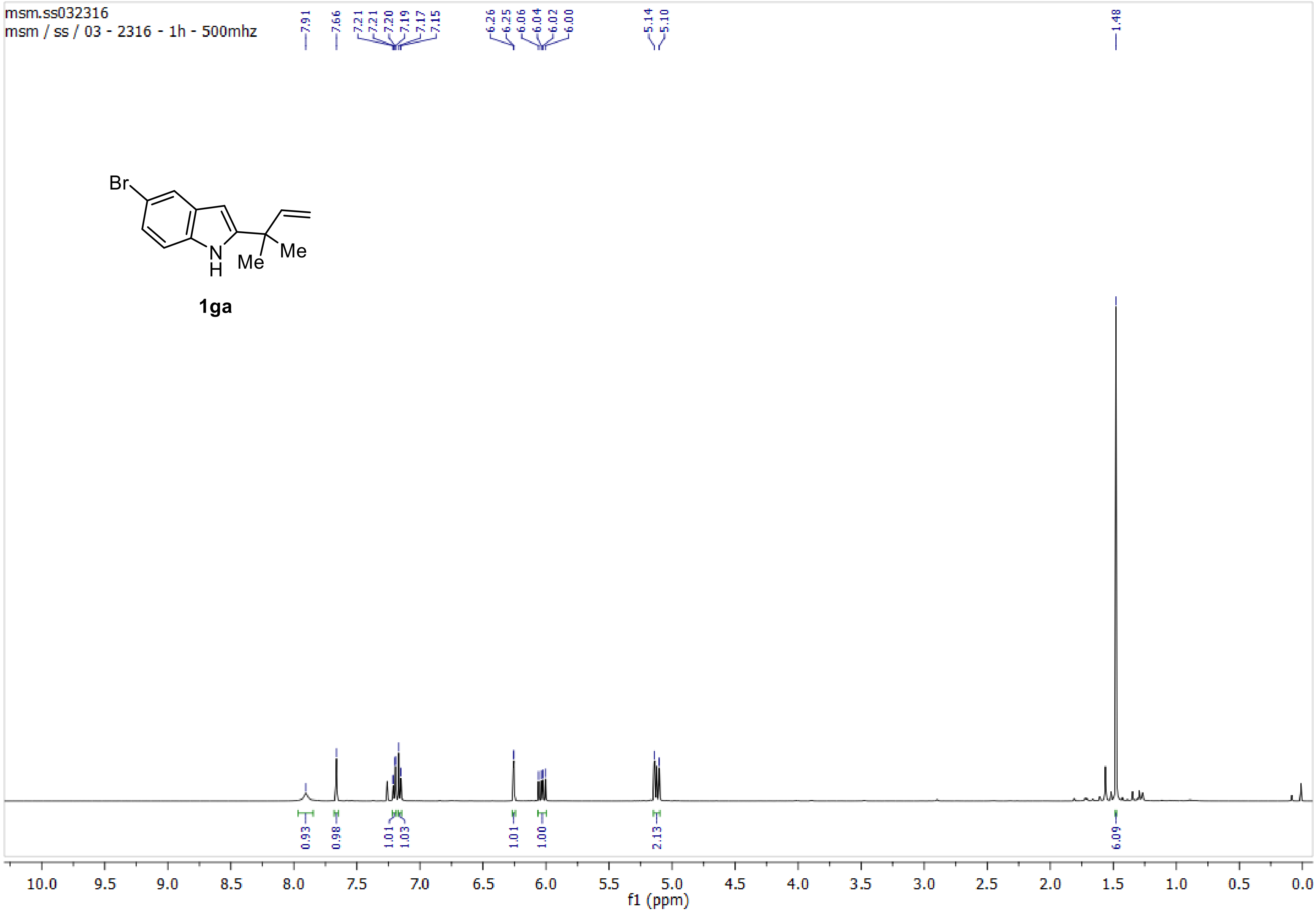
msm.ss032316
msm / ss / 03 - 2316 - 1h - 500mhz
7.91
7.66
7.21
7.21
7.20
7.19
7.17
7.15
6.26
6.25
6.06
6.04
6.02
6.00
5.14
5.10
1.48
Br
N
H
Me
Me
1ga
0.93
0.98
1.01
1.03
1.01
1.00
2.13
6.09
10.0
9.5
9.0
8.5
8.0
7.5
7.0
6.5
6.0
5.5
5.0
4.5
4.0
3.5
3.0
2.5
2.0
1.5
1.0
0.5
0.0
f1 (ppm)

msm.ss032316.3.fid
msm / ss / 03 - 2316 - 1h - 500mhz

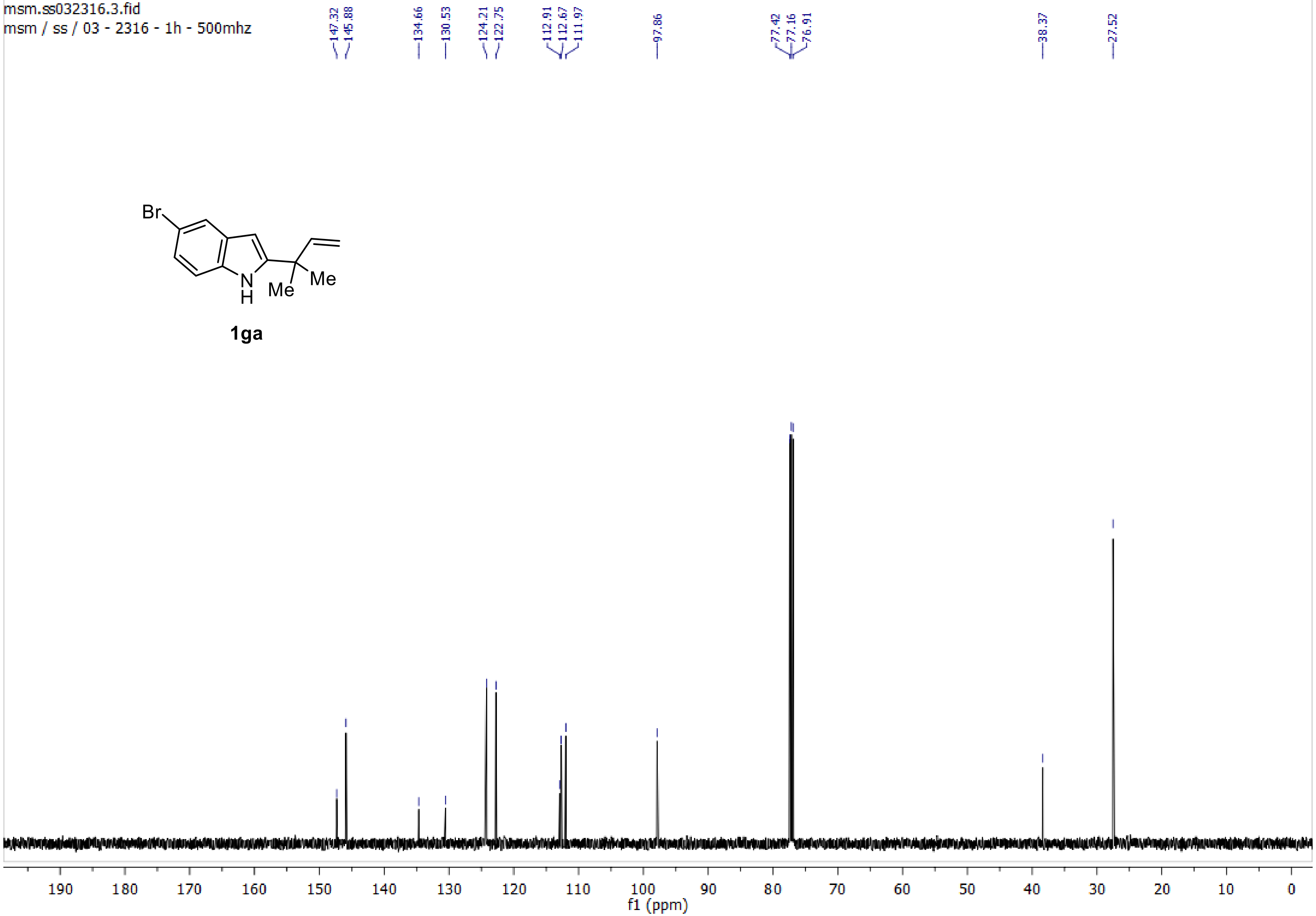

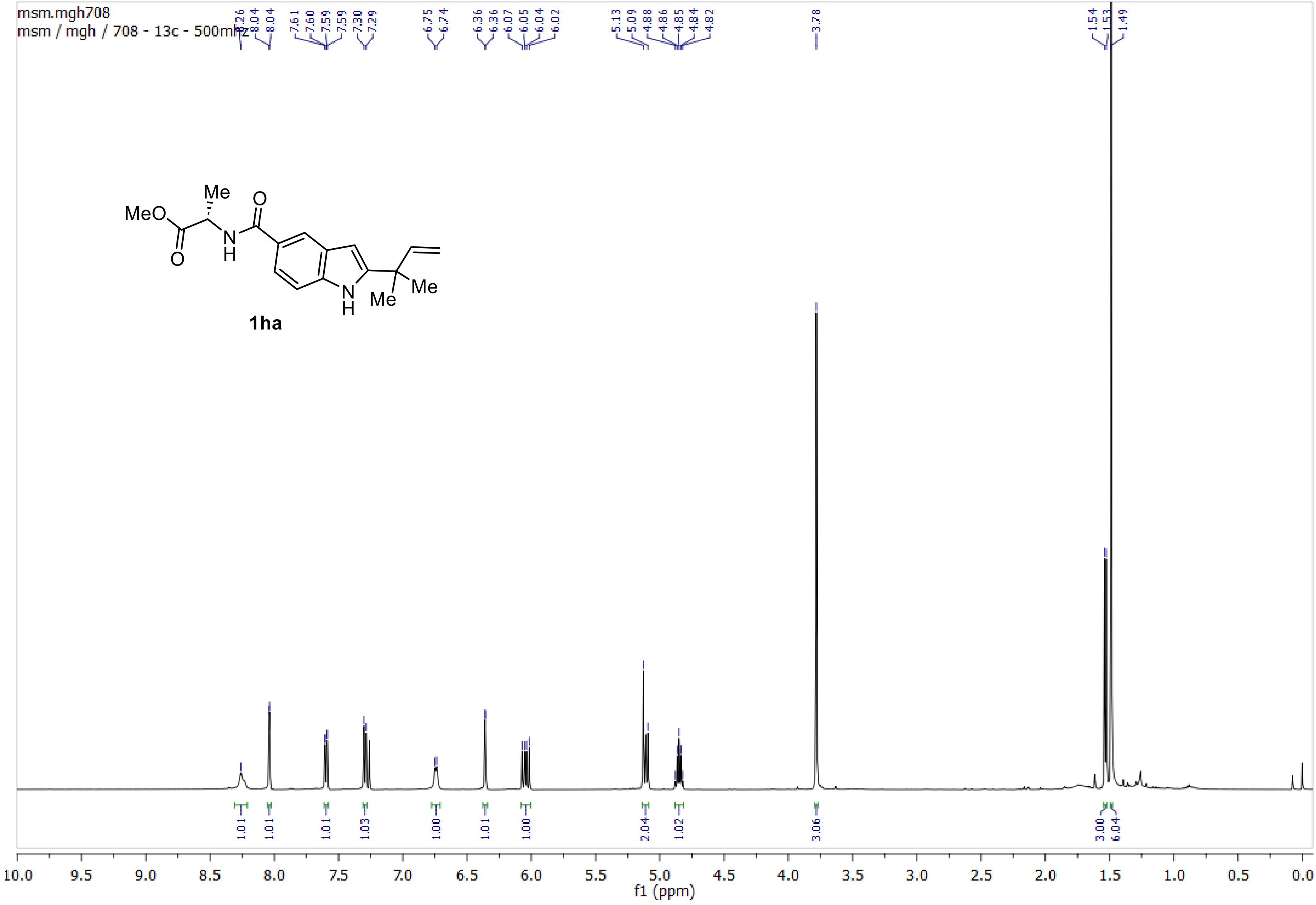
msm.mgh708
msm / mgh / 708 - 13c - 500mhz
8.26
8.04
8.04
7.61
7.60
7.59
7.59
7.30
7.29
6.75
6.74
6.36
6.36
6.07
6.05
6.04
6.02
5.13
5.09
4.88
4.86
4.85
4.84
4.82
3.78
1.54
1.53
1.49
MeO
Me
O
N
H
O
N
H
Me
Me
1ha
1.01
1.01
1.01
1.03
1.00
1.01
1.00
2.04
1.02
3.06
3.00
6.04
10.0
9.5
9.0
8.5
8.0
7.5
7.0
6.5
6.0
5.5
5.0
4.5
4.0
3.5
3.0
2.5
2.0
1.5
1.0
0.5
0.0
f1 (ppm)

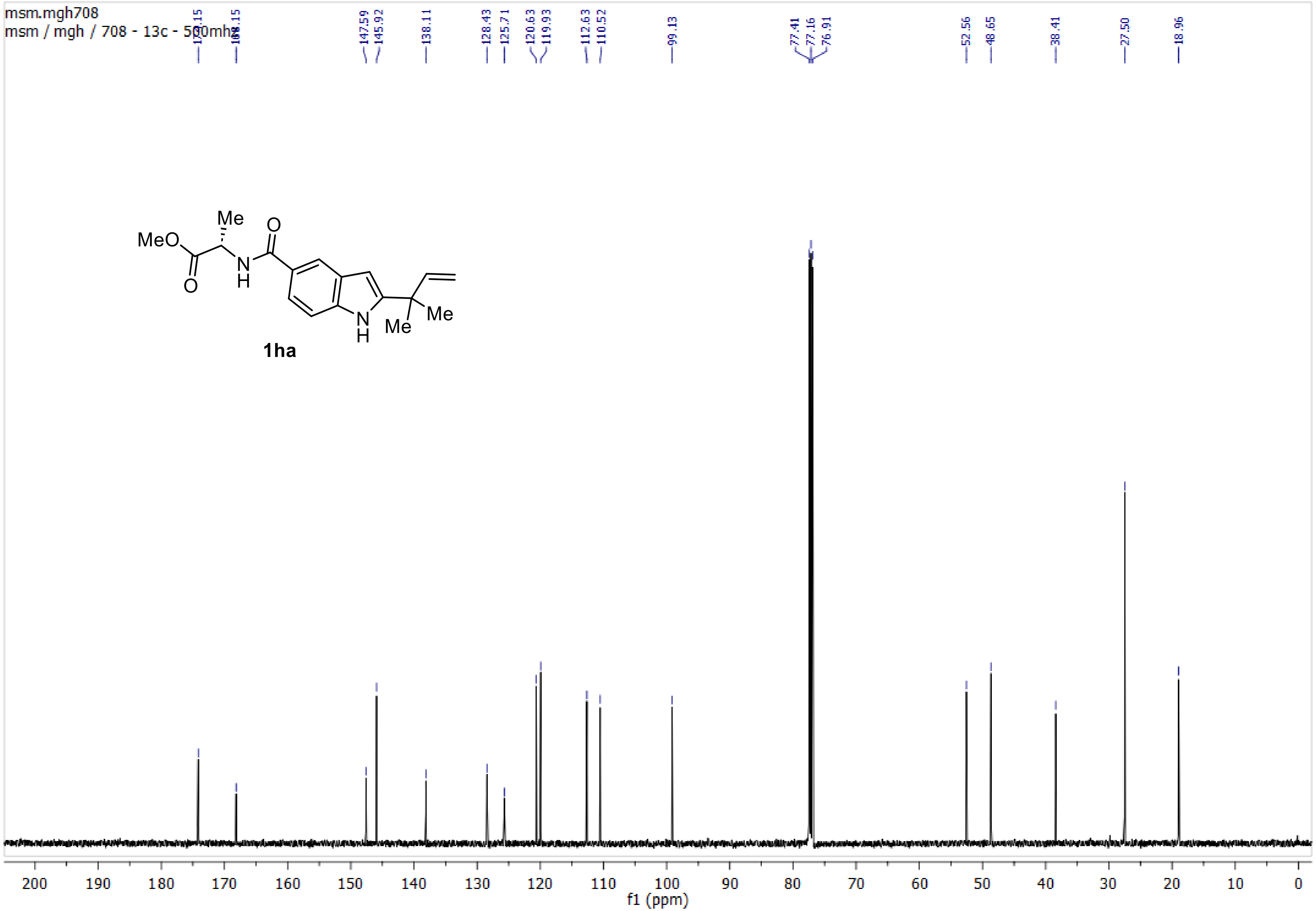
msm.mgh708
msm / mgh / 708 - 13c - 500mhz
174.15
168.15
147.59
145.92
138.11
128.43
125.71
120.63
119.93
112.63
110.52
99.13
77.41
77.16
76.91
52.56
48.65
38.41
27.50
18.96
MeO
Me
O
N
H
O
N
H
Me
Me
1ha
200
190
180
170
160
150
140
130
120
110
100
90
80
70
60
50
40
30
20
10
0
f1 (ppm)

IIT Kharagpur

Sample Information

Sample ID	: MSM-MGH-1007
Vial#	: 10
Injection Volume	: 15
Method File	: 80-20 ID3 60 min 0.5 mL-min- 15- 16.11.2023.lcm
Batch File	: 80-20 MGH 1007 ID3 0.5 ml-min -15- 60 min-16.11.2023.lcb

Chromatogram

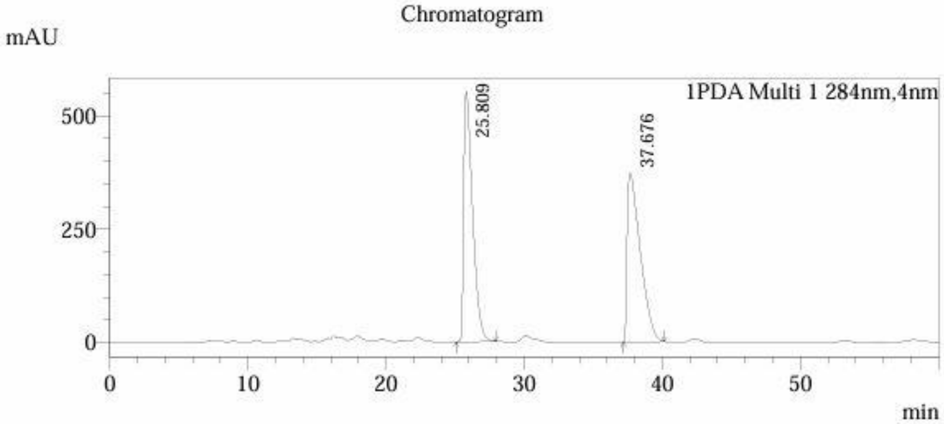

Peak Table

PDA Ch1 284nm

Peak#	Ret. Time	Area	Area%
1	25.809	24844898	50.097
2	37.676	24748902	49.903
Total		49593800	100.000

Sample Information

Sample ID	: MSM-MGH-708
Vial#	: 11
Injection Volume	: 5
Method File	: 80-20 ID3 60 min 0.5 mL-min-5- 16.11.2023.lcm
Batch File	: 80-20 MGH 708 ID3 0.5 ml-min -5- 60 min-16.11.2023.lcb

Chromatogram

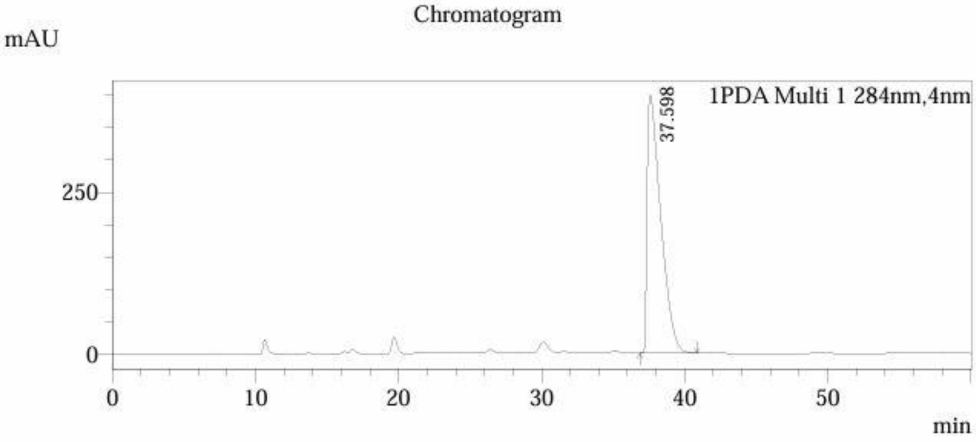

Peak Table

PDA Ch1 284nm

Peak#	Ret. Time	Area	Area%
1	37.598	26297892	100.000
Total		26297892	100.000

msm.ss032306
msm / ss / 03 - 2306 - 1h - 500mhz

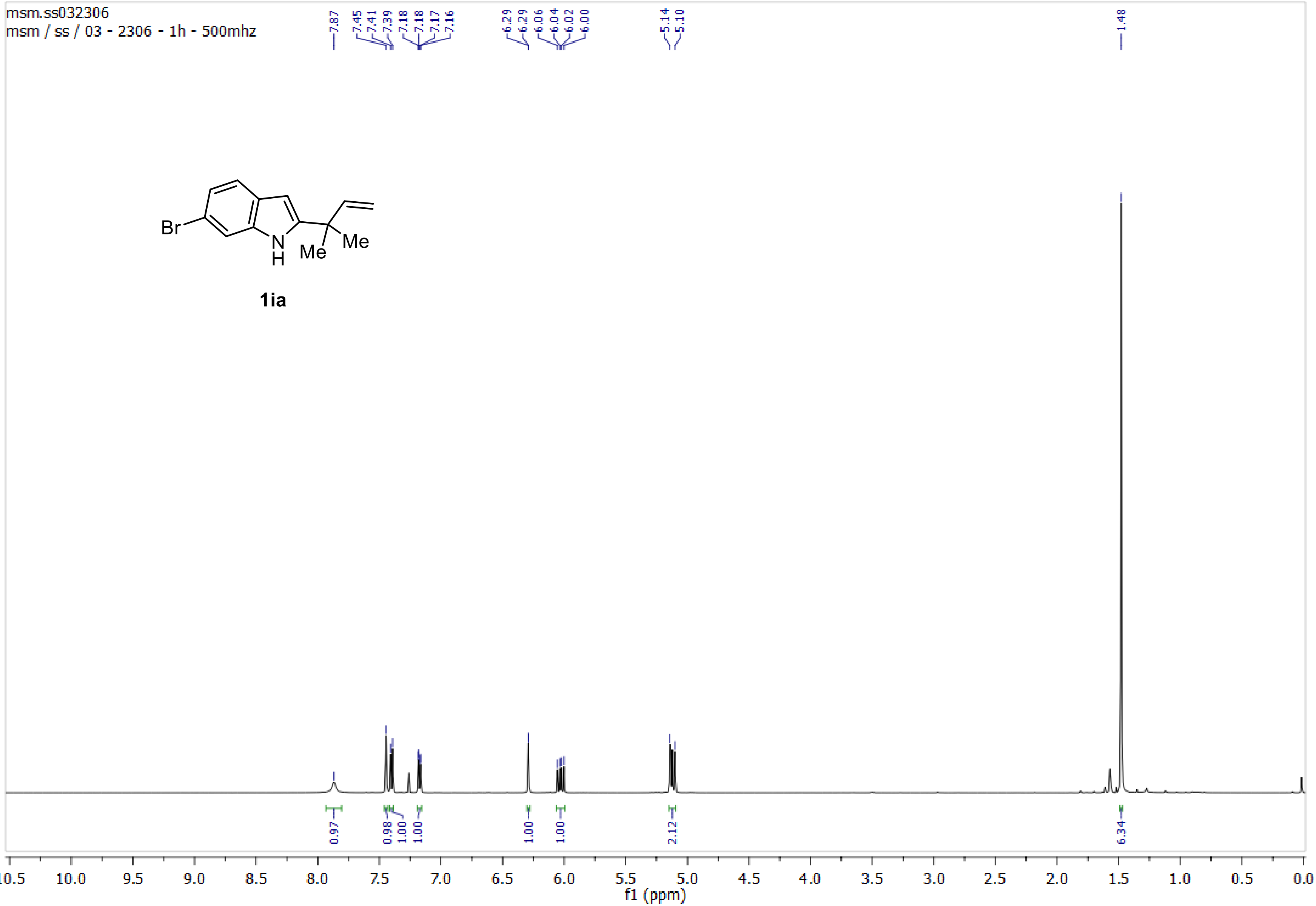

1ia

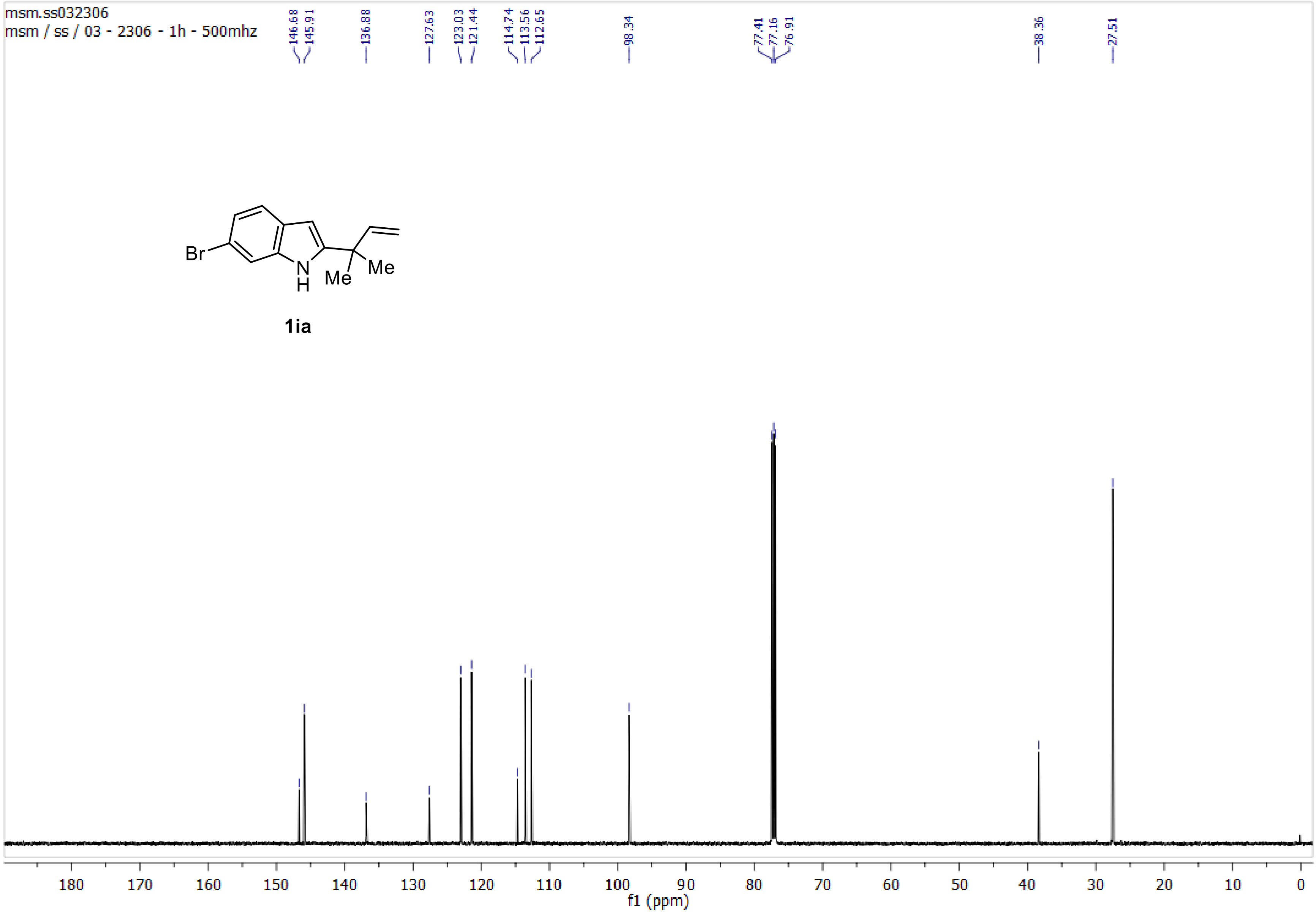
msm.ss032306
msm / ss / 03 - 2306 - 1h - 500mhz
146.68
145.91
136.88
127.63
123.03
121.44
114.74
113.56
112.65
98.34
77.41
77.16
76.91
38.36
27.51
Br
N
H
Me
Me
1ia
180
170
160
150
140
130
120
110
100
90
80
70
60
50
40
30
20
10
0
f1 (ppm)

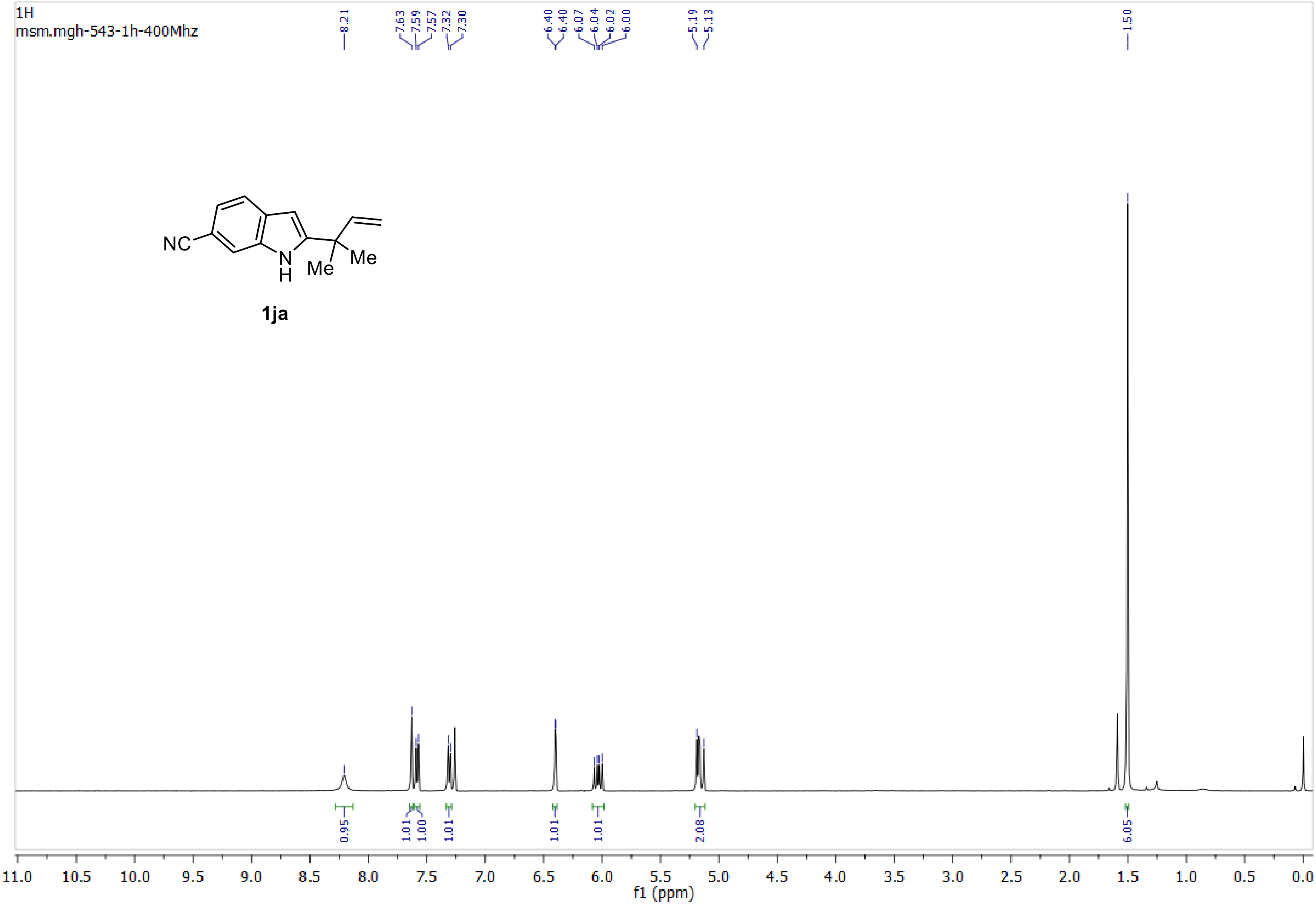
1H
msm.mgh-543-1h-400Mhz
8.21
7.63
7.59
7.57
7.32
7.30
6.40
6.40
6.07
6.04
6.02
6.00
5.19
5.13
1.50
NC
N
H
Me
Me
1ja
0.95
1.01
1.00
1.01
1.01
1.01
2.08
6.05
11.0 10.5 10.0 9.5 9.0 8.5 8.0 7.5 7.0 6.5 6.0 5.5 5.0 4.5 4.0 3.5 3.0 2.5 2.0 1.5 1.0 0.5 0.0
f1 (ppm)

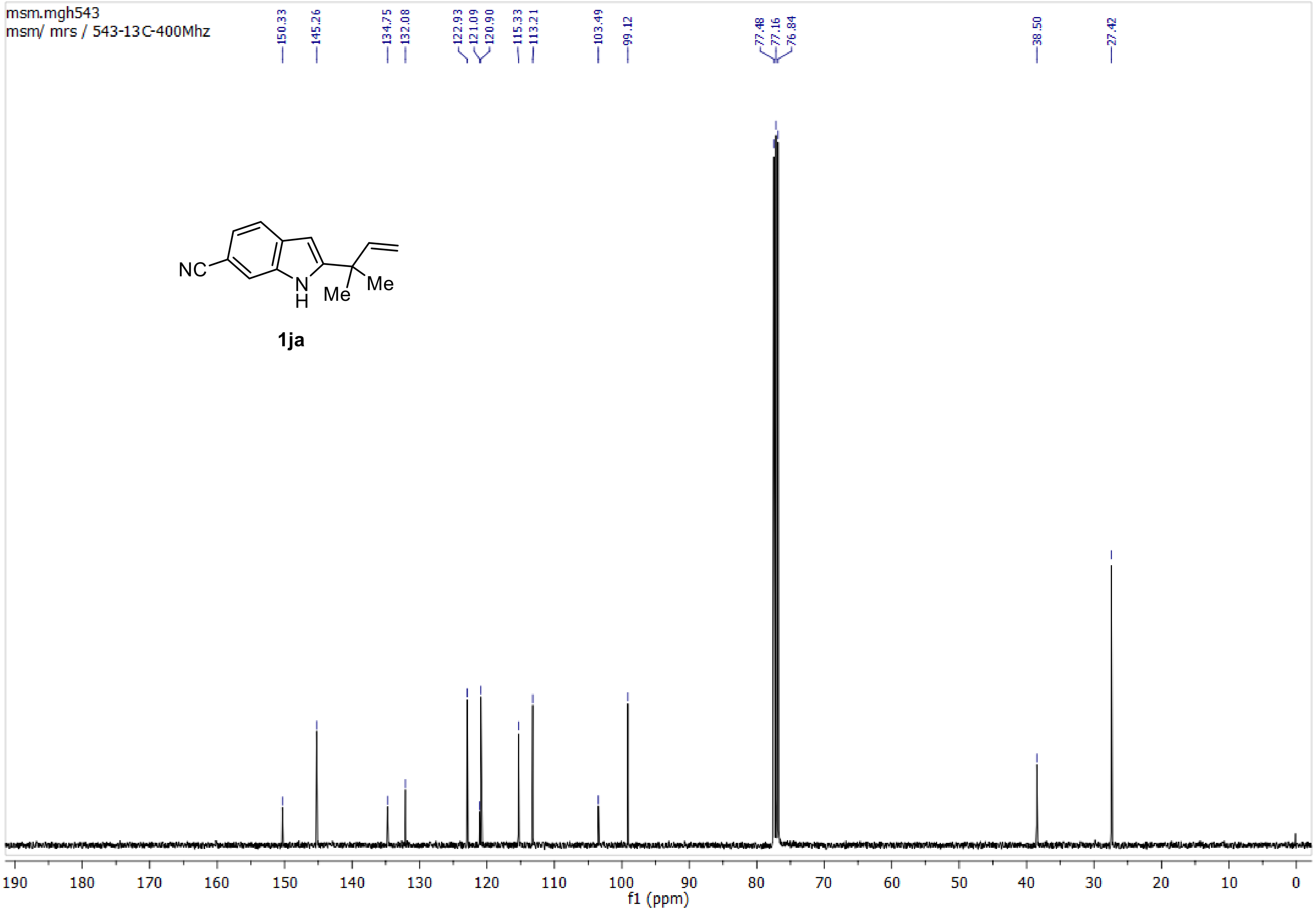
msm.mgh543
msm/ mrs / 543-13C-400Mhz
150.33
145.26
134.75
132.08
122.93
121.09
120.90
115.33
113.21
103.49
99.12
77.48
77.16
76.84
38.50
27.42
NC
N
H
Me
Me
1ja
190
180
170
160
150
140
130
120
110
100
90
80
70
60
50
40
30
20
10
0
f1 (ppm)

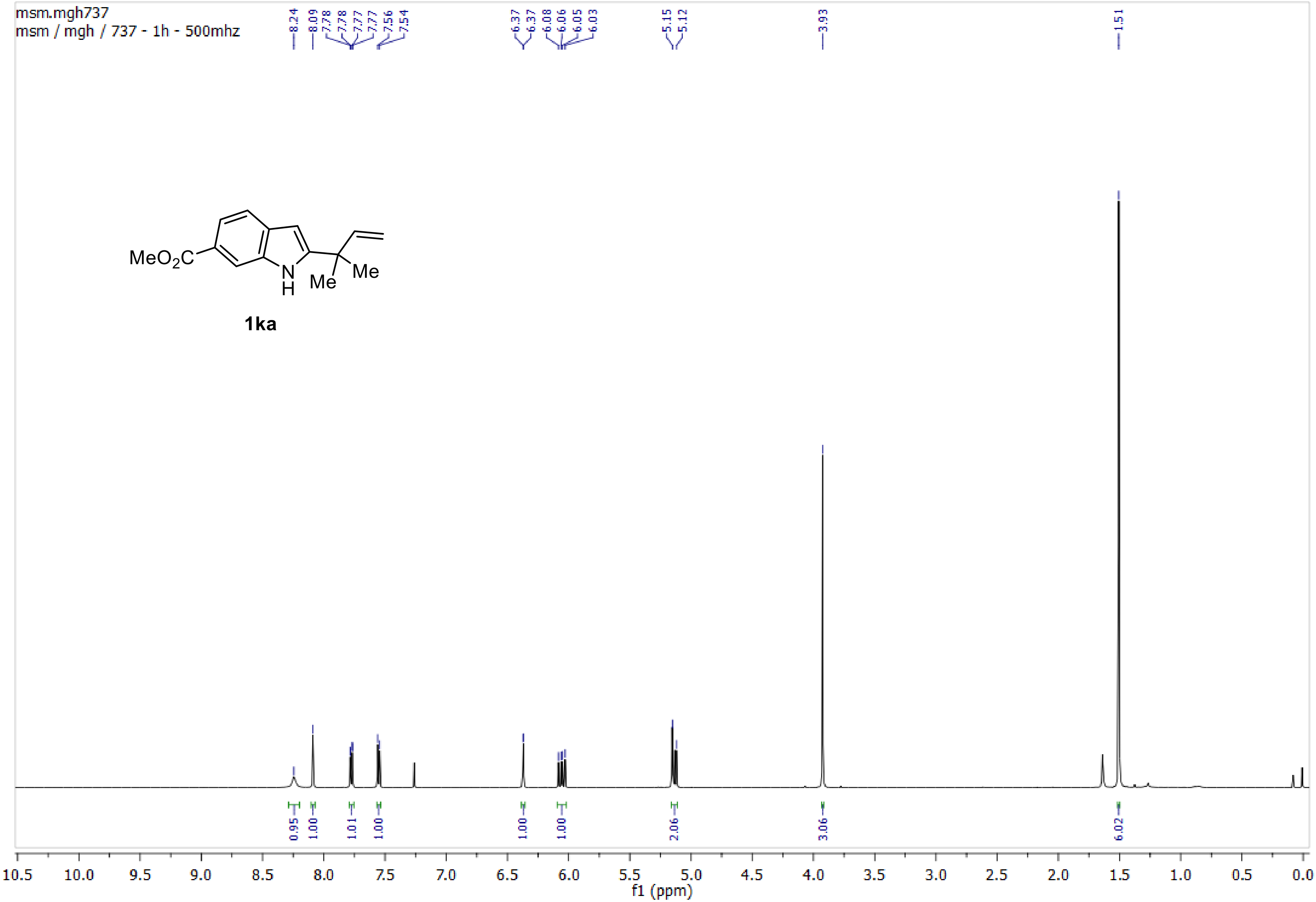
msm.mgh737
msm / mgh / 737 - 1h - 500mhz
8.24
8.09
7.78
7.78
7.77
7.77
7.56
7.54
6.37
6.37
6.08
6.06
6.05
6.03
5.15
5.12
3.93
1.51
MeO2C
N
H
Me
Me
1ka
0.95
1.00
1.01
1.00
1.00
1.00
2.06
3.06
6.02
10.5 10.0 9.5 9.0 8.5 8.0 7.5 7.0 6.5 6.0 5.5 5.0 4.5 4.0 3.5 3.0 2.5 2.0 1.5 1.0 0.5 0.0
f1 (ppm)

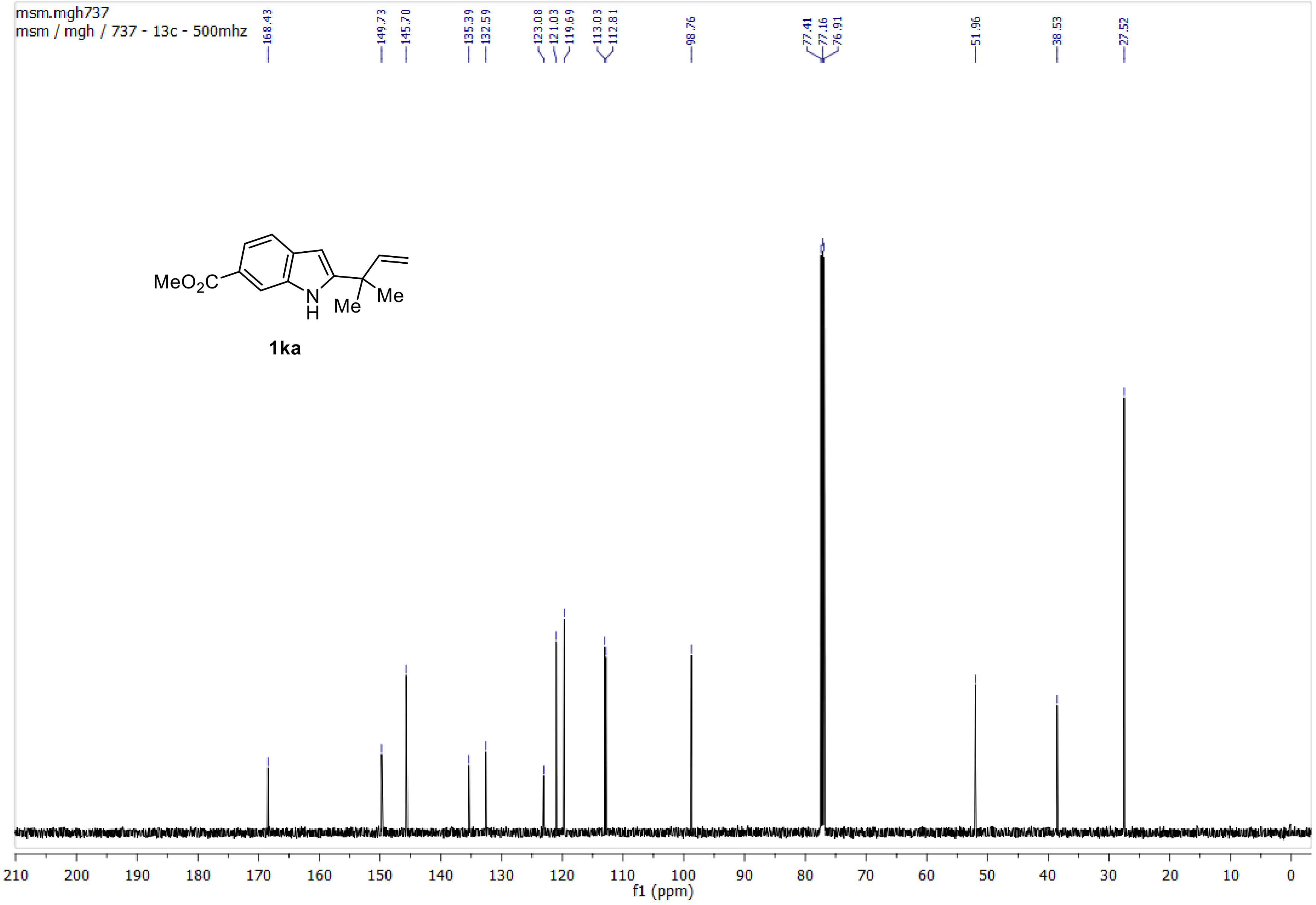
msm.mgh737
msm / mgh / 737 - 13c - 500mhz
168.43
149.73
145.70
135.39
132.59
123.08
121.03
119.69
113.03
112.81
98.76
77.41
77.16
76.91
51.96
38.53
27.52
MeO2C
N
H
Me
Me
1ka
210
200
190
180
170
160
150
140
130
120
110
100
90
80
70
60
50
40
30
20
10
0
f1 (ppm)

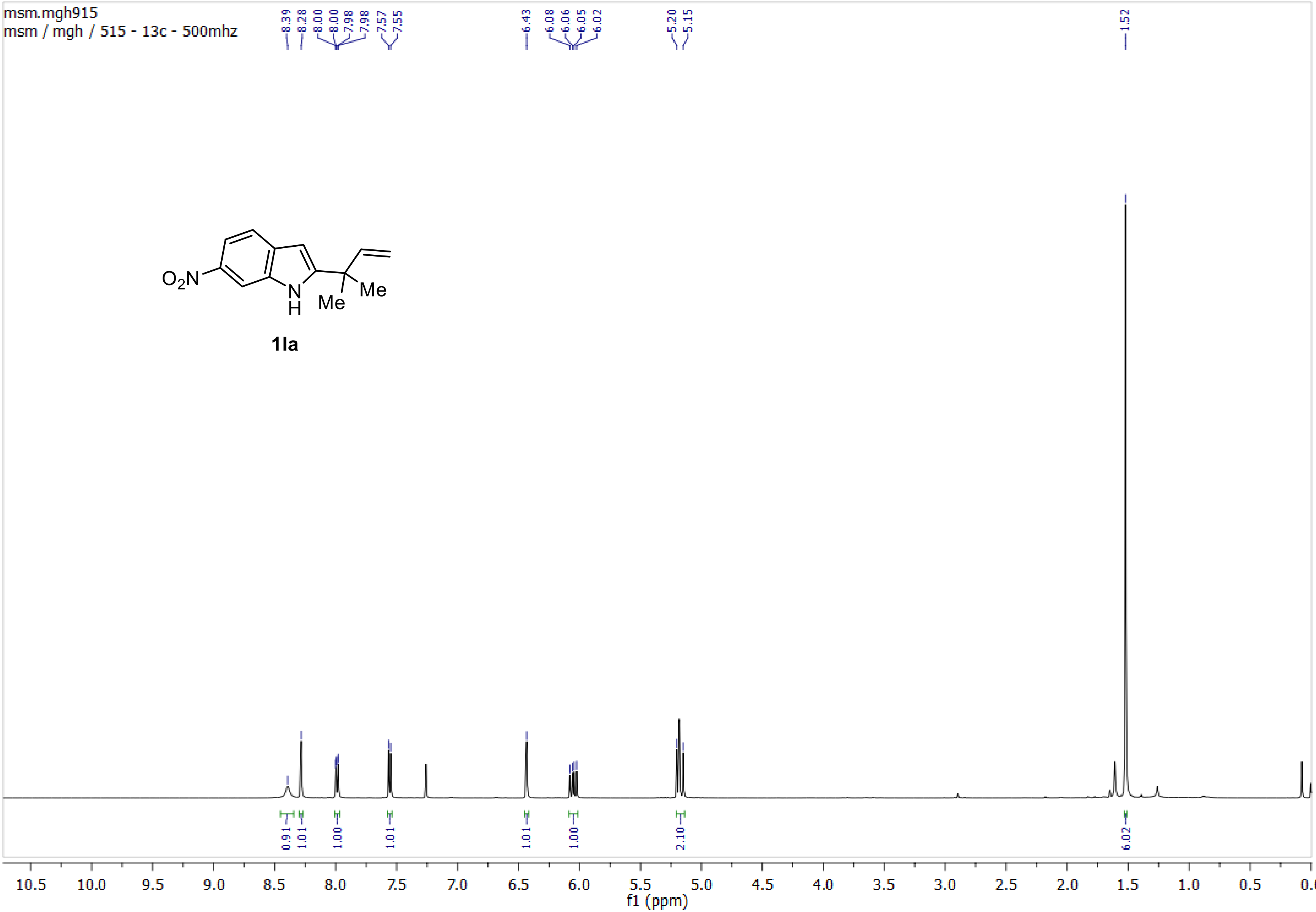
msm.mgh915
msm / mgh / 515 - 13c - 500mhz
8.39
8.28
8.00
8.00
7.98
7.98
7.57
7.55
6.43
6.08
6.06
6.05
6.02
5.20
5.15
1.52
O_2N
N
H
Me
Me
0.91
1.01
1.00
1.01
1.01
1.00
2.10
6.02
10.5
10.0
9.5
9.0
8.5
8.0
7.5
7.0
6.5
6.0
5.5
5.0
4.5
4.0
3.5
3.0
2.5
2.0
1.5
1.0
0.5
0.0
f1 (ppm)

1la

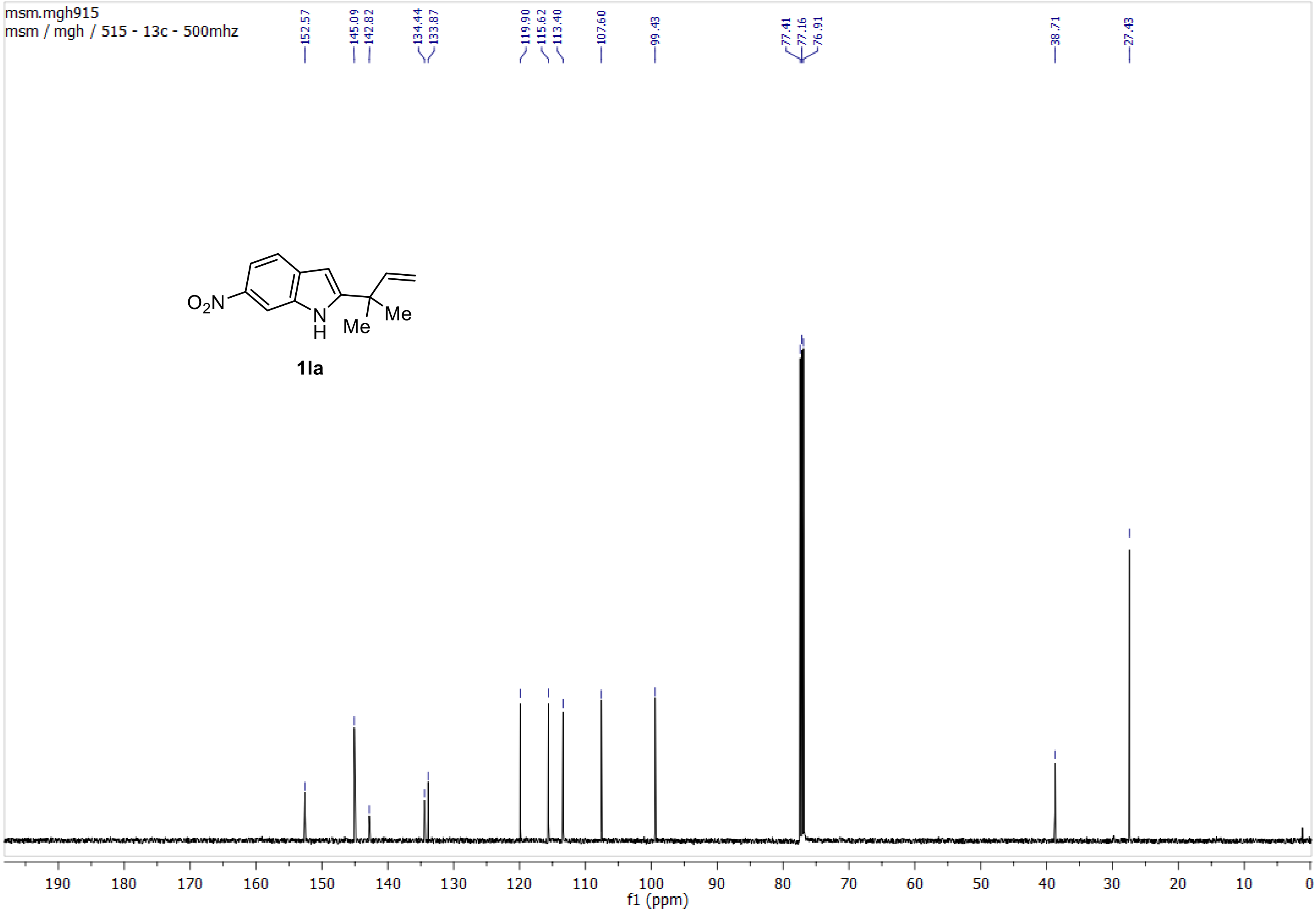
msm.mgh915
msm / mgh / 515 - 13c - 500mhz
152.57
145.09
142.82
134.44
133.87
119.90
115.62
113.40
107.60
99.43
77.41
77.16
76.91
38.71
27.43
O_2N
N
H
Me
Me
1la
190 180 170 160 150 140 130 120 110 100 90 80 70 60 50 40 30 20 10 0
f1 (ppm)

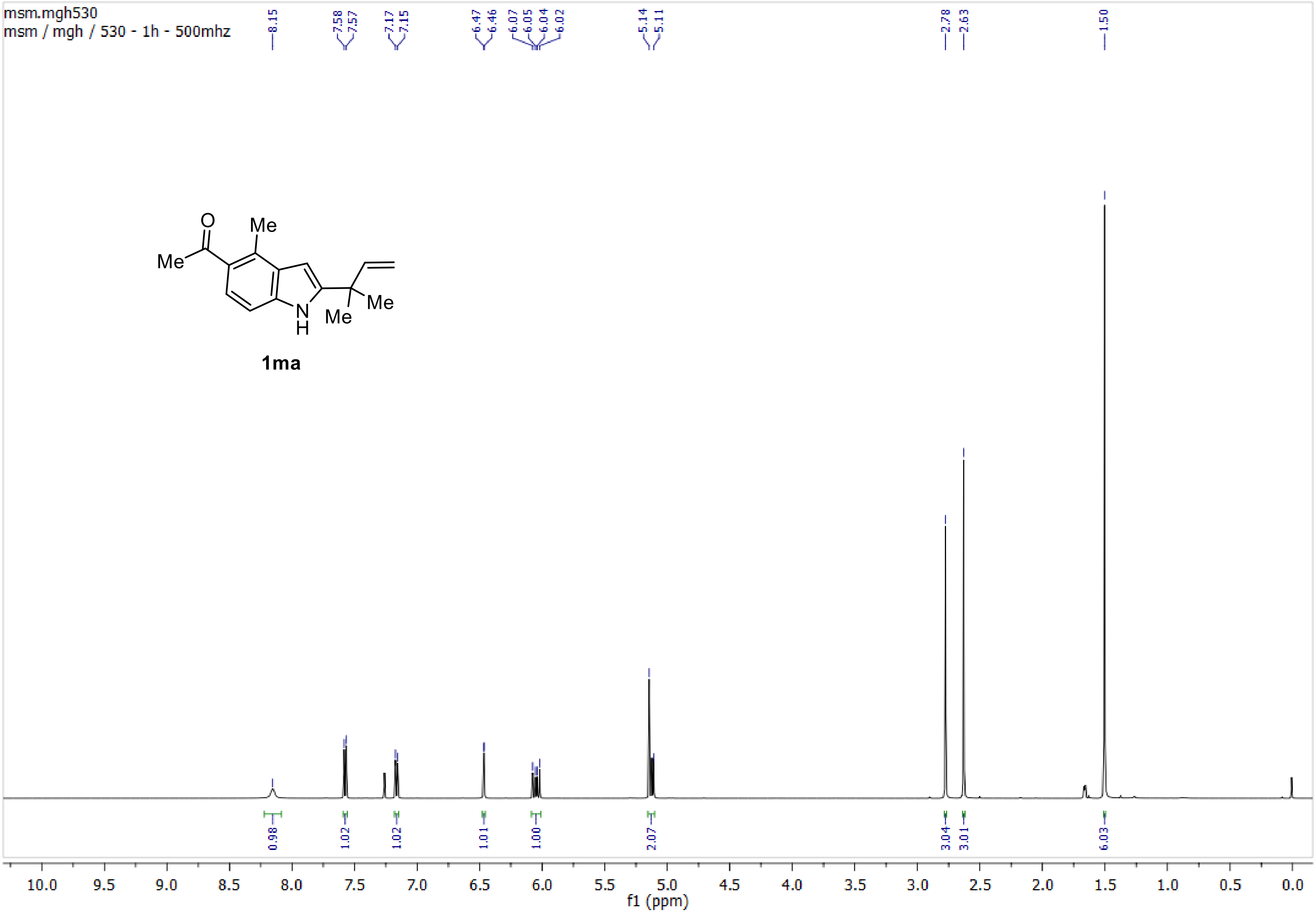

msm.mgh530
msm / mgh / 530 - 1h - 500mhz
8.15
7.58
7.57
7.17
7.15
6.47
6.46
6.07
6.05
6.04
6.02
5.14
5.11
2.78
2.63
1.50
0.98
1.02
1.02
1.01
1.00
2.07
3.04
3.01
6.03
10.0 9.5 9.0 8.5 8.0 7.5 7.0 6.5 6.0 5.5 5.0 4.5 4.0 3.5 3.0 2.5 2.0 1.5 1.0 0.5 0.0
f1 (ppm)
O
Me
Me
N
H
Me
Me

1ma

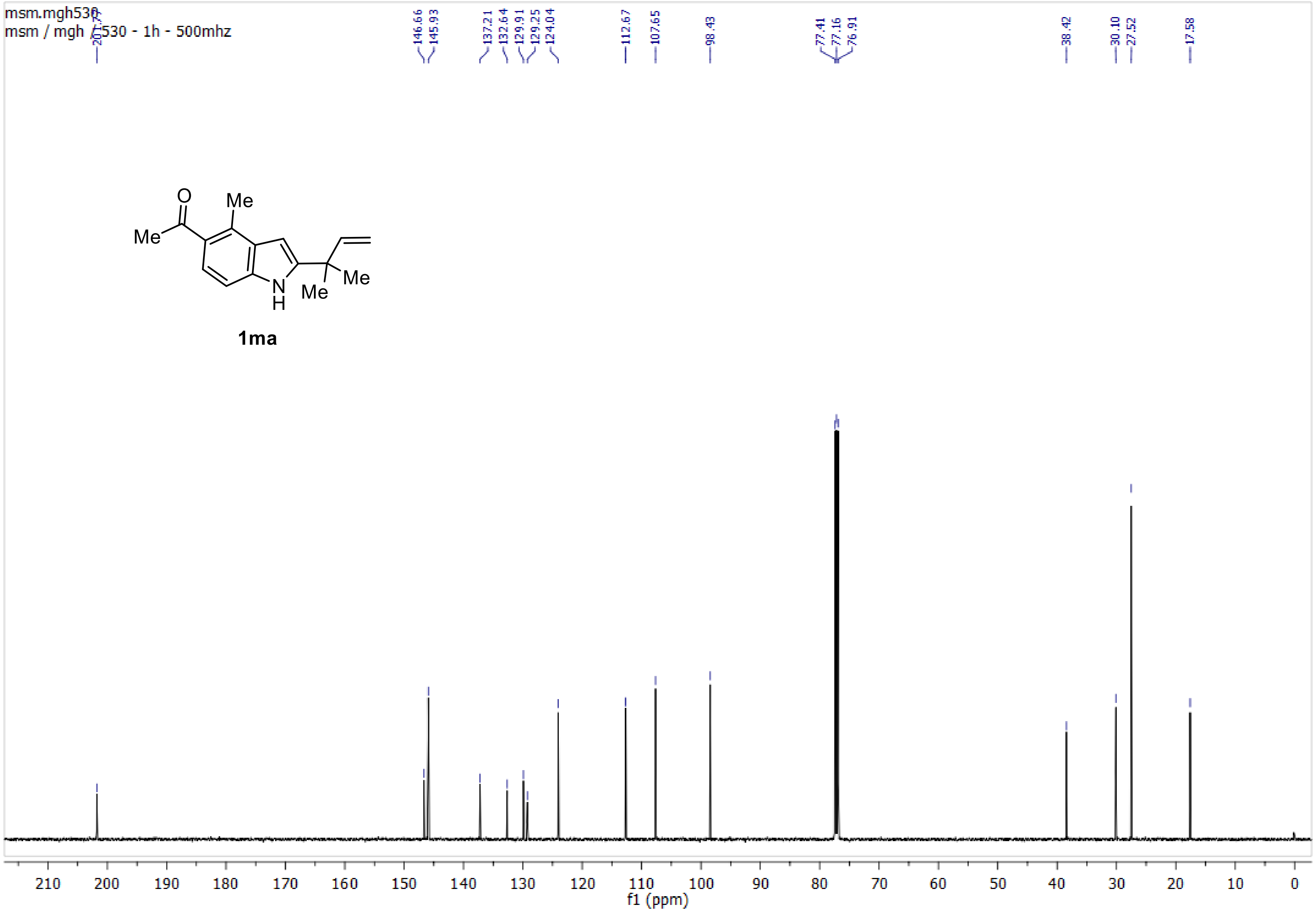

msm.mgh530
msm / mgh / 530 - 1h - 500mhz
201.77
146.66
145.93
137.21
132.64
129.91
129.25
124.04
112.67
107.65
98.43
77.41
77.16
76.91
38.42
30.10
27.52
17.58
O
Me
Me
N
H
Me
Me
1ma
210 200 190 180 170 160 150 140 130 120 110 100 90 80 70 60 50 40 30 20 10 0
f1 (ppm)

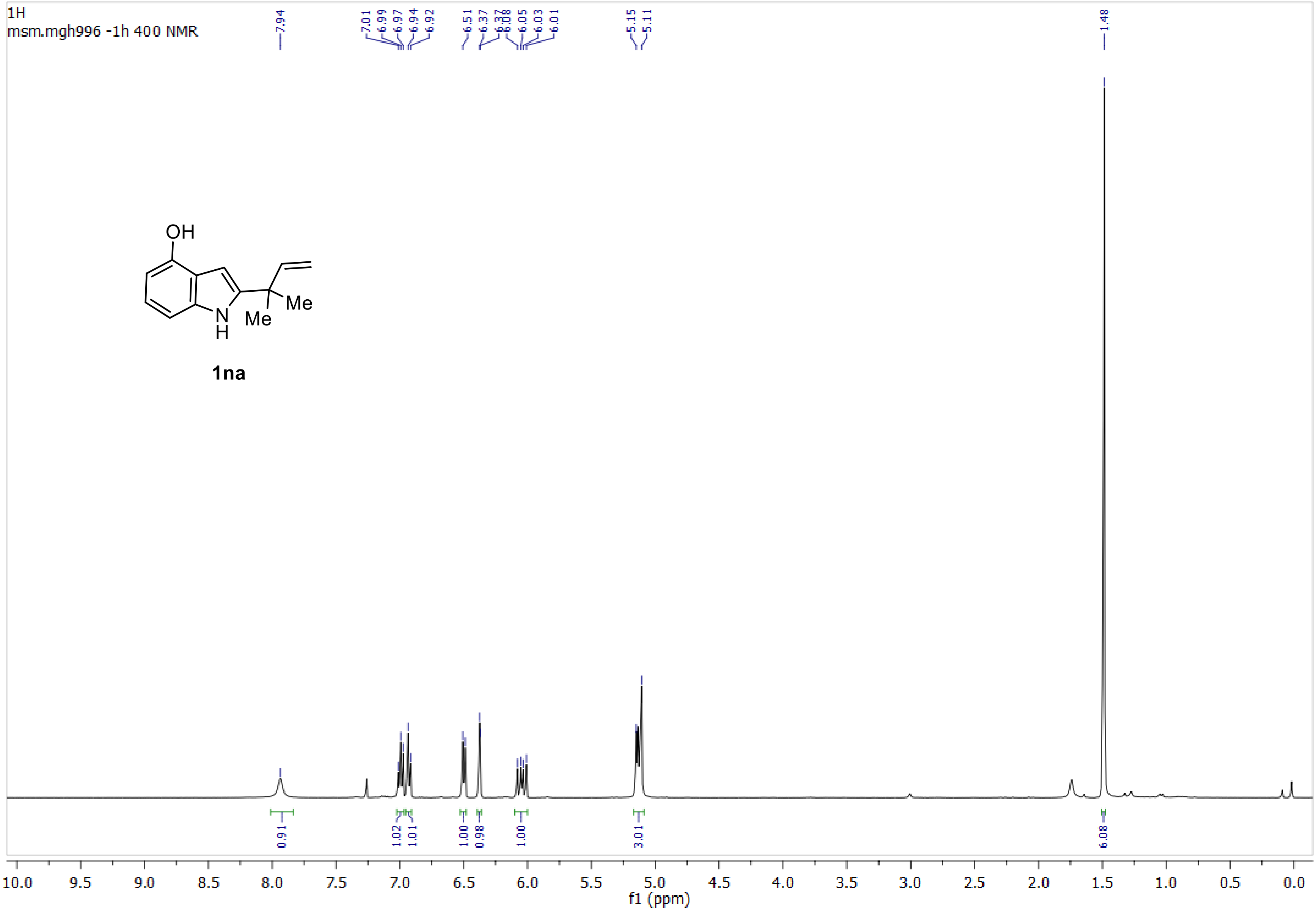
1H
msm.mgh996 -1h 400 NMR
7.94
7.01
6.99
6.97
6.94
6.92
6.51
6.37
6.37
6.08
6.05
6.03
6.01
5.15
5.11
1.48
OH
N
H
Me
Me
1na
0.91
1.02
1.01
1.00
0.98
1.00
3.01
6.08
10.0
9.5
9.0
8.5
8.0
7.5
7.0
6.5
6.0
5.5
5.0
4.5
4.0
3.5
3.0
2.5
2.0
1.5
1.0
0.5
0.0
f1 (ppm)

13C
msm.mgh996 -13c 400 NMR

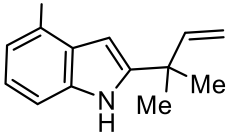

1na

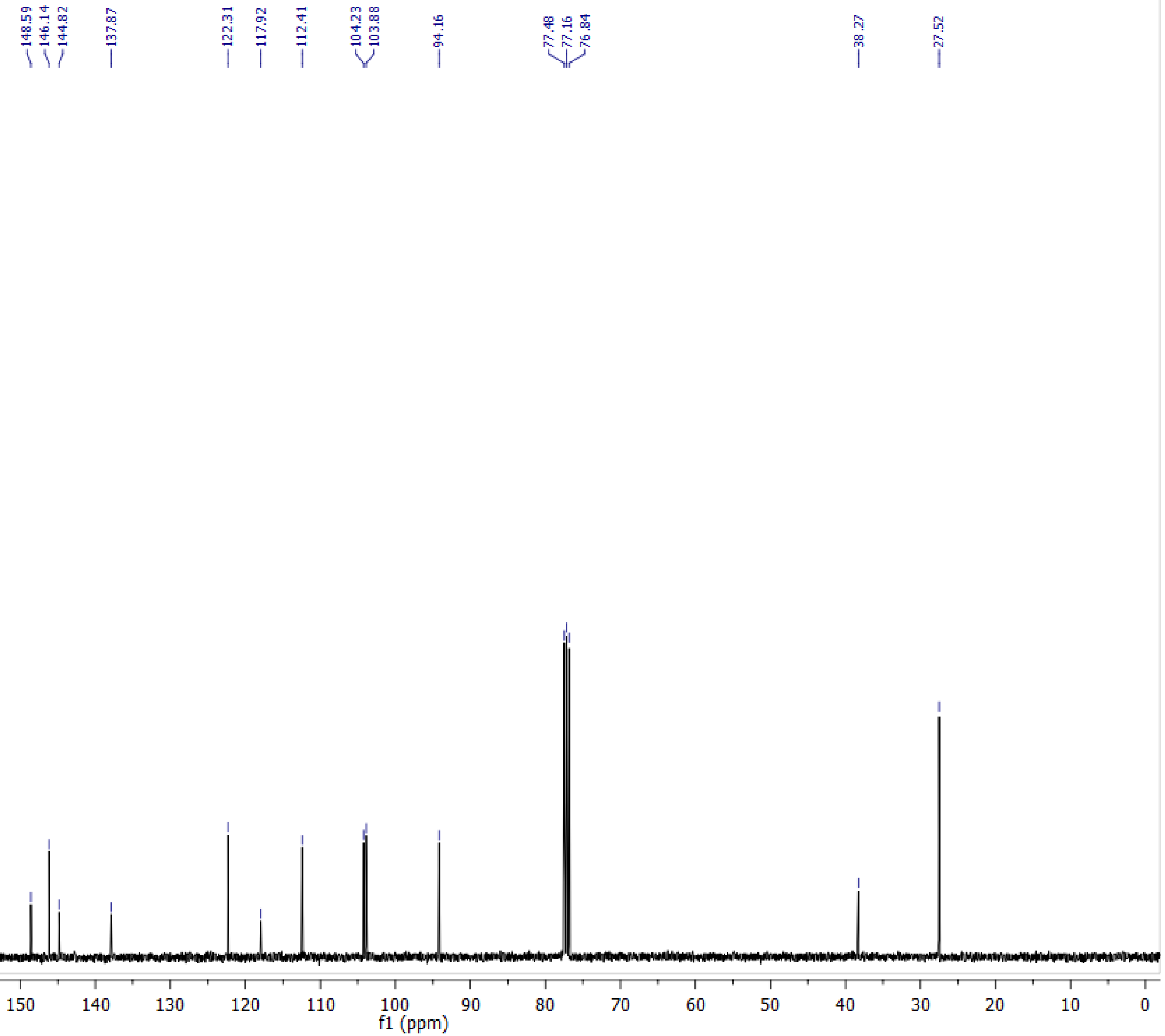

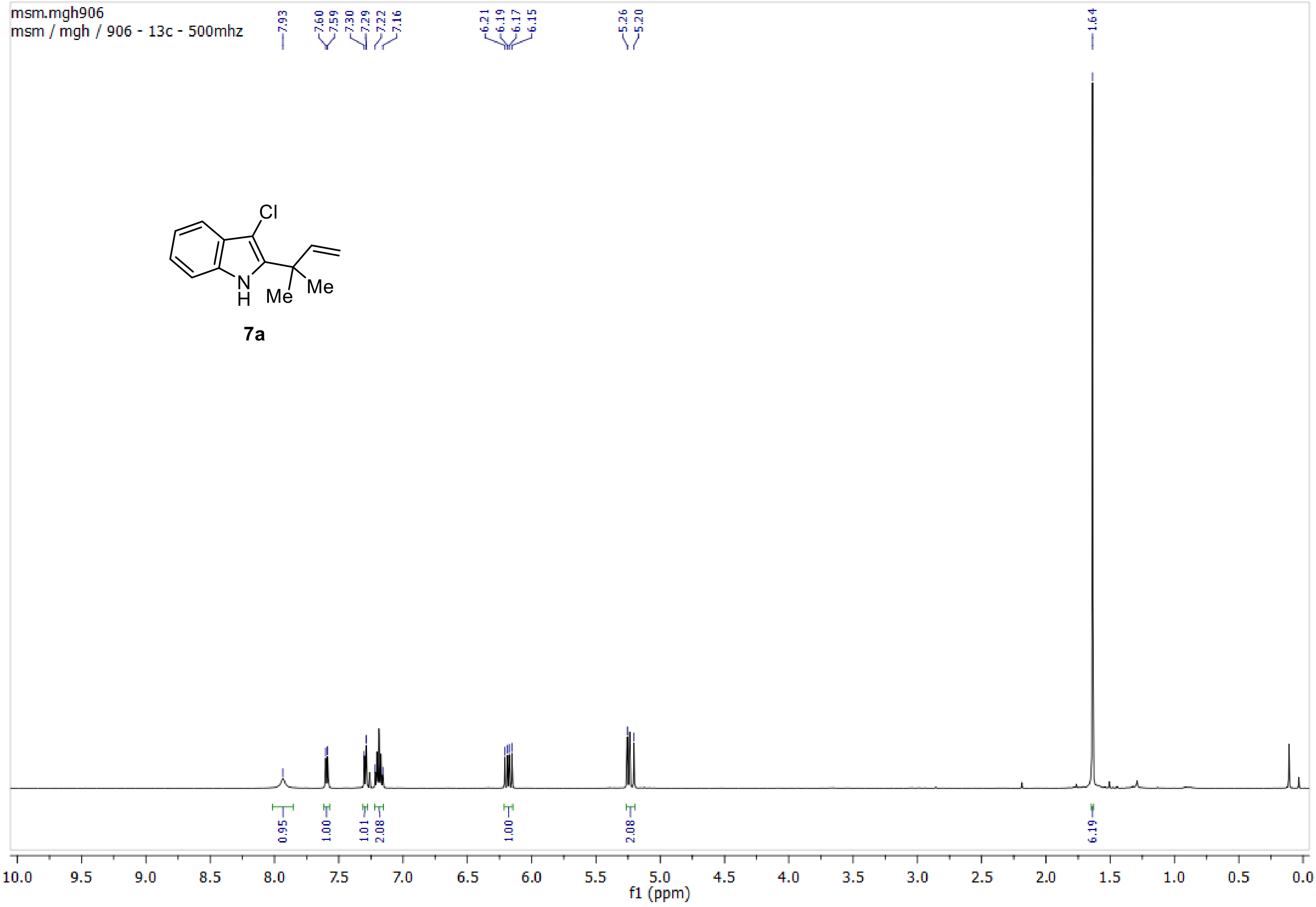
msm.mgh906
msm / mgh / 906 - 13c - 500mhz
7.93
7.60
7.59
7.30
7.29
7.22
7.16
6.21
6.19
6.17
6.15
5.26
5.20
1.64
Cl
N
H
Me
Me
7a
0.95
1.00
1.01
2.08
1.00
2.08
6.19
10.0
9.5
9.0
8.5
8.0
7.5
7.0
6.5
6.0
5.5
5.0
4.5
4.0
3.5
3.0
2.5
2.0
1.5
1.0
0.5
0.0
f1 (ppm)

msm.mgh906
msm / mgh / 906 - 13c - 500mhz

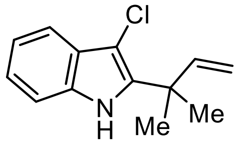

7a

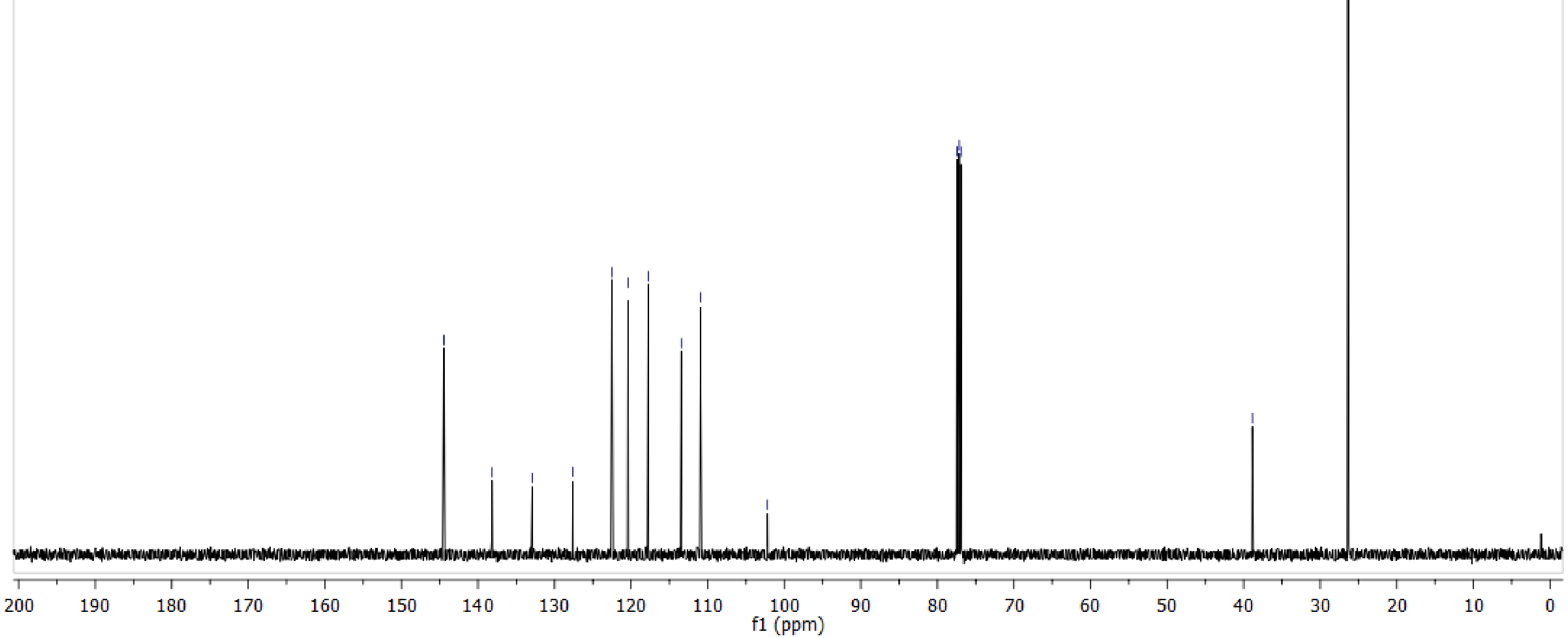

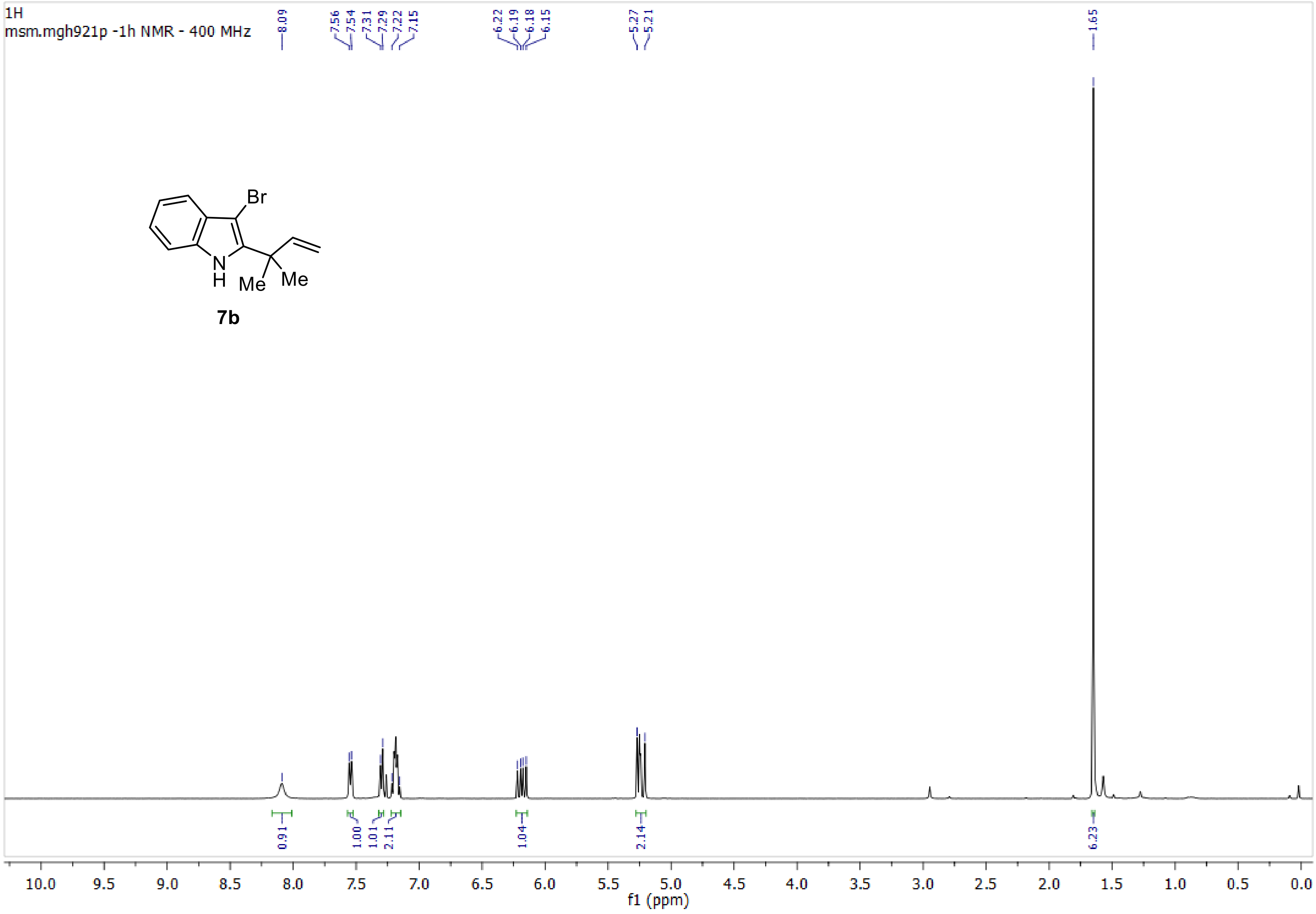
1H
msm.mgh921p -1h NMR - 400 MHz
8.09
7.56
7.54
7.31
7.29
7.22
7.15
6.22
6.19
6.18
6.15
5.27
5.21
1.65
Br
N
H
Me
Me
7b
0.91
1.00
1.01
2.11
1.04
2.14
6.23
10.0
9.5
9.0
8.5
8.0
7.5
7.0
6.5
6.0
5.5
5.0
4.5
4.0
3.5
3.0
2.5
2.0
1.5
1.0
0.5
0.0
f1 (ppm)

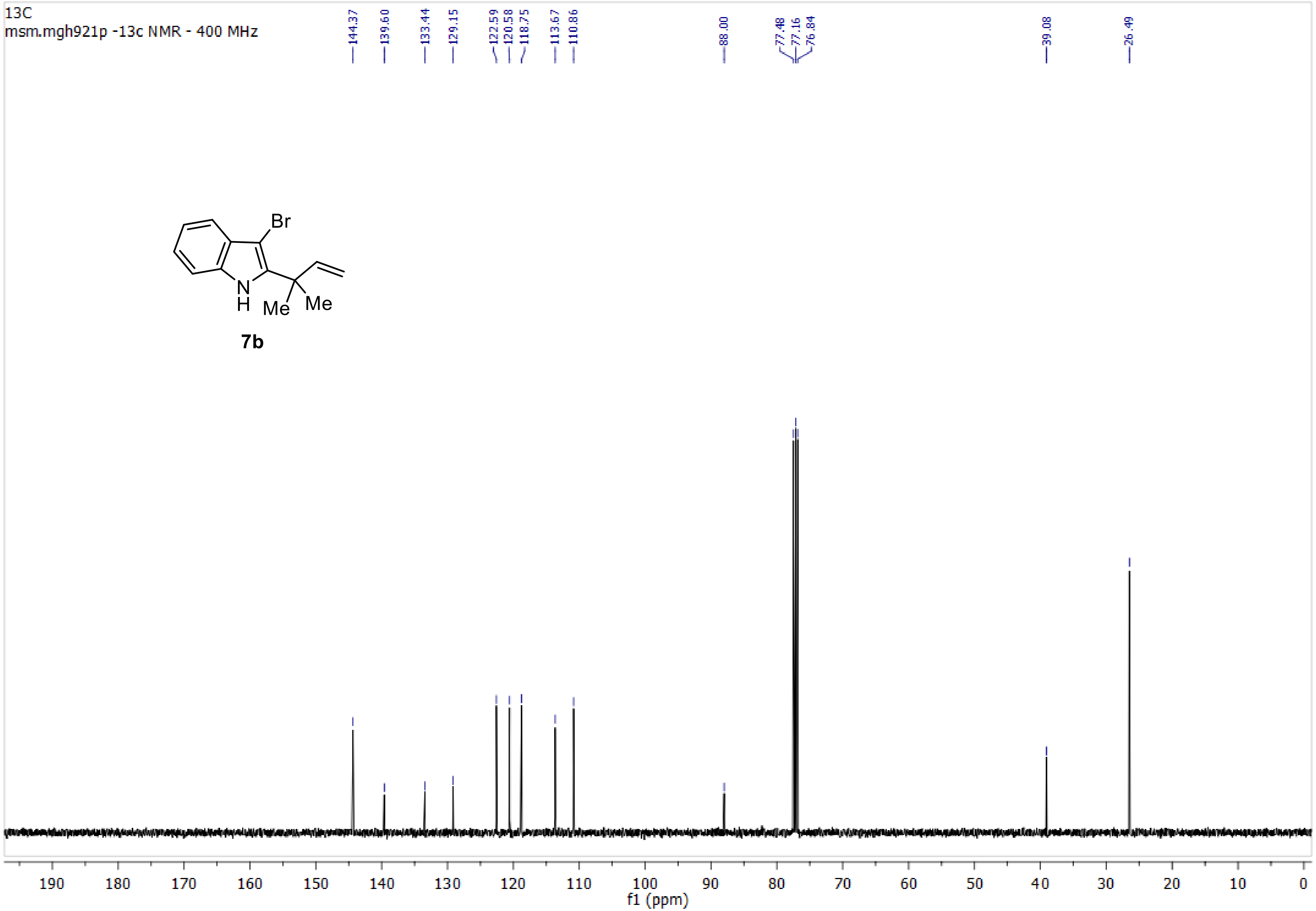
13C
msm.mgh921p -13c NMR - 400 MHz
144.37
139.60
133.44
129.15
122.59
120.58
118.75
113.67
110.86
88.00
77.48
77.16
76.84
39.08
26.49
Br
N
H
Me
Me
7b
190 180 170 160 150 140 130 120 110 100 90 80 70 60 50 40 30 20 10 0
f1 (ppm)

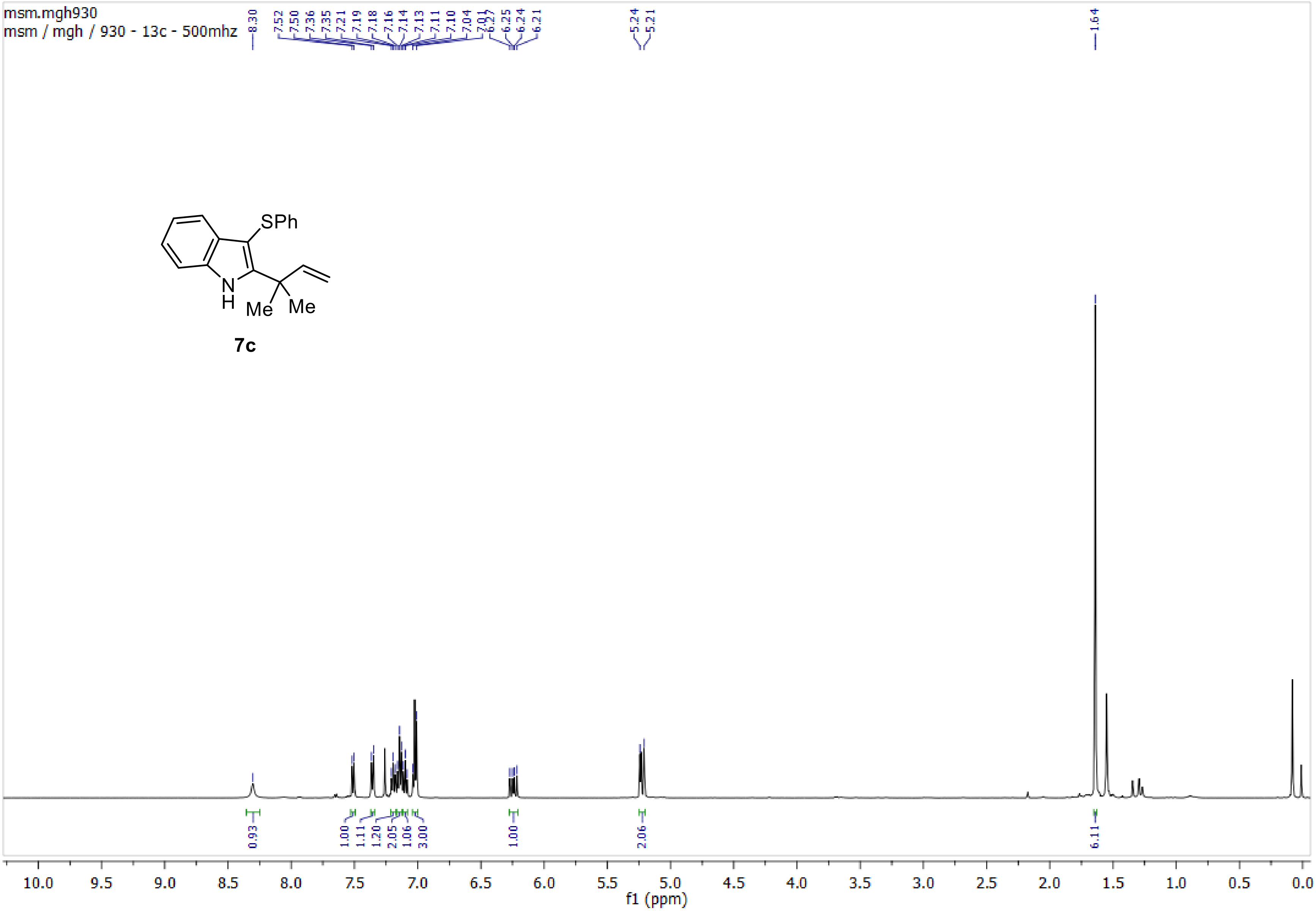
msm.mgh930
msm / mgh / 930 - 13c - 500mhz
8.30
7.52
7.50
7.36
7.35
7.21
7.19
7.18
7.16
7.14
7.13
7.11
7.10
7.04
7.01
6.27
6.25
6.24
6.21
5.24
5.21
1.64
0.93
1.00
1.11
1.20
2.05
1.06
3.00
1.00
2.06
6.11
f1 (ppm)
SPh
N
H
Me
Me

7c

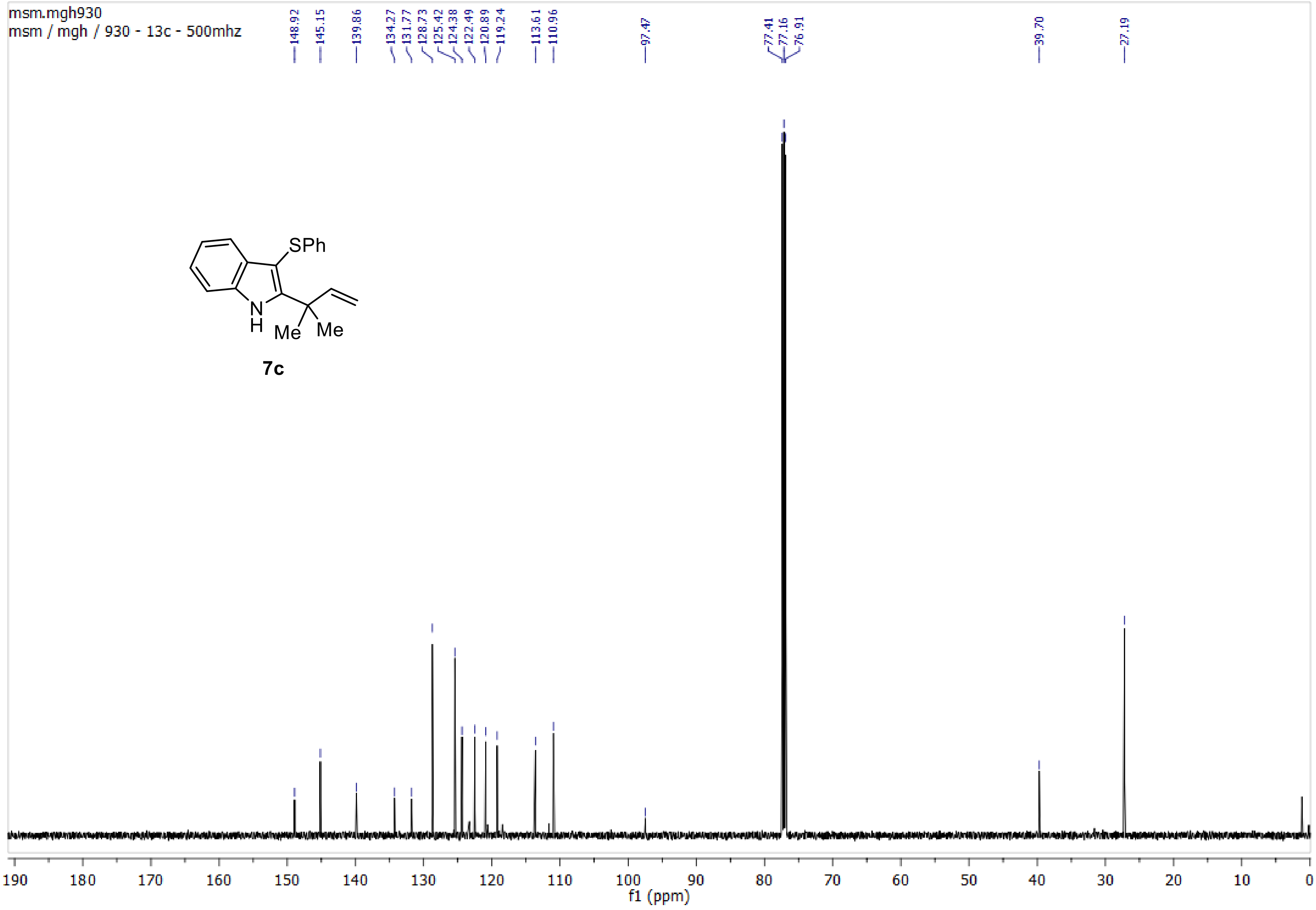
msm.mgh930
msm / mgh / 930 - 13c - 500mhz
148.92
145.15
139.86
134.27
131.77
128.73
125.42
124.38
122.49
120.89
119.24
113.61
110.96
97.47
77.41
77.16
76.91
39.70
27.19
SPh
N
H
Me
Me
7c
190
180
170
160
150
140
130
120
110
100
90
80
70
60
50
40
30
20
10
0
f1 (ppm)

msm.mgh922p
msm / mgh / 922p - 13c - 500mhz

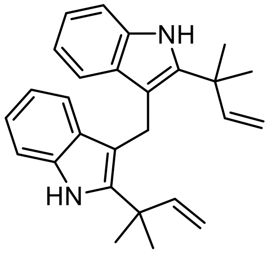

7d

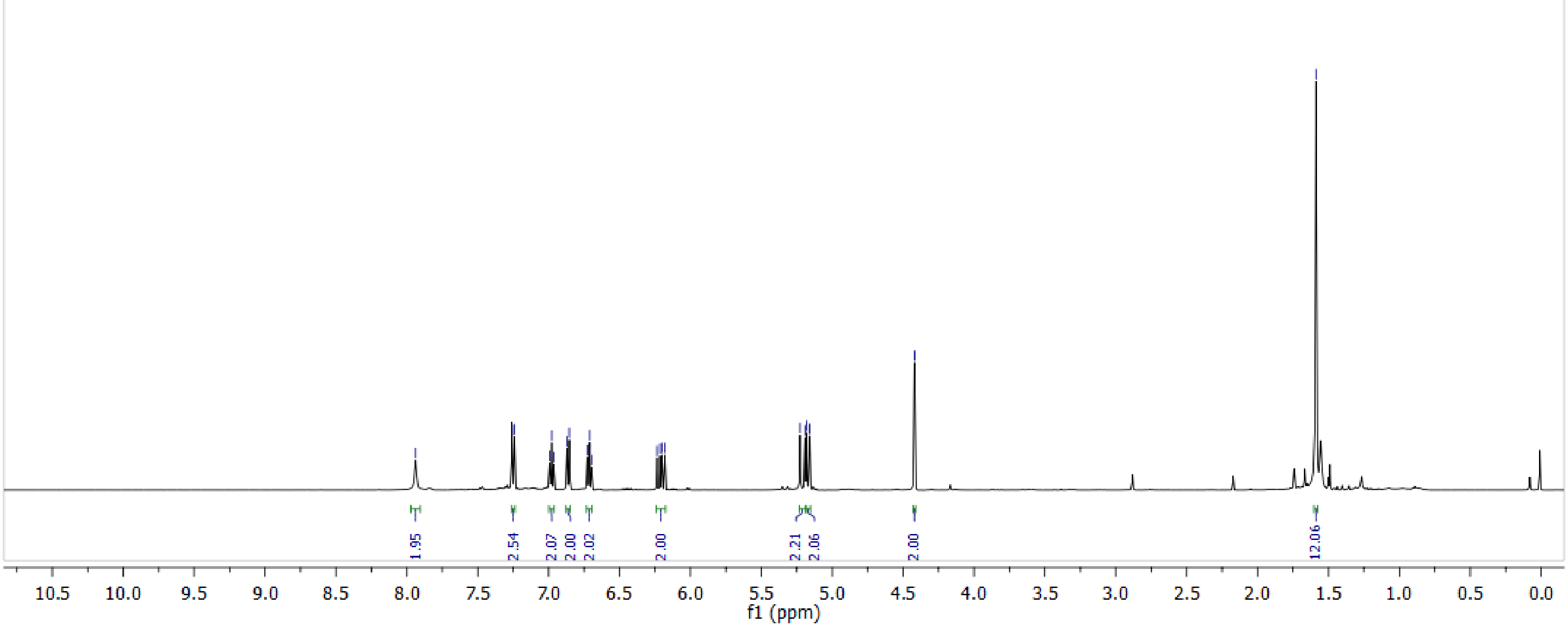

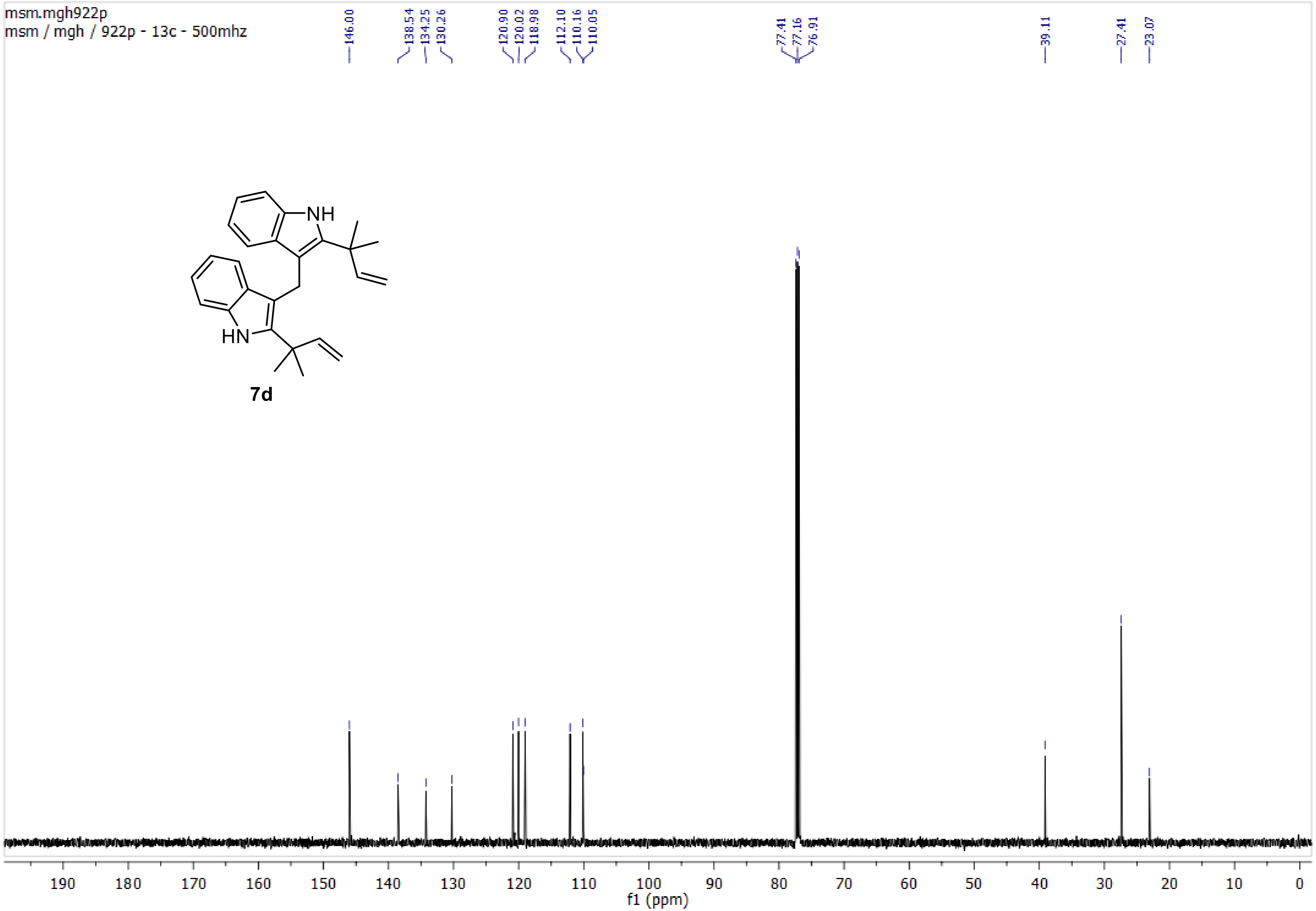
msm.mgh922p
msm / mgh / 922p - 13c - 500mhz
146.00
138.54
134.25
130.26
120.90
120.02
118.98
112.10
110.16
110.05
77.41
77.16
76.91
39.11
27.41
23.07
NH
HN
7d
190 180 170 160 150 140 130 120 110 100 90 80 70 60 50 40 30 20 10 0
f1 (ppm)

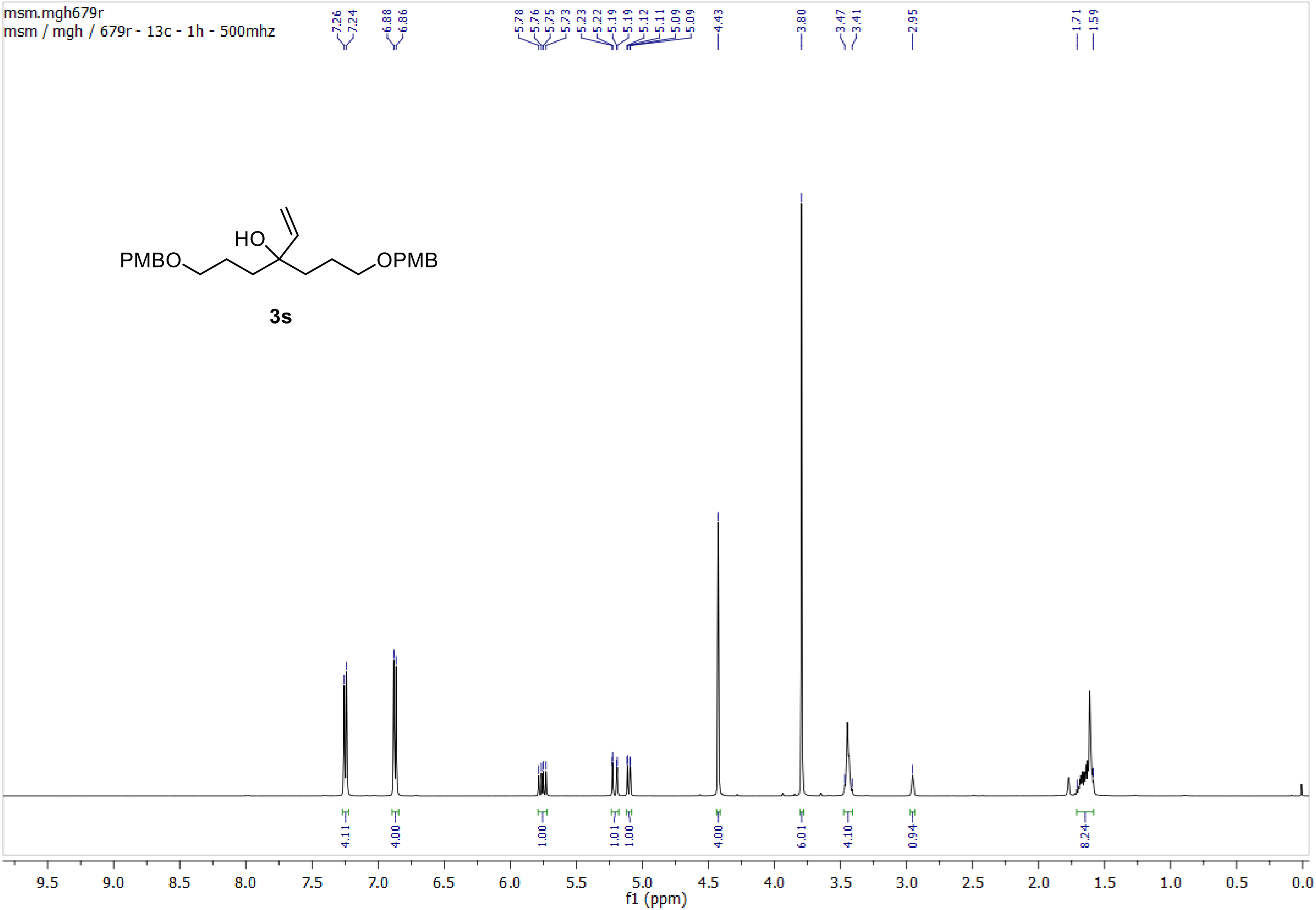
msm.mgh679r
msm / mgh / 679r - 13c - 1h - 500mhz
7.26
7.24
6.88
6.86
5.78
5.76
5.75
5.73
5.23
5.22
5.19
5.19
5.12
5.11
5.09
5.09
4.43
3.80
3.47
3.41
2.95
1.71
1.59
HO
PMBO
OPMB
3s
4.11
4.00
1.00
1.01
1.00
4.00
6.01
4.10
0.94
8.24
9.5
9.0
8.5
8.0
7.5
7.0
6.5
6.0
5.5
5.0
4.5
4.0
3.5
3.0
2.5
2.0
1.5
1.0
0.5
0.0
f1 (ppm)

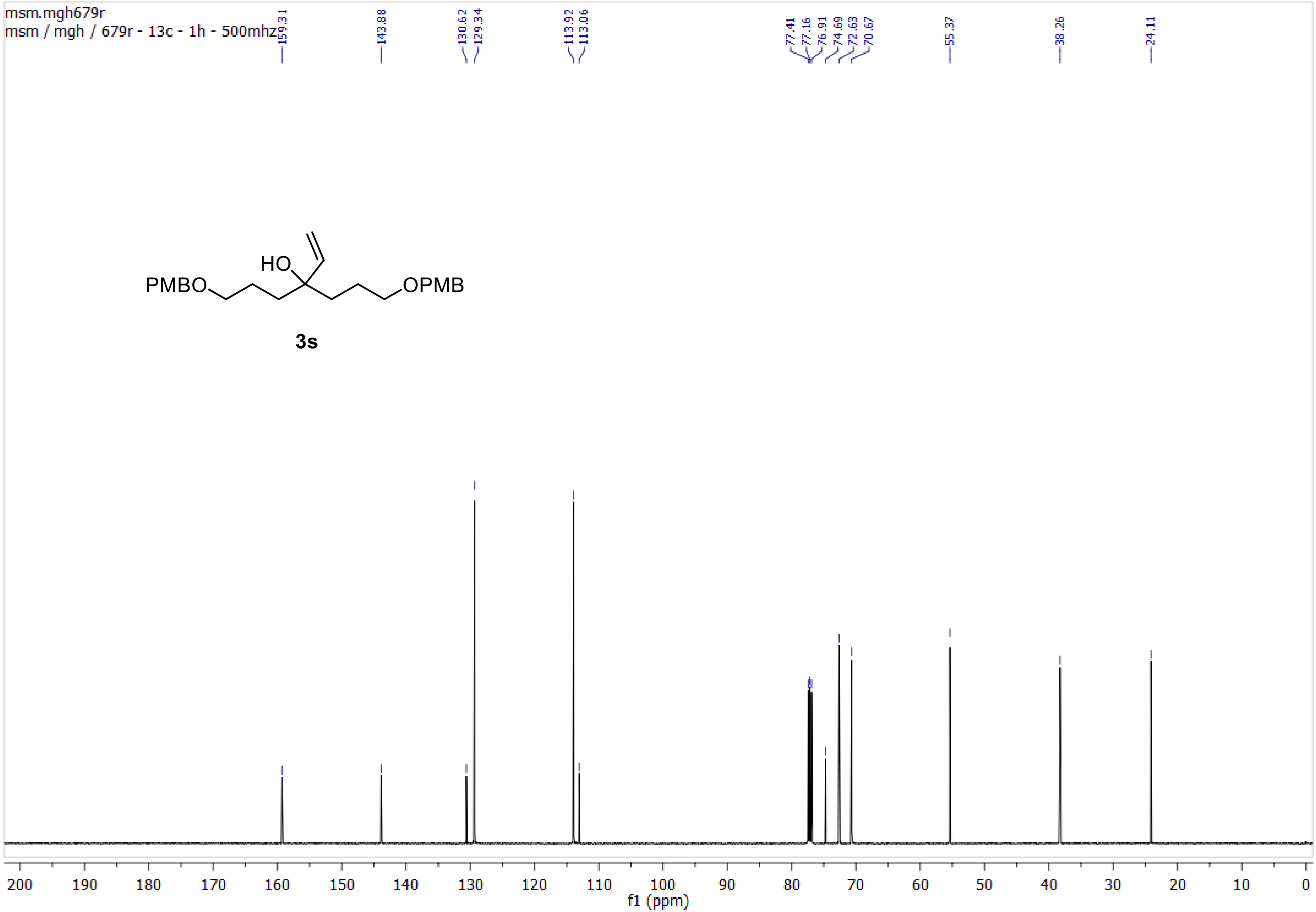
msm.mgh679r
msm / mgh / 679r - 13c - 1h - 500mhz
159.31
143.88
130.62
129.34
113.92
113.06
77.41
77.16
76.91
74.69
72.63
70.67
55.37
38.26
24.11
HO
PMBO
OPMB
3s
200 190 180 170 160 150 140 130 120 110 100 90 80 70 60 50 40 30 20 10 0
f1 (ppm)

msm.mgh753a
msm / mgh / 753a - 1h - 500mhz

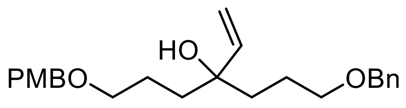

3t

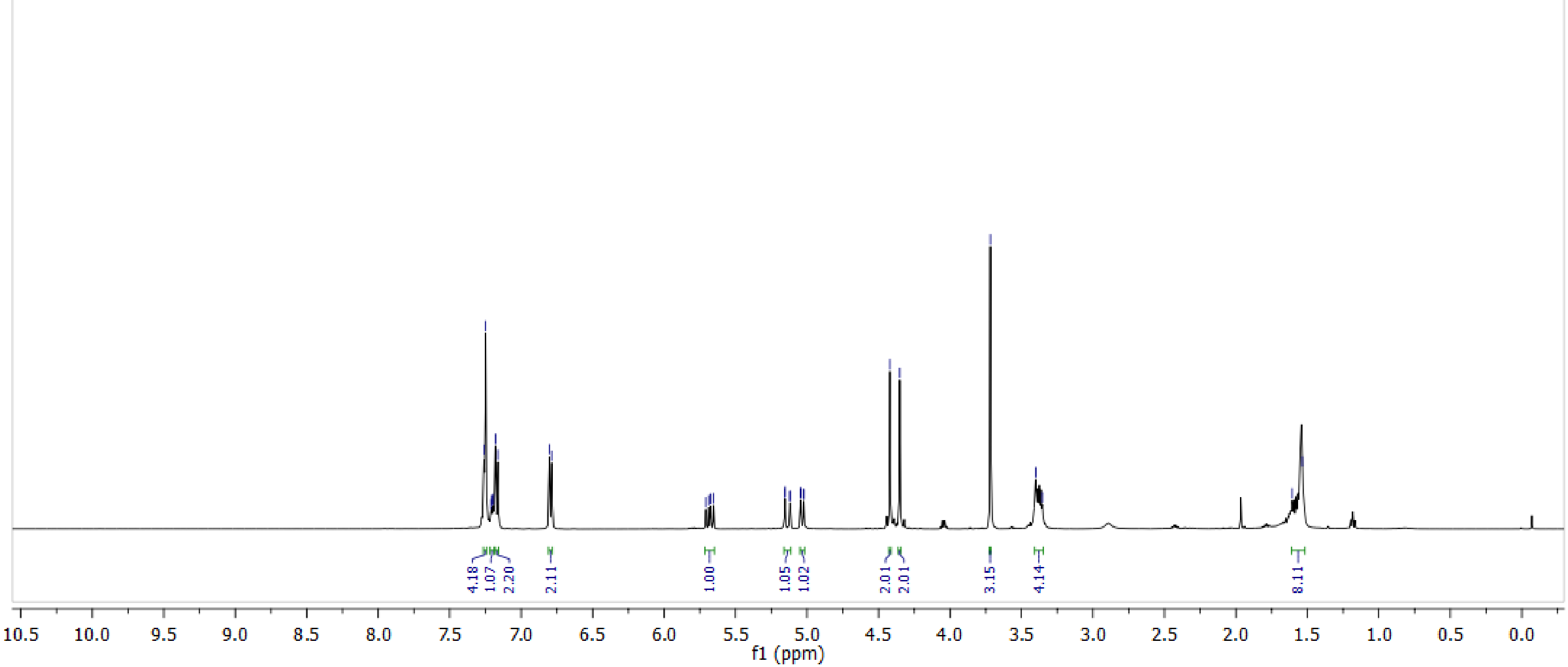

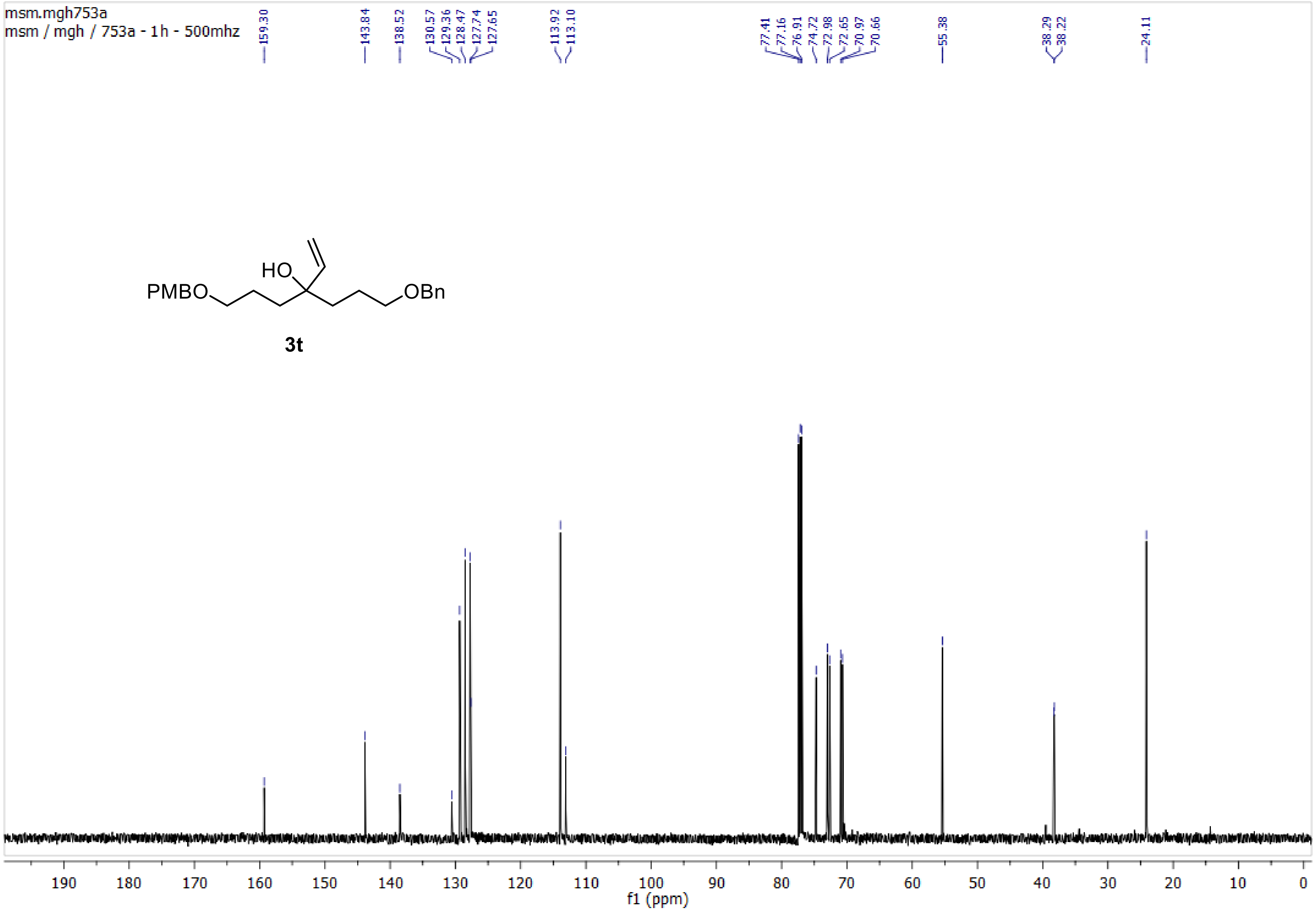
msm.mgh753a
msm / mgh / 753a - 1h - 500mhz
159.30
143.84
138.52
130.57
129.36
128.47
127.74
127.65
113.92
113.10
77.41
77.16
76.91
74.72
72.98
72.65
70.97
70.66
55.38
38.29
38.22
24.11
HO
PMBO
OBn
190 180 170 160 150 140 130 120 110 100 90 80 70 60 50 40 30 20 10 0
f1 (ppm)

3t

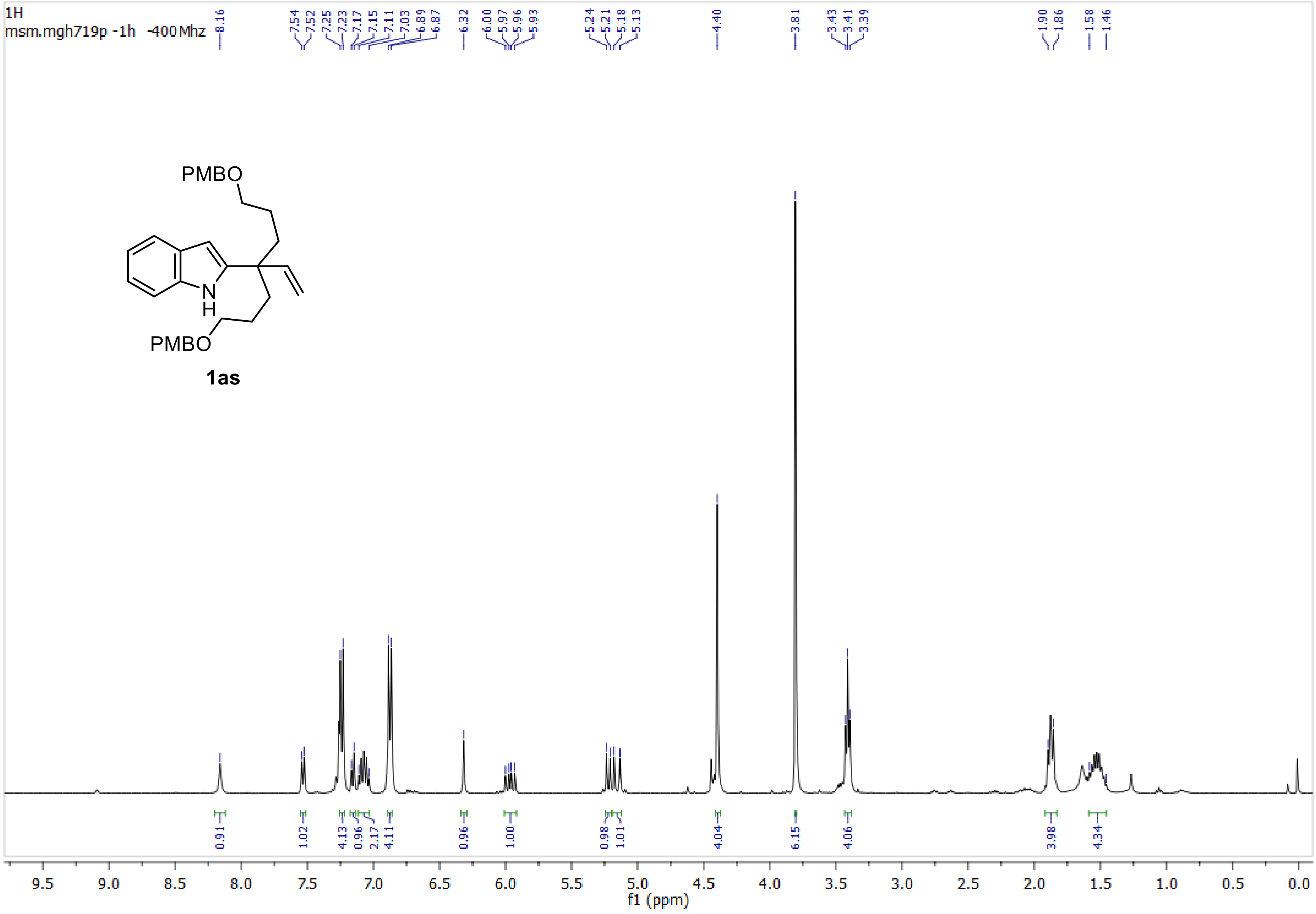
1H
msm.mgh719p -1h -400Mhz
PMBO
N
H
PMBO
1as
f1 (ppm)

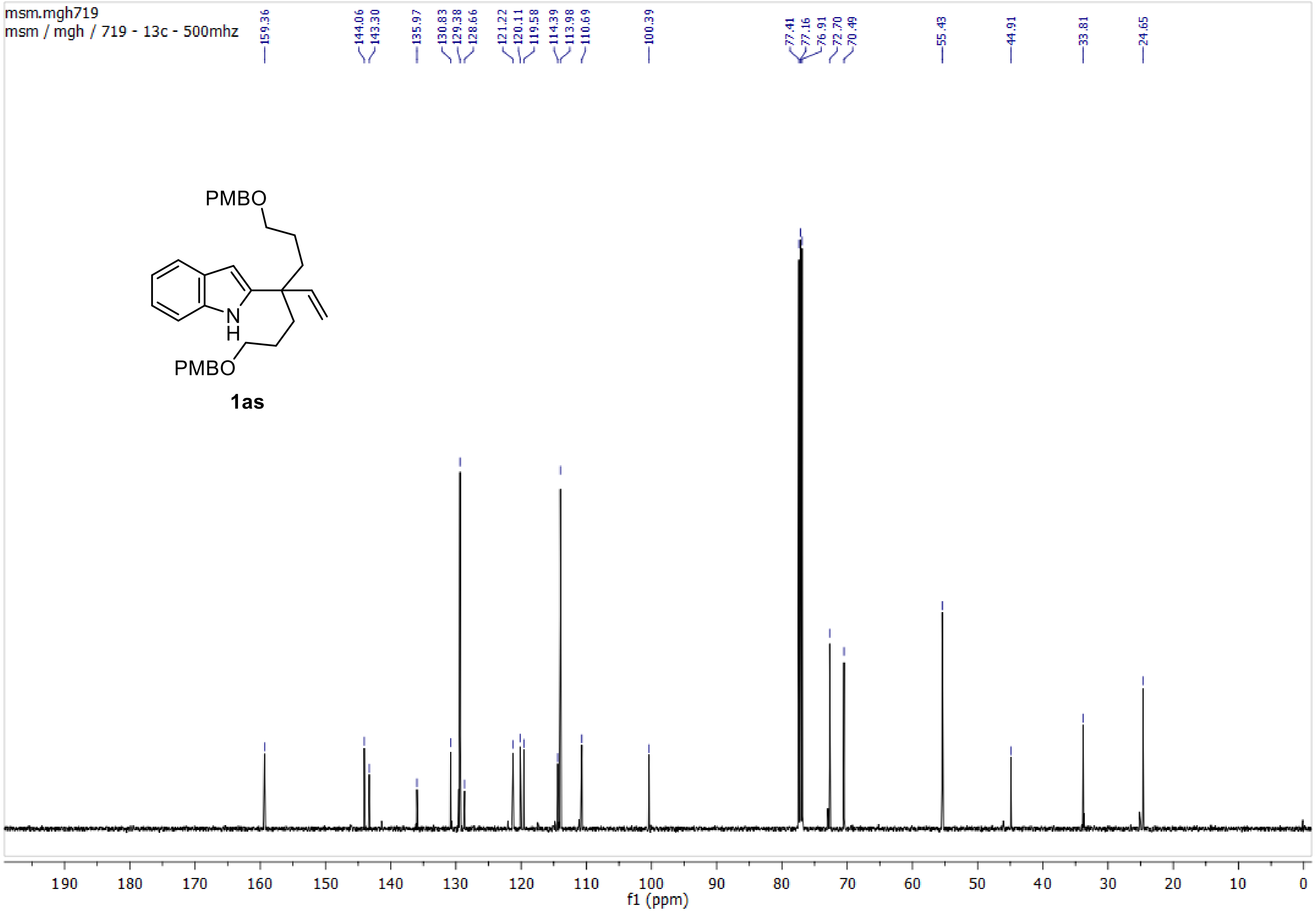
msm.mgh719
msm / mgh / 719 - 13c - 500mhz
159.36
144.06
143.30
135.97
130.83
129.38
128.66
121.22
120.11
119.58
114.39
113.98
110.69
100.39
77.41
77.16
76.91
72.70
70.49
55.43
44.91
33.81
24.65
PMBO
N
H
PMBO
1as
190
180
170
160
150
140
130
120
110
100
90
80
70
60
50
40
30
20
10
0
f1 (ppm)

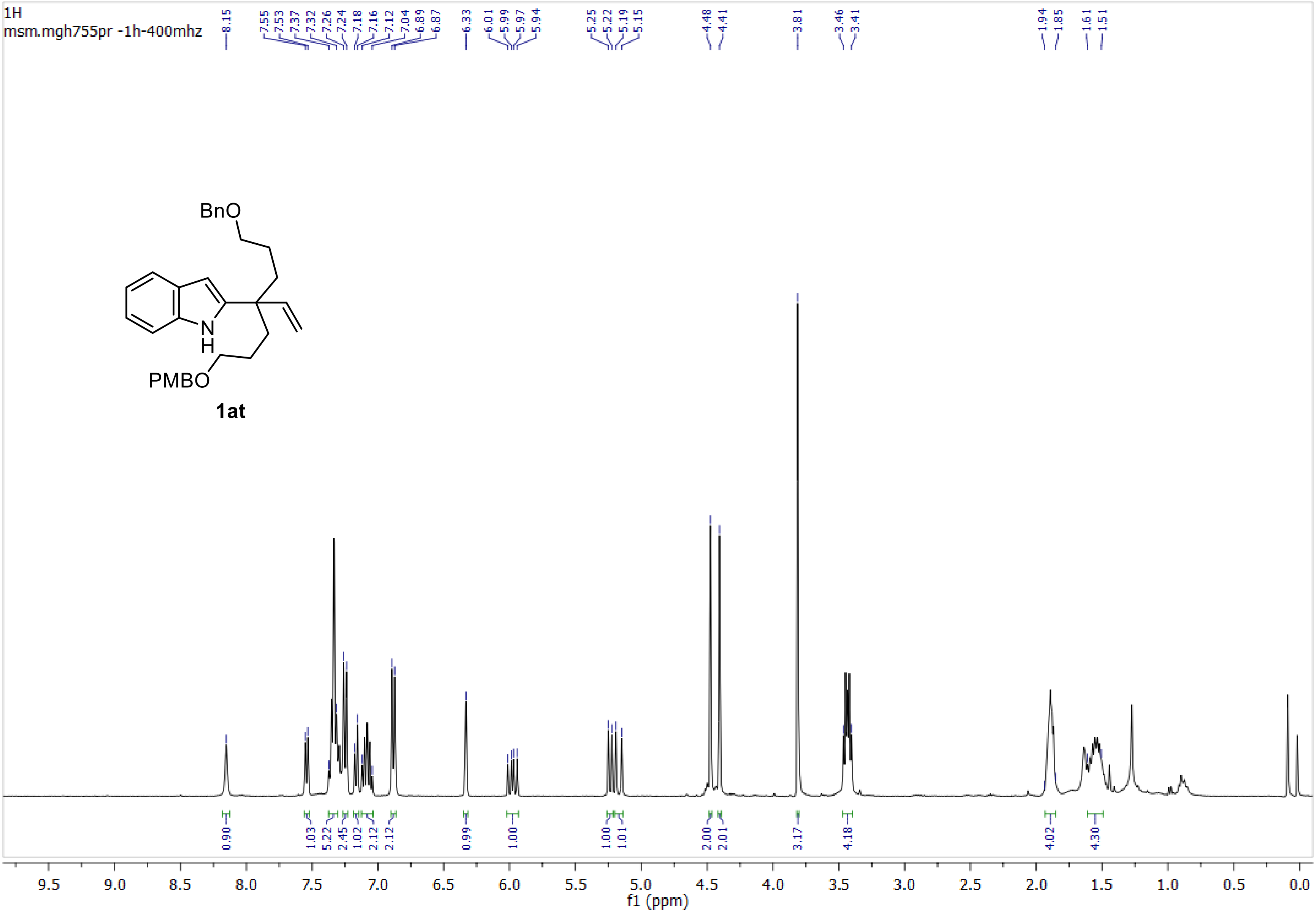
1H
msm.mgh755pr -1h-400mhz
8.15
7.55
7.53
7.37
7.32
7.26
7.24
7.18
7.16
7.12
7.04
6.89
6.87
6.33
6.01
5.99
5.97
5.94
5.25
5.22
5.19
5.15
4.48
4.41
3.81
3.46
3.41
1.94
1.85
1.61
1.51
BnO
N
H
PMBO
1at
0.90
1.03
5.22
2.45
1.02
2.12
2.12
0.99
1.00
1.00
1.01
2.00
2.01
3.17
4.18
4.02
4.30
9.5
9.0
8.5
8.0
7.5
7.0
6.5
6.0
5.5
5.0
4.5
4.0
3.5
3.0
2.5
2.0
1.5
1.0
0.5
0.0
f1 (ppm)

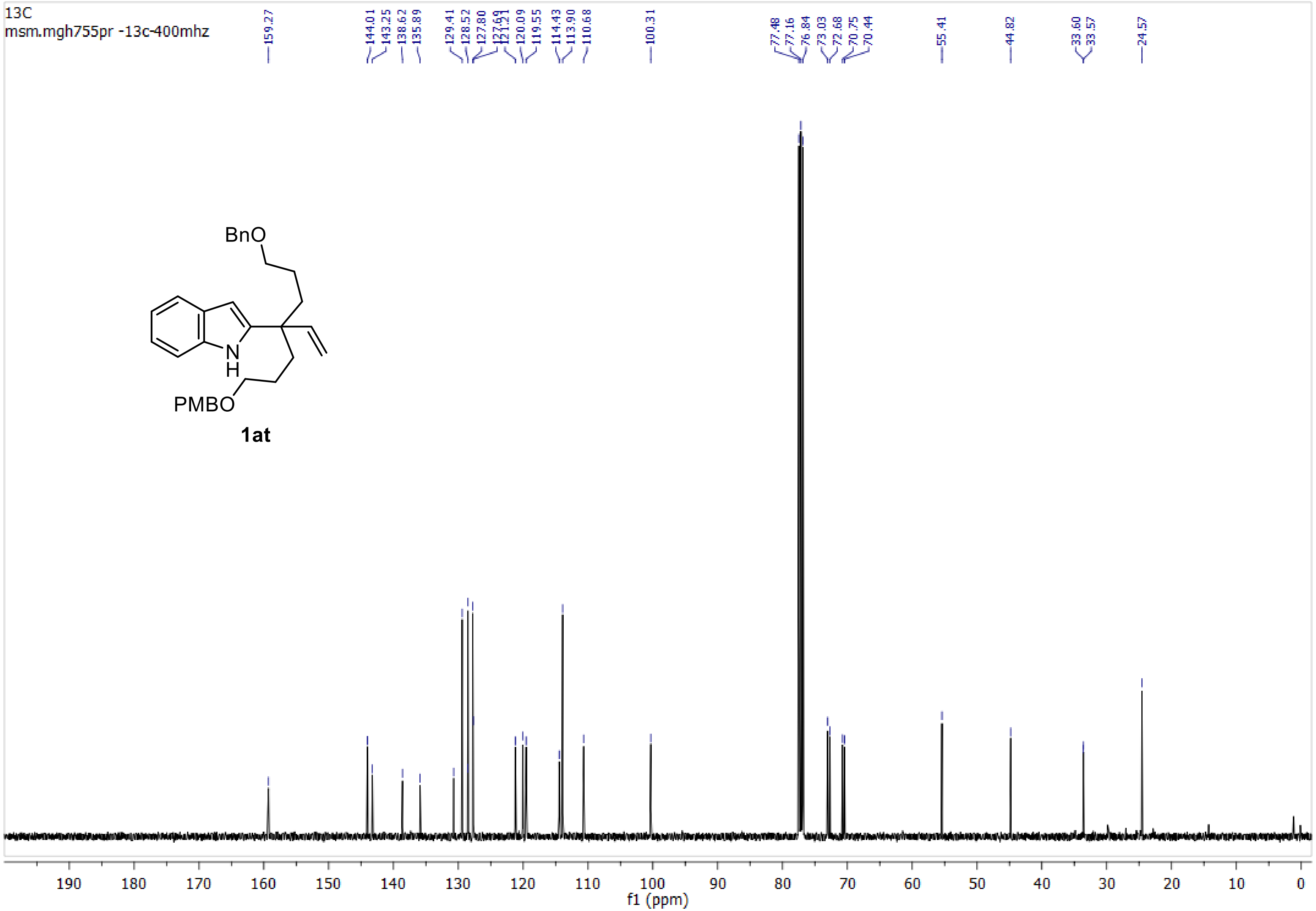
13C
msm.mgh755pr -13c-400mhz
159.27
144.01
143.25
138.62
135.89
129.41
128.52
127.80
121.21
120.09
119.55
114.43
113.90
110.68
100.31
77.48
77.16
76.84
73.03
72.68
70.75
70.44
55.41
44.82
33.60
33.57
24.57
BnO
N
H
PMBO
1at
190
180
170
160
150
140
130
120
110
100
90
80
70
60
50
40
30
20
10
0
f1 (ppm)

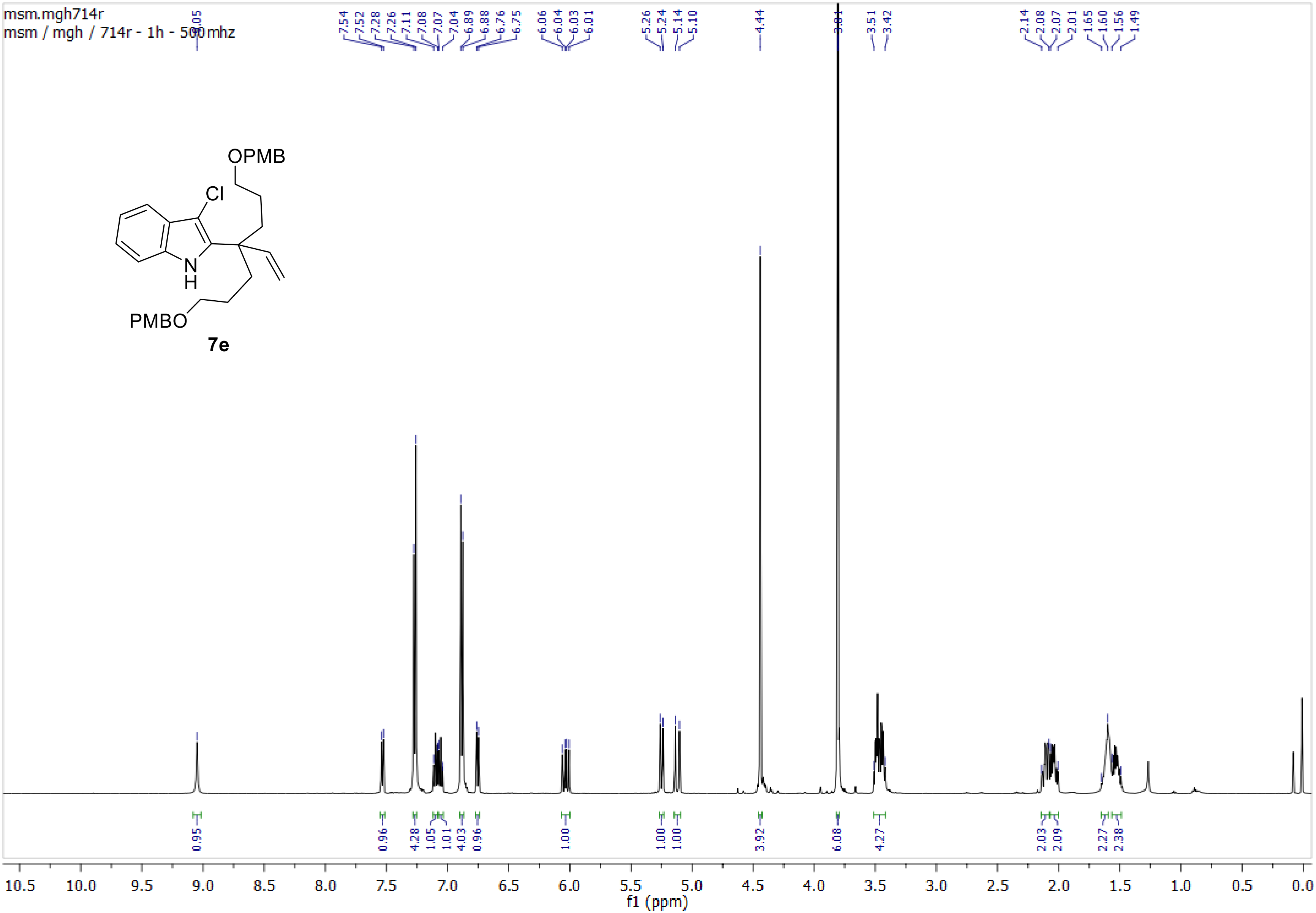
msm.mgh714r
msm / mgh / 714r - 1h - 500mhz
OPMB
Cl
N
H
PMBO
7e
9.05
7.54 7.52 7.28 7.26 7.11 7.08 7.07 7.04 6.89 6.88 6.76 6.75
6.06 6.04 6.03 6.01
5.26 5.24 5.14 5.10
4.44
3.81
3.51 3.42
2.14 2.08 2.07 2.01 1.65 1.60 1.56 1.49
0.95
0.96 4.28 1.05 1.01 4.03 0.96
1.00
1.00 1.00
3.92
6.08
4.27
2.03 2.09
2.27 2.38
10.5 10.0 9.5 9.0 8.5 8.0 7.5 7.0 6.5 6.0 5.5 5.0 4.5 4.0 3.5 3.0 2.5 2.0 1.5 1.0 0.5 0.0
f1 (ppm)

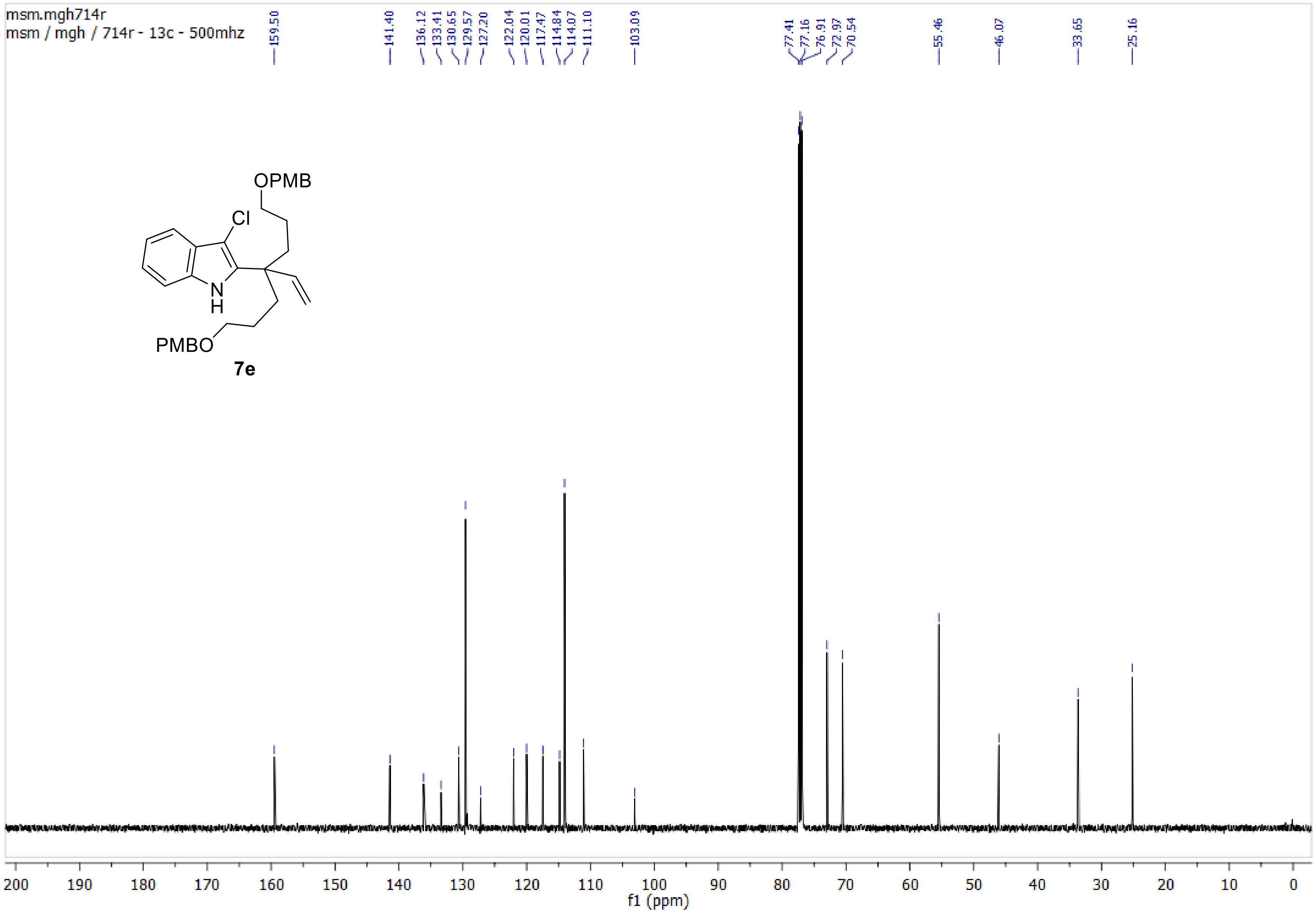

msm.mgh714r
msm / mgh / 714r - 13c - 500mhz
159.50
141.40
136.12
133.41
130.65
129.57
127.20
122.04
120.01
117.47
114.84
114.07
111.10
103.09
77.41
77.16
76.91
72.97
70.54
55.46
46.07
33.65
25.16
OPMB
Cl
N
H
PMBO
7e
f1 (ppm)

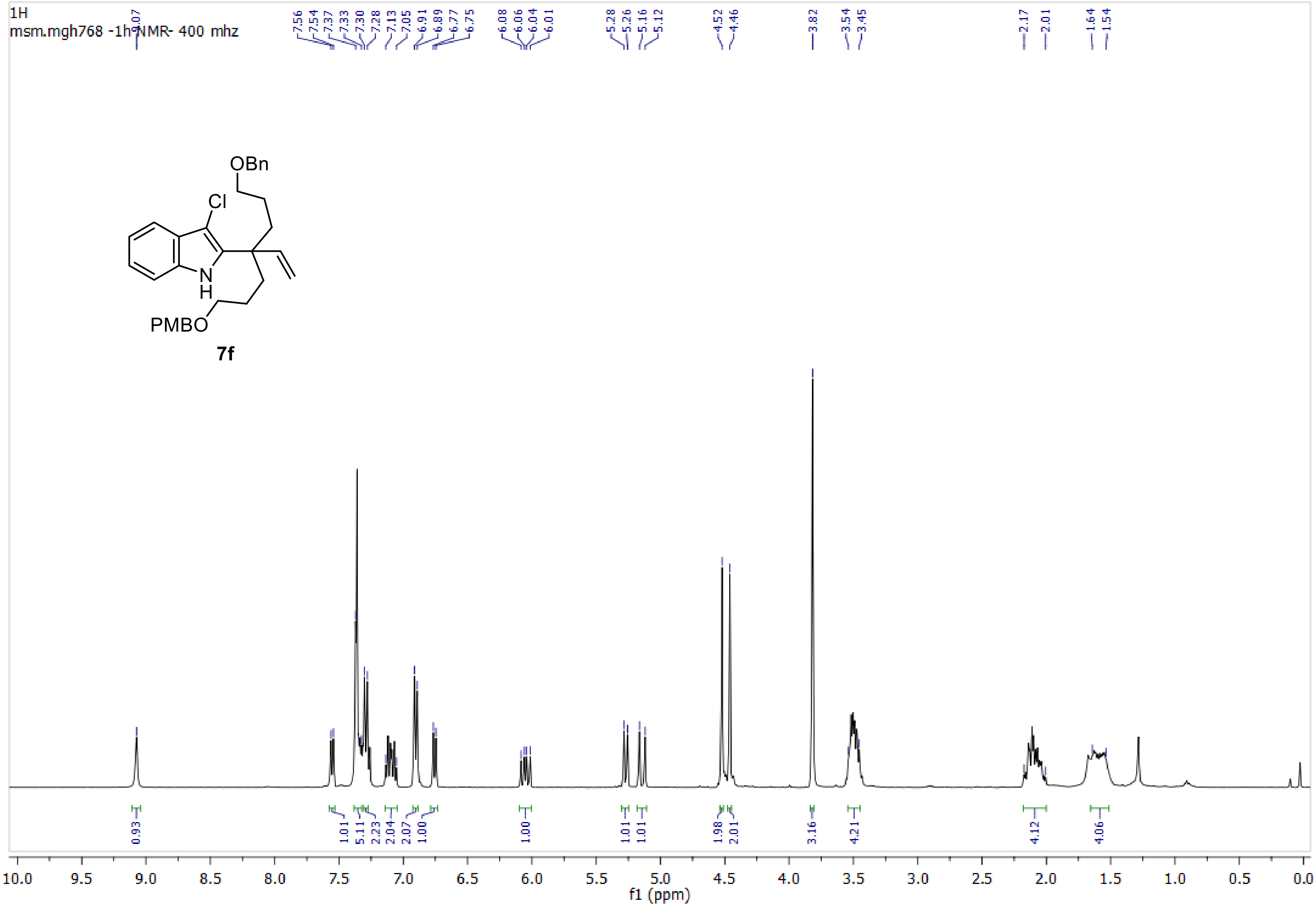
1H
msm.mgh768 -1h NMR- 400 mhz
9.07
7.56
7.54
7.37
7.33
7.30
7.28
7.13
7.05
6.91
6.89
6.77
6.75
6.08
6.06
6.04
6.01
5.28
5.26
5.16
5.12
4.52
4.46
3.82
3.54
3.45
2.17
2.01
1.64
1.54
OBn
Cl
N
H
PMBO
7f
0.93
1.01
5.11
2.23
2.04
2.07
1.00
1.00
1.01
1.01
1.98
2.01
3.16
4.21
4.12
4.06
10.0
9.5
9.0
8.5
8.0
7.5
7.0
6.5
6.0
5.5
5.0
4.5
4.0
3.5
3.0
2.5
2.0
1.5
1.0
0.5
0.0
f1 (ppm)

13C
msm / mgh / 768 - 1h - 500mhz

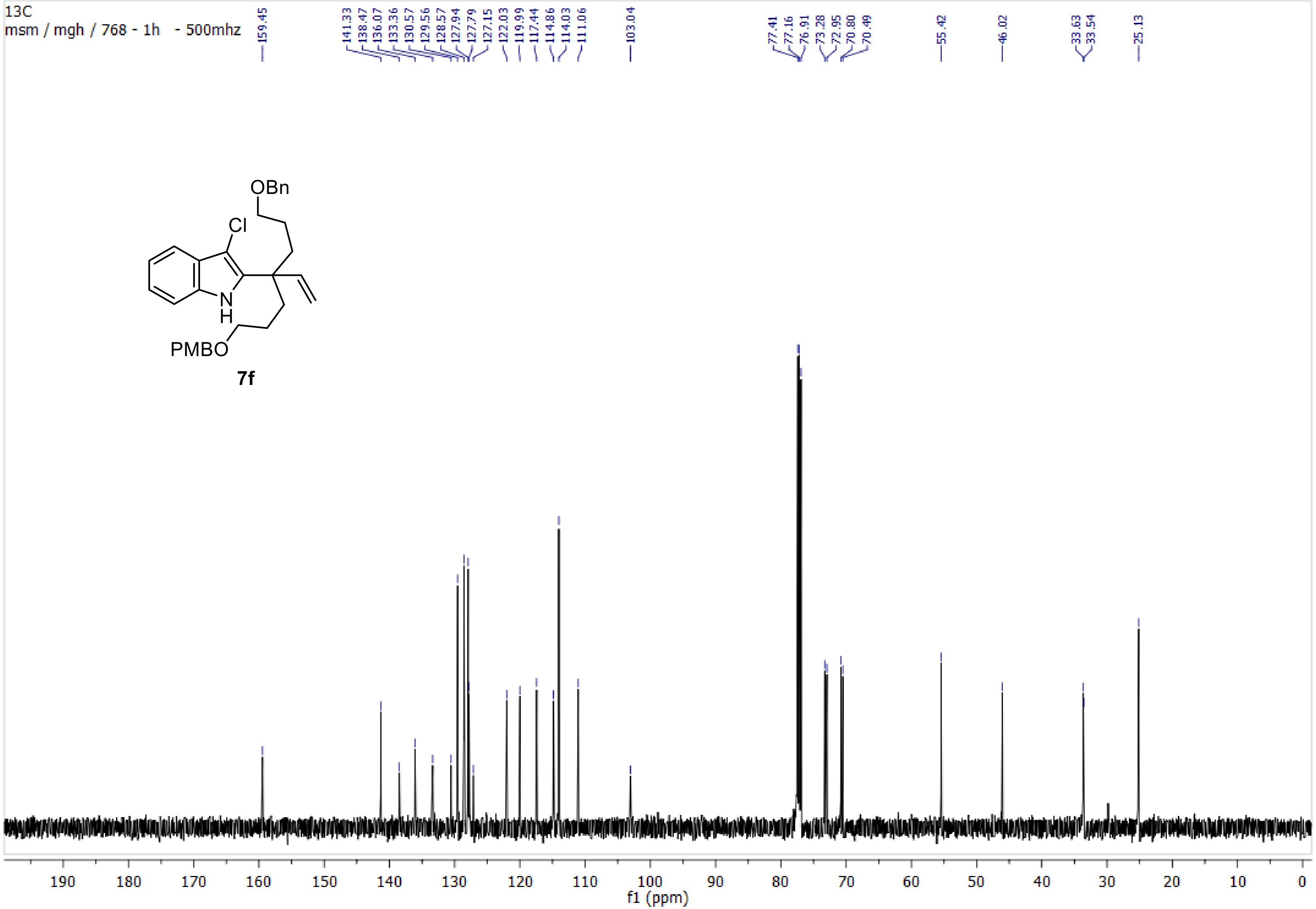

7f

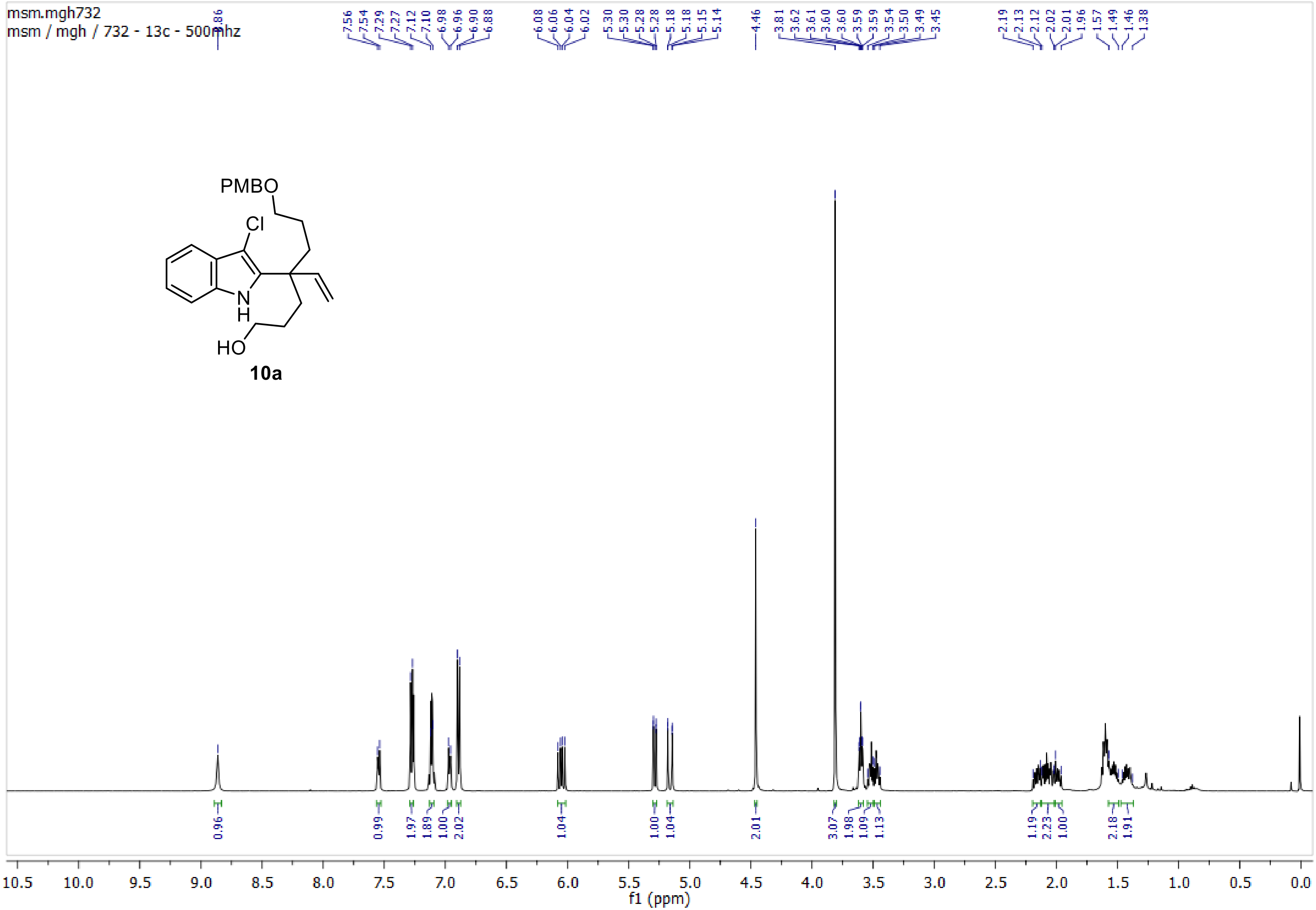
msm.mgh732
msm / mgh / 732 - 13c - 500mhz
8.86
7.56 7.54 7.29 7.27 7.12 7.10 6.98 6.96 6.90 6.88
6.08 6.06 6.04 6.02
5.30 5.30 5.28 5.28 5.18 5.18 5.15 5.14
4.46
3.81 3.62 3.61 3.60 3.59 3.59 3.54 3.50 3.49 3.45
2.19 2.13 2.12 2.02 2.01 1.96 1.57 1.49 1.46 1.38
PMBO
Cl
N
H
HO
10a
0.96
0.99 1.97 1.89 1.00 2.02
1.04
1.00 1.04
2.01
3.07 1.98 1.09 1.13
1.19 2.23 1.00
2.18 1.91
10.5 10.0 9.5 9.0 8.5 8.0 7.5 7.0 6.5 6.0 5.5 5.0 4.5 4.0 3.5 3.0 2.5 2.0 1.5 1.0 0.5 0.0
f1 (ppm)

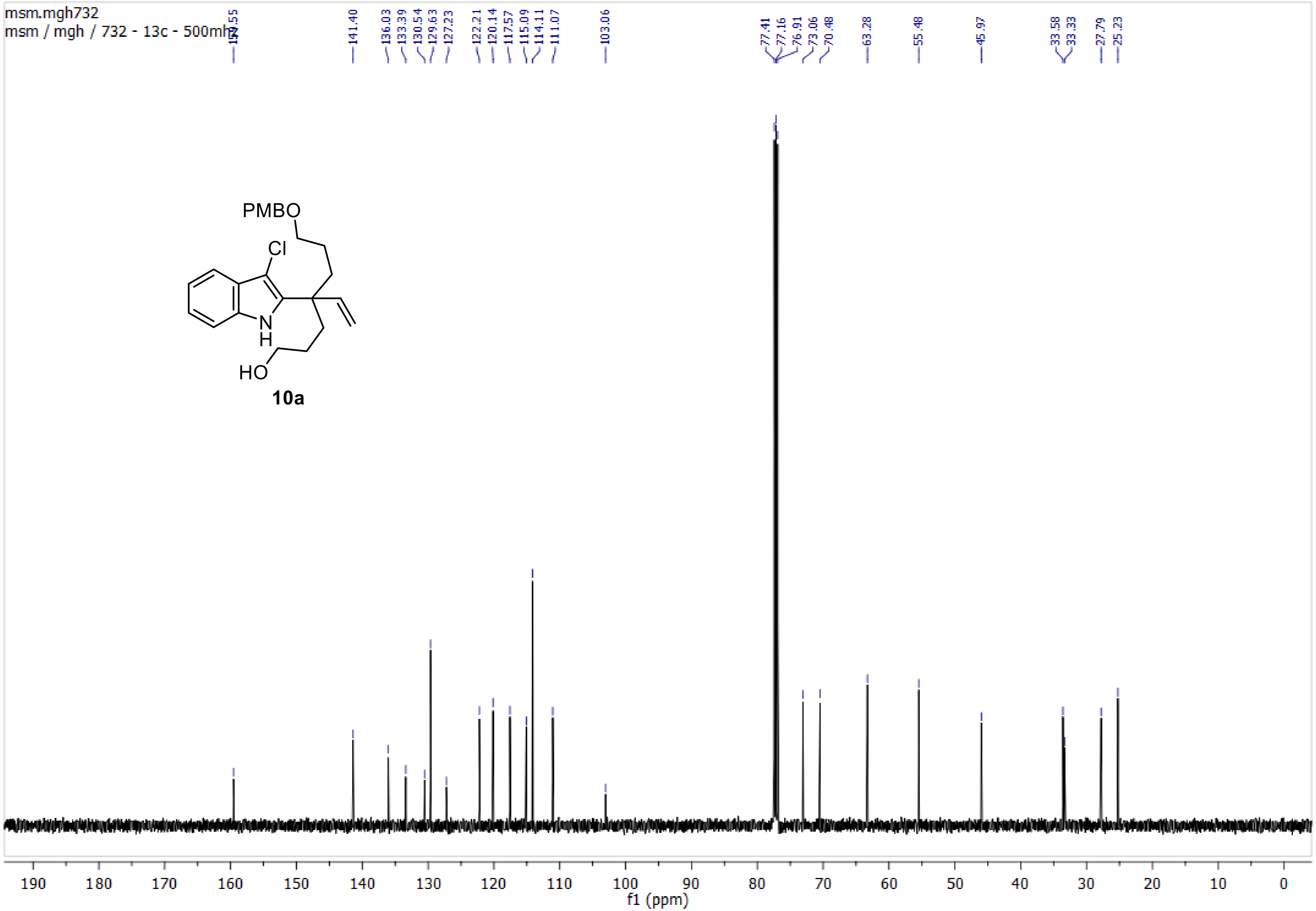
msm.mgh732
msm / mgh / 732 - 13c - 500mhz
141.40
136.03
133.39
130.54
129.63
127.23
122.21
120.14
117.57
115.09
114.11
111.07
103.06
77.41
77.16
76.91
73.06
70.48
63.28
55.48
45.97
33.58
33.33
27.79
25.23
PMBO
Cl
N
H
HO
190 180 170 160 150 140 130 120 110 100 90 80 70 60 50 40 30 20 10 0
f1 (ppm)

10a

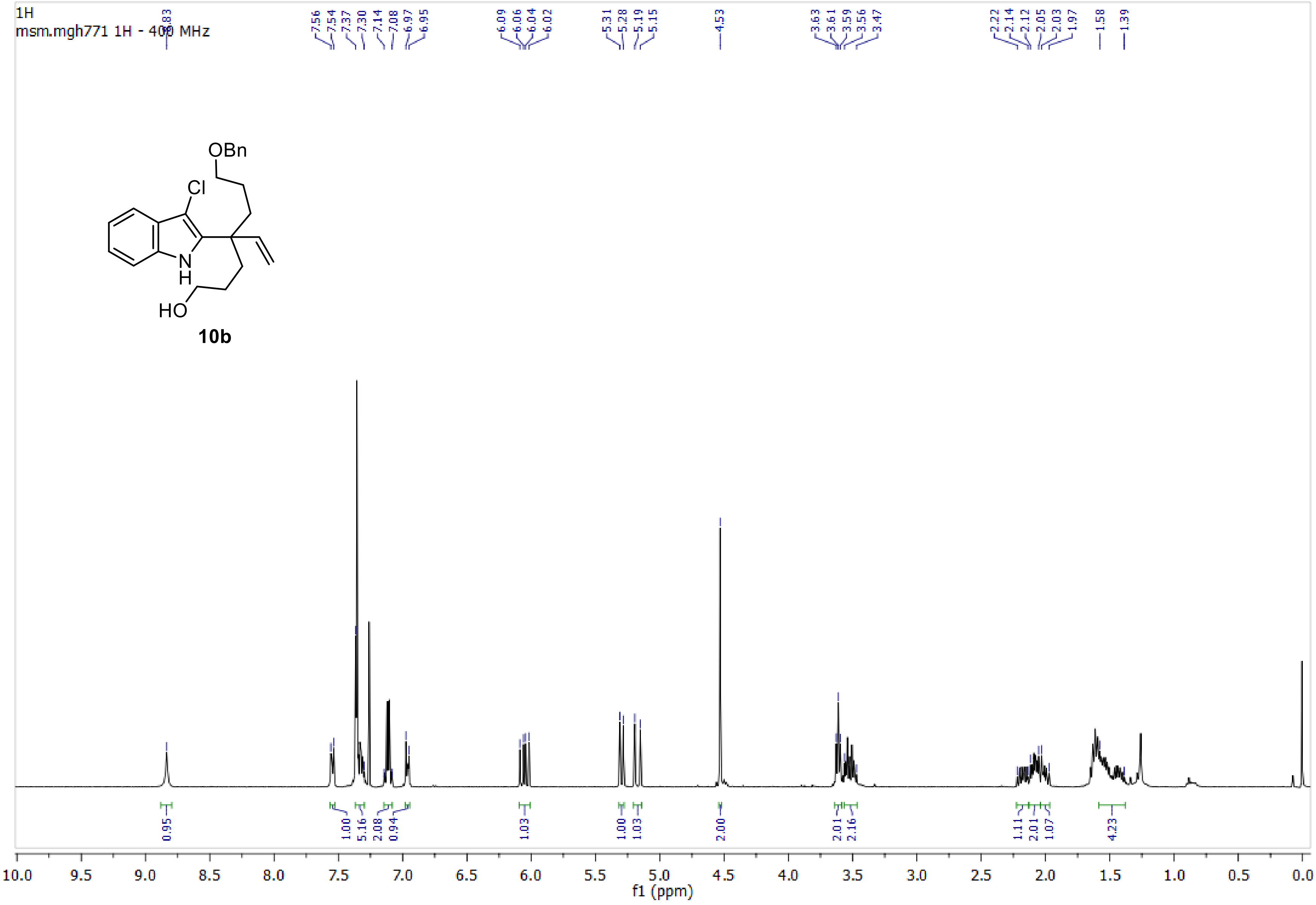
1H
msm.mgh771 1H - 400 MHz
OBn
Cl
N
H
HO
10b
f1 (ppm)

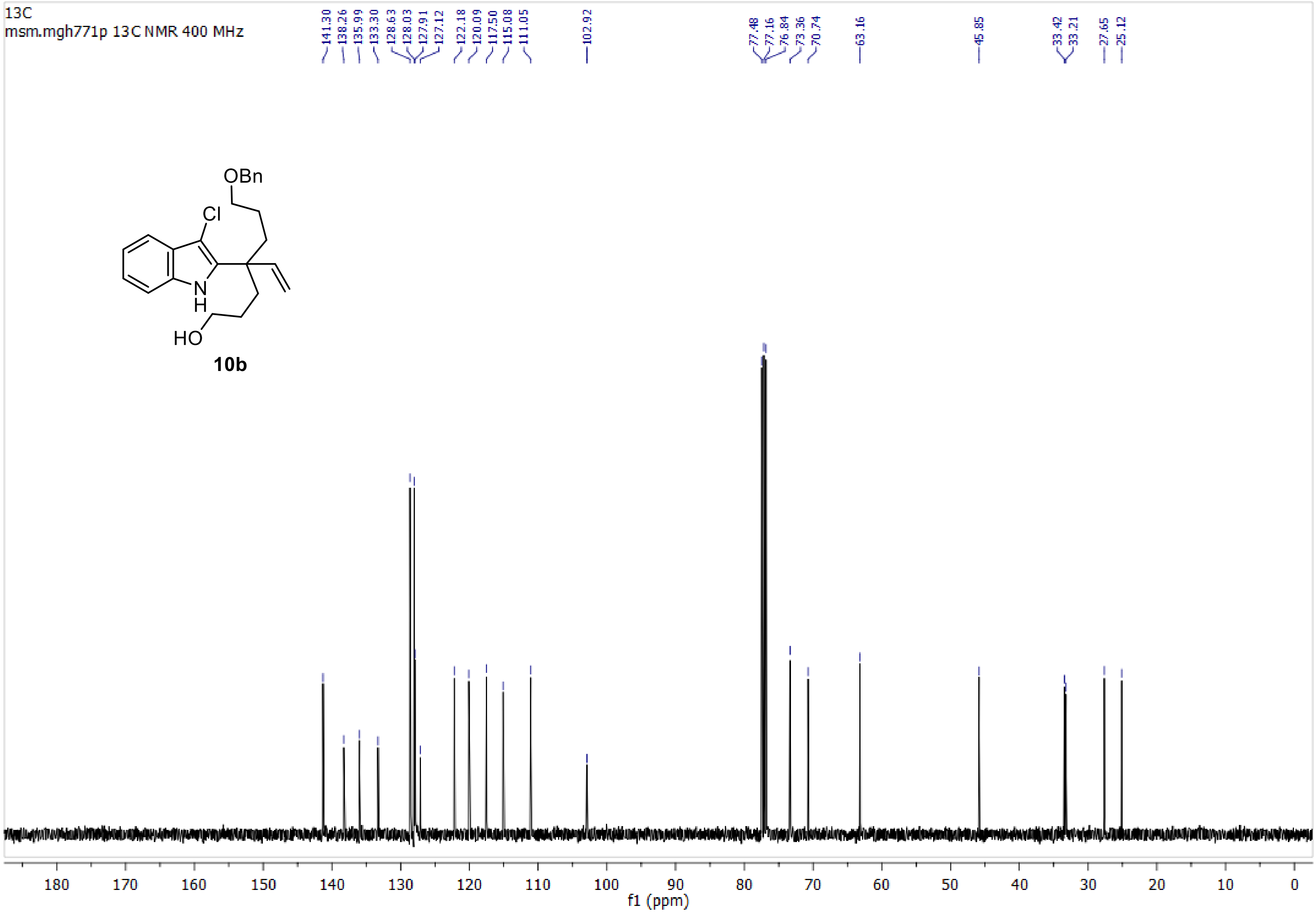
13C
msm.mgh771p 13C NMR 400 MHz
141.30
138.26
135.99
133.30
128.63
128.03
127.91
127.12
122.18
120.09
117.50
115.08
111.05
102.92
77.48
77.16
76.84
73.36
70.74
63.16
45.85
33.42
33.21
27.65
25.12
OBn
Cl
N
H
HO
10b
180 170 160 150 140 130 120 110 100 90 80 70 60 50 40 30 20 10 0
f1 (ppm)

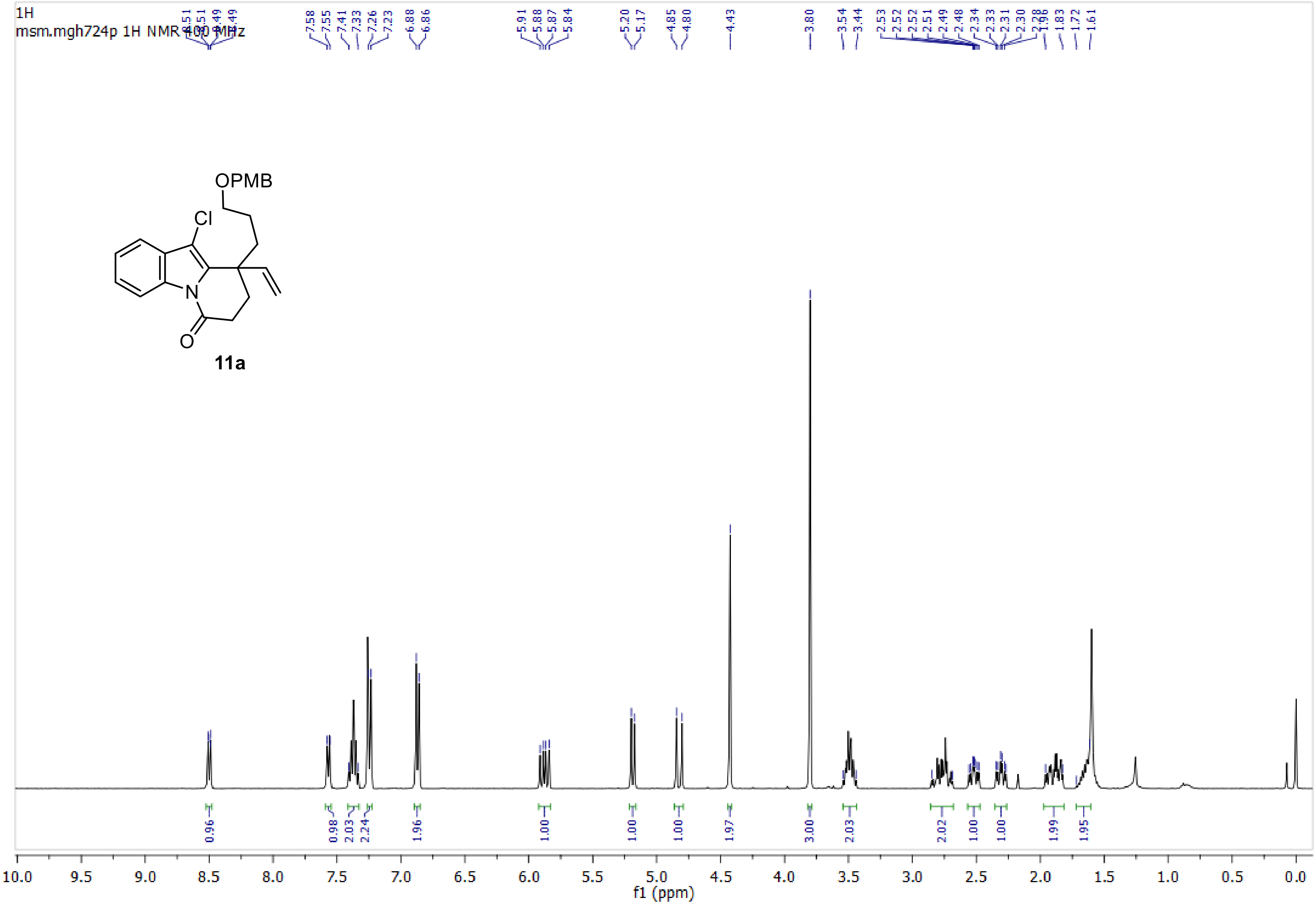

1H
msm.mgh724p 1H NMR 400 MHz
OPMB
Cl
N
O
11a
f1 (ppm)

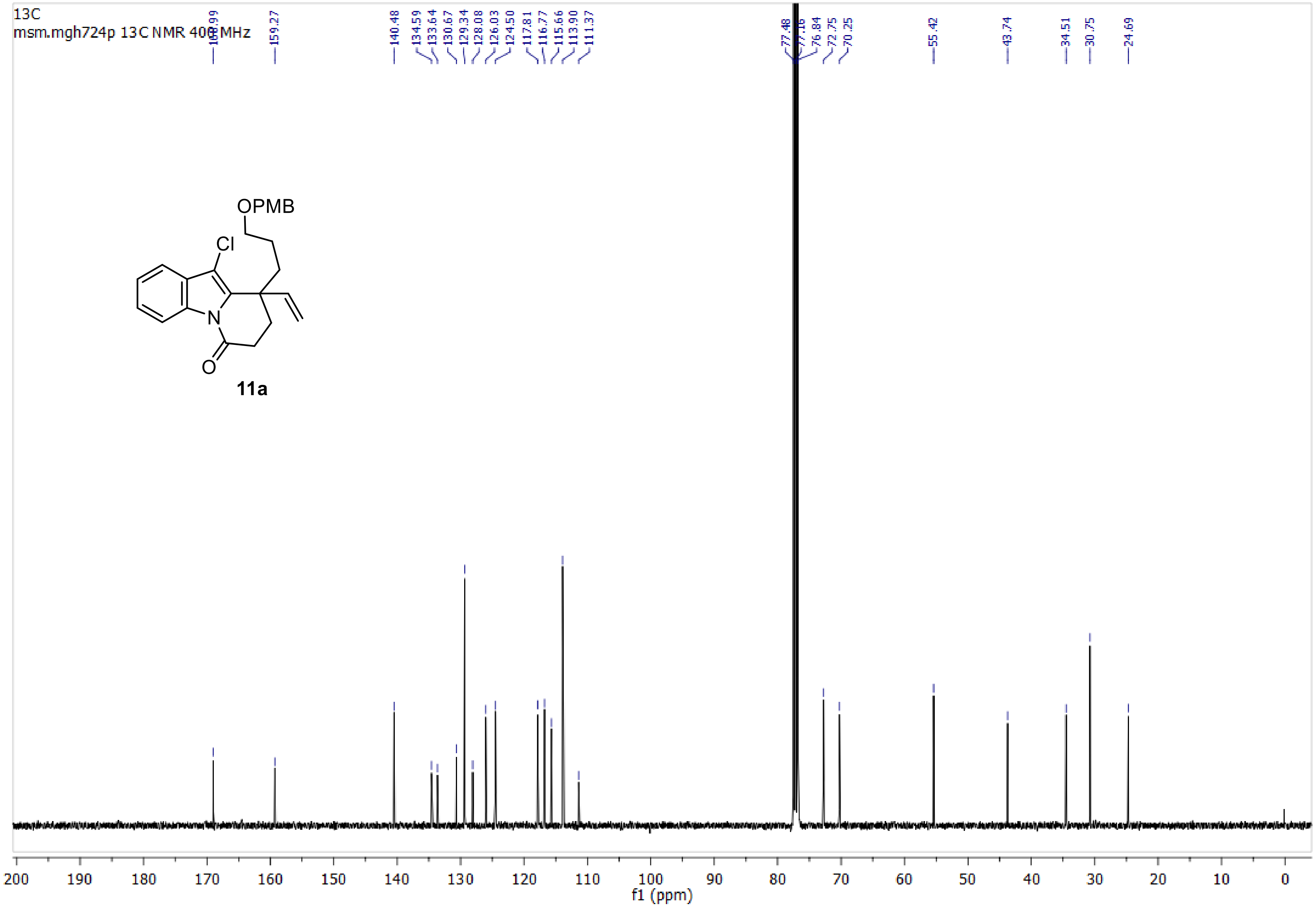

13C
msm.mgh724p 13C NMR 400 MHz
168.99
159.27
140.48
134.59
133.64
130.67
129.34
128.08
126.03
124.50
117.81
116.77
115.66
113.90
111.37
77.48
77.16
76.84
72.75
70.25
55.42
43.74
34.51
30.75
24.69
OPMB
Cl
N
O
11a
200 190 180 170 160 150 140 130 120 110 100 90 80 70 60 50 40 30 20 10 0
f1 (ppm)

msm.mgh776r
msm / mgh / 776 r - 13c - 500mhz

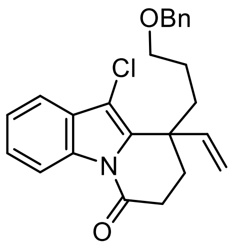

11b

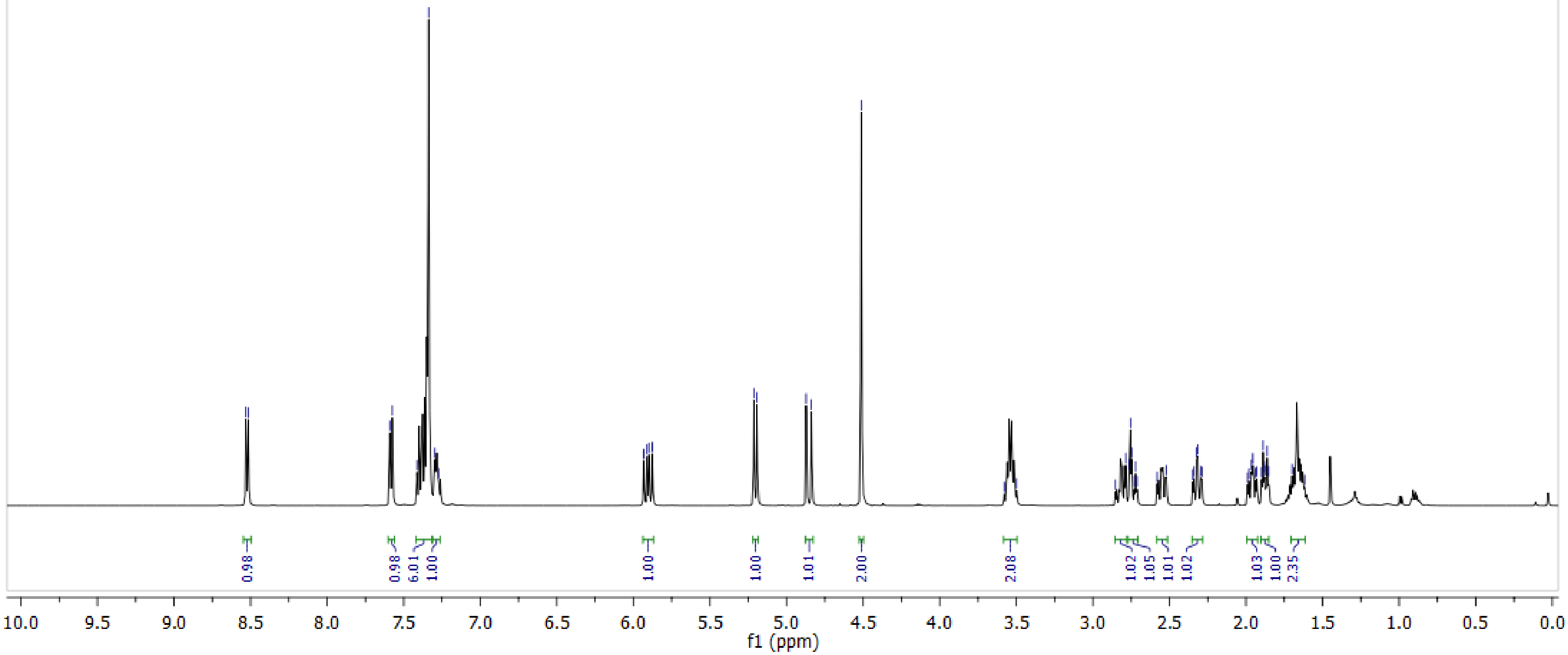

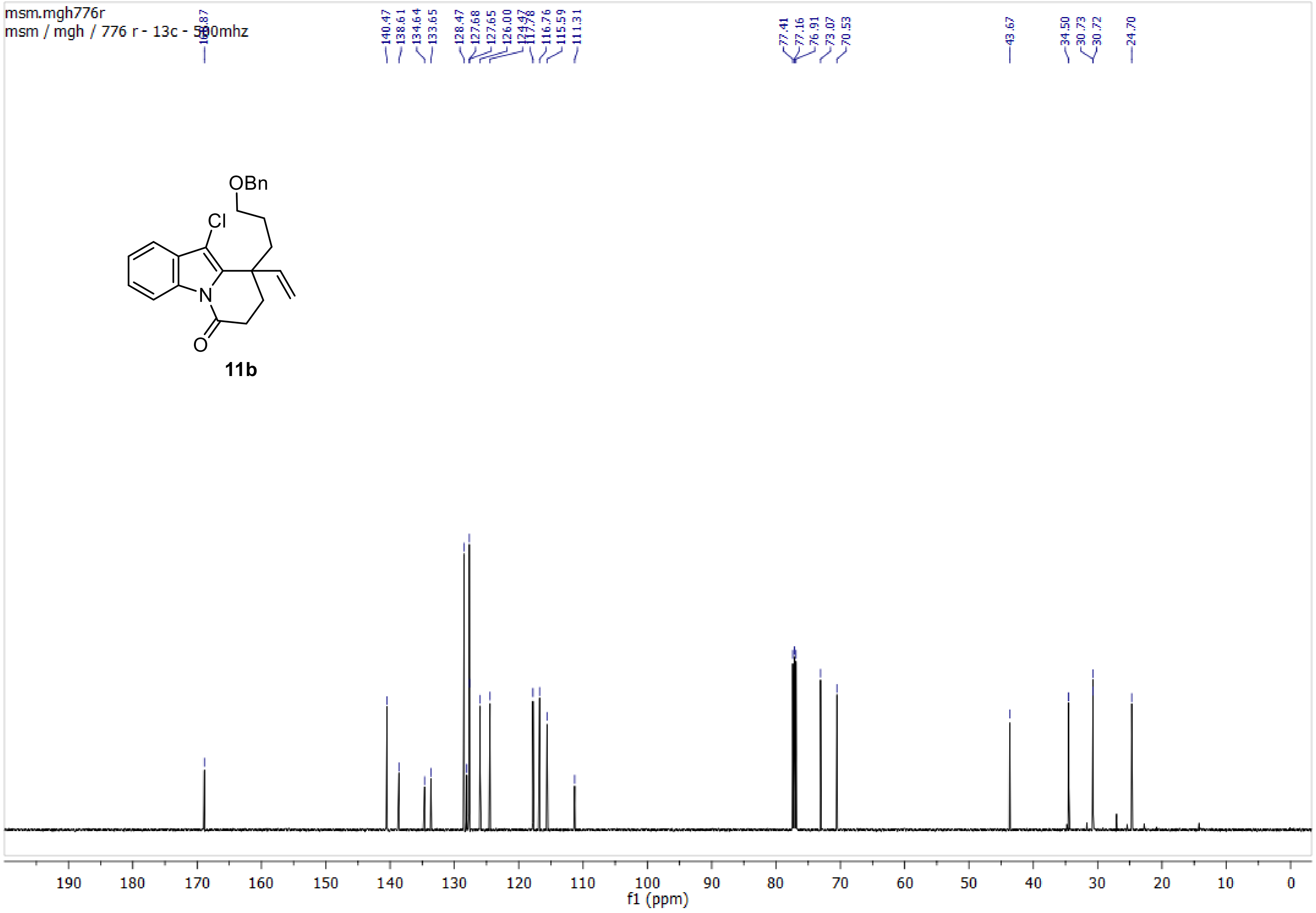
msm.mgh776r
msm / mgh / 776 r - 13c - 500mhz
140.47
138.61
134.64
133.65
128.47
127.68
127.65
126.00
124.47
116.76
115.59
111.31
77.41
77.16
76.91
73.07
70.53
43.67
34.50
30.73
30.72
24.70
OBn
Cl
N
O
11b
f1 (ppm)

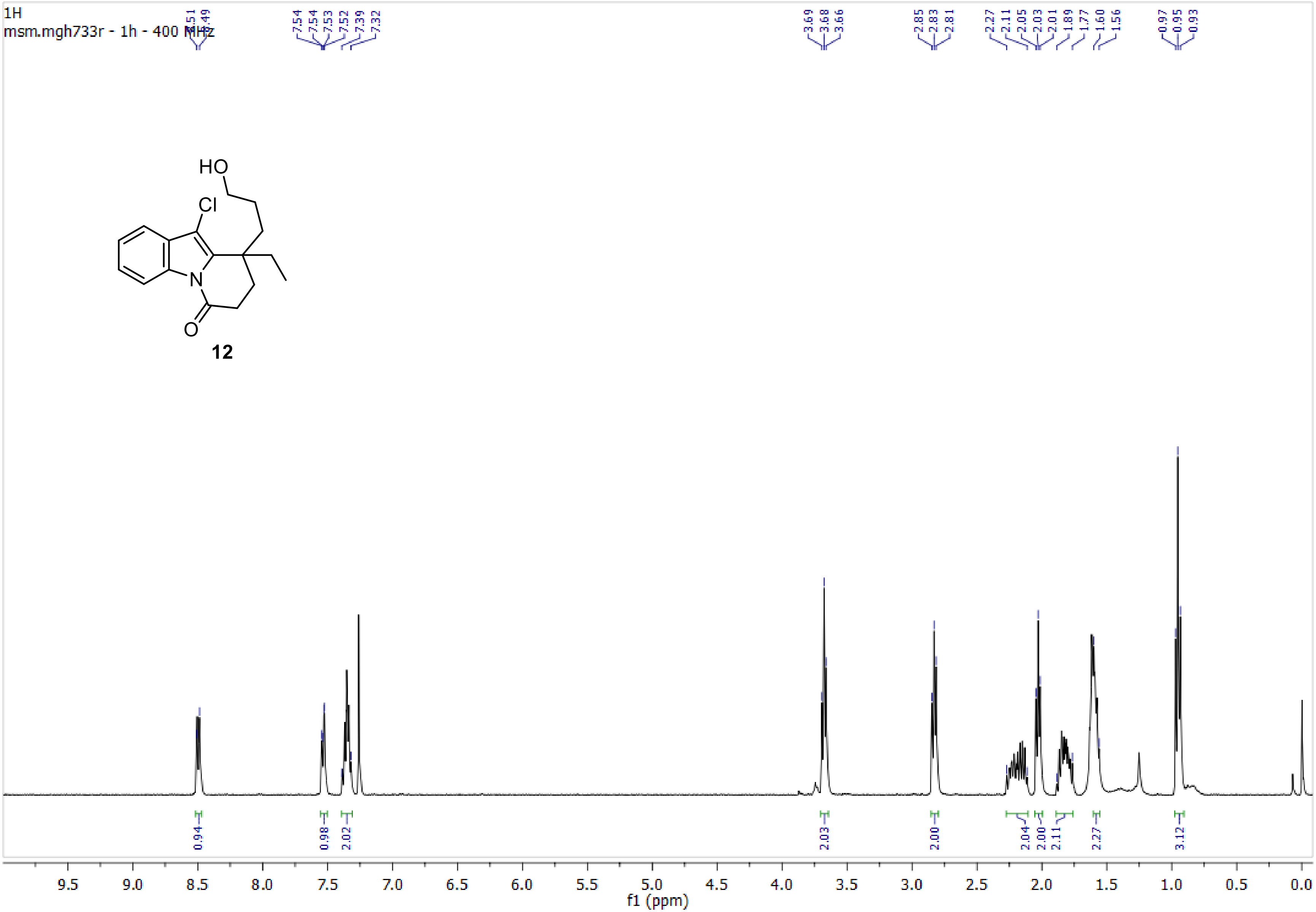
1H
msm.mgh733r - 1h - 400 MHz
8.51
8.49
7.54
7.54
7.53
7.52
7.39
7.32
3.69
3.68
3.66
2.85
2.83
2.81
2.27
2.11
2.05
2.03
2.01
1.89
1.77
1.60
1.56
0.97
0.95
0.93
HO
Cl
N
O
12
0.94
0.98
2.02
2.03
2.00
2.04
2.00
2.11
2.27
3.12
9.5
9.0
8.5
8.0
7.5
7.0
6.5
6.0
5.5
5.0
4.5
4.0
3.5
3.0
2.5
2.0
1.5
1.0
0.5
0.0
f1 (ppm)

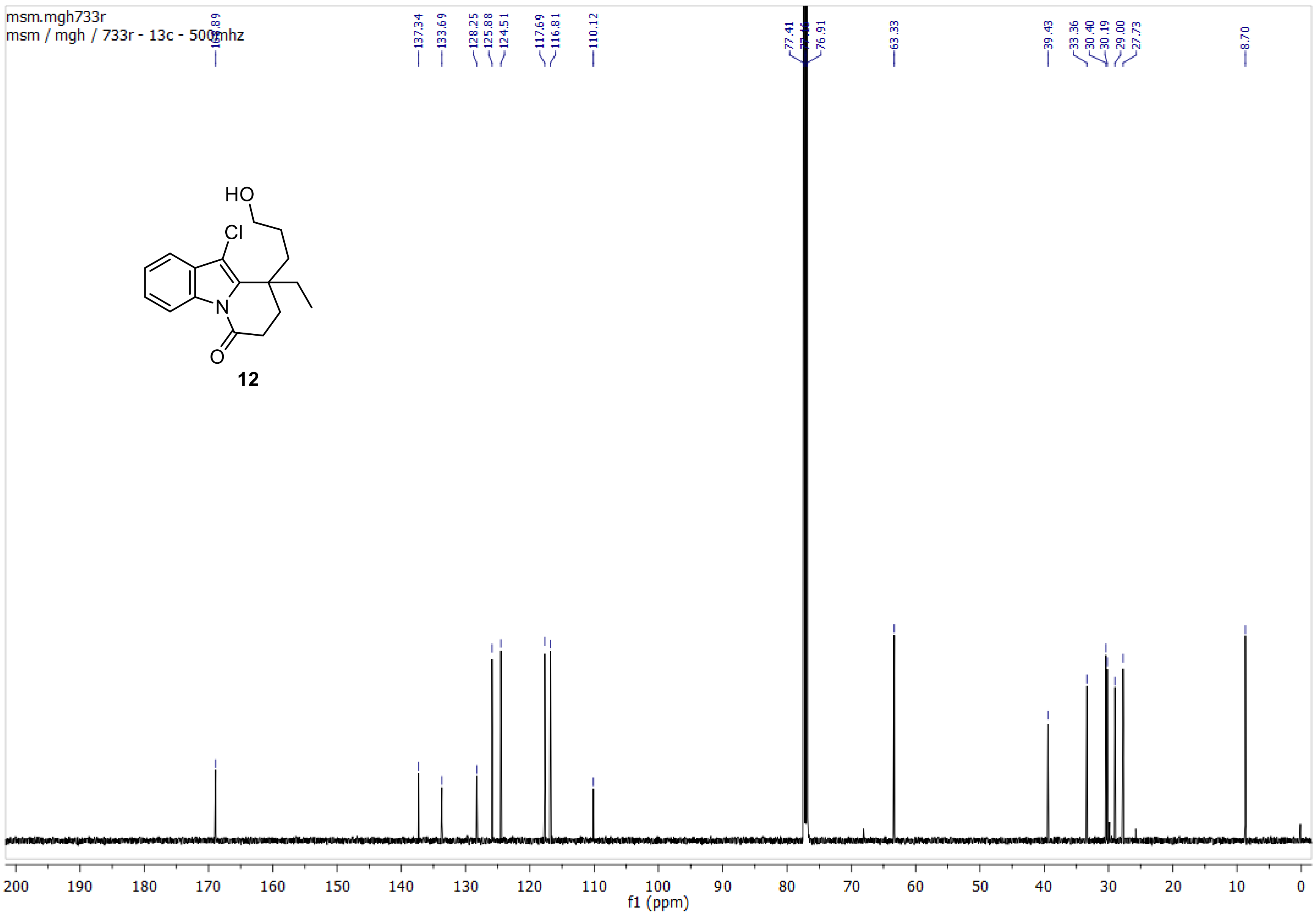
msm.mgh733r
msm / mgh / 733r - 13c - 500mhz
137.34
133.69
128.25
125.88
124.51
117.69
116.81
110.12
77.41
76.91
63.33
39.43
33.36
30.40
30.19
29.00
27.73
8.70
HO
Cl
N
O
12
f1 (ppm)

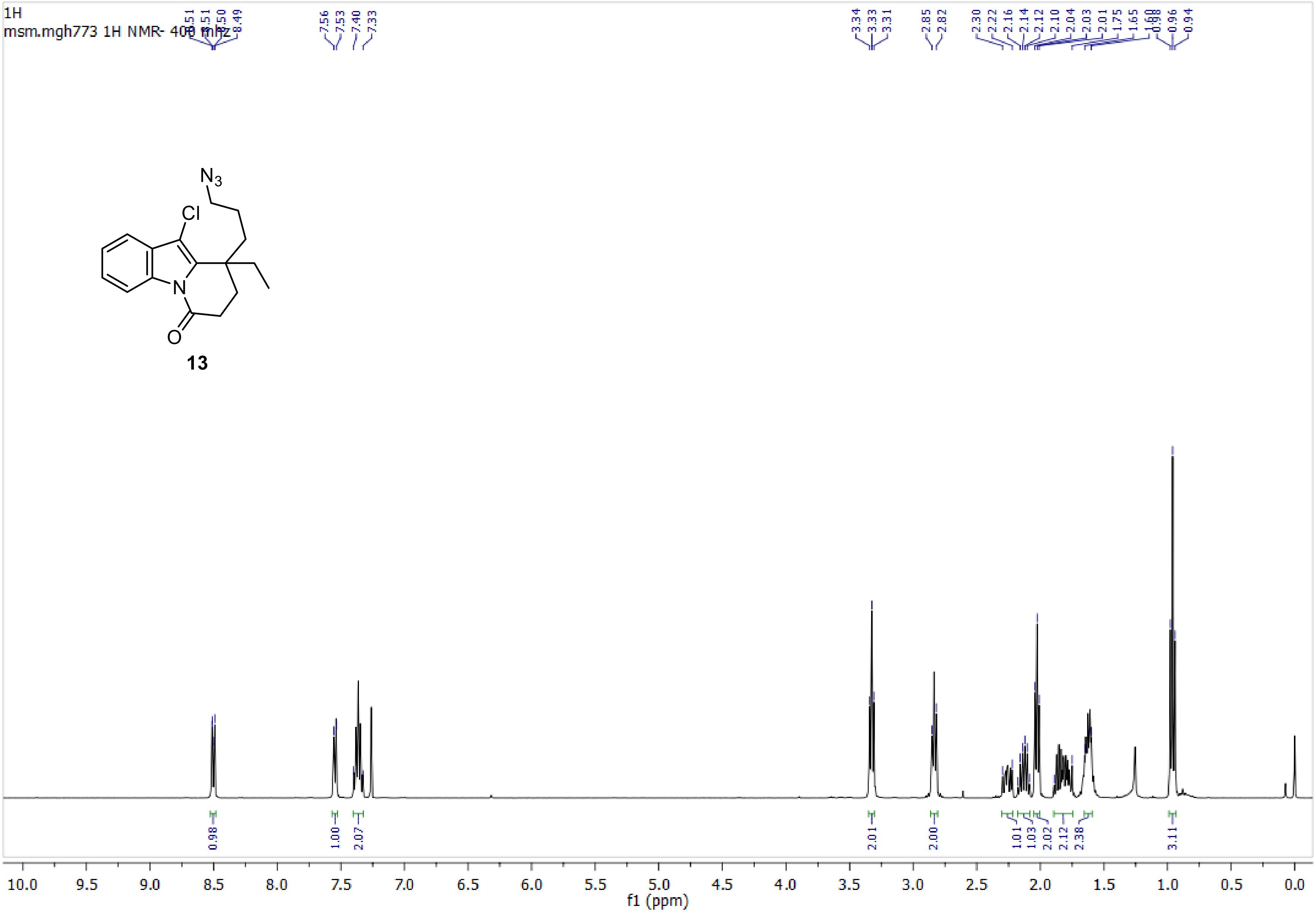
1H
msm.mgh773 1H NMR- 400 mhz
N3
Cl
N
O
13
f1 (ppm)

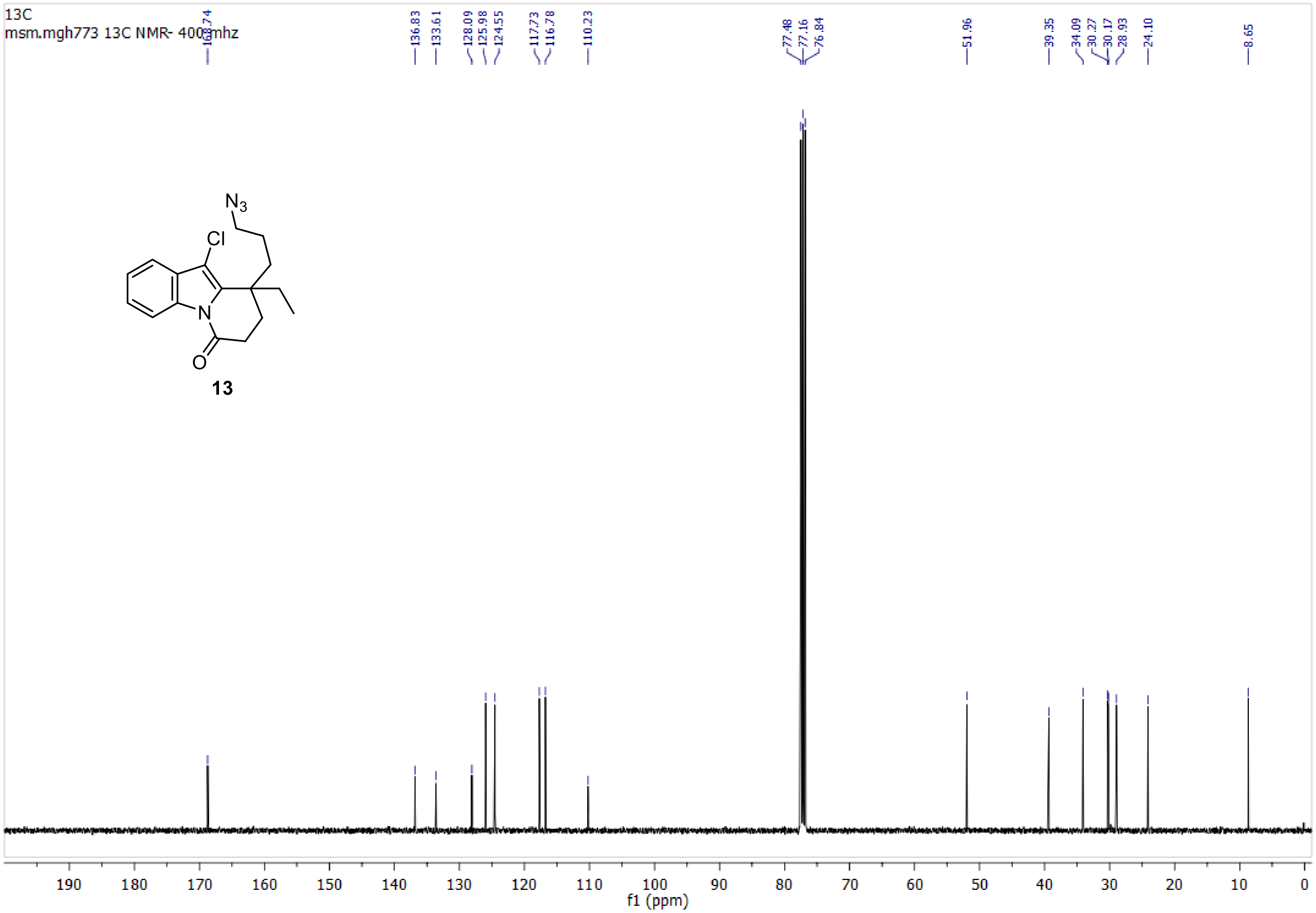
13C
msm.mgh773 13C NMR- 400 mhz
136.83
133.61
128.09
125.98
124.55
117.73
116.78
110.23
77.48
77.16
76.84
51.96
39.35
34.09
30.27
30.17
28.93
24.10
8.65
N3
Cl
N
O
13
190 180 170 160 150 140 130 120 110 100 90 80 70 60 50 40 30 20 10 0
f1 (ppm)

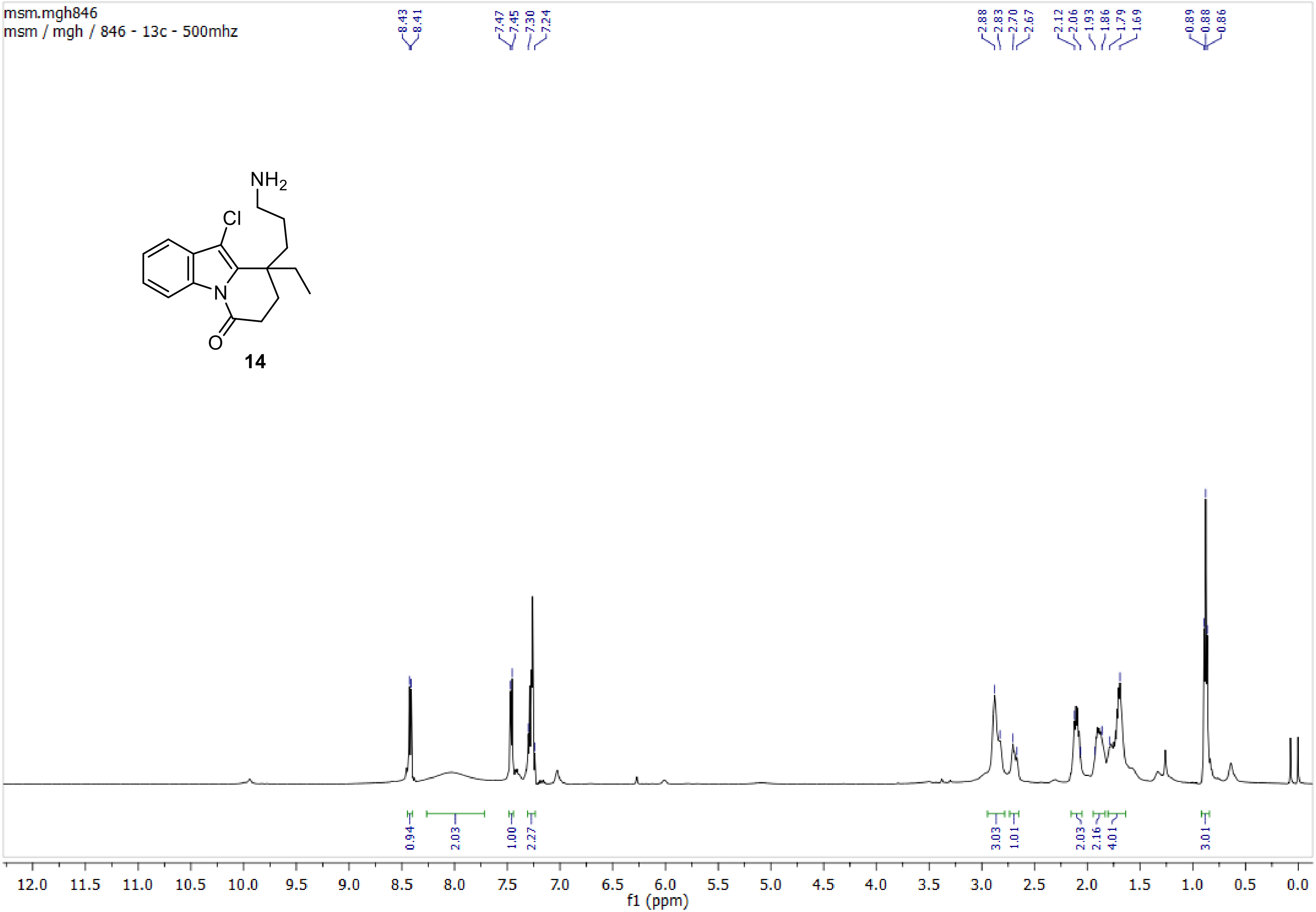
msm.mgh846
msm / mgh / 846 - 13c - 500mhz
8.43
8.41
7.47
7.45
7.30
7.24
2.88
2.83
2.70
2.67
2.12
2.06
1.93
1.86
1.79
1.69
0.89
0.88
0.86
NH2
Cl
N
O
14
0.94
2.03
1.00
2.27
3.03
1.01
2.03
2.16
4.01
3.01
12.0
11.5
11.0
10.5
10.0
9.5
9.0
8.5
8.0
7.5
7.0
6.5
6.0
5.5
5.0
4.5
4.0
3.5
3.0
2.5
2.0
1.5
1.0
0.5
0.0
f1 (ppm)

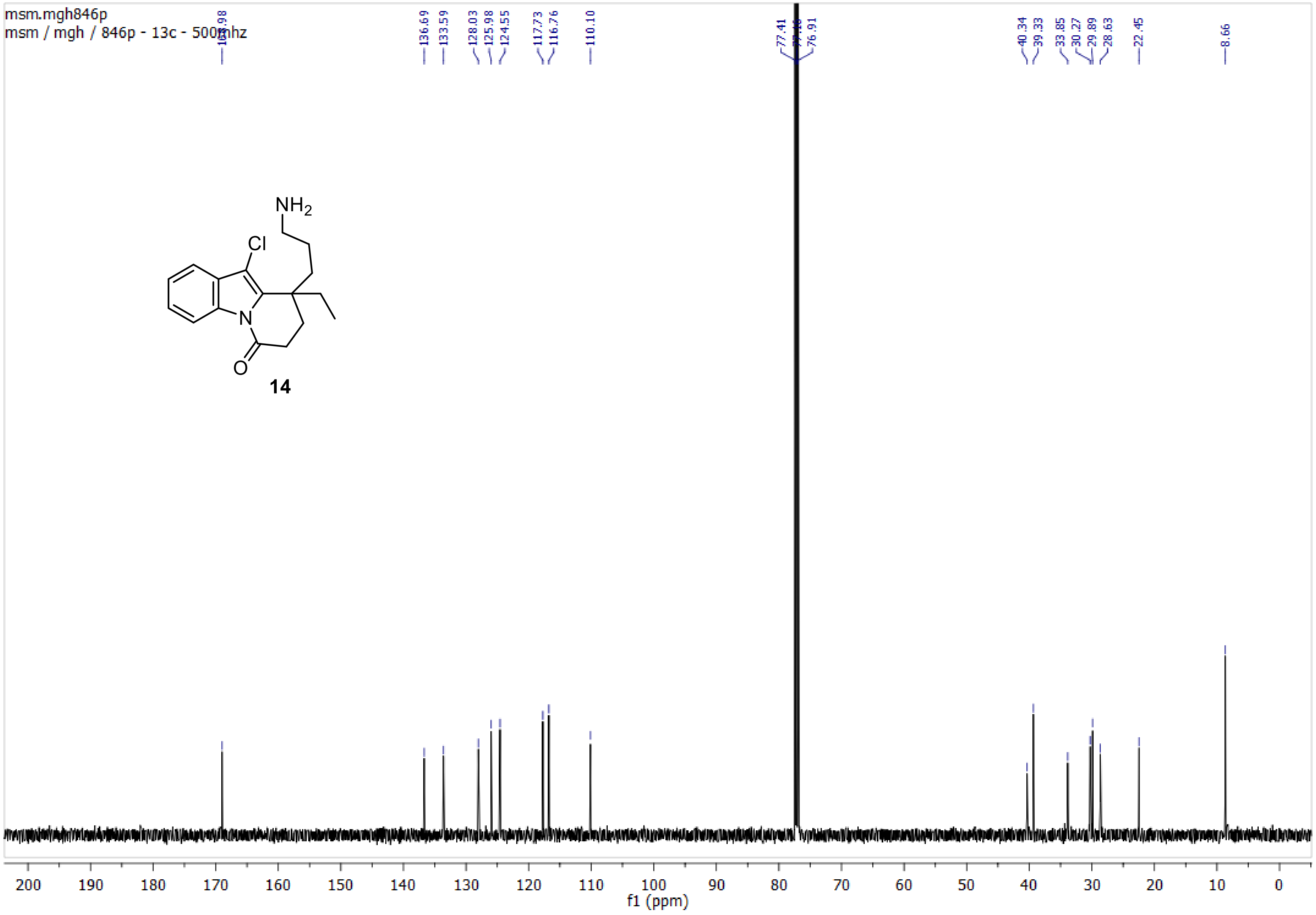
msm.mgh846p
msm / mgh / 846p - 13c - 500mhz
136.69
133.59
128.03
125.98
124.55
117.73
116.76
110.10
77.41
76.91
40.34
39.33
33.85
30.27
29.89
28.63
22.45
8.66
NH2
Cl
N
O
14
200
190
180
170
160
150
140
130
120
110
100
90
80
70
60
50
40
30
20
10
0
f1 (ppm)

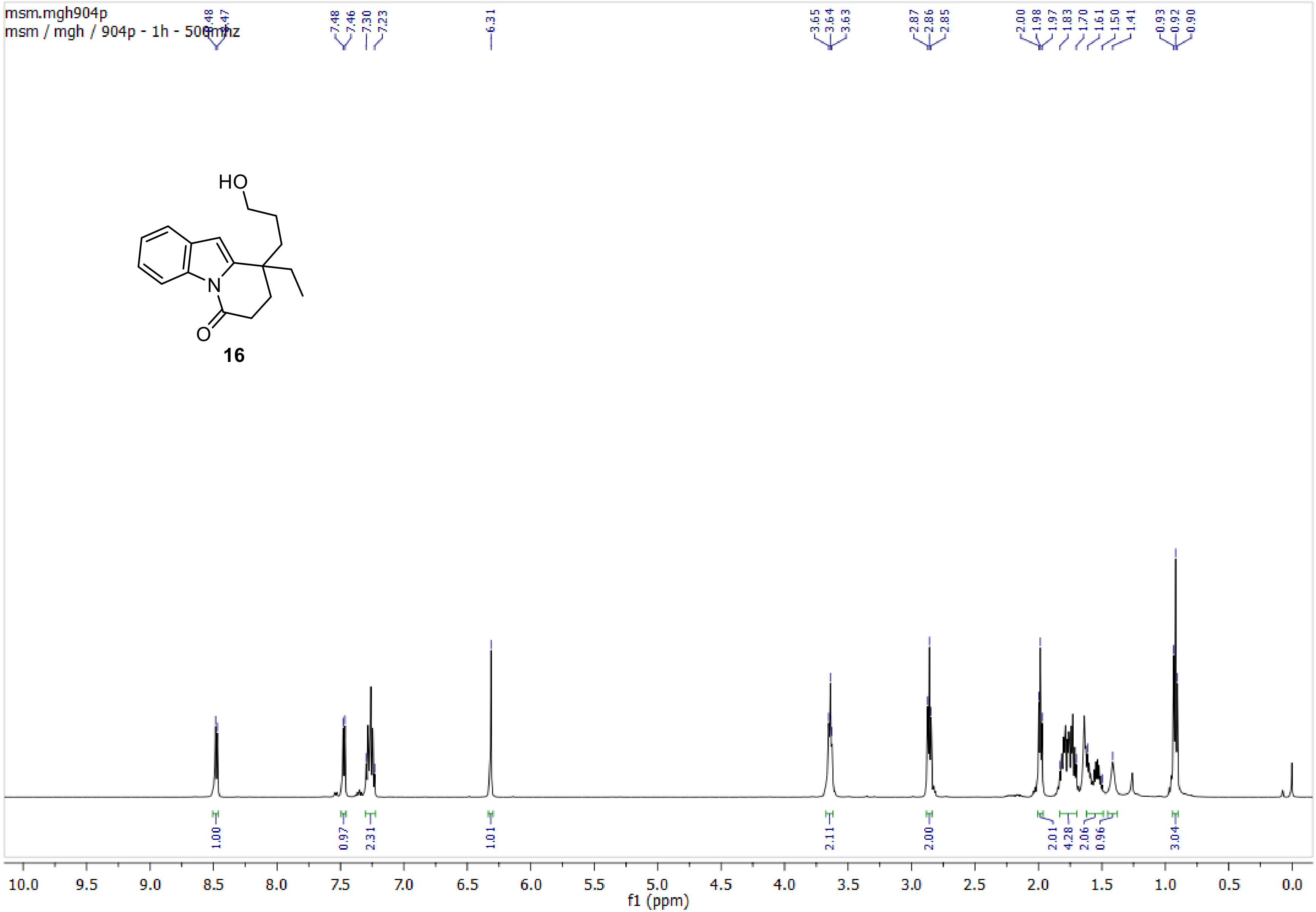
msm.mgh904p
msm / mgh / 904p - 1h - 500mhz
HO
N
O
16
f1 (ppm)

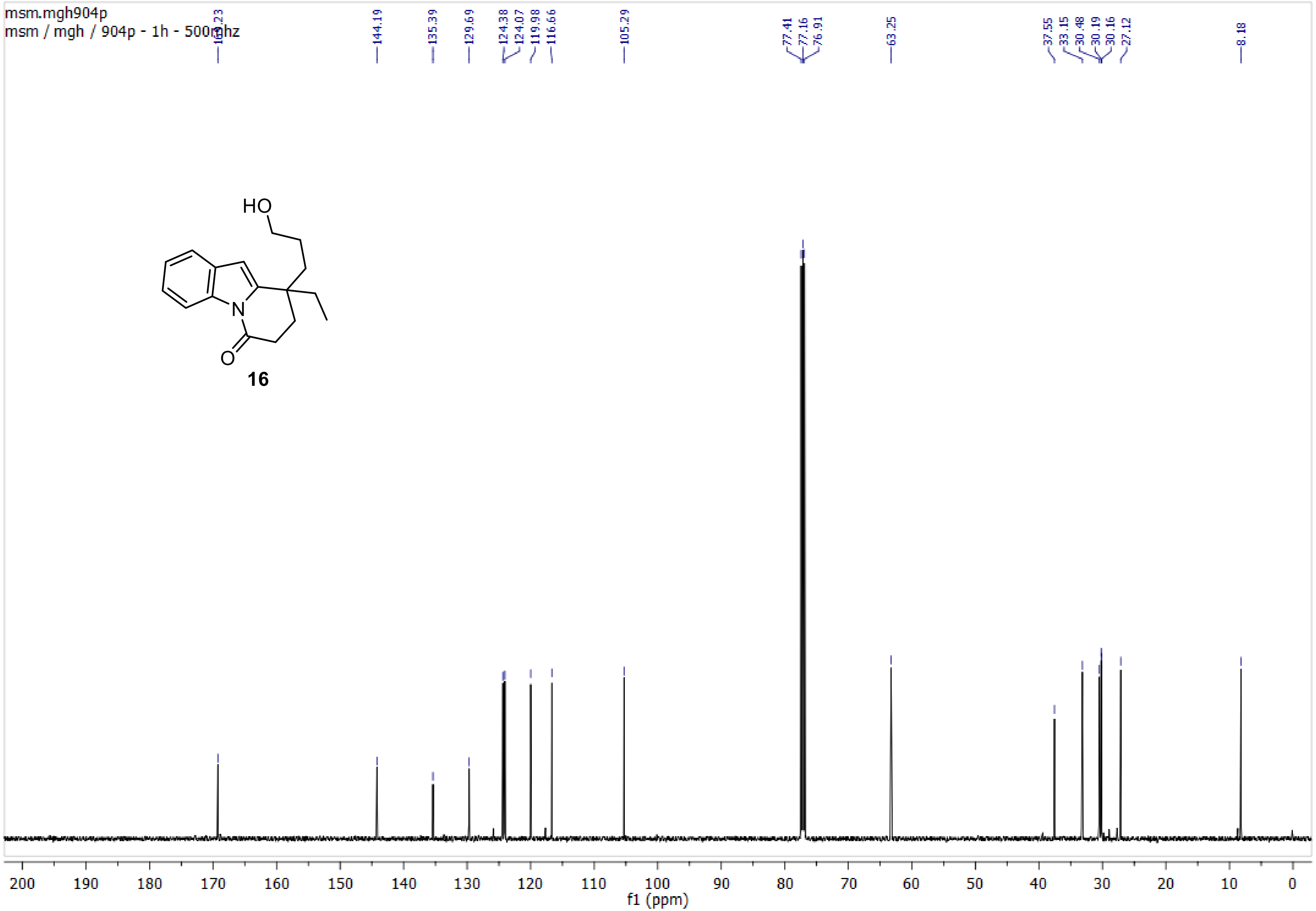
msm.mgh904p
msm / mgh / 904p - 1h - 500mhz
144.19
135.39
129.69
124.38
124.07
119.98
116.66
105.29
77.41
77.16
76.91
63.25
37.55
33.15
30.48
30.19
30.16
27.12
8.18
HO
N
O
16
f1 (ppm)
200 190 180 170 160 150 140 130 120 110 100 90 80 70 60 50 40 30 20 10 0

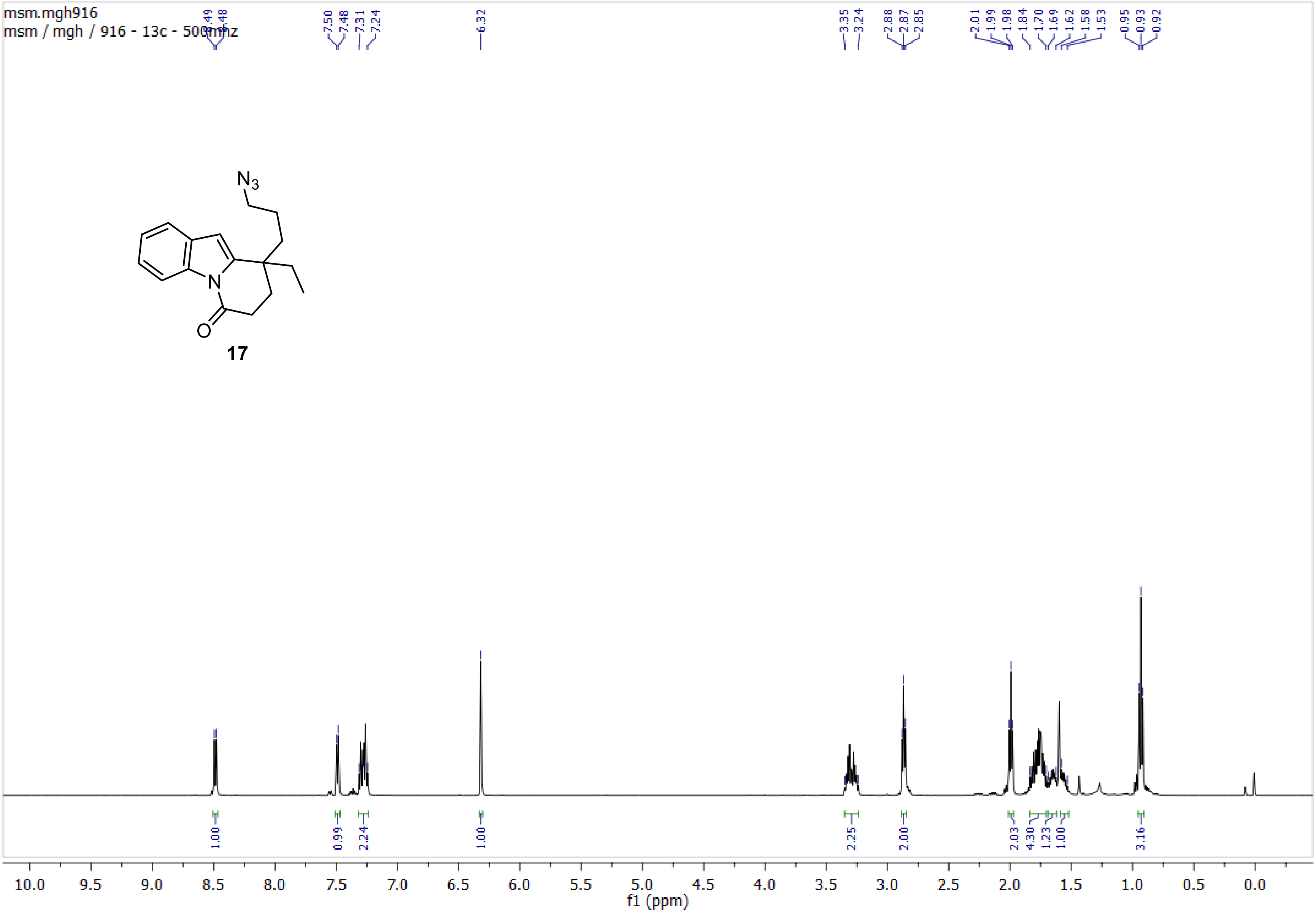
msm.mgh916
msm / mgh / 916 - 13c - 500mhz
N3
17
f1 (ppm)

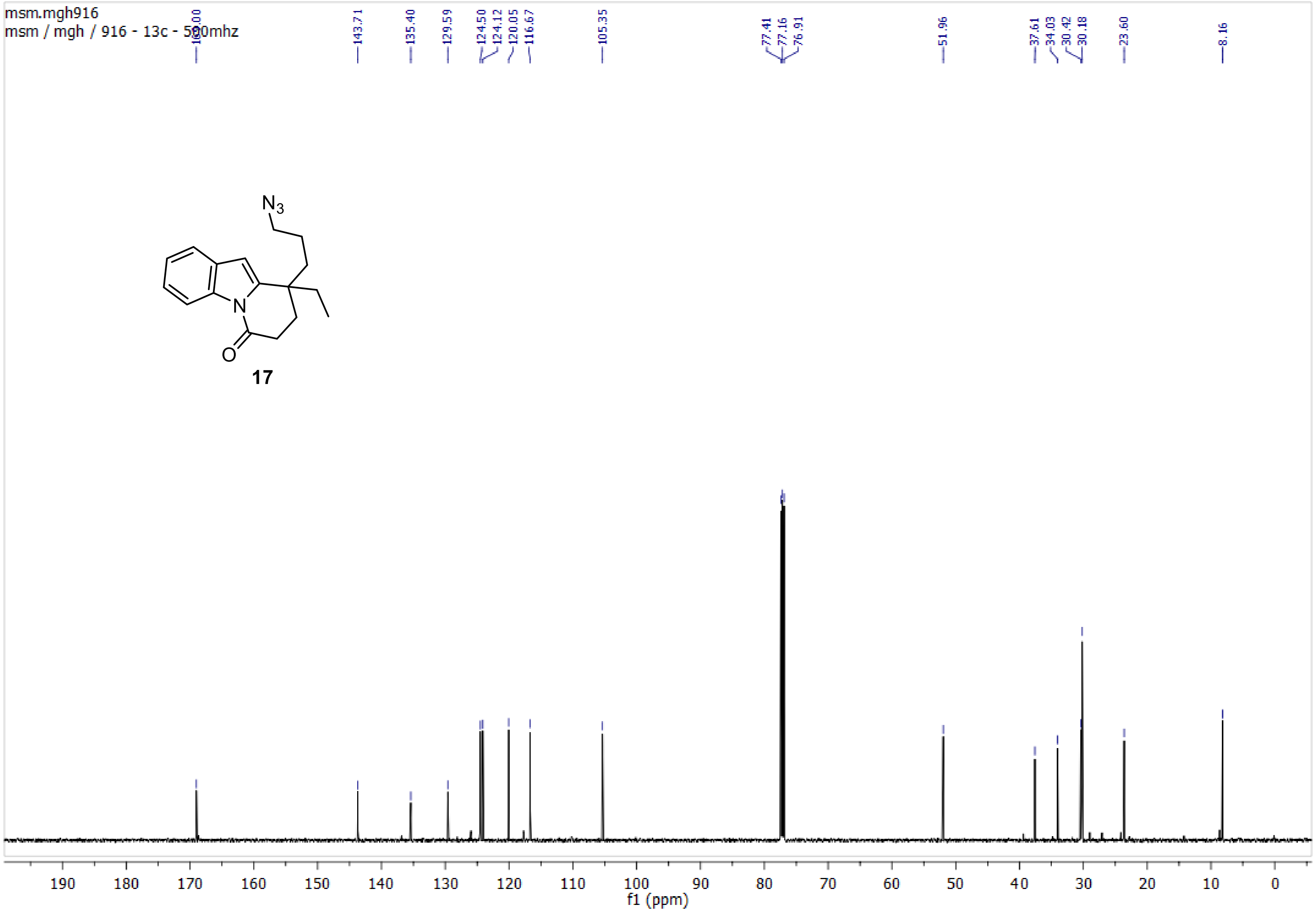

msm.mgh916
143.71
135.40
129.59
124.50
124.12
120.05
116.67
105.35
77.41
77.16
76.91
51.96
37.61
34.03
30.42
30.18
23.60
8.16
N3
N
O
17
f1 (ppm)

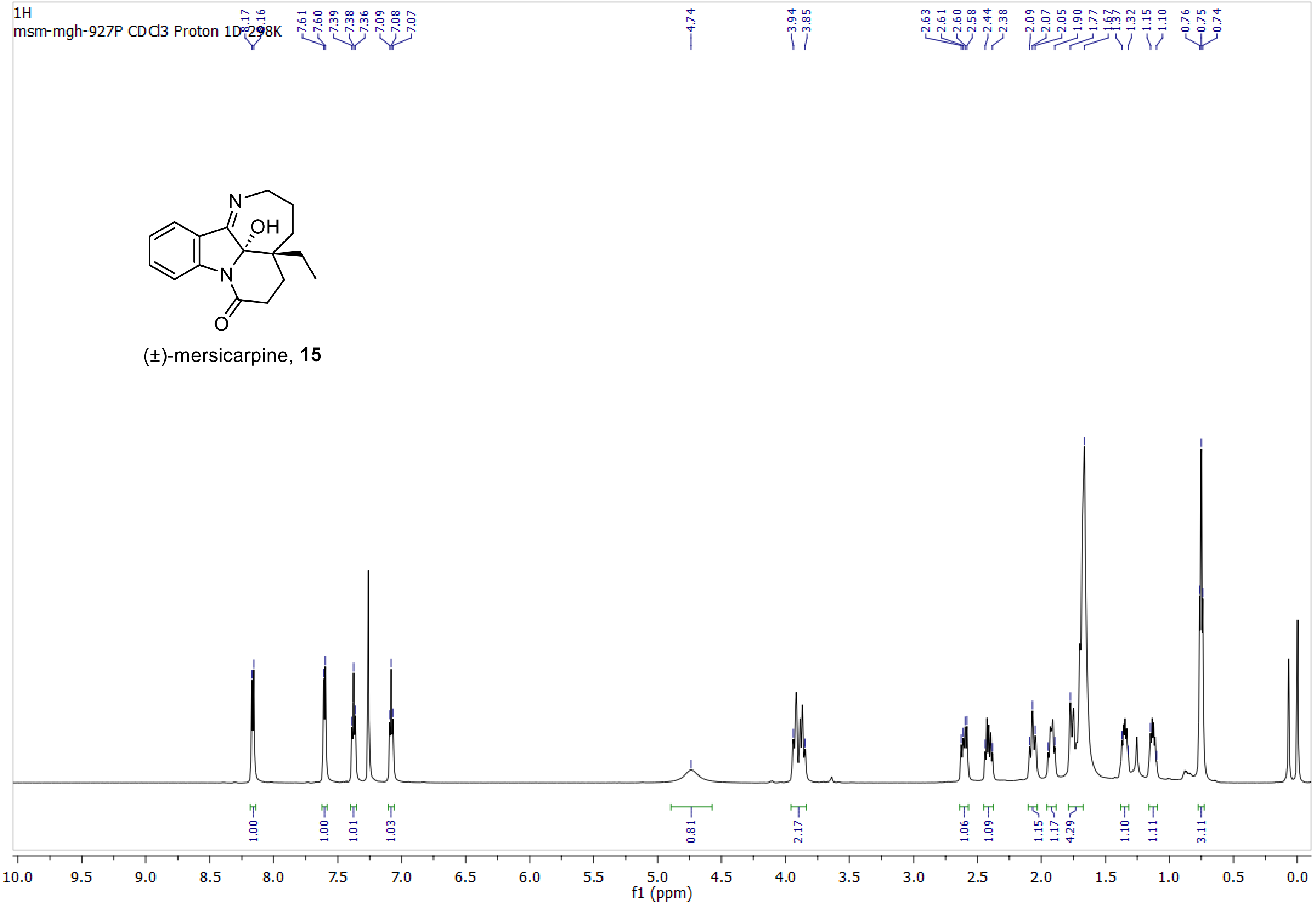
1H
msm-mgh-927P CDCl3 Proton 1D 298K
8.17
8.16
7.61
7.60
7.39
7.38
7.36
7.09
7.08
7.07
4.74
3.94
3.85
2.63
2.61
2.60
2.58
2.44
2.38
2.09
2.07
2.05
1.90
1.77
1.67
1.37
1.32
1.15
1.10
0.76
0.75
0.74
N
OH
N
O
(±)-mersicarpine, 15
1.00
1.00
1.01
1.03
0.81
2.17
1.06
1.09
1.15
1.17
4.29
1.10
1.11
3.11
10.0
9.5
9.0
8.5
8.0
7.5
7.0
6.5
6.0
5.5
5.0
4.5
4.0
3.5
3.0
2.5
2.0
1.5
1.0
0.5
0.0
f1 (ppm)

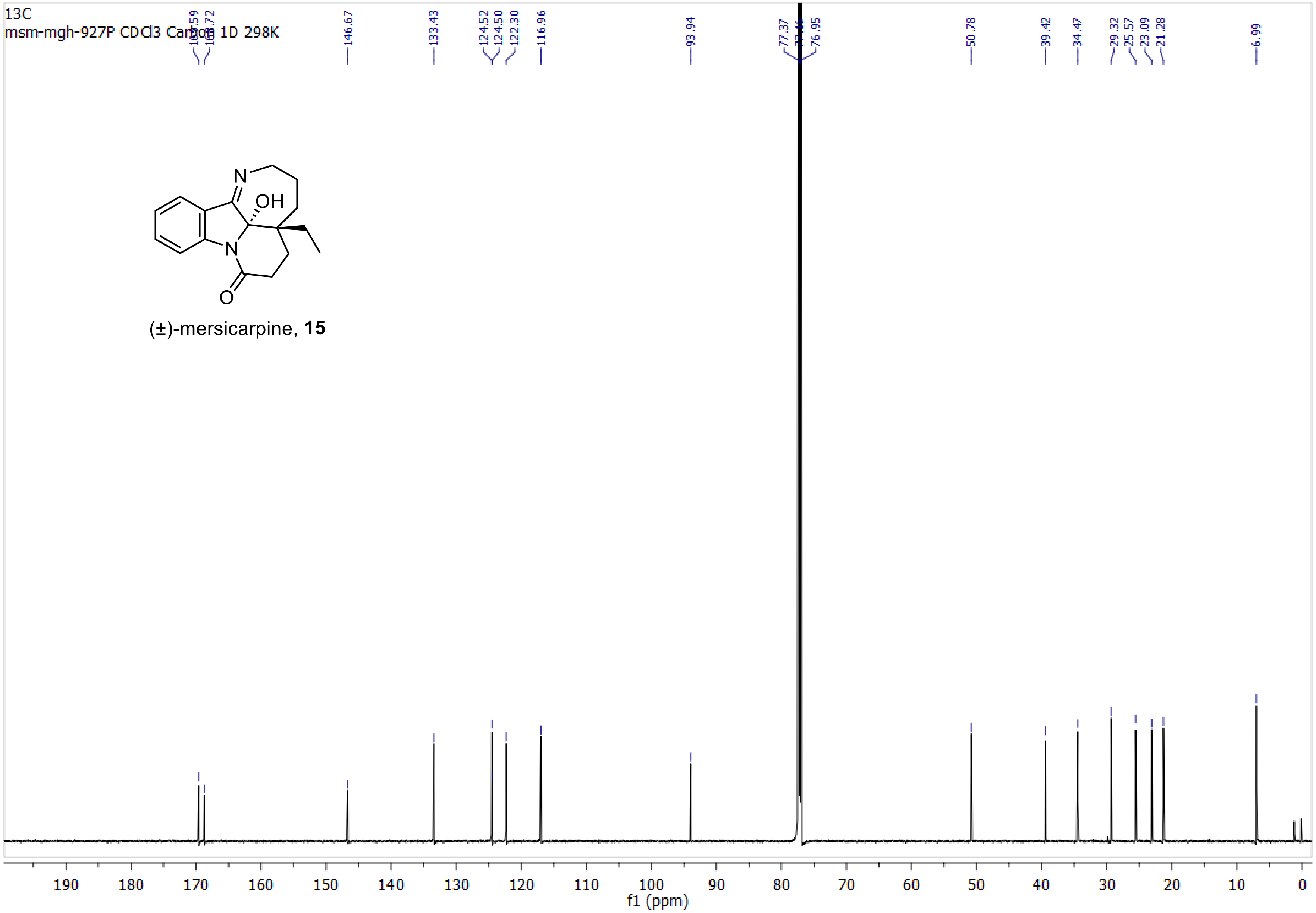
13C
msm-mgh-927P CDCl3 Carbon 1D 298K
146.67
133.43
124.52
124.50
122.30
116.96
93.94
77.37
76.95
50.78
39.42
34.47
29.32
25.57
23.09
21.28
6.99
N
OH
N
O
(±)-mersicarpine, 15
190 180 170 160 150 140 130 120 110 100 90 80 70 60 50 40 30 20 10 0
f1 (ppm)
